# MicroRNA-196b inhibits late apoptosis of pancreatic cancer cells by targeting CADM1

**DOI:** 10.1038/s41598-017-11248-3

**Published:** 2017-09-13

**Authors:** Hong-Ling Wang, Rui Zhou, Jing Liu, Ying Chang, Shi Liu, Xiao-Bing Wang, Mei-Fang Huang, Qiu Zhao

**Affiliations:** 1 Department of Gastroenterology, Zhongnan Hospital of Wuhan University, Wuhan, 430071 P.R. China; 2 The Hubei Clinical Center and Key Laboratory of Intestinal and Colorectal Diseases, Wuhan, 430071 P.R. China

## Abstract

Pancreatic cancer (PC), as the leading cause of cancer death worldwide, is one of the deadliest tumors with a very low 5-year survival rate. Therefore, it is urgent to seek new biomarkers of PC for more accurate and reliable treatments. To identify the differentially expressed miRNAs (DEM) in PC tissues, we performed the systematic microarray and qRT-PCR analyses. We found miR-196b was the top dysregulated DEM in PC tissues as compared with the corresponding adjacent tissues, and positively correlated with poor differentiation, tumor size, lymphatic invasion and TNM stage. Furthermore, the late apoptosis rate was significantly reduced, while the cell proliferation was increased in PANC-1 and ASPC-1 cell-lines after treatment with miR-196b mimics. The qRT-PCR and Western blot analysis demonstrated that the level of CADM1 in PANC-1 cells response to the alteration of miR-196b. Moreover, blockade of CADM1 could decrease the late apoptosis in PANC-1 cells as up-regulated by inhibition of miR-196b. Finally, luciferase report assay confirmed that CADM1 was the direct target gene of miR-196b. Overexpression of miR-196b in PC tissues can increase the late apoptosis of pancreatic cancer cells by targeting CADM1. These findings suggested miR-196b is a potential target for diagnosis and therapeutics of human pancreatic cancer.

## Introduction

Pancreatic cancer (PC), the 7^th^ leading cause of cancer death worldwide, is one of the deadliest tumors with a very low 5-year survival rate (ranges from 2% to 9%)^1, 2^. The incidence of PC in China has been increasing rapidly during past decades^3,4]^. The understanding of PC has been improved, but the prognosis is still very poor, which mainly results from delayed diagnosis, lack of curative treatments, and non-specific symptoms^5–7^. Therefore, it is urgent to seek new biomarkers of PC for more accurate and reliable treatments.

Recently, there are several elements such as smoking, genetics, and environment factors have been identified as risk factors of PC^5, 6, 8^. However, the exact causes of PC remain unclear. MicroRNAs (miRNAs), a group of non-coding RNAs, are involved in several biological behaviors (e.g., apoptosis, proliferation, and differentiation) through inhibiting the translation or degrading the target mRNAs^9, 10^. Moreover, miRNAs have been reported to be involved in various diseases, especially cancers^11–13^. Several studies have reported many miRNAs (e.g., miR-21, miR-25, miR-155, and miR-210) were dysregulated in PC, but the clinical value and the exact role in pathogenesis of PC were still not clear^14, 15^. Therefore, systematically identifying differentially expressed miRNAs, to account for the pathogenesis of PC and be associated with clinical characteristics of PC, will provide more evidence for the diagnosis and treatment of PC.

In this study, we identified the differentially expressed miRNAs in PC tissues, find out the correlation of these miRNAs with characteristics of PC patients, and investigated the role of miR-196b in regulating apoptosis and proliferation in PANC-1 and ASPC-1 cells. Our results indicated miR-196b is a potential target to diagnosis and therapeutics of pancreatic cancer.

## Results

### Profiling of differentially expressed miRNAs between pancreatic cancer tissues and adjacent tissues

**Figure 1:**
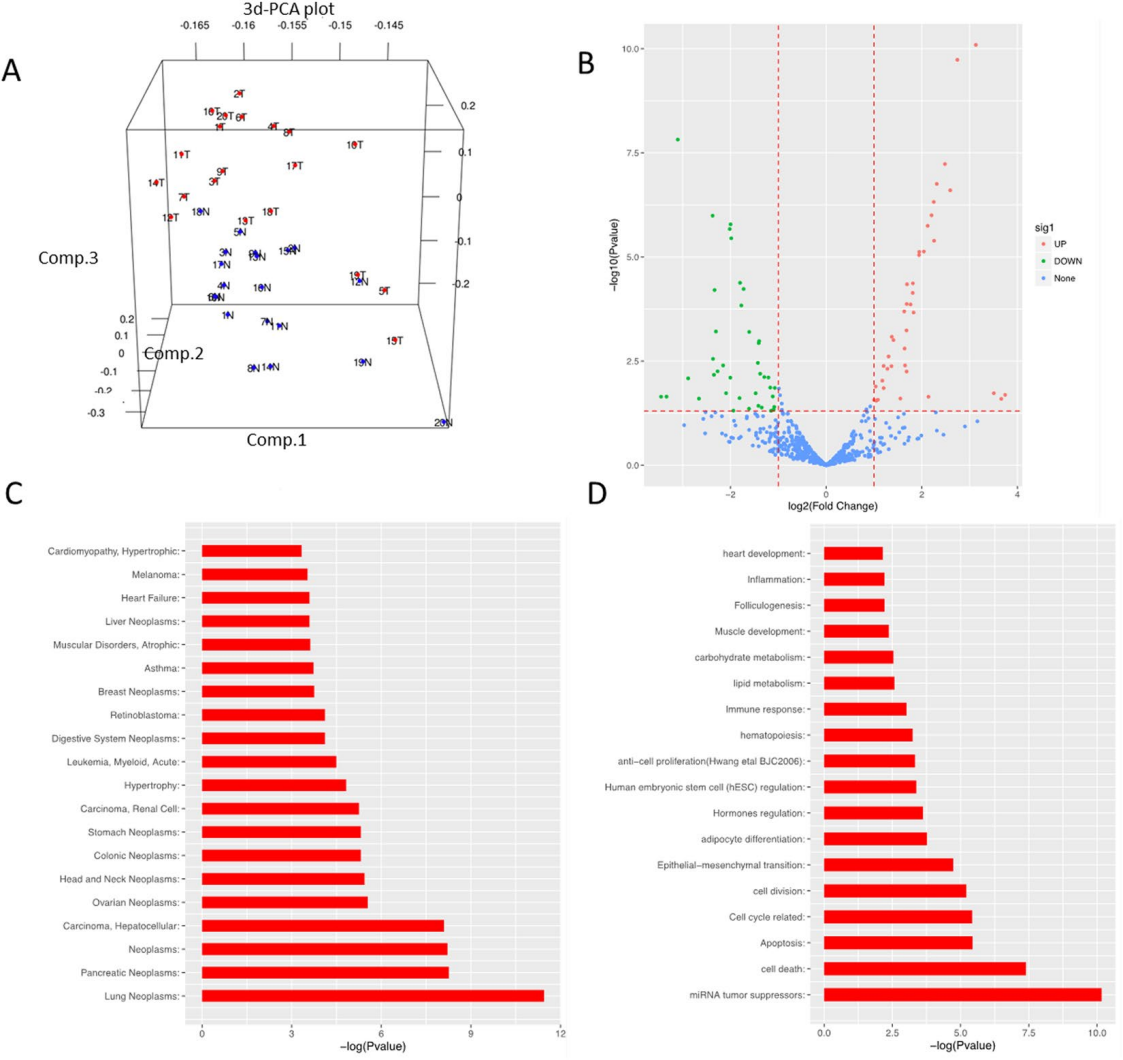
Analysis for miRNA profiles of pancreatic cancer (PC) and adjacent tissues (AT). (**A**) The 3d PCA (Principal Components Analysis) plot of 20 pairs of PC and adjacent tissues. (Comp.1, Comp.2 and Comp.3 stand for three principal components respectively) (**B**) The volcano plot of the differentially expressing miRNAs, up-regulated miRNAs represent with red circles; down-regulated miRNAs represent with blue circles. (**C**) The barplot of enrichment analysis of DEG miRNAs on Disease (HMDD2). (**D**) The barplot of enrichment analysis of DEG miRNAs on Gene Ontology (GO).

To find out candidate miRNAs associated with PC, 20 paired pancreatic cancer tissues and adjacent tissues were collected during resections, and were assessed by NanoString nCounter Human miRNA assay. The demographic characteristics of these patients were shown in Table 1. First, the discrete pattern of miRNAs in pancreatic cancer tissues and adjacent tissues was examined by Principal Component Analysis (PCA), and the 3-dimensional PCA (3d-PCA) plot showed that the expression profiles of miRNAs clustered into two separate groups, which was consistent with the source of tissues (Fig. 1A). In volcano plot, It was demonstrated that 39 miRNAs in pancreatic cancer tissues were significantly down-regulated, and 40 up-regulated compared with adjacent tissues (Fig. 1B, Table S1).

**Table 1:** Demographic characteristics of 20 PC patients detected in microarray.

Parameters	PC
Age (Years)	62 (54–69)
Gender (Female)	13 (65%)

Data distribution was descripted by median (25^th^–75th quarters) for continuous variables and count (percentage) for categorical variables; PC, pancreatic cancer.

Next, we identified the common character among these differently expressed miRNAs by enrichment analysis. According to the database of miRNA related diseases, Human microRNA Disease Database 2 (HMDD2), we found that these DEMs were mainly associated with cancers, such as lung neoplasms, and pancreatic neoplasms (Fig. 1C). Besides, Gene Ontology (GO) annotation was also used to analyze the common functions of these DEMs. Then we found that DEMs focused in and several GO terms associated with cell proliferation, cell apoptosis, and cell death (Fig. 1D). Detailed information of enriched terms was presented in Table S2.

### Validation of differentially expressed miRNAs in pancreatic cancer tissues

**Figure 2:**
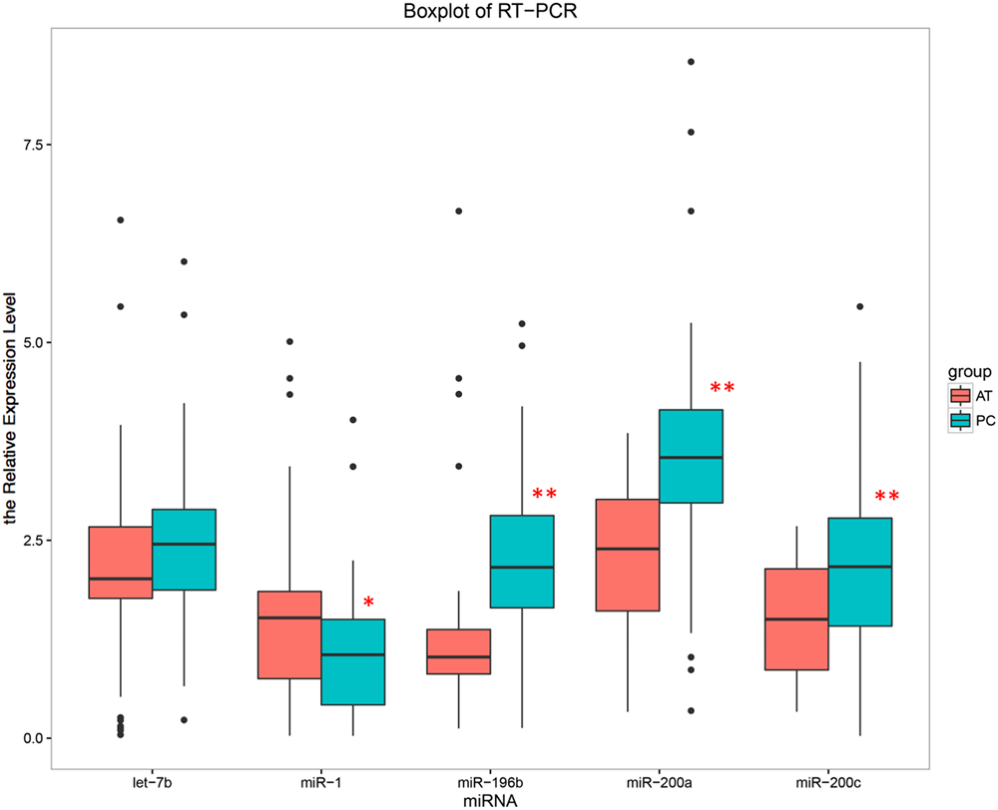
The boxplot of expression level of top5 DEG miRNAs in 80 pairs of pancreatic cancer (PC) and adjacent tissues (AT). The distribution of miRNA expression in PC represents as blue box, while that in AT represents as red box. Significance of the comparison was determined by Wilcoxon signed-rank sum test, **P value < 0.01; *P value < 0.05.

As shown in Table 2, we used the 5 top differentially expressed miRNAs (sorted by P value: miR-200c, miR-196b, miR-1, miR-200a, let-7b) in pancreatic cancer tissues compared with adjacent tissues in microarray as candidate miRNAs. In order to validate the difference for the expression level of these miRNAs between pancreatic cancer tissues and adjacent tissues, 80 paired pancreatic and adjacent tissues were examined (20 from the microarray cohort and 60 from the second cohort) with the demographic, clinical and pathological features presented in Table 3, were used to perform the qRT-PCR assay. Interestingly, we found that the expressions of miR-196b, miR-200a, and miR-200c were up-regulated, but miR-1 was down-regulated in pancreatic cancer tissues compared with adjacent tissues by Wilcoxon signed rank sum test (Fig. 2). However, no statistical difference of let-7b was observed between pancreatic cancer tissues and adjacent tissues. Moreover, miR-196b was observed to be the most significantly differentially expressed one among these miRNAs.

**Table 2:** Top 5 differentially expressing miRNAs in microarray.

miRNA	logFC	AveExpr	*P* Value	Sig
hsa-miR-200c	3.13	10.62	8.06E-11	UP
hsa-miR-196b	2.74	6.42	1.84E-10	UP
hsa-miR-1	-3.10	4.35	1.51E-08	DOWN
hsa-miR-200a	2.48	9.18	5.86E-08	UP
hsa-let-7b	2.31	12.96	1.76E-07	UP

Significance of the comparison was completed by limma package in R. Top 5 differentially expressing miRNAs (according to abs (logFC)) were represent with the logFC, AveExpr, *P* Value and Sig. The logFC, log2(fold change); AveExpr, average of expression level; Sig: UP. Up-regulated, Down, Down-regulated.

**Table 3:** Demographic, clinical and pathological characteristic of PC patients in validation stage.

Parameters	PC
Sample size	80 (100%)
Age (years)	60.50 (51.25–68.00)
Gender (male/female)	46/34
**Differentiation**	
Well	20 (25%)
Moderate	39 (48.8%)
Poor	21 (26.3%)
**Tumor Size (cm)**	
≧3	44 (55%)
<3	36 (45%)
**Lymphatic invasion**	
Yes	43 (53.8%)
No	37 (46.3%)
**Distant metastases**	
Yes	3 (3.8%)
No	77 (96.3%)
**TNM stage**	
I	16 (20%)
II	27 (33.8%)
III	34 (42.5%)
IV	3 (3.8%)

Data distribution was descripted by median (25^th^–75^th^ quarters) for continuous variables and count (percentage) for categorical variables; PC, pancreatic cancer.

### The correlation of miRNA expression and clinicopathological features of PC patients

Next, we investigated whether the levels of these differentially expressed miRNAs were associated with characteristics of PC patients. As shown in Table 4, miR-196b was found to be positively associated with poor differentiation, tumor size, lymphatic invasion and higher TNM stage, indicated its critical role in PC progression. In addition, let-7b and miR-200c were demonstrated to be positively correlated with poor differentiation, and miR-200a was positively associated with tumor size, while miR-1 was negatively correlated with poor differentiation.

**Table 4:** Correlation analysis between the expressions of miRNAs and clinicopathological features of PC patients.

		let-7b	miR-1	miR-196b	miR-200a	miR-200c
Age	Coefficient R	−0.014	0.029	0.058	−0.017	0.042
	*P* value	0.905	0.796	0.608	0.883	0.713
Gender (male)	Coefficient R	0.044	0.196	0.055	0.073	−0.003
	*P* value	0.700	0.081	0.626	0.518	0.977
Differentiation	Coefficient R	0.277*	−0.276*	0.360**	0.068	0.290**
	*P* value	**0.013**	**0.013**	**0.001**	0.549	**0.009**
Tumor size	Coefficient R	0.141	−0.111	0.402**	0.223*	0.084
	*P* value	0.211	0.327	**<0.001**	**0.047**	0.460
Lymphatic invasion	Coefficient R	0.185	−0.046	0.326**	−0.031	0.027
	*P* value	0.100	0.684	**0.003**	0.785	0.815
Distant metastases	Coefficient R	0.150	−0.078	0.004	−0.141	0.090
	*P* value	0.185	0.490	0.970	0.212	0.429
TNM stage	Coefficient R	0.220	−0.090	0.340**	0.136	0.082
	*P* value	0.050	0.428	**0.002**	0.228	0.472

Correlation was determined by Spearman test. The spearman coefficients and *P* value were present in the table. Differentiation was numbered by 1- well differentiation; 2- moderate differentiation; 3-poor differentiation for the analysis; while TNM was numbered by 1-Stage I; 2-Stage II; 3-Stage III;4-Stage IV for the analysis. **p Value < 0.01; *p Value < 0.05; PC, pancreatic cancer.

### MiR-196b increased late apoptosis and inhibited cell proliferation of PANC-1 and ASPC-1 cells

**Figure 3:**
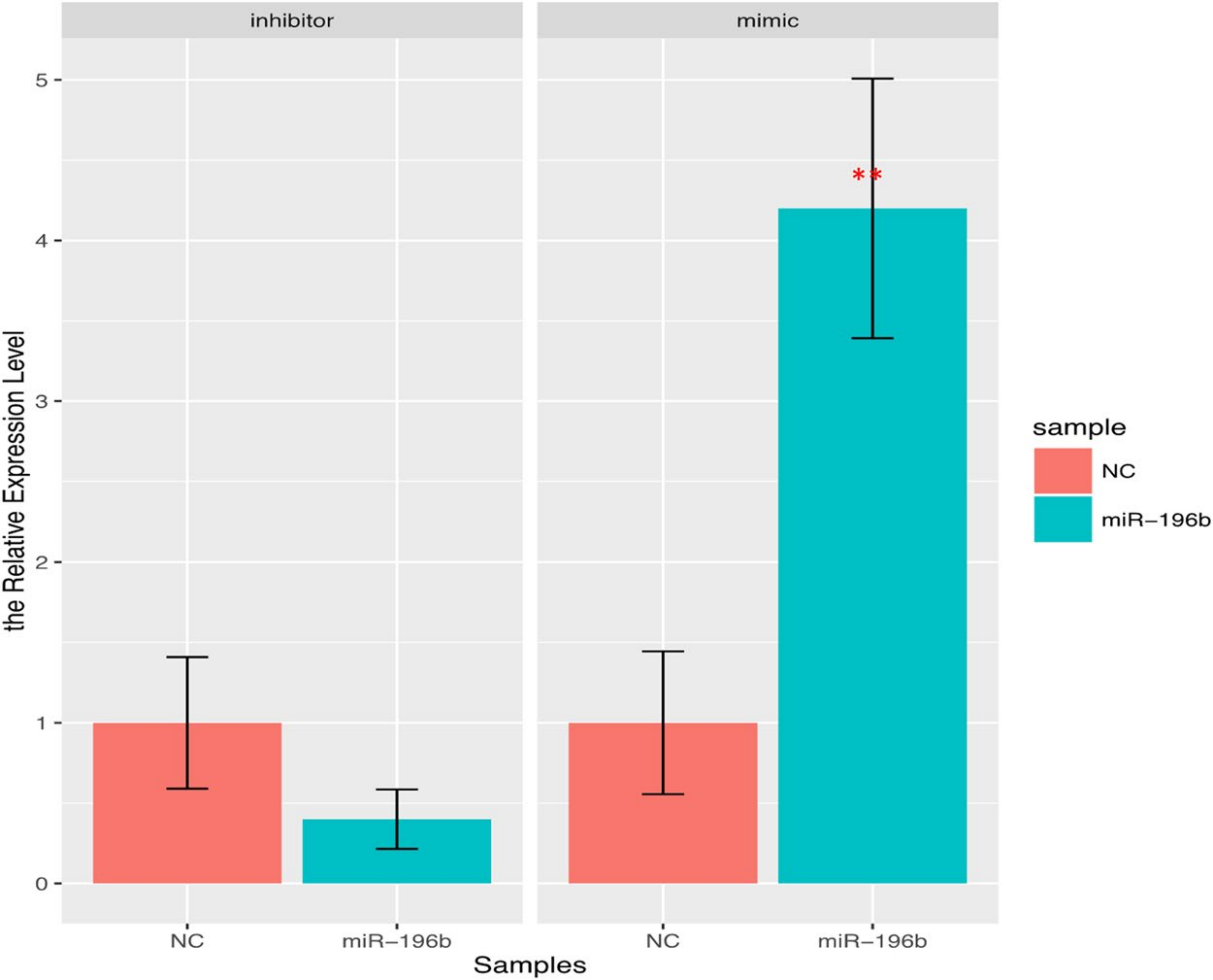
The expression level miR-196b in PANC-1 cell line after treatment with miR-196b inhibitor or mimic. The expression level of miR-196b in two interrupting systems, inhibitor and mimic, represent as blue box, while their negative controls represent as red box. Significance of the comparison was determined by t test, **P value < 0.01; *P value < 0.05.

As MiR-196b has been observed to be a candidate biomarker in diagnosis of PC and correlated with clinicopathological features, but the function of miR-196b in PC was still unknown. Therefore, we investigated the function of miR-196b in PC cells by interference experiment. Firstly, we confirmed that the expression of miR-196b was significantly decreased after transfection with miR-196b inhibitor, and was increased after transfection with miR-196b mimics compared with each negative controls (Fig. 3). These results were also confirmed in ASPC-1 cells (Fig. S1).

**Figure 4:**
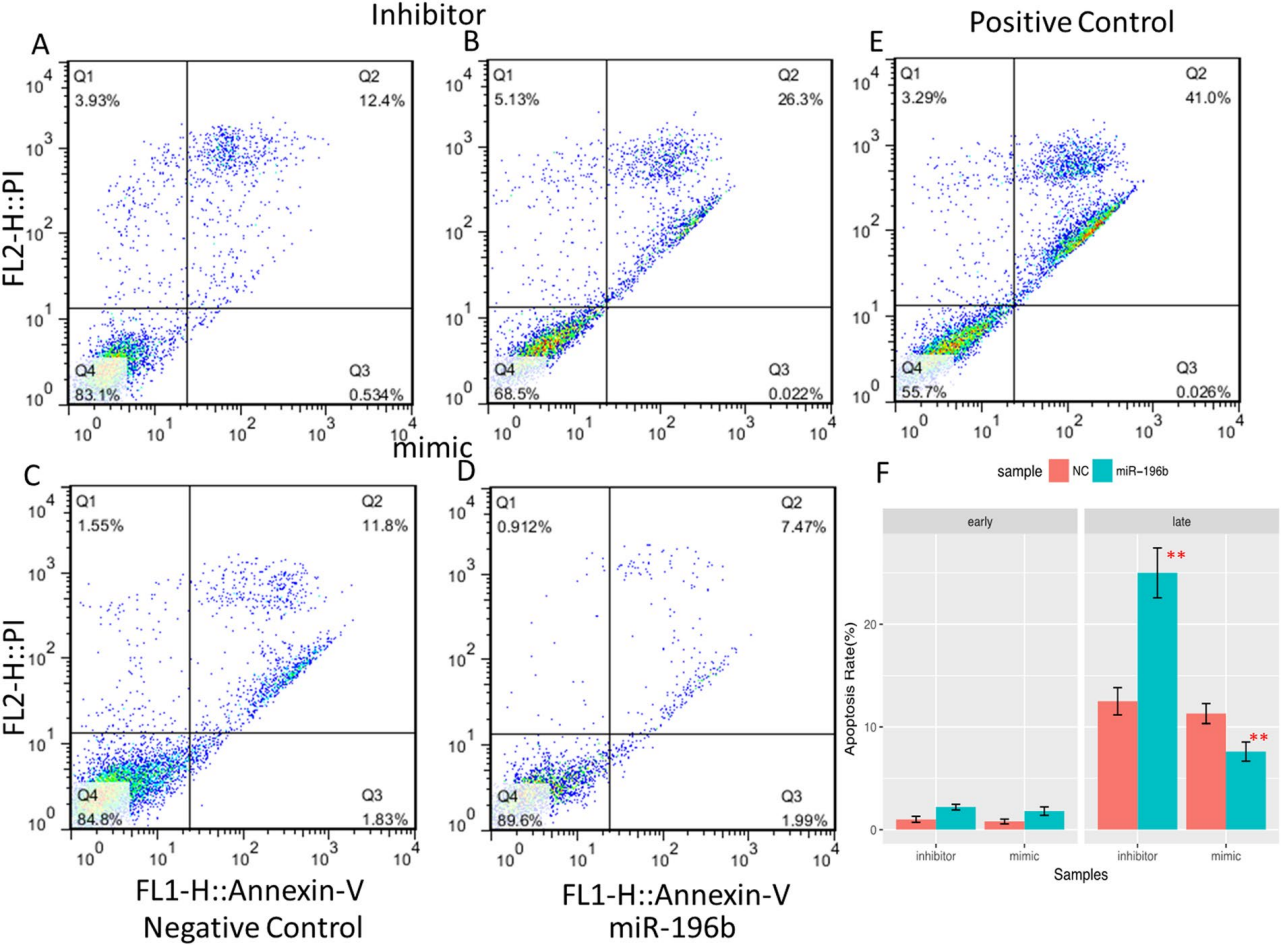
The interruption of miR-196b affected cell apoptosis by flow cytometry in PANC-1 cell line. Annexin-V FITC/PI assay was used to evaluate cell apoptosis for negative control (A) for inhibitor system and miR-196b inhibitor (**B**), negative control (**C**) for mimic system and miR-196b mimic (**D**), positive control (**E**). The lower right quadrant represents the early apoptosis, while the upper right quadrant represents the late apoptosis. The rates of early and late apoptosis were shown on barplot (**F**). The means of cell early and late apoptosis in two interrupting systems were compared with its negative control by t test, **P alue < 0.01; *P value < 0.05.

Next, we assessed cell apoptosis in PANC-1 cells by two markers, FITC annexin V and propidium iodide (PI). We found that late apoptosis rate was reduced in PANC-1 cells with treatment with miR-196b mimics, and increased with miR-196b inhibitors compared with controls (Fig. 4A–F). While there was no significant change has been detected for the early apoptosis rate after transduction of miR-196b mimics or miR-196b inhibitor into PANC-1 cells. We repeat cell apoptosis assay in ASPC-1 cells and found the similar effects of miR-196b on cell apoptosis (Fig. S2A–F).

**Figure 5:**
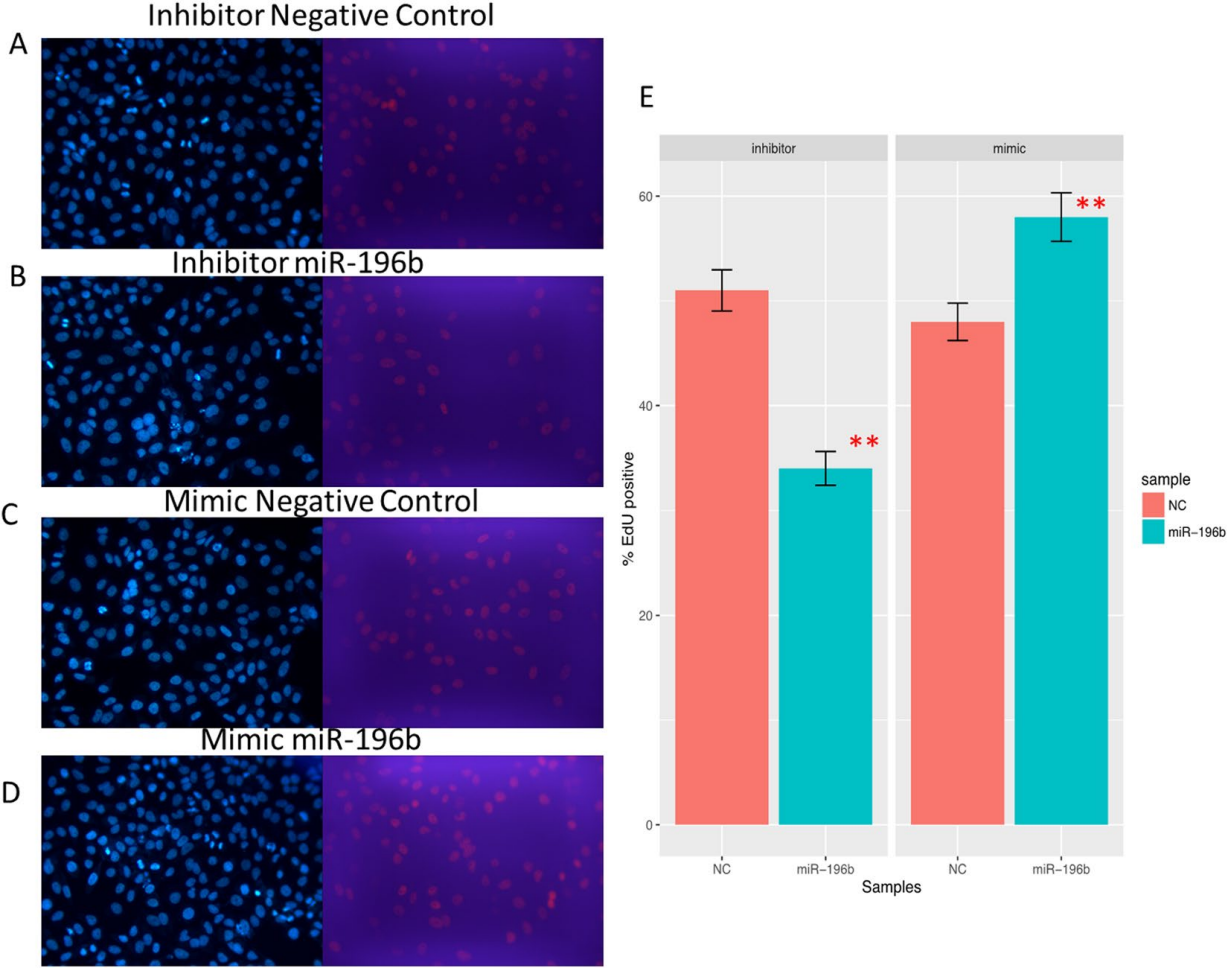
The interruption of miRNA-196b affected cell proliferation by Edu analysis in PANC-1 cell line, negative control (**A**) for inhibitor system and miR-196b inhibitor (**B**), negative control (**C**) for mimic system and miR-196b mimic (**D**). Proliferating cells were labeled after conjugated reaction of Apollo dye and EdU (red), while the total cells represent as blue (cell nuclei marked by Hoechst 33342). The rates of proliferating cells were shown on barplot (**E**) and compared with its negative control by t test, **P Value < 0.01; *P Value < 0.05.

**Figure 6:**
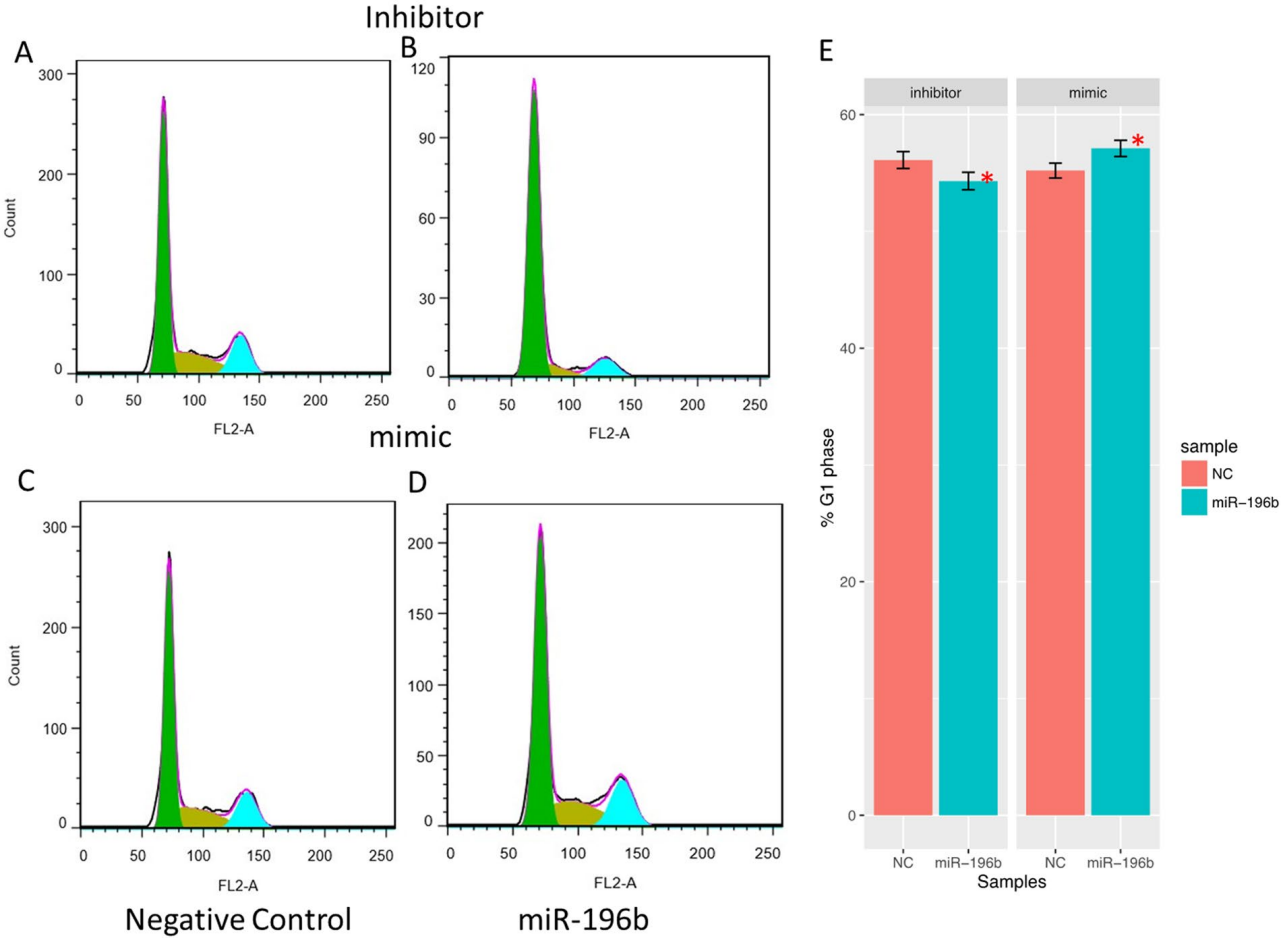
The interruption of miRNA-196b affected cell cycle by flow cytometry analysis in PANC-1 cell line, negative control (**A**) for inhibitor system and miR-196b inhibitor (**B**), negative control (**C**) for mimic system and miR-196b mimic (**D**). The percent of G1 phase cells was shown on barplot (**E**), and compared with its negative control by t test, **P Value < 0.01; *P Value < 0.05.

Then, We investigated whether miR-196b could regulate proliferation in PANC-1 cells, and found that the percentage of EdU positive cells was decreased in cells transfected with miR-196b inhibitors, while increased after transfected with miR-196b mimics compared with negative controls (Fig. 5A–E), indicating that miR-196b could facilitate proliferation in PANC-1 cells. The similar results were also found in ASPC-1 cells (Fig. S3A–E). Moreover, more percentage of cells was accelerated at G1 phase when treatment with miR-196b inhibitor, and which was decreased after treatment with miR-196b mimics in PANC-1 cells (Fig. 6A–E).

### MiR-196b down-regulates the expression of CADM1 in PANC-1 and ASPC-1 cells

**Figure 7:**
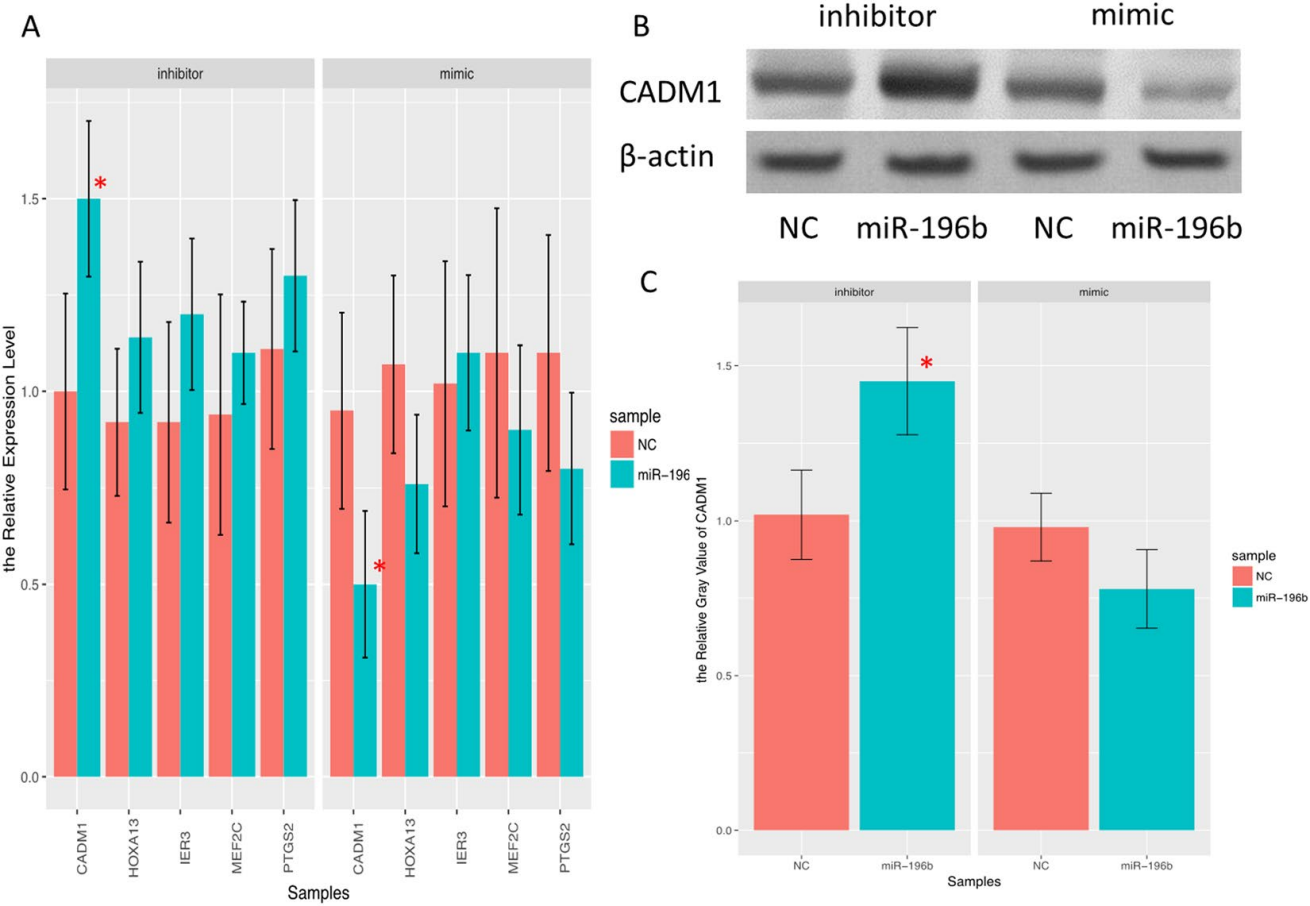
The barplot of mRNA expression level of 5 candidate targets of miR-196b in PANC-1 cell line, inhibitor miR-196b and its negative control, mimic miR-196b and its negative control. (**A**) Significance of the comparison was determined by t test, **P Value < 0.01; *P Value < 0.05. The relative protein expression of CADM1 in PANC-1 cell treated with inhibitor miR-196b and control, mimic miR-196b and control by Western blot (**B**).

Using TargetScan analysis (http://www.targetscan.org/) (Table 5), We found 5 candidate genes (CADM1, HOXA13, IER3, MEF2C, PTGS2) targeting miR-196b. It was demonstrated that only the mRNA level of CADM1 was significantly increased in PANC-1 cells after treatment with miR-196b inhibitor, and was decreased after treatment with miR-196b mimics (Fig. 7A). These results from ASPC-1 cells confirmed the finding in PANC-1 cells. Furthermore, it suggested that HOXA13 may also response to treatment of miR-196b (Fig. S4). Consistently, miR-196b reduced the protein level of CADM1 in PANC-1 cells (Fig. 7B,C). These results suggested that CADM1 may be a direct target of miR-196b.

**Table 5:** The targets of hsa-miR-196 predicted by TargetScan.

Ortholog of target gene	Representative transcript	Total sites	8mer sites	7mer-m8 sites	7mer-A1 sites	6mer sites
MEF2C	ENST00000340208.5	1	0	0	1	0
IER3	ENST00000376377.2	1	0	0	1	0
CADM1	ENST00000452722.3	2	0	2	0	0
PTGS2	ENST00000367468.5	1	0	0	1	0
HOXA13	ENST00000222753.4	1	0	1	0	0

The targets of has-miR-196 were predicted by TargetScan.

### MiR-196b inhibited CADM1 expression by binding to its 3′UTR

**Figure 8:**
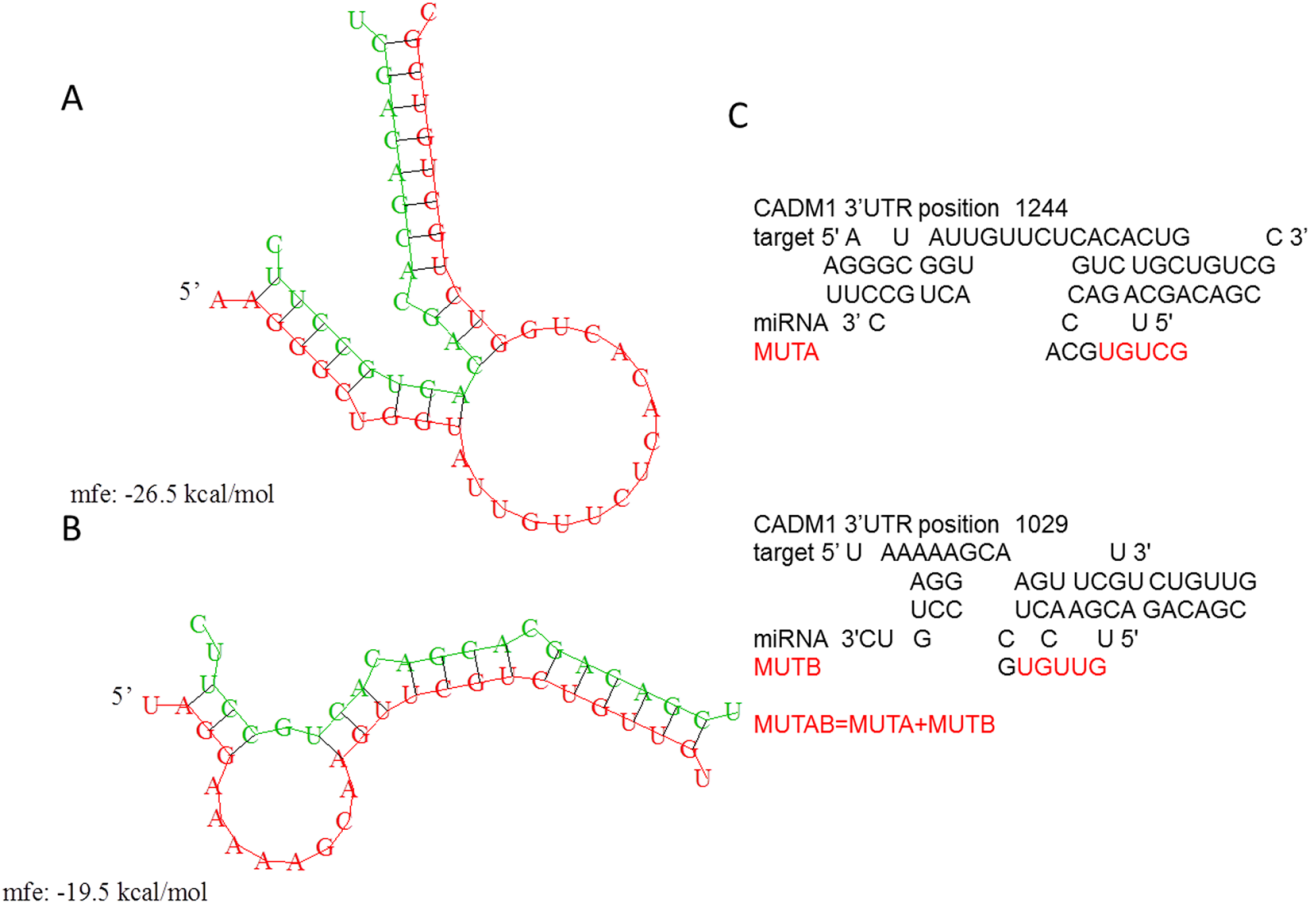
The miR-196b binding site in CADM1 3′UTR. The second structure of miRNA-3′UTR complex for the first (**A**) and second (**B**) binding sites were predicted by RNAhybrid (mfe: minimum free energy). (**C**)According to the position and sequence information of two binding sites, we developed two single mutant and one double mutant of CADM1 3′UTR, whose mutated bases were labeled in red.

**Figure 9:**
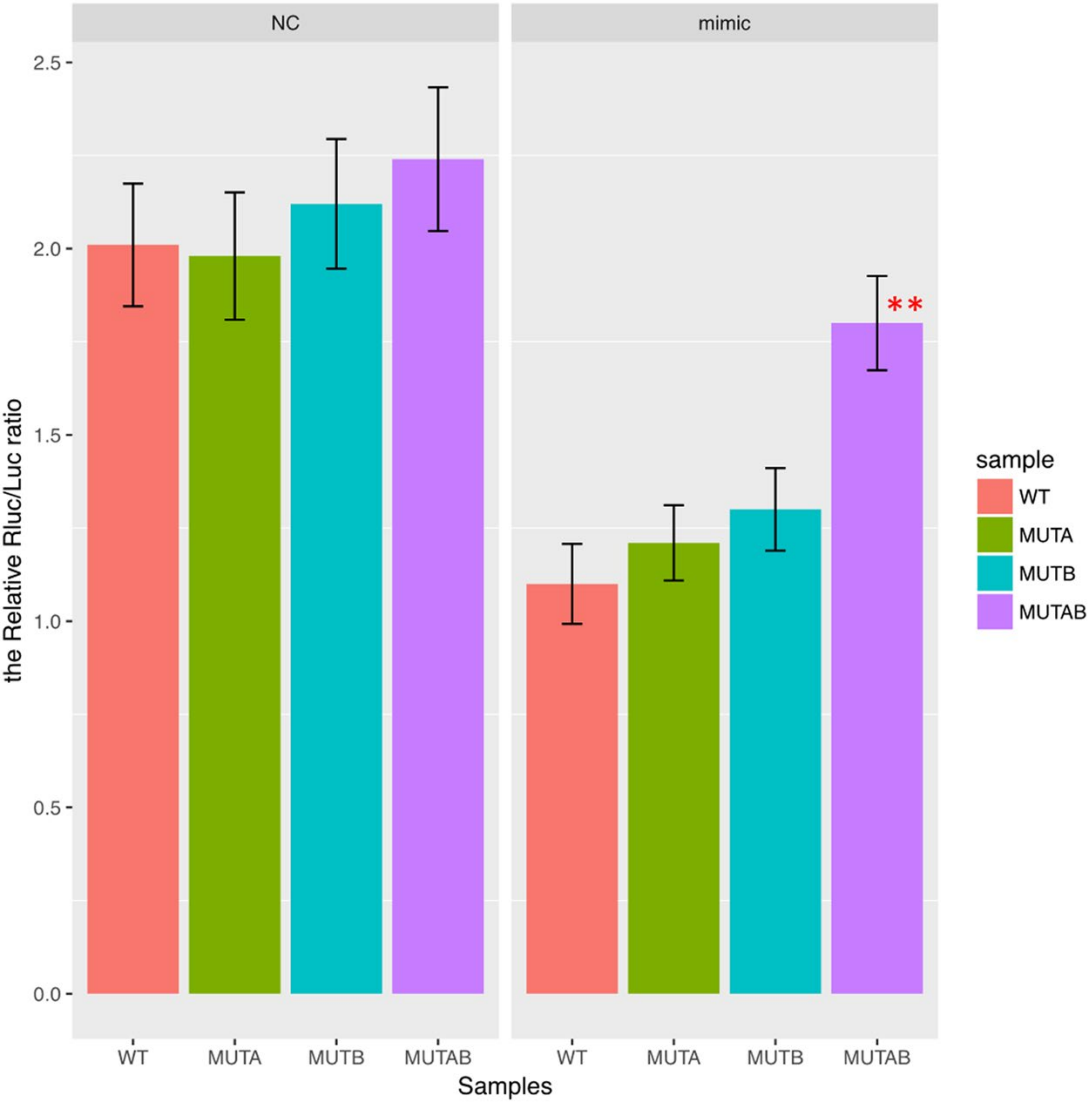
The directly binding of miR-196b to 3′UTR of CADM1 was tested by luciferase reporter assay in PANC-1 cell line. The four genotype of 3′UTR of CADM1 (wild type (WT), two single mutant (MUTA, MUTB) and double mutant MUTAB) were detected in mimic miR-196b system and its negative control. Significance of the comparison with WT 3′UTR was determined by t test. **P Value < 0.01; *P Value < 0.05.

To verify that CADM1 was the direct target gene of miR-196b, luciferase report assay was performed. By RNAhybrid software, we confirmed that there are two significant binding sites of miR-196b in 3′UTR of CADM1 (Fig. 8). Then, we constructed two single mutants at each binding site and one double mutant at both binding sites, labeled as MUTA, MUTB and MUTAB in luciferase report system (Fig. 8). We found that the luciferase reporter activity of CADM1 was significantly repressed after treatment with miR-196b mimic (Fig. 9, left panel). These results suggested that CADM1 was a direct target of miR-196b. Furthermore, we found that only signal from cotransfection of CADM1 with double mutant at both binding sites (MUTAB) had significant changes as compared to WT + miR-196b mimic, while no obvious difference to the WT + NC group (Fig. 9, right panel). It suggested that both of these two binding sites in 3′UTR of CADM1 were required for the effectively binding between miR-196b and CADM1.

### MiR-196b regulated late apoptosis through CADM1 in PANC-1 cells

**Figure 10:**
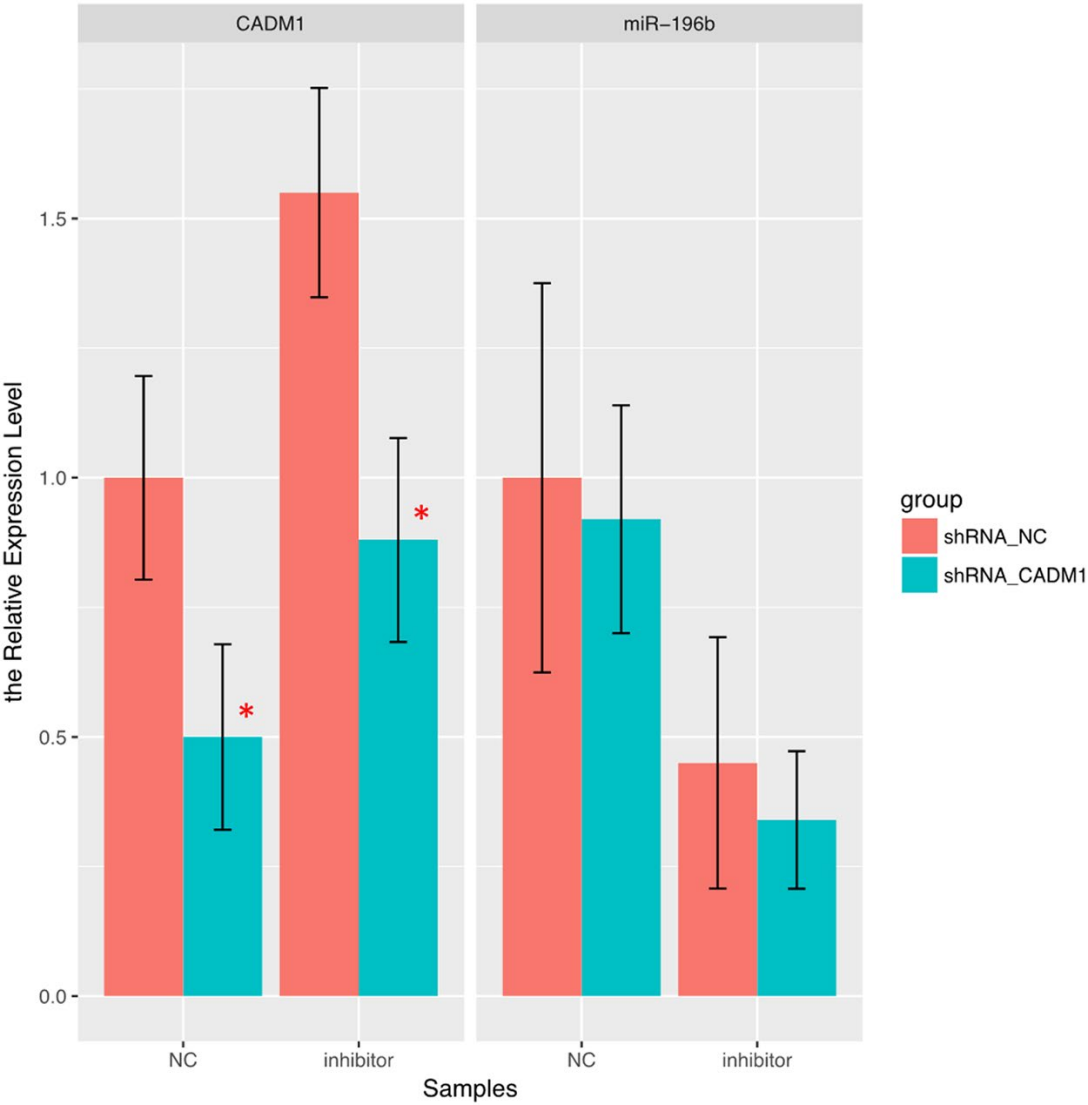
The barplot of expression level of miR-196b and CADM1 in rescue experiments in PANC-1 cell line. The CADM1 were knocked down by shRNA in PANC-1 treated with inhibitor of miR-19b and its negative control. Significance of the comparison was determined by t test, **P Value < 0.01; *P Value < 0.05.

**Figure 11:**
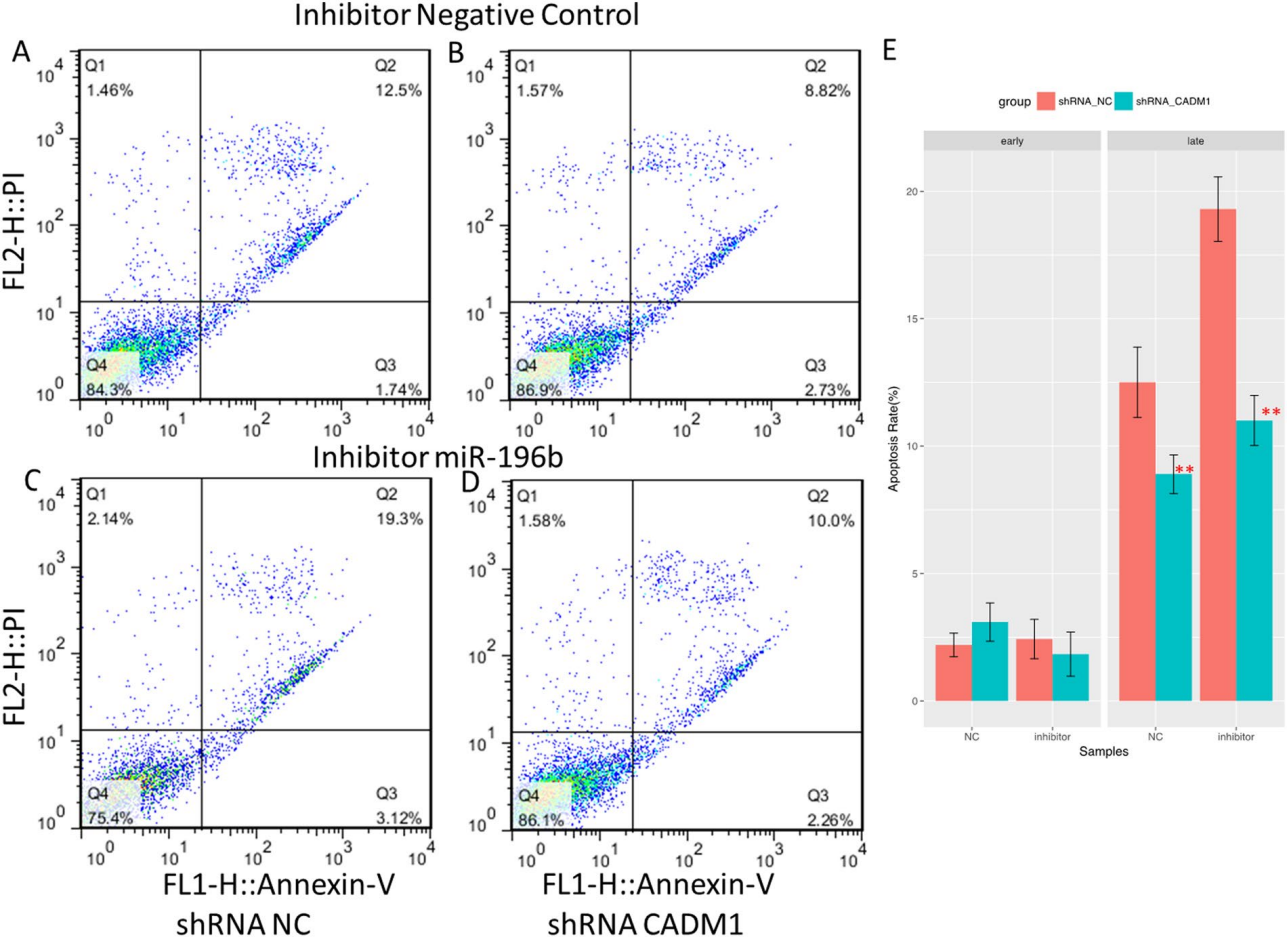
The apoptosis assay of rescue experiment in PANC-1 cell line. Annexin-V FITC/PI assay was used to calculate apoptotic cells for shRNA_NC (**A**) and shRNA CADM1 (**B**) in inhibitor_Negative Contrl, shRNA_NC (**C**) and shRNA CADM1 (**D**) in inhibitor miR-196b. The lower right quadrant represents the early apoptosis, while the upper right quadrant represents the late apoptosis. The rates of early and late apoptosis were shown on barplot (**D**), Significance of the comparison was determined by t test, **P Value < 0.01; *P Value < 0.05.

To find out whether miR-196b regulated PANC-1 cell apoptosis through CADM1, the loss-of-function experiment for CADM1 by shRNA was performed. As shown in Fig. 10, transfection of shRNA targeting CADM1 into cells suppressed the expression of CADM1, even in cell with miR-196b inhibitor. It was also demonstrated that miR-196b inhibitor decreased the expression of miR-196b in PANC-1 cells, but blockade of CADM1 did not change the level of miR-196b (Fig. 10). Intriguingly, the late apoptosis rate was decreased after transfection of shRNA targeting CADM1, and blockade of CADM1 could decrease the late apoptosis up-regulated by inhibition of miR-196b (Fig. 11A–E). These findings indicated that miR-196b regulated PANC-1 cell apoptosis through down-regulation of CADM1.

## Discussion

PC has been recognized as one of the most malignant cancers worldwide, as the annual mortality of PC is almost same as the morbidity^5, 6^. Therefore, finding new indicators for helping the diagnosis, treatment, and prognosis of PC, is necessary. Besides, miRNAs have been found to be as tumor suppressors or promoters through regulating the expression of target genes^12^. In our study, we demonstrated that miR-196b expression is significantly up-regulated in PC tissues, positively correlated with differentiation grades and metastasis, and miR-196b regulates apoptosis and proliferation of PANT-1 and ASPC-1 cells via targeting CADM1.

The miR-196 family, including miR-196a and miR-196b, has been reported to be up-regulated in many cancers, such as breast cancer, leukemia, and colonic cancer^16–18^. Moreover, the level of miR-196b has been demonstrated to be increased in serum of PC patients, but the exact functions of miR-196b in PC was still unknown. In our study, we demonstrated that miR-196b expression was up-regulated in PC tissues compared with adjacent tissues, which was consistent with other cancer tissues and serum of PC patients in previous studies.

Accumulating studies have shown that miRNAs could act as biomarkers for different diseases, especially cancers, and be correlated with clinical characteristics of those patients^10, 12^. In this end, we next questioned whether miR-196b was correlated with characteristics of PC patients. We found miR-196b was positively associated with differentiation grades and metastasis in PC patients. Previous studies have shown that miR-196b could regulate differentiation and migration of gastric cancer cells, which may account for the results in this study^19^. The functions of miR-196b in cancers were also studied, and miR-196b was found to be regulating proliferation, apoptosis, differentiation and migration in different cancer cells^19–21^. In current study, miR-196b was found to promote proliferation, but inhibit apoptosis of PANC-1 and ASPC-1 cells, which was consistent with the results in other types of cancer cell in previous studies.

Our finding demonstrated that CADM1 was a target gene of miR-196b in PANC-1 cells. CADM1, which is also called as TSCL1, has been reported to be involved in cell adhesion, migration, and apoptosis^22–24^. Besides, previous study has found that CADM1 expression was down-regulated in different types of cancers, including gastric cancer, breast cancer, and lung carcinoma^25–27^. In this study, we found that miR-196b could reduce the expression of CADM1 in PANC-1 cells. Furthermore, the late apoptosis rate was decreased after transfection of shRNA targeting CADM1, and blockade of CADM1 could decrease the late apoptosis up-regulated by inhibition of miR-196b. Finally, luciferase assay confirmed that CADM1 was the target gene of miR-196b in PANC-1 cells.

In summary, our data showed that miR-196b expression is up-regulated in PC tissues, and miR-196b regulate apoptosis and proliferation of PANT-1 cells via targeting CADM1. Besides, the level of miR-196b was positively correlated with differentiation grades and metastasis in PC patients. Therefore, miR-196b might be an important biomarker in PC patients.

## Methods

### Clinical Specimens

Paired cancer tissues and adjacent tissues were obtained from patients recruited at Department of Gastroenterology, Zhongnan Hospital of Wuhan University, from August 2014 to July 2016, and all the patients provided written informed consent before the study protocol, with Ethical Approval provided by the Ethics Review Board of Zhongnan Hospital of Wuhan University. This study was carried out in accordance with declaration of Helsinki.

Two patient tissue cohorts were used in this study. One cohort consisted of 20 paired cancer tissues and adjacent tissues from patients with PC, which were used for miRNA microarray profiling. The other cohort with 60 paired cancer tissues and adjacent tissues from PC patients was added to the former cohort to further validate the differentially expressed miRNAs in RT-PCR. The demographic characteristics of the two groups of PC patients were described in Table 1 (20 patients) and Table 3 (80 patients).

### miRNA nanostring analysis

RNA was extracted from tissue samples using TRIzol (Invitrogen, USA), and 100ng total RNA of each samples was used for analysis the expression of miRNAs using NanoString nCounter Human miRNA assay (v1) (NanoString, USA), which contains 654 probes for human miRNAs, according to manufacturer’s instructions. Global miRNA array analysis was performed by importing all data into nSolver Analysis Software v1.0 (Nanostring Technologies) and normalized to the geometric mean of the 100 miRNAs with the highest expression values. All analysis was performed using R/Bioconductor, and filtering of low-intensity signals were then conducted by requiring the mean signal intensity for tissue samples to be larger than 500.

3d PCA (Principal Components Analysis) and volcano plot were applied to describe the pattern of miRNA expression between pancreatic cancer tissues and adjacent tissues. R package limma was used to compare miRNA expression between pancreatic cancer tissues and adjacent tissues, and the differentially expressed miRNAs were required to meet criteria (absolute (log (fold change)) >1 and adjust p value < 0.05). Human microRNA Disease Database 2 (HMDD2) and Gene Ontology (GO) annotation were used to identify the relationship of the differentially expressed miRNAs with diseases and functions. The enrichment analysis of differentially expressed miRNAs was competed by R, based on the hypergeometric distribution.

### Quantitative RT-PCR

For analyzing the expressions of candidate miRNAs (miR-200c (Sense: CTCAACTGGTGTCGTGGAGTCGGCAATTCAGTTGAGCCAAACAC Anti-sense: ACACTCCAGCTGGGCGTCTTACCCAGCAGTGTTTGG), miR-196b (Sense: CTCAACTGGTGTCGTGGAGTCGGCAATTCAGTTGAGCCCAACAA Anti-sense: ACACTCCAGCTGGGTAGGTAGTTTCCTGTTGTTGGG), miR-1 (Sense: CTCAACTGGTGTCGTGGAGTCGGCAATTCAGTTGAGATACATAC Anti-sense: ACACTCCAGCTGGGTGGAATGTAAAGAAGTATGTAT), miR-200a (Sense: CTCAACTGGTGTCGTGGAGTCGGCAATTCAGTTGAGACATCGT Anti-sense: ACACTCCAGCTGGGTAACACTGTCTGGTAACGATGT), let-7b (Sense: CTCAACTGGTGTCGTGGAGTCGGCAATTCAGTTGAGAACCACA Anti-sense: ACACTCCAGCTGGGTGAGGTAGTAGGTTGTGTGGTT), RNA was reverse transcribed into cDNA using the All-in-One™ miRNA First-Strand cDNA Synthesis Kit (GeneCopoeia), and the miRNA expression was then determined by real-time PCR using the All-in-One™ miRNA qPCR Kit (GeneCopoeia). As for measurement of the expressions of mRNAs (CADM1 (Sense: TGCTGTGCTTGCTCATCATTCT Anti-sense: TCTGCGTCTGCTGCGTCAT), HOXA13 (Sense: CGCTTCAGAACTCGTTGCTTTG Anti-sense: CGGAAGAACTGGCAGTCTTTACCT), IER3 (Sense: CCAGATCCTGATGGCTGAAGAG Anti-sense: TGAGGTCCAGAGCGTAGTCC)

MEF2C (Sense: TCTGTCTGGCTTCAACACTG Anti-sense: TGGTGGTACGGTCTCTAGGA), PTGS2 (Sense: TGACCAGAGCAGGCAGATGA Anti-sense: CCAGTAGGCAGGAGAACATATAACA)), RNA was subjected to reverse transcription with 5 × All-In-One RT MasterMix kit (Applied Biological), and quantitative real-time PCR was conducted using a SYBR Green PCR kit (TaKaRa). miRNA and mRNA expression were evaluated by using the 2^-ΔΔCT^ method with normalization to internal control, U6 (Sense: CTCGCTTCGGCAGCACA Anti-sense: AACGCTTCACGAATTTGCGT) and β-actin (Sense: ATCATGTTTGAGACCTTCAACA Anti-sense: CATCTCTTGGTCGAAGTCCA), respectively.

### Cell culture and transfection

PANC-1 and ASPC-1 cells were cultured in Dulbecco’s modified Eagle medium containing 10% FBS, 1% penicillin/streptomycin, and 1% glutamine. Here, 5 nmol/L of miR-196b mimic, 50 nmol/l of miR-196b inhibitor and each negative control (Ambion, Austin, TX) were transfected into PANC-1 by using Lipofectamine RNAiMAX (Life Technologies) according to manufacturer’s instructions. The shRNA targeting CADM1 was from position + 1225 to + 1274, relative to the transcription start site, while its negative control did not match any known mammalian genomic sequence.

### Cell apoptosis assay

After culture in serum-free medium for 24 hours to induce apoptosis, cells were harvested and stained with FITC annexin V and propidium iodide (PI). Cells fixed by 4% paraformaldehyde for 15 mins were used as positive control for cell apoptosis assay. Subsequently, apoptosis rate was analyzed by cytometry.

### EdU cell proliferation assay

Cell-light 5-ethynyl-20-deoxyuridine (EdU) Apollo Imaging Kit (RiboBio, China) was used for measuring the cell proliferation according to manufacturer’s instructions. The percentage of EdU cells was assessed using a fluorescence microscope.

### Cell cycle assay

Cells were harvested, fixed in 70% ethanol overnight, and incubated with RNaseA, followed by staining with PI in the dark for 30 minutes. Flow cytometry was used to analyze the results.

### miRNA target prediction

The candidate target genes of miR-196b were predicted using TargetScan (http://www.targetscan.org/) and the miRNA-3′UTR binding sites were calculated by RNAhybrid (https://bibiserv.cebitec.uni-bielefeld.de/download/tools/rnahybrid.html).

### Western-blot

Cell lysates (20 g) were subjected to sodium dodecyl sulfate polyacrylamide gel electrophoresis, and transferred to polyvinylidene fluoride membrane. Subsequently, the membrane was blocked, and incubated with primary and secondary antibodies. Signals were detected using the Amersham ECL Western Blotting detection system. The beta-actin and CADM1 antibodies were purchased from Abcam.

### Luciferase reporter assay

3′UTR luciferase reporter assay was conducted to validate the target gene of miR-196b using the wide type and mutant 3′UTR of CADM1. The sequences of Renilla luciferase and firefly luciferase were constructed for reporter fluorescence (Rluc) and calibration fluorescence (Luc), respectively. Cells, miRNA mimic, and vectors were mixed, and cultured for 24 hours, and the luciferase activity was examined by a dual-luciferase reporter assay system.

### Statistical analysis

The continuous data following normal distribution (or not) were expressed as mean ± SEM (or medium (IQR)) in this study, while categorical variables were descripted by count (percentage). Student t test and Wilcoxon sum test were used to compare two mean or median of two independent groups, while paired t test and Wilcoxon signed-rank test for paired groups. Besides, the spearman coefficients were used to identify the correlation between miRNAs and characteristics of PC patients. All statistical analysis was conducted using R, and statistical difference was set at *p* value < 0.05 (*), *p* value < 0.01(**).

## Electronic supplementary material

Figure S1- Figure S4

Table S1

Table S2
