# Supplementary material for: MicroRNA-196b inhibits late apoptosis of pancreatic cancer cells by targeting CADM1
Source: Sci Rep. 2017 Sep 13;7:11467. doi: 10.1038/s41598-017-11248-3 (PMC5597590; doi:10.1038/s41598-017-11248-3)
Supplement: Supplementary file 1 — Figure S1- Figure S4 [file 41598_2017_11248_MOESM1_ESM.pdf]

MicroRNA-196b inhibits late apoptosis of pancreatic cancer cells by targeting CADM1

Hong-Ling Wang[1,2], Rui Zhou[1,2], Jing Liu[1,2], Ying Chang[1,2], Shi Liu[1,2], Xiao-Bing Wang[1,2], Mei-Fang Huang[1,2] & Qiu Zhao[1,2*]

[1]Department of Gastroenterology, Zhongnan Hospital of Wuhan University, Wuhan 430071, P.R. China

[2]The Hubei Clinical Center and Key Laboratory of Intestinal and Colorectal Diseases, Wuhan 430071, P.R. China

*Corresponding Author: Qiu Zhao, Department of Gastroenterology, Zhongnan Hospital of Wuhan University. 169 East Lake Road, Wuhan 430071, China. Tel: +86-27-67812888; Fax: +86-27-67812892; E-mail: qiuzhaoznyy@163.com

Figure S1

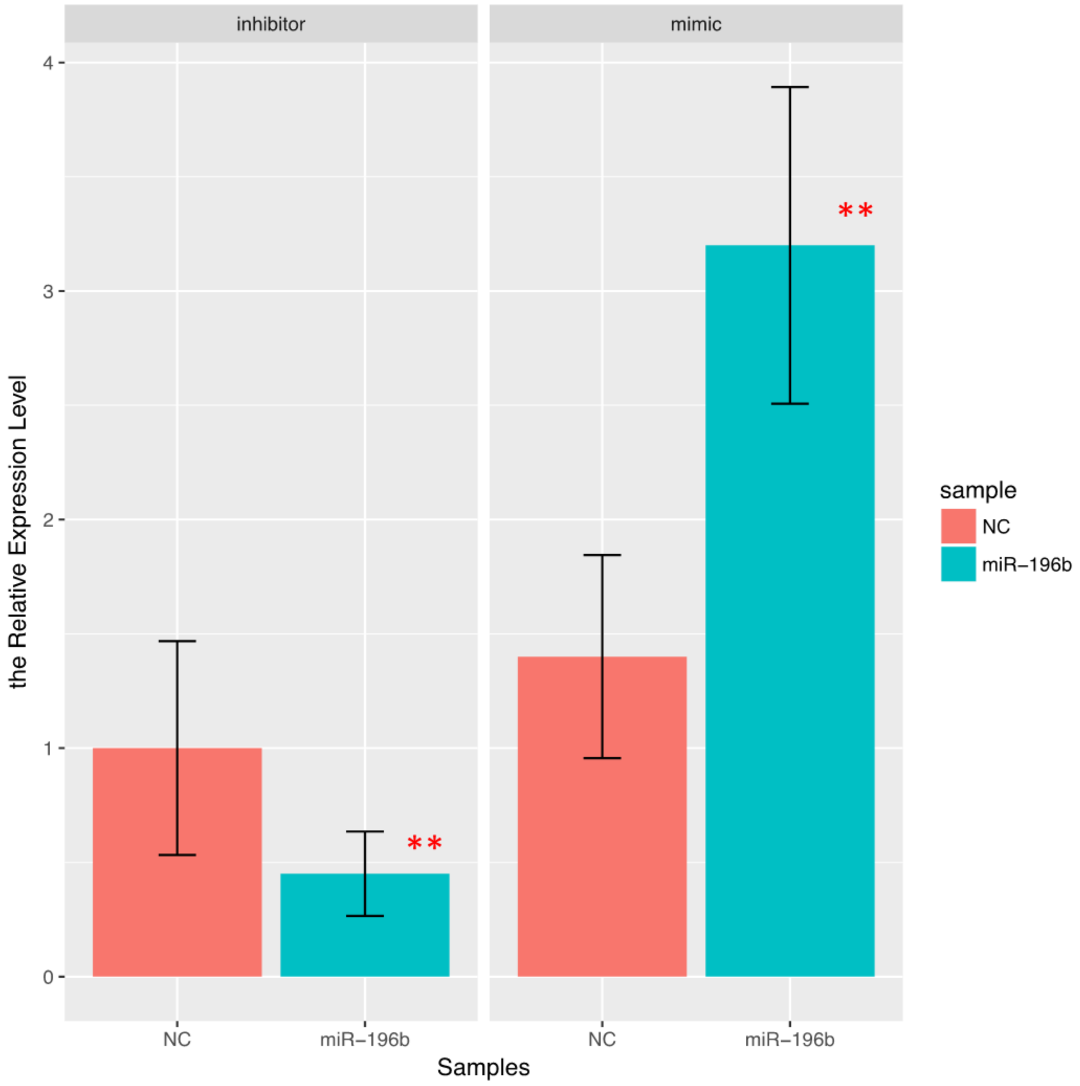

Figure S1. The barplot of expression level miR-196b in ASPC-1 cell line within or without interrupt miR-196b by inhibitor or mimic. The expression level of miR-196b in two interrupting systems, inhibitor and mimic, represent as blue box, while their negative controls represent as red box. Significance of the comparison was determined by t test, **: *P* Value <0.01; *: *P* Value <0.05.

Figure S2

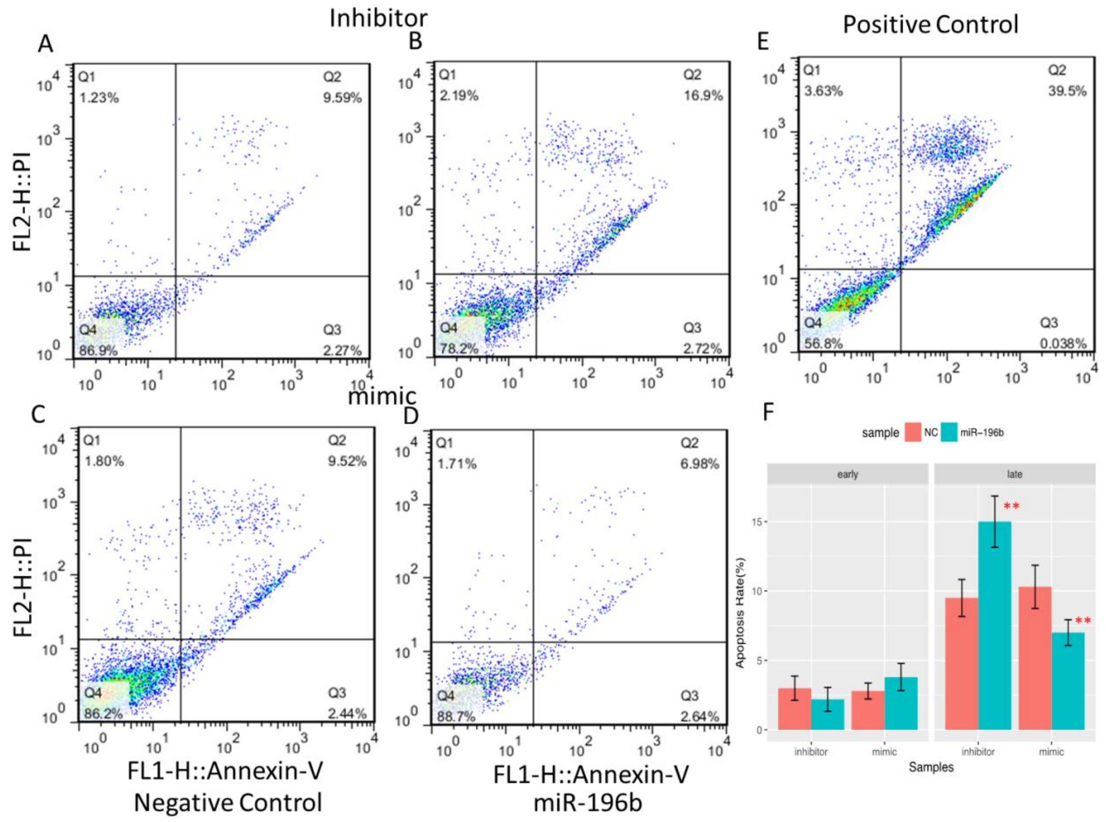

Figure S2. The interruption of miR-196b affected cell apoptosis by flow cytometry in ASPC-1 cell line. Annexin-V FITC/PI assay was used to evaluate cell apoptosis for negative control (A) for inhibitor system and miR-196b inhibitor (B), negative control (C) for mimic system and miR-196b mimic (D), positive control (E). The lower right quadrant represents the early apoptosis, while the upper right quadrant represents the late apoptosis. The rates of early and late apoptosis were shown on barplot (F). The means of cell early and late apoptosis in two interrupting systems were compared with its negative control by t test, **: *P* Value <0.01; *: *P* Value <0.05.

Figure S3

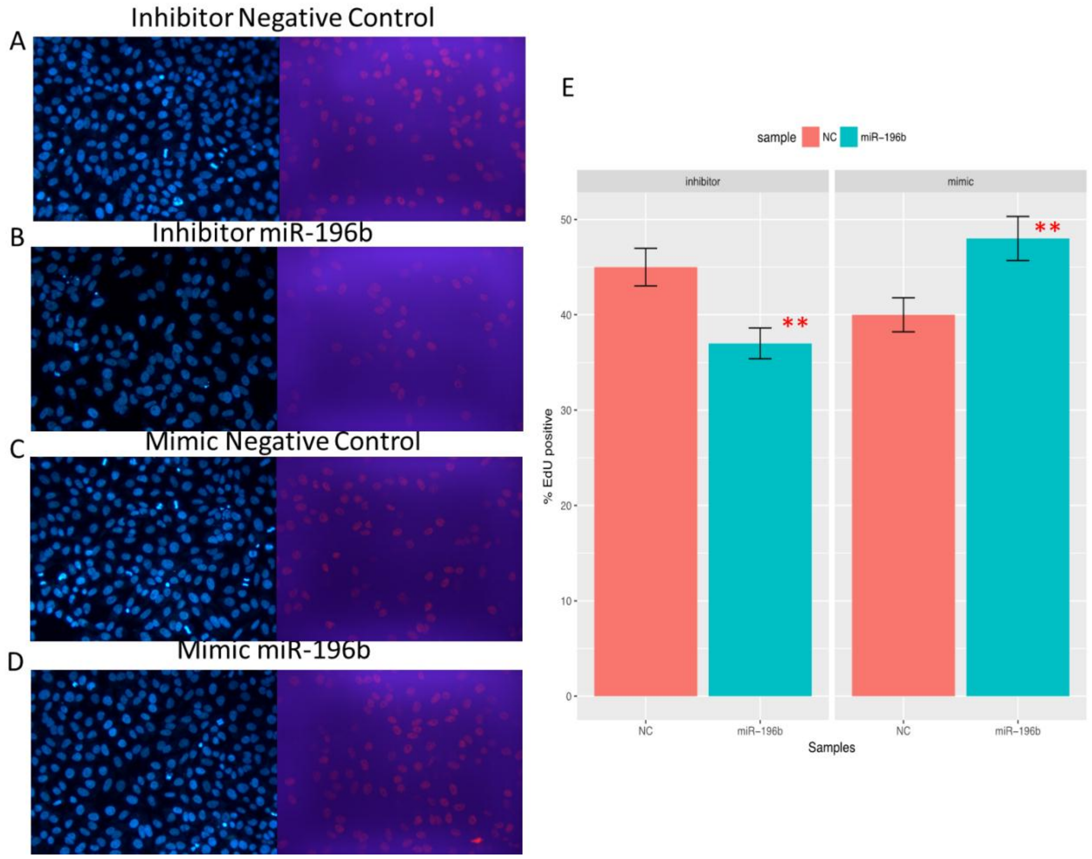

Figure S3. The interruption of miRNA-196b affected cell proliferation by Edu analysis in ASPC-1 cell line, negative control (A) for inhibitor system and miR-196b inhibitor (B), negative control (C) for mimic system and miR-196b mimic (D). Proliferating cells were labeled after conjugated reaction of Apollo dye and EdU (red), while the total cells represent as blue (cell nuclei marked by Hoechst 33342). The rates of proliferating cells were shown on barplot (E) and compared with its negative control by t test, **: *P* Value <0.01; *: *P* Value <0.05.

Figure S4

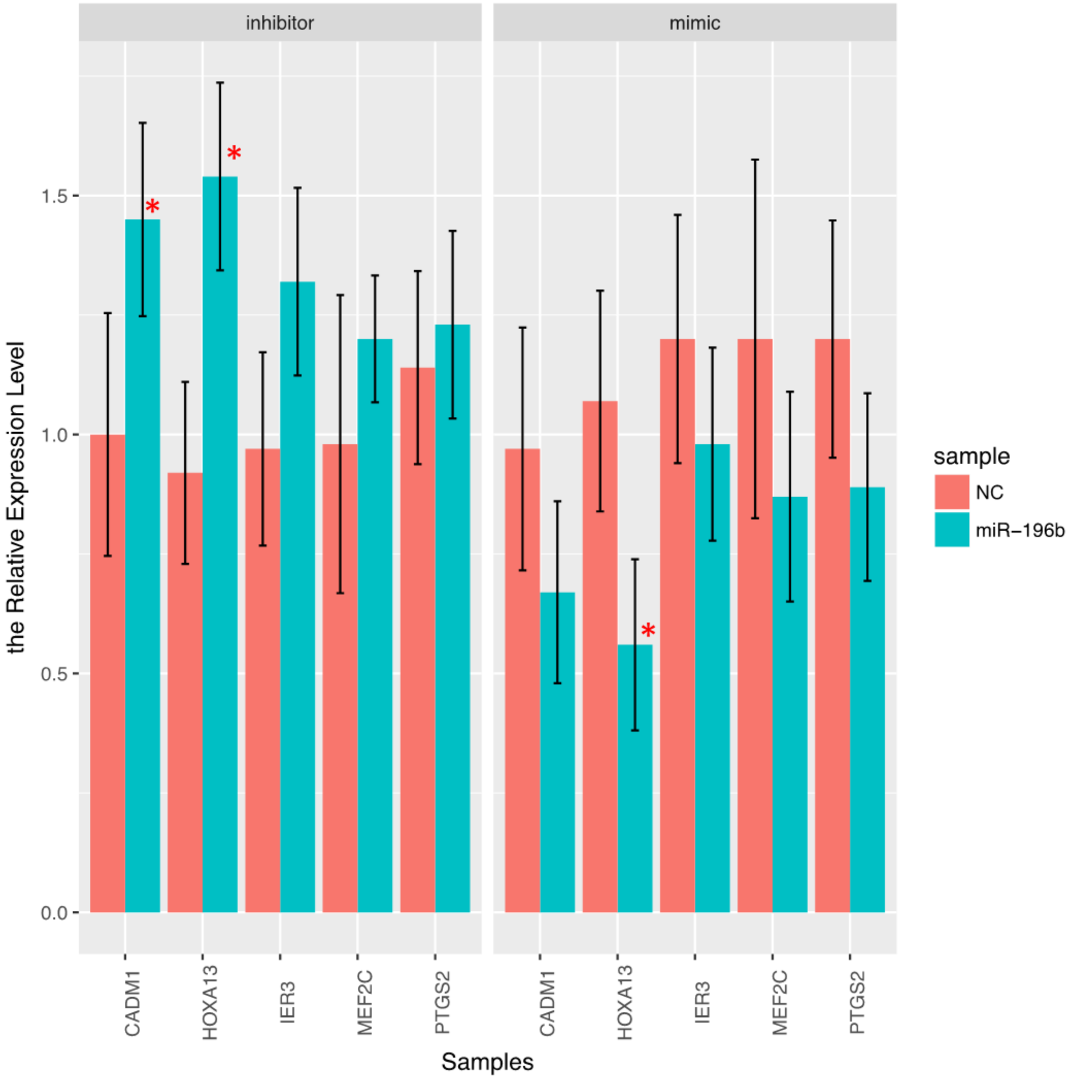

Figure S4. The barplot of mRNA expression level of 5 candidate targets of miR-196b in ASPC-1 cell line, inhibitor miR-196b and its negative control, mimic miR-196b and its negative control. Significance of the comparison was determined by t test, **: *P* Value <0.01; *: *P* Value <0.05.
