# Supplementary material for: MicroRNA-196b inhibits late apoptosis of pancreatic cancer cells by targeting CADM1
Source: Sci Rep. 2017 Sep 13;7:11467. doi: 10.1038/s41598-017-11248-3 (PMC5597590; doi:10.1038/s41598-017-11248-3)
Supplement: Supplementary file 2 — Table S1 [file 41598_2017_11248_MOESM2_ESM.pdf]

MicroRNA-196b inhibits late apoptosis of pancreatic cancer cells by targeting CADM1

Hong-Ling Wang[1,2], Rui Zhou[1,2], Jing Liu[1,2], Ying Chang[1,2], Shi Liu[1,2], Xiao-Bing Wang[1,2], Mei-Fang Huang[1,2] & Qiu Zhao[1,2*]

[1]Department of Gastroenterology, Zhongnan Hospital of Wuhan University, Wuhan 430071, P.R. China

[2]The Hubei Clinical Center and Key Laboratory of Intestinal and Colorectal Diseases, Wuhan 430071, P.R. China

*Corresponding Author: Qiu Zhao, Department of Gastroenterology, Zhongnan Hospital of Wuhan University. 169 East Lake Road, Wuhan 430071, China. Tel: +86-27-67812888; Fax: +86-27-67812892; E-mail: qiuzhaoznyy@163.com

ID_REF T_1 T_2 T_3 T_4 T_5 T_6 T_7 T_8 T_9 T_10 T_11 T_12 T_13 T_14 T_15 T_16 T_17 T_18 T_19 T_20
N_1 N_2 N_3 N_4 N_5 N_6 N_7 N_8 N_9 N_10 N_11 N_12 N_13 N_14 N_15 N_16 N_17 N_18 N_19 N_20
logFC AveExpr t P.Value adj.P.Val B sig1
hsa-mir-200c 12.6623758049673 11.950938970557 13.7956352432248 13.933905484207 14.2779008194893 13.4757696961821 10.5279909062123
9.59807142191202 13.9631999102024 12.956208024603 9.50200171780968 11.3028558437563 10.2011294270433 10.8717900122232 12.7029713740332
12.4554781887742 14.7969889633086 9.95882955551113 13.4189074484709 11.287076839627 11.0478871899568 5.90017059112536 10.3774349135539
7.20423300318096 7.80273688264717 10.9813929170445 8.24648527047341 10.9334975553933 8.60410862233725 10.0939404950637 11.0373661438727
6.67999708113933 7.94600524635973 9.34808510502486 6.25870050276054 9.44417529960632 8.16249153663442 9.17823804917481 10.3098208764078
11.5074425904339 3.1287907889962 10.6176058881076 6.99993546265741 8.06407290534347e-11 4.86263596192212e-08 14.2879459645271 UP
hsa-mir-196b 7.97446101755046 8.16872534594945 8.38516515831015 8.21797305345102 7.14270031197944 7.87679796076692 8.62148717337304
7.2325681045045 7.38019953288119 10.8522359855229 6.92105950324976 5.14421026492395 8.61465531138493 7.73750142109517 NA
7.50123018330691 5.9486512117496 7.25812165642417 9.16187332490999 8.64119640941857 5.456285727859 5.48652607022773 6.84992791024115
4.02495936403758 4.53744150267842 5.03617161700105 3.81125013777798 5.17348899717274 5.47094538575182 6.15241135067426 5.06991175333698
6.65324401755212 5.07146349006532 4.70142333835685 2.5934281773101 5.23922769363617 5.82392238722913 4.90748718114215 4.68273812805287
5.00599432381094 2.74315667392282 6.42382208935042 6.8490441623118 1.83952409972767e-10 5.54616516067892e-08 13.4963235354997 UP
hsa-mir-1 1.87532717352032 1.99029760962534 3.51855044431627 1.42990511721102 NA 6.38882439958355 2.44510115963078 0.443848305651538
1.26797806734953 4.57528054363105 5.78127661599806 2.42965174783713 NA 1.85726383503049 NA 0.396810031313066 2.38373288510141
2.65745371748111 NA 3.90040658776199 3.56986156995479 9.09373877521148 10.1274686864708 7.82148354411027 6.63192683518126
6.30947236816768 3.49743626857736 1.7682213260576 8.9753313478953 4.63326106551977 4.28772194148439 6.80488509303835 4.76443134015061
3.69359015491474 6.81245161536321 NA 7.57027946877561 3.72837160269778 4.51568739568806 NA -3.10256659044923 4.3513920188324
-6.00256777935681 1.51398858050803e-08 3.04311704682115e-06 9.28440322812589 DOWN
hsa-mir-200a 9.59131102140315 10.2016292917882 9.3342514591977 11.2072108015392 10.31367615241 11.4862045154868 8.13250019713152
8.72661383014363 11.5423930136932 11.3015762880153 9.39135983164007 9.85518166557613 10.2769739191041 11.0334293760979 9.99967391421197
12.9329147201881 9.23331905649779 11.1465850077667 12.0549129781851 10.6607876078409 8.31536294425553 3.11701413407305 7.70715852574326
8.7266163653567 7.89309739818045 7.89917921009127 7.53050290142492 8.80873529790295 7.64416409118514 8.37449469844066 7.83509997612401
6.86950619116399 11.9687542438925 9.92170648697596 3.60886886252045 8.79495920478292 8.47659483772899 8.15423757121818 8.16950763675113

8.94758966543858 2.48296772023334 9.1796413722792 5.71143070788051 5.85857356684217e-08 8.83179965201457e-06 7.9634013761443 UP
hsa-let-7b17.3532948332187 13.889218779197 12.7397815590678 12.7579413180859 13.7859734071486 15.9293063724949 16.0891864662774
13.7199778208395 12.3698045936041 12.7788357790027 14.1308211981073 12.4364147631042 13.2052505381197 11.916654276438 14.0406000838041
17.8914597463715 11.5591058909202 13.3691069303415 16.0443876896538 16.2286502487974 12.3504270768884 13.3196661565162 12.6371866193852
10.9913730540145 10.9095421794059 12.0248812052809 10.7258052576018 12.4392301270359 11.7110741672142 12.3724892285952 12.7053860508884
13.0635515314979 10.380545849905 11.3368104360217 11.282691390804 11.7606936353085 10.8771748306119 11.0224455852495 11.8495792106355
12.2229786832005 2.31261200092666 12.9554826142664 5.48099055579967 1.756361059819e-07 2.11817143814171e-05 6.91463802240499 UP
hsa-mir-31 6.90393074590568 11.001815461104 11.4102586962262 10.441723986665 NA 9.8137255755498 6.05888711869857 7.83013791898135
10.7725440480301 7.02925315557024 5.2370286856562 10.0065156548516 7.87365215673197 9.92043562144472 NA 6.00433210541302
10.2532532766653 10.273935941321 6.98921624342897 9.81590177826192 7.39616903873597 3.31772883498585 6.98956431498068 5.36066496461792
5.30573928116729 8.49915046820767 5.9650093272639 8.6709568317636 4.46572258086253 7.28415461656826 5.45997107211649 5.23689617707227
8.94537084484376 4.2110490665558 5.33348380431854 9.06346251978567 4.84110408790103 6.86546771868031 2.62857290404037 7.4286772278265
2.59414022535781 7.3922490487579 5.40938302280552 2.49721070566523e-07 2.50969675919356e-05 6.59170615033366 UP
hsa-mir-126 16.1893669671898 14.8173649290555 13.2127951756514 12.2661054472181 12.3159134538125 13.4140445777686 17.5132540999131
13.6715479253888 12.534746982457 16.2845921480236 13.501011931799 15.705893081565 14.7873062071784 15.0302256258055 11.1738787533776
14.9411726894746 11.9224768496105 13.8540982611557 13.6132394917036 12.6074439752684 11.091718300331 14.3611258988387 10.9108915638442
10.7460168840308 12.3849427611589 9.06156759873195 11.087239115258 11.3762545564996 11.0026315710205 11.1682777352286 11.424772271625
13.1906868962396 11.803725577606 11.348827733805 12.9196179962417 13.1859214777203 13.9612952149829 12.1412289395248 11.1765908627326
10.0323370120604 2.24904043029681 12.8433037135224 5.26629172987655 4.76473882801558e-07 4.1044821618477e-05 5.96323330974582 UP
hsa-mir-429 9.62282863722592 7.67652685961872 10.0343238203652 9.2728871194535 6.1071276193046 6.77240739607677 8.16732796641708
7.87589455610507 7.0939941924325 7.07469758120981 7.88062873457776 8.27237124840973 6.37092596949373 7.22531459927301 6.96973939829327
8.61270692851522 7.28502851778507 8.82482300317047 8.4527000148554 9.64679534965478 8.87828240577783 3.63340933869173 6.29924053090208
7.11611292300016 6.64265401699912 6.13602576080195 7.53719459042143 7.61393367708504 3.76887295549943 5.79030942178118 3.96980690238728
NA 3.56057988886339 5.41239813832858 2.95451985804943 6.35297619372967 5.40483041788797 3.93715226383588 8.82818425615074
5.56614984039035 2.20391913979171 6.8882482793031 5.10553671483879 9.96164282406319e-07 6.81152995022916e-05 5.26851182126068 UP
hsa-mir-144 1.76297352421977 3.11859687536858 1.80579337646582 4.78479314719732 6.12715078505558 2.45310823208942 7.10839915432514

3.97767221579113 6.35989648539662 6.1196066135647 4.68903903485267 3.16658259592322 7.86919431196501 5.81250415072338 7.22188755566389
4.63833940208686 5.86598851570691 5.00399906529186 4.99138539266613 8.71523429675975 7.58955333492179 9.86186047562669 8.4450650504346
6.02526002686792 6.4314407056986 9.05595629384099 6.57652924069913 4.64158978845879 5.98777470306431 8.53760687143931 8.6308351031018
12.4935641862928 5.89581908735373 6.27253328497464 6.3791734459932 9.49522275856555 7.13971041097283 6.05421946080792 6.89239000025926
6.67687188532696 -2.37454156917935 6.26687802114536 -5.09953469152526 1.01664626122823e-06 6.81152995022916e-05 5.24216744054812
DOWN

hsa-mir-29a 9.68780900261288 10.4968263172891 11.9712068454918 9.34002157217395 9.33316681088945 12.0748778457333 12.2067602251942
9.73787889738032 9.07239833250786 10.5289266816808 8.91821776536317 10.2739738524371 10.6136230484905 11.3033383714897 9.55398779213543
9.79366220348856 9.22664366498616 10.7779178381455 10.8866991331017 9.27869606874334 13.2674442011611 12.9580900536654 12.7971004752787
9.42349270745236 14.3606423790924 12.3459178989147 12.0871755908725 12.0044365076379 12.9622916127637 11.269493710061 14.4389742698635
13.0517130043228 10.3446601147822 11.7188533261842 11.5434767219134 13.3916887300926 10.5029156613734 12.7981879186606 12.284367162347
11.490066564629 -1.99821781708668 11.2529405220101 -4.99232670321278 1.6412956199173e-06 9.77653034111745e-05 4.78714271256877
DOWN

hsa-mir-24 11.0054742379532 13.877161167272 14.6510952674589 13.4678402939685 9.46137058382418 14.6112952618365 12.9144783334564
14.197990935744 12.0358025041231 11.8234670030935 11.8220613687696 12.0323082979349 13.8094527358545 12.7743083788558 11.7919976185591
13.5180386313879 12.8911795766215 13.5405777782721 10.9766784396647 13.4035295174301 10.5949174579927 9.77269900566161 10.9170158633614
9.12580261577224 11.2010858006779 10.2422169234389 10.3101421049034 11.2723424318409 12.8791113606555 10.2797452904386 7.53811778693381
9.95934717835971 9.74738794903772 11.908778829556 12.9241103891441 13.6015240940404 9.35614576331933 10.4149919475201 12.4566807683279
7.63978731455552 2.12320785282714 11.6687014701905 4.97358408418417 1.78344666255874e-06 9.77653034111745e-05 4.708293924678 UP

hsa-mir-376c 5.6858541795736 3.86964789452031 2.57710223140943 5.00278014485622 5.99606453915808 6.620681279753 6.1255186699004
2.79537677079059 6.87529680100238 2.88209716948844 6.49302108523863 5.79759591371713 4.40103698672958 5.04645256171181 4.32101359089718
4.68234984829261 3.91353725253459 7.0958362437063 4.88186794344804 3.68145781239561 7.31615127040102 5.45315796149883 8.75338985346303
8.14404924820535 6.88577138369123 4.68407500972659 6.28654524137086 8.70915576642401 7.00135224080241 6.81485915670722 6.26265001778922
7.5508927793993 6.76500585899571 7.93759689457619 8.45498136640257 7.44592908959015 7.31870907269448 5.23435474422661 5.92081059898659
6.12729618696863 -2.0161072411398 5.9452830665261 -4.9338951589325 2.12499093030859e-06 0.000106780794248007 4.54202177577756 DOWN

hsa-mir-103 7.89096600180273 7.39994143250922 9.1347579266056 6.75915115335896 5.33805555886 7.29653785117244 8.17482272689552

6.4906031898128 9.27056852627903 11.1937125864697 7.69104729884588 10.0584251952361 6.98058550208981 6.46379969749699 9.86342649764425
8.36795580667321 8.3690213973379 10.9111123669867 7.36977069888423 6.14785744402381 10.3580137206724 9.15269454814116 10.7588846281618
9.75395909747859 9.96982133622468 10.4723215113052 11.3223645784161 9.99945886362082 11.2616129304006 10.0018728728612 10.4058579165757
8.50377364697397 11.8512600395938 9.5338713023639 10.1086947496906 9.99091961569012 7.56802273001719 9.965350235165 10.0208434025354
9.87330433400348 -1.98503916004534 9.05112552297192 -4.81659163523316 3.5475700393726e-06 0.000164552671826283 4.05618518706661
DOWN

hsa-mir-135b 10.640986561725 8.84352734151979 10.1024120777676 8.65727616675021 7.79388447002543 11.2742834594924 6.77775588769628
9.72646279416529 10.2604220545702 10.8352576910224 8.67578050376459 10.8106225189456 5.20486150298143 12.0781147479519 5.68701752796479
11.2549097410102 10.5467100971216 9.25990971970488 8.55649747863603 7.19796033512562 6.50928647442337 3.93606851584936 6.84745975009071
6.38626211911284 8.2327884677399 7.71722088570482 6.18683091521781 8.97954589676541 6.34206557719261 7.31594266388911 6.08156867453431
4.35942003606442 10.5116776734282 5.87778830579244 4.51930018827684 7.23559394366802 6.77097057140537 11.3453854050454 6.95911452742221
NA 2.25584891854848 8.11022931460422 4.78596260998943 4.07169393606214e-06 0.000175373674531819 3.93339916362368 UP

hsa-mir-107 5.92288402140784 6.79029196297856 9.83757280274347 4.4034359575114 4.57957771018872 7.49901151782595 8.31765221330889
8.73898141203576 6.38157818218608 6.80601645457097 3.67455081558034 5.78463132410242 5.96344332928572 8.94473022585678 7.82856647811481
9.72436564139093 9.97606759555772 7.63974389570051 5.93233689173011 4.95887622522415 5.34683847035815 4.43679668699262 5.93487808399181
5.53927895158963 2.59859932129658 4.3978284720967 5.44221872574696 5.52666914898066 6.13993071434008 5.52145033265924 5.79335013166464
5.24738110419991 5.83152623862116 3.26603580680778 5.14160367693888 5.15959207400179 6.29967485154104 2.37944481127166 3.91347311967832
NA 2.04223832640308 5.99027911230971 4.64618084414332 7.39623113475525e-06 0.000283841775127726 3.36908269804589 UP

hsa-let-7d9.36403021165325 8.76083627519051 10.5059689093089 12.3457358976622 11.4683387930883 12.7980863082337 11.9884291427601
14.3779545041488 9.29201981760108 10.9079172132804 11.6958603990653 11.6035149825291 8.64521570972637 11.095654464225 8.37715380809012
13.7888857178032 12.2503033622338 12.2880598651626 9.6993572969749 11.5853382654723 8.72145632631669 7.52945211835248 9.71761630636087
10.3594585999044 6.71980440169236 8.33753956849527 9.10320616630633 8.63041346861103 10.177530720049 8.46898675274544 9.84104304149175
9.38970792266153 9.64741028140057 10.6234800877418 10.5304294833543 8.83395888935876 8.62111838503182 9.49339732685037 8.68546417121249
10.4789966129698 1.94640951566514 10.1687282893779 4.64076401174922 7.531456719807e-06 0.000283841775127726 3.34395422813424 UP

hsa-mir-95 6.37421773948078 8.76319052622518 7.25821332205026 6.7862145943616 3.99993611294952 7.6081152622238 5.02413376615113
10.0256025389214 6.63200836154842 NA 7.80933733309415 6.19991383409345 4.85620313772742 7.31869164601925 NA 6.24316581455833

5.65614803620253 8.99360196698234 7.3964277461715 6.96337119735964 4.23818880755807 3.96417703540637 4.63342511280321 3.78173408677102 4.61407062365326 NA 4.86740764927514 5.05491952328463 4.74372221737177 4.34733699428405 4.73752546156833 5.26735228090628 4.82864596590049 6.73928603937506 5.81345818052401 4.03216693359899 3.71408122344579 5.91481048221505 6.4375989718454 6.15246827278865 1.94262748614016 5.88623969726206 4.60203535681371 8.99322783786132e-06 0.000318995081542963 3.20052550390156 UP

hsa-mir-497 5.94899423938511 7.51798161592792 4.08780380272495 8.71472244806855 4.32770406229702 6.95229746137363 7.97716085909837 5.53785905090889 5.82100070096349 4.64880776670537 6.07543636463404 4.92783929673754 7.63262286979168 9.77223441399501 5.06665501061242 6.78957198086283 7.4618061376513 6.4164663510809 7.38887961455798 7.27047274963073 9.06970680448421 8.3848133080443 8.86846455857283 10.2518347371573 9.20802002326518 9.45678449245033 9.10991437825296 7.63306558534072 8.51646752966886 8.27227000131655 6.60087150628945 10.1147603742513 7.20963003778811 6.96018130197281 10.935324429584 6.04651547818242 9.40791568290467 5.39241961998272 7.26144431783835 7.64809344222588 -1.80060904062826 7.41712036016452 -4.22297703686421 4.17167852453504e-05 0.0013612665243446 1.73207696393479 DOWN

hsa-mir-23a 13.3374874201205 12.2084710406997 12.1777114467496 10.843379069227 11.134912419603 12.9147029656325 10.0078478491509 12.8333714034538 11.6490851993284 15.193721585438 12.9530425657637 11.7673747593584 13.8721390081212 16.8994999770064 11.9014117149074 12.1355263653511 15.3419216841075 13.7428716635321 11.3391507009996 13.9383634135404 8.30952895686828 12.7140437481316 11.9631091879861 9.26674836875341 13.7758610708524 11.279715567557 9.53338386327991 9.71270474571644 9.90441370283197 11.3277170394891 11.3469799300595 10.518878627647 10.3760768890732 10.6466816679743 11.8036612671372 12.9092540000637 12.4151943834903 10.9360300451726 10.9798251613725 10.198499766467 1.81368421310839 11.9027575060504 4.21583990921103 4.29129721314574e-05 0.0013612665243446 1.70556091073886 UP

hsa-let-7e 8.53937967199809 10.2351740493433 9.67419991468957 11.4718361826773 8.32704070534934 9.69125032629284 9.99152014807691 8.66161012711196 7.87107884733796 10.9159071783679 10.1603932014295 8.14755949034953 11.8977740154778 10.4278062075622 11.0546452531979 10.1074029216622 12.3319278872504 10.8590202960328 11.6312707058336 12.7646902155976 8.8693260456516 8.85292676260955 9.31555941796364 7.875635057079 7.98618207161552 8.10904483723982 8.99924261214135 8.37282969883304 9.22482916644688 7.59580435176204 8.34149633503329 10.5054424526452 6.88158254113354 8.38457807528487 10.2768902616671 9.13006876989388 7.61178790428026 8.8160319684531 8.04287258668588 7.77052302743896 1.68994167008901 9.39310353223743 4.20299082792471 4.51498018024744e-05 0.0013612665243446 1.65791221572139 UP

hsa-mir-7 4.66309668873793 3.48036178976373 NA 0.0915226731821854 NA 4.39617820676054 NA 0.501105589396315 NA 1.71566885197385 4.2338707890296 1.23084924776944 NA 2.65243718371116 NA 2.88876585647631 1.77002813223469 2.36544244682048 NA NA 3.81687576197936 3.81239998905757 3.34954260702896 5.02978854597888 4.88103873369221 NA NA 3.89188318554829 NA NA 5.77720336139506 5.09032977120117

NA 4.92775246025188 NA NA NA 7.74687167127237 NA NA -2.33325798741922 3.55968243378464 -4.1388285023457 6.19597058658832e-05 0.00169825921077852 1.53504012462933 DOWN

hsa-mir-26a 10.285949647339 8.40738317029156 13.1973080159437 7.88511467909088 12.3841841491643 12.0295983887215 12.5349394929444 11.8035139185957 10.6884688381596 11.1176319969631 11.3206319021481 11.7886449740792 12.2923490159612 9.88884465051344 11.3520599479847 11.2101854182382 9.52496152373342 12.006198409427 12.1676780017138 10.4885013298453 9.73474826167942 12.1005145086632 14.1967498590082 12.966899101673 13.8094853577262 15.0523719512491 12.9569483951433 12.2183429031958 12.6499091108233 13.2339684965924 10.7216995471244 14.4400404854933 11.8794752108443 14.9027250206129 13.9223901455061 13.4223283731394 11.4428695312295 11.7318377428317 12.2694431483757 13.249781150994 -1.72641904155236 11.9819168943191 -4.13686041724144 5.85439576171398e-05 0.00168104792586358 1.41448851951428 DOWN

hsa-let-7i 14.7136917586842 12.7175729379212 9.86922480782977 12.9241244613465 11.3012898144013 15.3302778279891 13.8002506534557 13.4473518737137 12.2132184079011 14.6219169877628 13.1297899157401 13.1022711687763 12.7487332294294 12.9372673325211 11.8208965342182 10.1667562462535 10.7608323031688 8.06029403356092 9.34445996609237 11.924756828208 8.3285083848072 11.1670856304837 10.8885132928464 11.4658686785107 13.5343304449117 11.0576528457744 10.7459831320321 9.9304137830828 11.1335148654798 10.2994947626714 10.2470290088772 9.75832298876554 10.7924158292738 7.48626017609632 11.8897048063387 10.389058152193 10.9206214456438 11.7600768411214 8.59817832568539 8.35338611604063 1.80942787891691 11.3420349149903 4.08116493258863 7.26988719882438e-05 0.00190597477430048 1.2118445112292 UP

hsa-mir-214 5.97885164690173 6.25065086065257 8.2298357883266 7.8626962683232 8.02269764204776 9.73428478729413 6.44714175061736 6.2610273005758 6.16257488214956 6.85036981395933 6.07736007871403 8.92506830972034 5.47808784667595 7.6111004529909 9.16876466769524 4.19046846343349 8.69837579455162 6.36953413119107 8.63418828295066 7.29150077366171 3.66572636199059 6.35553517850568 4.68435581692142 7.76191436666362 5.51717406108294 4.12543797287739 4.09743455429668 4.34689804995389 6.91161996849889 7.4041398147401 3.54806851133832 6.5784839095693 5.4219601249789 NA 7.11351410497652 4.6514547267877 6.54280650555384 5.5976732517916 5.19604530742838 NA 1.68332661112411 6.41486374027339 3.9220377998662 0.000134135497806085 0.00332149150583806 0.659443569739071 UP

hsa-mir-224 6.99224873708089 5.41921479927151 8.38369484462389 5.85878502753518 NA 8.87864156476562 10.2428446934576 6.29743989774292 7.28532575463732 10.6084256331953 5.26551192560246 7.30635391479214 3.98374288716617 6.59306236199822 7.0358312535698 9.97457061005124 6.68054253827559 8.88377719386383 NA 7.45539421437323 5.53693761026225 5.76272282970855 6.51957472135231 6.41075693667657 6.86190477731676 7.08955273713364 4.96309766165464 3.90504049247058 6.41968890143133 4.7234514353525 6.12771272941428 4.46573066326653 2.98993450339725 5.57801326455518 6.65786620152719 6.09069742379689 7.48198659196436 6.4874094639034 2.93521565390392 NA

1.76500422925283 6.49061357975922 3.91575768822136 0.000137706944686487 0.00332149150583806 0.643733992287821 UP
hsa-mir-424 3.87878168572128 5.33336217785816 3.59359006571642 1.49077672952876 2.59531482069485 2.60257640748391 1.8535750879716
4.48467611383252 NA 2.10863741909461 2.26626441460961 0.837818153705987 3.71300332437511 3.29478170461756 NA 2.13585456452729
1.16879149349658 NA NA 1.55622222284321 2.4383683385839 4.67769062159418 5.91640413561452 3.57917081915023 5.02288258260888 NA
4.01162657601022 3.4559832221124 3.64219631983864 3.89683035031931 5.92607479205558 5.30872878483414 3.76063764813 NA
2.63267341612919 NA 5.08163100501748 5.62887164824875 6.31397444638275 NA -1.77373239503454 3.56899284664711 -3.90461556547764
0.000145583416220739 0.00337641538388868 0.641134665808304 DOWN
hsa-mir-99a 13.5954865219256 11.2930270510365 10.1531011823205 10.0402232372927 7.90925106383972 13.7163145549996 10.9979094055715
9.43390526820365 11.1044452040734 9.62475216777484 13.1990463002844 10.8998290652681 11.137672705957 9.6152726536226 8.05755904665296
11.6132412553247 11.4149273470844 10.0275043854409 10.1570958987697 11.1701266457236 8.62002560832653 11.7346167098056 7.77873525315647
9.59325313072745 8.88213825500971 7.39206503290104 7.69832674183842 8.72601855302126 8.28887712324505 7.57276628430424 11.0640609231037
11.6769513263686 10.6546077086436 9.989123143264 10.5144240819058 7.54603911060296 8.14261460944138 9.61764093508172 7.27122580248692
9.72513484482864 1.63360228915516 9.94123340348074 3.81021779304092 0.000202324380731403 0.00451857783633467 0.257592796734522 UP
hsa-let-7c9.83018549626964 7.1824958088193 13.6544634926123 14.6900071108647 8.78156488310547 7.43417687331675 10.7819602639123
12.3305603436069 7.99060848044194 11.5166977364806 11.5116260351781 6.96488053491081 9.61308376718995 8.06361268032897 11.6352265933573
10.7725717470363 9.17454211833699 12.0024380921076 11.3281713705522 15.3276333867219 9.59508364215984 10.9615358278719 8.73114875909384
9.00115250225631 7.58010688896318 9.42117747800542 6.63233835842091 8.05050662629492 8.20110640777623 7.11062796924883 9.21342315086074
8.7069415680693 9.15348524514376 9.41246170380699 12.4171673763621 7.44260856269152 5.59906745226855 7.5463047165449 8.47113419296662
10.7870317794443 1.827604830345 9.615522925585 3.79358876636209 0.000215088331508691 0.00463208085356218 0.200763961950766 UP
hsa-mir-525-5p 2.50459902871729 2.70318736984758 0.644552706387084 2.43113534642849 NA NA NA NA NA NA 1.43734006968586 NA NA
3.58177867512411 NA 2.80681482877614 1.8625643834444 NA NA 2.57649422977772 NA 3.62146224798073 NA NA NA NA 5.41664677886157
NA NA NA NA 6.48953095513118 NA 5.02329195050208 3.16461466696973 NA NA 1.89728643035568 6.50253204304699 NA -2.30474633632239
3.29148948193979 -3.5142896661313 0.000613988563350486 0.0122148848572874 -0.480104923992949 DOWN
hsa-mir-18a 3.58077507648 3.52041049544751 4.06154942684364 8.52788922147326 5.76971079799025 3.66889633461859 5.65956480656486
6.11808532686284 9.60834993773406 5.41653609151166 5.93213505419348 7.10205391496613 4.45640748629366 4.97575630103055 NA
8.10597644334063 5.21106617505133 NA NA 4.17823378530559 1.65048129276699 3.28476815213675 4.59014950246755 4.6044909361003 NA

4.29433019016249 1.54616848385855 1.8084161018685 3.62810394077836 2.68978322235112 4.57843243424787 3.00003412259797 3.9914080474089
5.23920117331731 3.76282216032839 6.44826904495186 NA 5.93891121010189 6.28502913285129 NA 1.679564560436 4.80100575952953
3.52024782089547 0.000578210822360966 0.0120227974442642 -0.65258177752866 UP
hsa-mir-29c 7.91387741606639 6.29944640361075 6.35064484960566 6.86168696877719 11.1794079379574 6.31238747806154 7.5613188792093
9.95471908170162 2.86337910037144 5.67354619005593 10.3896965828819 8.03512640191305 5.37021206688892 8.09743048182414 8.77870303048508
8.56985383251888 6.06137806676437 4.99792780527149 9.48536625891966 9.70047589569534 10.1139938342859 10.3565118169047 6.44175067085512
9.84548090337528 9.19301948709886 7.23667003624658 8.69701948207102 9.4249990316454 9.13589673512988 9.36604855377679 12.5853522831938
7.28149077804844 9.23639508192208 8.14810500105664 9.56459828293366 6.47475489554425 9.45066139445227 11.4090143038295 8.02285659347272
10.6671310221281 -1.60975827296955 8.32770837291378 -3.49323328155656 0.000627962571436001 0.0122148848572874
-0.790050855719061 DOWN
hsa-mir-132 11.2799317395925 9.49719914439631 7.06055568926572 8.58251581396303 9.11662894746402 9.19041650246438 10.8310656467765
7.77238441882414 9.83661050269875 9.56859119321627 10.2308453192174 6.23741543797368 8.29036954775572 8.34052401605744 7.16553743588426
8.53893230591299 7.8930585649486 9.60438928488107 10.8801708011976 11.0797375214668 8.87192082813308 7.25082589927711 8.226406343745
8.52708882375144 7.97291023634272 6.90785845850393 7.44022623964924 7.19365270979226 6.52542938173081 7.03081019664037 8.06045852105236
6.75383261574318 7.90821648425672 7.7889604436966 7.23394159445446 10.3200240216052 7.61035088009438 7.67172984868296 7.27175004999691
7.01139645534717 1.37095449007307 8.36436674666133 3.41534425449028 0.000820388521765423 0.0154591962070172 -1.03575100355614 UP
hsa-mir-708 6.51942245355468 8.60136906486785 8.44570516934851 9.03711102660091 7.43688737660669 7.02655042155159 8.51949810653966
7.8595896680568 6.43365351627141 7.08850641537551 9.70081282881502 5.32169491907653 9.37174461302409 6.97598688905811 7.74725231963103
9.98784220799431 9.04827557912585 5.73526415154871 9.25456627284936 7.4945676868155 6.37961817555329 6.21331258453176 6.52249843006093
6.38765633317573 9.86955061686755 7.66131320108932 6.41031983743324 6.31835035238096 4.77170174823607 6.48131418935321 6.51967941705488
5.58820842684951 5.78963675336338 2.80127597328091 6.09222927295412 8.03827572360485 9.02867800405905 6.40035093841357 6.27939775473844
5.91413053975771 1.40694012069768 7.17684497398677 3.36126178283347 0.000985134237895247 0.0180010892560859 -1.20357445426321 UP
hsa-mir-30d 2.63507563079668 5.01815403739063 6.60523942697594 4.34150055727857 8.4830591411195 3.55905416932423 3.34381403678954
7.76520323410099 6.38878189340802 6.21479698885771 5.69637612458641 6.89916392897016 5.96668618770341 5.49488432818525 8.43270680535012
5.94356338962371 4.75937615274946 4.88943355327164 6.52966182934853 7.49091468207127 6.67027195780966 7.42514182294474 6.76761882939687
7.77896377125211 8.35829274716364 7.17386680804467 9.24100803256283 6.76742123500216 7.1036098028209 6.74185953312014 6.52156261798914

6.45934511714617 7.61445436396157 7.6911061372307 6.5562340789825 7.21299316971367 5.72460192431052 4.66631418477028 8.31080006795983
9.70703898770558 -1.4017529545993 6.52374878219474 -3.34434577495705 0.00104270877949826 0.0184927468834544 -1.25559571658509
DOWN
hsa-mir-195 9.10659220783241 7.77800160362338 6.12515910979904 7.49091878724816 3.50252407334547 8.7358963176629 4.42703192745365
5.16435199270548 5.90617213236089 7.10477524149169 4.09075166268436 7.70207268873142 7.49190108290689 6.08899805133817 5.84926093638824
6.49214219804596 6.98747686862636 7.25202098170949 7.63119562366088 4.66965323207137 8.6833884214001 9.15965448648098 9.22610031711044
7.92746308434784 10.2830162178223 7.60528664783915 8.22110042953103 8.05696546208001 8.48814707077679 6.7930363109714 7.87443076589695
6.32673638785839 6.52424922971625 4.02318392740393 9.87861395632754 7.10692146424177 7.26374293353426 8.72662053091928 6.79675113627866
8.84748857053458 -1.41080003156927 7.18524485176895 -3.31077615442661 0.00116639359404772 0.0200952953488793 -1.35816374422938
DOWN
hsa-mir-197 6.26221165012429 NA 4.93962434113135 6.01044445632871 4.98128826631271 NA 5.73296273911633 8.13514429010838 5.24521202159443
4.31303412209359 2.80722444174095 6.38087940091226 6.05660632689618 3.68155562463027 NA 3.2178480604277 6.80896278324566 NA NA
5.27995062185846 NA 4.43924738162911 NA 4.3924479896897 NA NA 3.31290601603944 3.56943274510934 3.29161213107207 1.90345175965638
NA NA 4.89209873639479 3.47875107909596 4.89839494430537 2.11291397653844 NA 1.89556231534254 6.02707711775886 NA 1.63903859371542
4.59506834589457 3.22511593401698 0.00157596760090544 0.0263974573151661 -1.4786405432527 UP
hsa-mir-1226 1.87463501469881 NA NA 1.17578717201531 NA NA 0.062835236900886 0.810592039052547 2.77404050601618 1.01415090299026
NA NA NA NA NA 0.187098778685992 0.847058054931593 NA NA NA NA 3.87678914829817 NA NA NA 3.71057824109753 NA NA
NA NA NA NA NA NA 2.71665290780231 NA NA 3.52921596111575 NA NA -2.36503435141699 1.88161949696711 -3.05189881886604
0.00279356088938746 0.0443294004289641 -1.73484570290331 DOWN
hsa-mir-122 5.24354628678016 5.47675129040304 6.69686500712932 4.23106042143584 5.58401841779216 9.40913079738398 2.65455683145683
9.54881644532532 3.2222366994342 6.63810618546469 5.64385052452128 3.87983234437058 4.80192539518693 5.71662306569758 7.05159862127343
6.26384136630965 6.63434648776588 3.35458564152178 7.86065266701404 6.44356133427389 4.07211877181365 4.71833932213819 3.85933493350012
3.73155345560297 4.92676134698373 5.38115793999597 3.49385591751063 5.95101123616548 3.23732402661425 4.25369226076436 4.65965832506987
5.03357656121939 5.34817459595222 4.63753306759868 5.2478048296917 4.03418258162548 4.46904203618198 3.69178560713112 3.39811492823249
6.13085026888476 1.30400169089318 5.16579444608044 3.0827565504506 0.00244288360016692 0.0398124002946123 -2.03106583839627 UP
hsa-mir-326 0.216940768579958 2.21678586087001 NA NA NA NA NA 5.07065340295012 NA NA NA 1.53106286615989 NA 2.31671218374429

NA 2.23643640810654 2.54000359100968 NA NA 3.36393764279126 NA 4.52583656401279 NA NA NA NA 4.17042250936398 NA NA NA 5.03475512050616 5.34983966937687 NA NA NA NA NA NA 3.87431949088901 NA -2.15446808030329 3.26520815987389 -2.93261528900575 0.00401277549935858 0.0586043893493939 -2.07538019152201 DOWN
hsa-mir-138 2.44957469775516 3.20724456429886 NA NA NA NA NA 1.10847536351628 NA 2.67534652165631 NA 0.704535773350951 NA NA NA NA NA NA NA NA NA NA NA NA NA NA NA NA NA NA NA NA NA NA 4.44449464939908 NA 4.53061266228888 4.31245232957535 4.10390046049305 4.1026829765385 -2.26979323154346 3.16393199988724 -2.82367063035427 0.00556422641472251 0.0737896858863081 -2.30872877642327 DOWN
hsa-mir-130b 2.17992321004487 2.24428905749505 4.73596371402284 3.74929430617039 5.89597309419089 2.73122304599017 4.53956978869869 2.01112205345116 4.22639961277622 NA 3.30700842022861 3.4204595369812 1.22165021657197 3.83346057482455 8.1690395849551 2.25004058515601 2.81894383997772 0.0366011811783471 7.05407511089673 0.402009421879261 6.07496517367725 2.94690501641567 5.07986277048476 2.42095995938564 5.20766749393267 5.44965051274959 6.19830505146169 6.8535247836567 3.20648410937926 3.61580070694654 3.77952697713129 NA 4.68918120897523 7.07608138552471 5.89742679500908 2.76162797476111 NA 2.35321519645981 6.80045207432465 6.71940916487642 -1.42866387821367 4.106975478666 -2.9698228120826 0.00348293910101591 0.0538515968695537 -2.31881540548934 DOWN
hsa-mir-380 0.554128005435339 NA NA 0.350472558312195 NA NA NA 1.57475441753964 NA NA NA NA NA NA NA 0.755889457401941 1.587701948676 NA NA NA NA 2.92346582267792 NA NA NA NA NA 4.07894127503185 NA NA NA NA NA NA NA NA NA 3.23495072637261 3.00608025808615 NA -2.34627024306911 2.00737605217041 -2.75860715240369 0.00672649062500806 0.0845015384766637 -2.44533844629829 DOWN
hsa-mir-375 9.59074568043077 10.5957393112291 10.1073237828219 13.4332166627854 12.1080437157921 9.04427724143888 8.15710943384971 7.50785562370097 6.03463854559857 4.49418440223077 7.0857089126961 10.2751409665514 5.42462066338354 10.3691530500872 13.0202861805103 11.7381455031613 9.03201324390383 13.1386676259772 12.2310980082155 9.4235239128627 10.6333510499945 3.57345523833038 6.48965721880139 8.89746591280662 9.06135477639503 9.71173339609576 5.47675573519488 11.1593003137625 2.56786685717993 4.71783377079751 9.81646545259063 6.13935834555538 8.54863659799613 11.7473363148003 6.5602445395473 8.17315108580405 4.5943042384182 9.84570615554553 8.5976640693323 13.515611334862 1.64921200317085 8.81596862177594 2.92195050564801 0.00401889113481627 0.0586043893493939 -2.48023057193176 UP
hsa-mir-191 8.23372419093405 9.43881968909815 8.3542694782844 11.2122473637575 8.02218889111651 10.6863108794982 8.59494025177385 8.99797053532979 8.09914487019467 10.5463668962698 5.64380715517843 9.92192467599421 9.33471008081175 9.23669979582261 7.67640684244763

7.10871264217704 7.05016505935698 10.3046672823537 7.80929078134384 7.55025425053768 8.00002421719698 7.76374198886244 7.67401540645716
8.03330185553085 7.68940533405047 9.37275482412896 7.77589133153424 7.04639865263911 7.8432828595226 7.91762741887319 7.92502113204382
9.19639009944452 6.70484898310383 5.74187771437445 6.49070243351544 6.26851290261148 6.84386172422846 10.042086407405 6.21296026000732
5.26920662931292 1.20053547187188 8.0908633446781 2.91324693999352 0.00412633227317817 0.0586043893493939 -2.5039326039226 UP
hsa-mir-34a 13.2130369868242 7.27638992032286 13.4430252794234 9.74579015965258 8.29606009112141 9.85823797116649 7.89039001880122
11.014052318808 11.8455539861444 7.69399414107181 13.2322416080681 7.78441024238077 9.31511429413597 10.6223690004128 8.59089740064059
14.6240189766373 6.03654698578999 11.0460635462632 10.6078073491607 9.31955661046936 8.30487590322808 6.2545143926977 6.3341818564703
7.38913216953832 9.36030799802775 10.3199451435053 11.0806032274546 8.98347812766797 7.46668913694705 10.9630574618746 8.42095438945427
7.78432615581341 10.0557451494749 9.22764273263875 8.34733558435543 10.6188691748526 8.87634435959012 8.20635192950029 8.16833936159205
7.99904900388054 1.36469068143655 9.39043250364648 2.90904927088631 0.00417908580766822 0.0586043893493939 -2.51534142554926 UP
hsa-mir-1322 0.0430521240216024 3.5113293005118 NA NA NA NA NA NA NA NA NA NA NA NA NA 0.142117511690542 NA NA NA
NA NA 2.78348378226602 NA NA NA NA NA NA NA NA NA NA NA NA 4.33789579275759 NA NA 5.22925655760813 NA NA
-2.8847123988026 2.67452251147595 -2.69046128000739 0.00819401071419051 0.0950190088587861 -2.55474009021641 DOWN
hsa-let-7g11.4160672002211 11.7013117875198 12.8353913680629 14.7068134420381 12.2221450158173 15.4303337044285 11.8678092389033
12.8903630126683 11.3476740948212 10.9994069660561 9.08938646081066 13.9564654832489 8.23766735153179 12.0207286978098 12.8092373223087
10.3313059793734 16.083417899313 11.3251319089167 14.5665220738569 10.2248777086937 11.6759102778305 10.9525139469101 11.8287968997823
10.1073084053629 11.8573129367654 11.5284761062687 10.6418275188919 11.5466742833508 11.2360638135956 10.5200500942459 12.590518542999
12.4839390295735 11.1719240914065 8.67436790143392 10.5864268736763 9.53489461784672 11.7089593086116 11.5173860174039 7.01171132969344
11.2737385042601 1.28066281082455 11.5627714304077 2.86100619472847 0.00482844845688975 0.0661716913523754 -2.64487203651424 UP
hsa-mir-638 0.303687695335751 NA 3.16775757820236 NA NA NA NA NA 4.30341844566626 NA NA 1.63606280387953 NA NA NA
3.29459293759489 NA NA NA 1.6409416502984 NA NA NA NA NA 4.589420435943 3.98719594880221 4.48360053512969 NA NA
5.15499433620723 NA NA 4.62678993856646 NA NA NA 3.52306850315684 NA NA -2.00310143113804 3.39262756739855 -2.70096572914673
0.00790185598049595 0.0934278265929227 -2.64833912316879 DOWN
hsa-mir-1912 3.76647596157256 3.87735091516389 2.33773025387517 1.58487829051198 NA 3.21236790833965 3.1745195955643 4.34544804633934
2.92276174431373 6.79434000614912 2.55867226027939 1.43929613708419 3.46910969626109 3.83048999427334 NA 2.48721664093663 NA NA NA
3.39892126909234 2.32484641753707 4.82418953623084 NA NA 4.28031937005306 NA 3.42018627338949 NA NA 5.66512776477458

5.39668624323656 6.23984693172175 NA NA 4.09403058375318 NA 4.95112017926544 5.14675069185581 3.65657049671418 5.91994450203179
-1.3799963345632 3.89330361890076 -2.77142119462649 0.00636054215396235 0.0816044025284957 -2.72528815712813 DOWN
hsa-mir-216a 8.50605796095827 11.00293545091 9.16929300785359 6.72666295395778 13.3026961600664 3.70527232218688 12.0588688883238
7.7949907778269 8.81813108369653 7.27813021176282 7.40745967977662 8.04300835108709 8.2819154247659 5.35435494693592 16.1891089107537
7.37422335501247 7.09430988605482 6.06917166330868 16.3649396004105 5.16947720436665 9.84158822214348 6.38944623436144 5.05968374783208
8.28566748381106 5.15129846479238 6.08255028102812 5.08701939625139 10.2393890859493 4.28739200655449 2.75994967611767 8.01686289861905
6.76214923748022 9.66553306520272 11.2094093202398 8.81091434958718 6.86889246988902 5.32734531478253 5.25335389098858 4.65436755883607
12.3109336137629 1.68236307608929 7.94436885395612 2.80931517857521 0.00562906393162549 0.0737896858863081 -2.78208198728959 UP
hsa-mir-1178 5.33546870145852 5.60358536010086 4.19421609963099 3.25693945854242 NA 3.50842072248184 1.18398590207207 4.92603798852964
3.9582626233591 2.76000155967667 3.87189599160104 3.79834122677374 NA 3.4951738443522 NA 3.10658954561431 4.46308624096814 NA NA
2.72240508414236 3.92848872494615 6.08508389624617 8.70126788336519 6.89337744729816 3.78221256907425 5.23125225710729 2.62018592930294
5.43240314371044 5.61353033386401 7.40580275382024 NA 3.42503953378454 4.6742693724039 NA 4.01544964894765 NA 5.72542683226929
4.22644217460081 2.86042437172947 NA -1.29316369790915 4.41306668457337 -2.70713972732222 0.00762901216826997
0.0934278265929227 -2.94526469665515 DOWN
hsa-mir-127-3p 4.0412139147414 2.07855548264076 5.22670879640647 2.55445027279825 NA 4.67879478932112 NA 3.56410253255987
3.04587665070187 4.62316886989712 3.2376508546568 3.90805691243598 2.20224236613708 3.13094462654169 5.15345150206983 3.2011894841672
3.94021693305841 5.26827333905699 NA 2.17037002121787 4.95515845058417 5.36361651250864 5.64539552243068 7.61677126857412
4.9475888198678 4.71929721505401 4.35005185918167 4.1933001636394 2.52367639991817 NA NA 6.27294275161783 6.51123552465275 NA
5.80969727684945 2.75113426034247 3.28129458121738 2.31655143119897 4.55589520183164 6.73338141001376 -1.20716007653378
4.25212517640858 -2.69759736790244 0.00782148449122422 0.0934278265929227 -3.00583137056224 DOWN
hsa-mir-2052 NA NA NA 3.28614308150224 NA NA NA NA 5.13965254574395 5.12210171062663 NA 1.44450105673486 NA NA NA NA NA NA
NA NA NA NA NA NA NA NA NA NA NA NA NA NA NA NA NA NA NA 0.242164079782929 NA NA 3.50593551886899
3.04691249487812 2.38670703876989 0.0186393473753917 0.184938949631023 -3.16604186292196 UP
hsa-mir-32 7.95703294801218 6.01943736171352 2.67843602928762 5.28336556579968 4.80905885627823 6.20331049821425 NA 5.10441142542671
4.9440020408328 6.71009338577758 4.74733641216635 5.67505849513373 3.49497513309463 2.38381811584358 8.31203250775297 3.70960074437898
5.2958132894608 7.79484647340086 7.64266558172321 6.57227666781151 4.28928911281444 4.01738725004224 4.59441770915628 NA

4.97393741217124 4.59314662140838 2.92080818299382 6.73248429841667 2.96768890864389 4.49883254869402 NA 4.8606941431417
3.71246307096132 6.21093327000941 3.93053155877317 4.43637576393727 4.84601144993602 3.80151251049001 2.9036031678446 NA
1.17407583107307 4.98965801420955 2.63487067641005 0.00932871472994028 0.106136131738755 -3.18422800663812 UP
hsa-mir-513a-5p NA NA NA NA NA NA NA NA NA 5.19872957768338 NA NA NA NA NA NA NA NA NA 3.30298298291761 NA NA NA
NA NA NA NA NA NA NA NA NA NA NA NA NA NA 0.508722120925362 NA NA 3.74213415937513 3.00347822717545
2.3504597920653 0.0204926558584258 0.19930760455856 -3.24377826718532 UP
hsa-mir-503 1.79346281314436 2.84062992537323 NA 3.50855272452617 NA NA NA 0.0236142460336155 1.40186430514229 NA NA NA NA
0.979597133063631 NA 0.104683582510718 NA NA NA 1.64244633045051 2.20148341991415 NA NA NA NA NA 3.83455662104507 NA
NA NA NA NA NA NA 4.85159031443997 NA NA NA NA NA -2.09235373593583 2.10749831051307 -2.38577254555969
0.0186015470940248 0.184938949631023 -3.27878735265759 DOWN
hsa-mir-668 NA NA NA NA NA NA NA NA NA NA NA NA NA NA NA 0.0797522766974312 NA NA NA NA NA 2.68532021705376 NA
NA NA NA NA NA NA 2.59140468584478 4.09261084671975 NA NA NA NA NA NA 4.30113950935555 NA NA -3.33786653804603
2.75004550713425 -2.3129691333024 0.0225106805209168 0.204269270499352 -3.29910099684551 DOWN
hsa-mir-563 NA NA NA NA NA NA NA NA NA NA NA NA NA NA NA 0.130978758979801 NA NA NA NA NA 4.64127356441623 NA
NA NA NA NA 2.90804123393604 NA 3.20250784989143 NA NA NA NA NA NA NA NA NA NA -3.4529621237681 2.72070035180588
-2.31106047782458 0.0226356349642693 0.204269270499352 -3.3045283018868 DOWN
hsa-mir-124 NA 7.63715434463369 NA NA NA NA NA NA NA NA NA NA NA NA NA NA NA NA NA NA NA NA NA NA NA NA NA
NA NA NA NA NA NA NA 5.94834129454629 NA NA 2.00489333346581 NA NA 3.66053703062764 5.19679632421526 2.26387699001878
0.0254993928423694 0.216565265971109 -3.39140181077832 UP
hsa-mir-645 1.09178804685385 NA NA NA NA NA NA NA NA NA NA NA NA NA NA 1.31915184788196 NA NA NA 1.21110449697788 NA
NA NA NA NA NA NA NA NA 3.38992464948562 4.3441540201131 NA NA NA NA NA NA NA NA NA -2.65969120422813
2.27122461226248 -2.26901308600311 0.0251412168372366 0.216565265971109 -3.40192274610255 DOWN
hsa-mir-625 2.73232927823368 5.45744864447523 NA 7.58824754175619 NA NA NA NA NA NA NA NA NA NA NA NA NA NA NA
5.4589707833891 NA 2.50536888083028 NA NA NA 4.03232722593925 NA NA NA 2.62177786737584 NA NA NA NA NA NA NA NA
3.51918761644936 NA 2.13958366431487 4.23945722980612 2.30891082782365 0.0226965856110391 0.204269270499352 -3.41715631149593
UP

hsa-mir-663b 3.24400925218287 2.37240421929976 2.50900744955165 1.7386423951164 NA NA 2.60493599765371 NA 3.88068179353733 5.34853665615356 2.54022680776439 NA 3.67043220939312 1.76937508539352 NA 2.74103047465624 2.3622691737807 NA NA 1.83063816925344 2.2521174753061 3.20412161815237 NA 2.76400939529079 4.85143350239834 2.46356743941337 4.87764034918857 3.31074860284347 4.37917586856655 4.26116195077534 NA 6.02688747438718 NA 5.32270377254716 3.92726225098803 3.30418219970843 4.83903699279895 4.20424852649153 3.77744684221293 NA -1.16903673295246 3.46130806706227 -2.49828744677245 0.013649326961433 0.148514945409858 -3.42349806713713 DOWN

hsa-mir-369-3p 3.83966691919187 5.29719766569726 5.65998187130673 2.29727088164005 NA 4.28035565690297 5.40328126205268 4.66087685936714 5.11467488543796 5.50908971302615 4.84147994220206 3.10569321034634 4.89024150966137 4.94203317698056 6.24333469893361 4.46028150744297 6.14262669538047 4.05553063281661 NA 3.54901642019597 4.78802329609243 3.95130791017613 2.81035776638448 2.79428084498746 3.07493515716943 4.7805902574528 5.3576441995438 3.3600232855935 5.16559329291192 4.53188356892888 3.07487520153267 3.4815522170349 2.42422915313703 4.26030520866258 4.17284664636209 2.7045732050206 NA 0.813027654942634 3.88700926315816 3.88152761152735 1.03478799219864 4.15154646619464 2.51866079497317 0.0128544356454769 0.143541198041158 -3.478549067275 UP

hsa-mir-1286 0.952592210324086 2.14091942849081 NA NA NA 2.13853847252131 NA NA 3.28200088978421 4.07191344689431 NA NA NA 4.44322071214999 NA NA 1.23709946908007 NA NA NA NA 3.97622388737818 NA NA NA 3.9095141038325 NA 3.90660366631181 NA 2.74668442935242 5.4077032772185 3.27886996571763 NA 5.00574503891781 4.70771587232906 NA 4.69875755657186 2.30557239213302 4.98611258499655 NA -1.47503101950159 3.51087707800023 -2.38176500502877 0.0187085836276823 0.184938949631023 -3.49250336120569 DOWN

hsa-mir-652 NA NA NA NA NA NA 3.61865967017606 NA NA NA NA 0.444107910580384 NA NA NA 3.88528061351598 0.142861958598283 NA NA 1.27170533703259 NA 1.59348477730097 NA NA NA NA 3.96588616630889 3.89702423736709 NA NA NA NA NA 4.13492230332917 NA NA NA 3.38079843003569 5.13166544196129 NA -1.81144046140319 2.86058153147331 -2.2786381492162 0.0244498461079448 0.216565265971109 -3.56314392599634 DOWN

hsa-mir-181a 9.11398990753624 7.03088679152031 10.5879342146996 8.01542578746563 6.35489162961648 7.8715940338898 9.32137612431226 7.10460474453471 9.76001567691971 7.65471164766682 9.41239779096139 10.2579903427091 8.54550890232712 10.2937469324348 9.2570635713151 3.91312445199098 8.62230217647961 5.76926026833014 7.88983514354864 7.28886085290932 8.80620991349988 8.24405898643879 7.39582336919741 8.31876569015709 10.1444084939105 11.0184800158253 9.79785639859608 10.2143295741509 9.00123050381404 8.47023059143241 9.24212576731379 8.2129642577016 8.61613904352515 10.5827680583268 9.89069933487347 12.6435838997736 8.91235618116239 6.85286551049942 11.0084922207369

8.17026269440747 -1.07390647570876 8.74022928741277 -2.48905725423873 0.0139043555072153 0.148514945409858 -3.5815779879026 DOWN
hsa-mir-453 4.37844088101261 2.00896405896475 3.40739514897867 3.93477742111466 5.09394785379829 4.30426737448644 4.33726071930663 4.84141984008645 4.08541465907442 4.69175450796392 2.84500119654201 4.47063802778376 4.36483983047217 6.16676176308294 3.79290859759364 5.04468220264796 4.25951877773492 4.68910495964469 5.0432243764085 2.63935988167359 5.39651492669763 5.44626951537615 NA 5.59227133105799 4.03995018535257 5.64580162260857 5.870267684494 4.41941826618625 5.00777222245631 4.75059023625489 5.11377192749783 NA 3.54402993434855 4.96076330430575 3.91793772352137 5.39084166917227 7.84411224397098 4.66922665807535 5.70543646421408 6.41193783670741 -0.987066660098004 4.68754199554392 -2.47657772213684 0.0143949670296701 0.149658019291226 -3.58816561489546 None
hsa-mir-143 14.2915734058246 14.5726102857307 15.5785965260347 10.1592185009322 10.6842073043936 11.1145058308294 12.6050616066045 15.5280260110958 14.8163883985 9.22277892055727 12.0926516250195 14.0301304899098 9.62650612546106 14.9111932111414 10.0704833471937 12.1090412166711 10.7208320643935 10.4620352365301 12.3527834048331 11.8166267446619 9.94590232227997 12.9594311914741 15.9328716428457 12.6394537668296 11.7533481092392 10.5399189821316 11.4532905534281 9.9267397436152 14.0454250284566 11.8985552532933 8.33220326966042 9.48261552985006 12.1551927976556 9.96225045740599 12.0674882151218 11.2843887934439 12.6677146196181 8.401943833602 7.98882249743405 9.28898969186076 1.2019351978536 11.7372949138891 2.4854908744104 0.0140387261830214 0.148514945409858 -3.58998387092718 UP
hsa-mir-133b 4.37111909826623 4.81255473866115 5.01116782282654 NA NA NA NA NA 6.57878739963419 3.66294565168351 4.60299471903108 3.16069006955917 NA NA NA 3.7007519736919 5.17028540884256 NA NA NA NA 2.28214221662946 NA NA NA NA 0.122557298960562 3.73748968309698 NA NA NA NA NA NA NA 4.28498197663389 4.44982309105973 NA 3.19553130526089 NA 1.55138983608156 3.94292149692252 2.26812254468979 0.0250450035897526 0.216565265971109 -3.67498346891762 UP
hsa-mir-493 1.96162952287008 1.9908052332741 2.65780352635931 1.8636732958693 NA NA 3.9944068549909 0.532221108256395 NA 5.28452841431875 2.84899535502496 2.8869184564006 3.3607804073832 2.92417246349423 5.42912224683839 5.54009209381862 3.31875580314111 NA NA 2.20474881626225 0.471550862020059 4.92184980973388 4.03143038590408 3.9294288587267 4.89964989428401 NA 4.29460784239196 NA 4.11090089099645 3.44665760212593 5.80390421651288 6.45724873419058 2.91744461624542 NA NA NA 4.77262920059438 3.63702508748774 4.37651642933801 5.4837097844236 -1.117060041111573.6784402604426 -2.31137599845645 0.0222775789027077 0.204269270499352 -3.86473195482021 DOWN
hsa-mir-363 1.24168934832699 4.45255377680433 3.4068372482916 3.33918339678867 5.31097683737348 1.37181769451993 4.91052754916123

4.91253259899531 5.57461560480456 3.11614800926893 3.6001413657542 3.85709140463502 4.93710619553158 2.74708896454209 3.60696870505429 2.72659413456447 3.26126395766377 2.92174719182819 7.13654946448764 3.77366543211711 2.75427966490266 3.97174916675229 4.79763931401898 5.0373559324242 NA 2.93119869062476 4.9670804002544 3.48155684493697 6.90323112437575 5.77668268881211 4.59101863946741 6.03930735480355 4.96943193631154 4.81868430277369 6.23134609560808 4.75733552151089 4.9263677315475 3.43105436273446 4.9554484891252 5.3944425517966 -0.965282467173333 4.28052076136652 -2.33526913459985 0.020868260180737 0.199739061729912 -3.9227499472528 None

hsa-mir-1538 1.96754963252848 NA NA 2.47328874227449 NA NA NA NA NA NA NA NA NA NA NA NA NA NA NA NA NA NA NA NA 5.38080118385954 NA NA NA NA NA NA NA NA NA NA NA NA 4.10334063508706 NA NA -2.52165172207182 3.48124504843739 -1.95282475177482 0.0533029163095507 0.36228510752199 -3.92413134909619 None

hsa-mir-519e 2.84019289210559 NA NA NA NA NA 3.86254181840601 4.91444863864141 NA NA NA NA NA NA NA NA NA NA NA NA NA 1.79269089918526 NA NA NA NA NA NA NA NA NA NA NA NA NA NA NA 1.3655845003323 NA NA 2.29325674995889 2.95509174973411 1.94733763219502 0.0539433879991713 0.36228510752199 -3.9659462546003 None

hsa-mir-646 1.71085248287795 NA NA NA NA NA NA 4.03019739361973 NA NA NA 0.999351118566857 NA NA NA NA NA NA NA NA NA 3.76675831414049 NA NA NA NA NA 5.3593167115535 NA NA NA NA NA NA NA NA NA NA NA NA -2.31623718115882 3.17329520415171 -1.94627497224599 0.0540724041077598 0.36228510752199 -3.96767818743788 None

hsa-mir-372 NA NA NA NA NA NA 0.00960874120321809 NA NA NA NA 0.112923208013647 NA NA NA 1.221345990535 NA NA NA 0.441101165162208 NA 2.02896884132337 NA NA NA NA NA 2.69469148375842 NA NA NA NA NA NA NA NA 2.43490717739826 NA NA -1.93994439126483 1.27764951534202 -1.99188661856346 0.0487232243658516 0.345411533246168 -3.97483986935712 DOWN

hsa-mir-634 1.14659002106196 4.05995245325498 NA NA NA NA 2.32406225422463 NA NA 3.85816394178958 NA NA NA 0.160699516248965 NA 2.36643340777338 4.72333018164878 NA NA 2.90009805709554 NA 2.16200043772834 NA NA NA NA 4.91940438365039 NA NA 3.93991633228927 4.60195410164147 NA NA NA NA NA NA 5.00174275652035 3.55794802064749 4.58286980205235 -1.4169888900813 3.35367771117517 -2.10413513481413 0.0373787036718532 0.296570504133256 -4.01803398894634 DOWN

hsa-mir-147 0.488726468463458 NA NA 4.03513465452854 NA NA NA NA NA NA NA 1.42385996604665 NA NA NA 0.191435358934328 NA NA NA 3.82825014062435 NA 3.80874988601559 NA NA NA NA NA 3.85654080641714 NA 2.14946568631851 NA 4.97346526036984 NA 2.96464740636658 NA NA NA NA 3.88746018675229 NA -1.61324022098719 2.87343052916703 -2.03630693649474 0.0439119650892801

0.330986436860449 -4.03585732094108 DOWN
hsa-mir-603 3.58954970652788 5.69364756362467 NA 7.62591055231544 3.94682602807281 NA 3.56273022212366 5.03320070871702 2.91518829287283 6.82557322309924 4.8409153693825 4.21883564189727 6.01679057224104 4.55567334329419 6.49792230773479 5.92222296610201 6.23114319563959 NA NA 6.41842442891247 2.5947397906016 4.83640446796215 NA NA NA NA 3.5924660600575 4.55642618596508 2.74242569098136 4.30241539319264 6.08525860994361 NA 3.19595938729954 4.94313857509072 2.70057808521148 4.2279687995223 NA 4.22963202975329 5.24762185833188 5.50709427351336 1.04611468927223 4.75522277766613 2.21729548419412 0.0282214230967491 0.229966461180267 -4.06439468688321 UP
hsa-mir-877 NA NA NA NA NA NA NA NA NA NA NA 2.59778501735267 NA NA NA 1.83952212970854 NA NA NA NA NA 3.69972063150645 NA NA NA NA NA 5.53024501824791 NA NA NA NA NA NA NA NA NA NA NA NA -2.39632925134657 3.41681819920389 -1.85079461861969 0.0667999435092916 0.392733248056965 -4.08230151671402 None
hsa-mir-517b 1.04340249934868 NA NA NA NA NA NA 2.81110648706531 NA NA NA NA NA NA NA NA 1.17121505583312 NA NA NA NA NA NA NA NA NA NA 4.30180272193365 NA NA NA NA NA NA NA NA NA 3.40905730664474 NA NA -2.18018866687349 2.5473168141651 -1.85084250475344 0.0667702440527531 0.392733248056965 -4.11962935712304 None
hsa-mir-1271 2.67199021492087 NA NA 0.583151155815105 NA 4.01517777838193 NA NA 2.72413069567593 NA 3.15392691322427 2.16787360744017 NA NA NA 2.65230839053486 NA NA NA 3.19000467563029 NA 2.04328129269042 NA NA NA NA 5.14037503767497 3.81875605895272 NA 3.37723441063528 3.10052768081309 NA NA 3.75384722305205 NA NA 7.45454427893564 3.25175201135568 NA NA -1.3477193203108 3.31868008910833 -2.06348502384196 0.0411286002314453 0.313930961260273 -4.12234747282106 DOWN
hsa-mir-34b 5.0671282192528 5.97263124019435 6.36388954802228 3.73978780945821 5.08881786264291 3.29823417717022 5.61388533254683 10.2844578579033 3.58203471930862 2.62444010182076 4.13751661244021 4.39469946494307 6.77828772180437 1.68584577753171 6.67225884558428 5.97338806111173 4.42345308532027 4.98128905003777 12.3745365491873 2.94943910852276 4.64375043174233 3.5967076963849 3.79178850990119 2.47683049370559 3.89767775953934 6.01777127693304 3.07915120528524 1.05775618821927 3.75942821845635 5.119914881738 3.80423495931004 NA 4.17844251225613 4.80030843213337 4.59421222490157 3.4534677440248 7.00846745545867 4.65647325295536 4.72237082247335 5.49924653323439 1.08145892046898 4.77343645496043 2.23674531340897 0.0267932356330447 0.223766589513547 -4.13737507591163 UP
hsa-mir-549 0.977579566191284 NA NA NA NA NA NA 1.35818908295521 2.49840077049586 NA NA NA NA NA NA 2.29004975914354 NA NA NA NA NA NA NA NA NA NA NA 4.34928099362184 NA NA NA NA NA NA NA NA NA NA NA NA -2.56822619892537 2.29470003448155 -1.78366052069887 0.0771286695847838 0.427899114157902 -4.14230315557748 None

hsa-mir-21 16.5885127998074 12.6375852693975 14.7318336416324 16.8876647171334 11.4586904298516 15.1942936851726 13.572032785462 15.9616668810819 16.1385341374899 17.8032927554052 17.7063052934073 13.2546091829918 13.424577467551 17.7577754836415 14.9005560518165 15.4179436471983 17.8546136820551 15.0252096923765 11.716331076225 14.4778559241553 15.9276980683462 12.0633275063663 13.1864309646363 13.2164525116393 15.4263381641671 14.085925830548 15.7434038544463 13.8778302225412 13.9615097010455 13.6373225261552 13.8730343005268 13.4757545570218 13.0976372072228 12.1760155760044 13.7758325548832 14.945969350349 15.2611541121432 15.4531773903934 17.173410670731 12.1336194632727 1.00090200357063 14.6250432284073 2.23218984565209 0.0270894876193846 0.223766589513547 -4.15844174069872 UP

hsa-mir-939 NA NA NA NA NA NA NA NA NA NA NA NA NA NA NA 3.90428116121596 NA NA NA NA NA 0.742660325670083 NA NA NA NA NA NA NA NA NA NA NA NA NA NA NA NA NA NA 3.16162083554588 2.32347074344302 1.72027307587508 0.0881577512111412 0.458268310175156 -4.17501327729266 None

hsa-mir-505 3.74862882144282 3.90354099233257 NA NA NA 4.21469787040426 NA NA NA 4.81842888006497 NA NA NA NA NA NA NA NA NA NA NA 3.38376323716181 2.99518939642238 NA NA NA NA 2.1448478030938 NA 2.25254460339979 NA NA NA NA NA NA NA 1.9509537148306 NA NA 1.62586439007948 3.26806614657256 1.91165977456876 0.058335282602688 0.366418493848134 -4.19050349311189 None

hsa-mir-323-5p 2.32836920243706 NA NA 0.942713645689358 NA NA NA NA NA 3.45020219119802 NA NA NA NA NA 3.41188185435183 2.86591979896535 NA NA NA 3.83958257906386 3.2004304689814 NA NA NA NA 3.66175634502568 NA NA 4.04961642119653 5.84751067891468 NA NA NA NA NA NA NA 3.90149588356052 NA -1.48358139092879 3.40904355176221 -1.91743015491625 0.0575509534752846 0.366418493848134 -4.24971963285166 None

hsa-mir-220b 2.77523067980196 NA NA NA NA NA NA NA NA NA 1.38839769241254 NA NA NA NA 2.37291810104236 NA NA NA 1.8464330309807 NA 3.75627592085542 NA NA NA NA NA NA NA 3.19237490633733 4.10213719325081 NA NA NA NA NA NA 3.96172985524025 NA NA -1.65738459286156 2.92443717249017 -1.84378208067436 0.0677350875587469 0.392733248056965 -4.27075472427667 None

hsa-mir-337-3p 4.15904870479018 4.21345505786002 5.14908566724362 2.47407808504989 NA 5.86398324642607 4.5509621296374 3.13156641545302 2.46224254278307 3.20941615093358 4.47921487149464 3.74146145459586 3.43641871407524 4.60393808976259 NA 4.15086926278747 2.36668756132738 3.701265557274 NA 4.7619496580713 3.64006507496914 3.49857463615093 6.7298209221198 6.45954152269233 6.04126499390007 4.40442851719806 3.86097326146175 1.58379114341921 5.55247620825346 5.14530496357642 4.36876115405849 4.10372615588496 4.64190234587103 3.74541458660751 7.11659065690489 6.35310753233509 6.87571095441157 3.34347703364654 4.45396145994376 NA

-0.928680999616544 4.39929267480473 -2.1525824982819 0.033000837939815 0.265326737036113 -4.27769093181355 None
hsa-mir-1308 2.33717233463828 3.35394823483015 1.00761093356813 2.47953671626949 NA 4.90422893929459 3.39896952983471 NA 4.22087360477448 5.62764736925651 NA 1.94635696915166 NA 4.47167862635757 NA NA 4.37628081846337 5.16002945553351 NA 2.43441343138998 NA 3.93906256534359 5.62390419784455 NA 4.86660909493392 NA 3.74306968189581 2.29012220448361 NA 3.33657901689508 6.3772055663479 5.89733098445743 NA 2.38652915400942 5.11285616346549 3.26927343466792 6.97987573306255 6.00666317005575 NA NA -1.08541030800774 4.05953184349329 -2.07660992310817 0.0397271813991315 0.307121671585593 -4.29216391838759 DOWN
hsa-mir-212 NA NA NA NA NA NA NA NA NA NA NA NA NA NA NA 1.13370340272354 NA NA NA NA NA NA NA NA NA NA NA NA NA NA NA NA NA NA NA NA NA 4.10248601629903 NA NA -2.96878261357549 2.61809470951129 -1.61534765359623 0.109062407466682 0.543509352912472 -4.29840880948813 None
hsa-mir-599 3.32047210657396 NA NA 3.17859388698329 NA NA NA NA 1.1965845827724 NA NA NA NA NA NA 3.25267835233936 2.80503073571986 NA NA 1.23093733689515 NA 4.25688462313564 NA NA NA NA NA 4.41331223329885 NA NA NA 3.68759827652742 NA NA NA NA NA 3.05103664549802 4.31556993892179 NA -1.44749750992901 3.15533624715143 -1.88044768125009 0.0624618085241816 0.384331332041648 -4.31379713512105 None
hsa-mir-1253 1.87002720881016 NA NA NA NA NA NA NA NA NA NA NA NA NA NA 1.78795122378332 NA NA NA NA NA 4.64440461024753 NA NA NA NA NA NA NA NA 4.04444003439838 NA NA NA NA NA NA 3.59051304316113 2.74405596808805 NA -1.92686419767703 3.11356534808143 -1.73367208413673 0.0856454256376357 0.458268310175156 -4.32375143830529 None
hsa-mir-524-3p NA NA NA NA NA NA NA NA NA NA NA NA NA NA NA 3.83926682792404 NA NA NA NA NA 0.937714361552657 NA NA NA NA NA NA NA NA NA NA NA NA NA NA NA NA NA NA 2.90155246637138 2.38849059473835 1.57876698243476 0.117223151868406 0.560996512513083 -4.33971201135464 None
hsa-mir-1272 1.77843375676183 2.15960209230074 NA NA NA NA 2.77710255238101 NA NA NA NA NA NA NA NA NA NA NA NA 0.838037357728505 NA NA NA NA NA NA NA NA NA NA NA NA NA 5.27743371584193 4.61656772465538 NA 3.13240775162579 1.17227885697522 NA NA -1.66137807248156 2.7189829760338 -1.79982452056437 0.0744550579568673 0.420635000872264 -4.34220262324013 None
hsa-mir-499-5p 1.91760731192762 NA 2.52468736001039 1.37852584202547 2.29984231530018 NA 4.03434835263075 NA 3.35555631880569 NA 3.24715257538343 2.13950107072739 NA 4.07824974952056 NA NA NA NA NA 3.59087746231816 2.15849015319409 4.12392978298324 NA 3.06797175946123 NA NA NA 5.54049337100875 4.57555999205638 NA NA NA 3.58785568249197 NA NA 4.24300404754971 NA

2.68060510938109 2.69969723031018 7.418247746498 -1.1529506516285 3.43311016167921 -1.98495985881144 0.0492626730666176
0.345411533246168 -4.35901383022202 DOWN
hsa-mir-384 2.43156508164877 1.70783144192533 NA NA NA NA NA 1.99839156875479 2.01920490817498 NA NA NA NA NA NA NA
1.99273671875648 NA NA 2.41347007623815 NA 2.74437885586009 NA NA NA NA NA NA NA NA NA NA NA 6.1703816377951
2.20890335629719 NA NA 3.26387903512489 3.25244153005327 NA -1.43413025044302 2.74574401914809 -1.84678962540641
0.067231646389163 0.392733248056965 -4.37109546266563 None
hsa-mir-219-5p 2.84522228699428 1.3439668936944 NA NA 4.50094876139069 NA NA NA NA NA 4.33771713491734 NA NA NA NA
3.34723802483753 NA NA 6.83727043051163 2.68273662104811 1.17368567916201 NA NA NA NA NA NA NA NA 2.13020921034956 NA NA
NA NA NA NA NA 3.48416514977663 1.9885197229477 NA 1.50515508135445 3.1519709014209 1.82169723227225 0.0709819013017499
0.407638918904335 -4.38837216130066 None
hsa-mir-633 0.0504955630403563 NA NA 0.850752643196884 NA NA NA 3.68693769448617 NA NA NA 3.43927879387903 4.95903001540601
NA NA 2.96977682100417 NA NA NA NA NA 4.31889445687223 NA NA NA 3.69723406415397 5.1294005342813 2.51589999186694 NA
4.16276842077696 NA NA NA NA 5.76237420806427 NA NA 2.32406447217033 3.92397537306521 NA -1.3199478516543 3.41363450373313
-1.85214879903003 0.0663928932425026 0.392733248056965 -4.44727990306757 None
hsa-mir-221 9.9501862425696 9.10561363148506 12.9948538417909 6.4295247127441 8.56635907833301 8.29441666969892 10.3734487111892
9.360071391022 9.22243424177242 10.0851095955258 11.3082520217778 7.94630998550657 10.1615978641524 11.9649164658702 5.25825809175992
9.13165487785516 11.5324499978479 8.73226285003783 6.69352762003249 9.8910347218877 7.80018263055599 8.50362204272784 9.36579437172305
8.32215882717899 8.00673578134888 9.23479694894242 7.54268498662513 5.76208327579699 7.85779156107717 10.5435414890141 7.09000663119687
6.58980130752305 10.6935332274845 8.03926257841395 9.28808539157748 10.2513685936136 8.35071198752297 6.5699842928886 10.0753250284892
8.68478497342376 0.92150133428672 8.88936346349959 2.08496882127392 0.0387725680386499 0.303634526328648 -4.46261479123213 None
hsa-mir-558 NA 2.55690715636065 NA NA NA NA NA NA NA NA NA NA NA NA NA 0.500283627734279 NA NA NA NA NA NA NA
NA NA NA NA 4.0324943613849 NA NA NA NA NA NA 3.22933887137849 NA NA 3.01381426830998 NA NA -1.89662044164366
2.66656765703366 -1.60863761359798 0.110450212511241 0.545913755280968 -4.47303768462346 None
hsa-mir-1277 NA NA NA 5.9521263352623 NA NA NA NA NA NA NA NA NA NA NA NA NA NA NA NA NA NA NA NA NA NA NA
NA NA NA NA NA NA NA 3.39224934885954 NA NA 3.88373865314435 NA NA 2.31413233426036 4.4093714457554 1.45997438987207
0.147084774252367 0.643844495264813 -4.51756190865122 None

hsa-mir-381 1.85353914122732 3.45184469264039 1.8436464347667 4.28429853257836 NA 2.28700468887411 1.54476188627191 2.81841831888437 4.82485715348635 NA 4.52883772427567 5.92310384530181 1.69562071638913 2.99111572827198 5.82515365754448 1.67299438310977 3.40442667385436 2.93626804722874 5.40107450981191 1.15222355398108 3.56742192462925 4.58959758149609 6.08259404224391 5.27327802959521 3.77904553440809 2.94444145190827 5.64410367534435 5.71099676368607 3.45646280810038 2.08172369443756 4.77378817888799 NA 3.39669616122062 NA 3.96038658650942 6.20343667061349 NA 1.4254440462816 NA 3.86908719027416 -0.925784871866254 3.68228511847456 -2.00700667990207 0.0466275458907165 0.338751929784362 -4.54182697655948 None

hsa-mir-504 2.52265962793069 NA NA NA NA NA NA NA NA NA NA NA NA NA NA NA NA NA NA NA NA 5.05271813001471 NA NA NA NA NA NA NA NA NA NA NA NA NA NA NA NA NA NA -2.53005850208402 3.7876888789727 -1.37663298286447 0.17138453235755 0.669812834583758 -4.55173664009426 None

hsa-mir-630 3.93920114465371 4.17462318746692 11.0690014132963 5.06919226738872 6.68398070988174 3.84547665491703 5.64384635950675 6.36149771624555 6.94931237339624 8.97766964317528 5.06401461603744 1.29498896445166 5.73601507923095 4.10122724858142 6.07710477972054 5.29620058095447 6.40811550808072 0.528682178873137 5.7588682188996 2.29263957745952 2.55525572532911 7.95499879385977 8.67850102056087 1.748903768789 6.71274814397805 9.96377759360761 7.56186347176459 4.42327383187784 2.40620772832812 7.96425942253985 4.77956596269746 11.201417422782 5.72771029120979 NA 7.36864661388977 6.84067674739935 5.23286794388438 4.61702873159414 8.76474942140813 NA -1.097664457528 5.78352923309783 -2.01246640299822 0.0459913188094201 0.338204454171711 -4.58100620733757 DOWN

hsa-mir-604 NA NA NA NA NA NA NA NA NA NA NA NA NA NA NA 3.56567414139276 NA NA NA NA NA NA NA NA NA NA NA NA NA 1.11247705139711 NA NA NA NA NA NA NA NA NA NA 2.45319708999565 2.33907559639494 1.3348118333127 0.184657846501271 0.699206619743943 -4.5921448934324 None

hsa-mir-223 8.60588547952171 11.414674456969 11.0221940973639 12.0090514023819 10.7013852765201 10.7316295040669 12.159288522027 11.2189442949432 10.5537087877764 12.615231880022 11.1299746269343 10.7202013499234 11.7810874420734 11.869790477862 8.89881390796386 12.7075646024864 10.7531884390849 14.1499095745563 8.73135509379627 10.0731150372832 9.76375530727785 12.1643969166695 10.9303452504062 9.88219732505381 10.7828202901361 11.9530339153532 11.962297818106 10.7602653208358 11.2743726517551 9.59642264560717 9.65816197135991 9.99686770466737 8.28181080901978 9.98196660438114 12.5293728830419 7.87532677627954 9.38257123967179 11.3667792676534 8.46141065348037 8.41289396263339 0.84149624700834 10.6716015891736 2.01657691790217 0.0455305693304737 0.338204454171711 -4.59726975238607 None

hsa-mir-658 0.197549406291212 NA NA NA NA NA NA NA NA NA NA NA NA NA NA 0.428447415372419 NA NA NA NA NA

2.49031126470766 NA NA NA NA NA NA NA NA NA NA NA NA NA NA NA NA NA NA -2.17731285387584 1.03876936212376 -1.37399897495376 0.172174111708926 0.669812834583758 -4.61080949842899 None
hsa-mir-1200 NA NA NA NA NA NA 4.62253895462322 3.3907046848773 NA NA NA NA NA NA NA 3.0039642572087 NA NA NA NA NA 0.731188551325273 NA NA NA NA NA NA 3.77158127992256 NA NA NA NA NA NA NA NA 1.59625891913448 NA NA 1.63939304877564 2.85270610784859 1.54667023531457 0.124679172134921 0.58429337716315 -4.62384046568913 None
hsa-mir-128 1.81117874315163 1.5734490517169 3.72498808763011 4.02588333640245 NA NA 2.18934407165051 3.08196550529742 2.73120823723147 4.62639567284719 2.79294888899603 2.98301162591101 NA 3.86191455485745 NA 0.023314895696386 2.47718438382498 1.70994604326112 NA 0.57827950824443 2.29942621081147 3.67918882333109 3.82696863646154 2.57455875062278 NA 3.29652966560267 4.87443111306931 2.20042463291173 2.18195098291385 2.67149074643682 3.24269163338262 5.50406264189913 2.41095524057469 NA 5.66311374211509 NA NA 4.35389987739671 2.99287213364786 NA -0.905436814963884 2.99878591459655 -1.92923878440088 0.0557319633249568 0.366418493848134 -4.63463866972567 None
hsa-mir-487a 2.55396888019332 NA 2.59015391542373 3.50864126402355 NA NA 3.91976501289869 2.72868427808906 NA 2.99644650576146 3.20908284761555 1.78607113703632 3.31169224472936 3.7708145588966 NA 1.70474801273696 3.78001069649759 2.39336848697009 NA 2.70269240421127 3.40421107932652 2.64035062321998 4.62664107075902 4.26519178677718 5.89642429917187 NA 3.19193903857039 4.12738650651269 3.32577020714468 5.28283514220244 NA 3.4742409556797 2.6964503086854 NA 4.64222411844962 1.32337824183612 NA NA 4.65288050291712 NA -0.899555974012084 3.37521657594058 -1.91085544747427 0.0581050401314446 0.366418493848134 -4.63814310698252 None
hsa-mir-194 8.32477911177557 7.09529878838935 9.78829412024677 6.05866506601591 5.28928901358688 7.86473781034414 5.08726521759042 9.79298203759279 8.95032359662749 3.64500319169954 7.61824061715954 6.61902885105404 9.92760734038767 7.13991714575397 5.55019042024145 8.03351753583159 1.97424910339138 4.86056189838131 9.07216537836518 8.70146186675942 7.64304602251455 7.20191873464628 7.3891535057047 7.50326085683286 9.2142352412491 8.29028020640895 9.13715906865598 9.41832567505647 8.83795788070862 6.99444010811331 7.83472087721051 6.1875464457043 12.6863114767605 7.24871883665944 8.36124961654062 5.22958373947175 6.25764911286082 7.24920263626105 7.72914420905624 9.50549993812848 -0.926291303867507 7.53282455749347 -1.98453707594793 0.0490266311707626 0.345411533246168 -4.6588909935569 None
hsa-mir-345 2.35597403755198 NA 1.62488691493231 2.35453702637577 NA 2.11496249994191 4.54068316057135 3.21970309011893 2.76779670393137 3.4585477862893 2.2760290285435 5.13663434681721 3.94562048461787 2.05113019253559 NA 3.28483931998617 4.13580451729303 NA NA

4.35857445892286 1.88494069516919 4.00466574614303 NA 2.78412458749357 NA NA 5.00167952964068 NA 3.39943445330735 NA
4.79582037494059 3.09102872150207 3.2860111440419 4.92944561287919 4.04922643286498 NA 6.51799197395308 3.2031908394193
4.4787902873055 5.62489479180623 -0.900040704280913 3.60955064685848 -1.90422776378868 0.0589548522268041 0.366492534976937
-4.66504281650324 None
hsa-mir-1321 NA NA NA NA NA NA NA NA NA NA NA NA NA NA NA 0.193294253488016 NA NA NA NA NA NA NA NA NA NA
NA 3.29981414260273 NA 1.28600773435796 NA NA NA NA NA NA NA NA NA NA -2.09961668499233 1.59303871014957
-1.31798349077021 0.190187297769589 0.703576322423696 -4.66864134330079 None
hsa-mir-568 0.907373047017185 6.11289449207886 NA NA NA NA NA NA NA NA NA NA NA NA NA 6.05157522879522 NA NA NA
2.47656534688992 NA 0.63728609243329 NA NA NA NA NA NA NA NA 4.56873600782829 NA NA NA NA NA NA 1.65177986103133 NA
NA 1.60116804159766 3.20088715372487 1.53553281789001 0.127365133899103 0.58429337716315 -4.6831858318805 None
hsa-mir-663 0.801425269553601 NA NA NA NA NA NA NA NA NA NA NA 3.3237142419136 NA NA NA NA NA NA NA NA NA NA
NA 5.70802844859383 NA NA NA NA NA 3.84609179005995 NA NA NA NA NA 2.85982940880108 2.85459405075936 3.10410039516786 NA
-1.61195906294282 3.21396908640704 -1.48042831449332 0.141459022104843 0.627204340656032 -4.69546459384886 None
hsa-mir-483-3p 0.800128777922285 NA NA NA NA NA NA 4.41455949045437 NA NA NA NA NA NA NA 0.855240061982592 NA NA
NA NA NA 0.0335871731595792 NA NA NA NA NA NA NA NA NA NA NA NA NA NA NA NA NA NA 1.9897222702935
1.52587887587971 1.30831096190962 0.193411832069827 0.706832331746094 -4.70755871393923 None
hsa-mir-572 NA 4.53996465100517 NA 3.96997928617878 NA NA NA NA 5.49399717474664 NA NA 1.88258873421863 3.18569243344045 NA NA
6.04650079707955 NA NA NA 2.68945661868242 0.81155131869874 0.562855317856713 NA NA NA NA 2.45658429190713 NA NA NA NA
NA NA 5.96030982677641 NA NA NA 3.18004211819596 3.34473020588324 NA 1.25325158611625 3.39417329035922 1.67579077128999
0.0963304107545551 0.488128047773082 -4.71277240050198 None
hsa-mir-222 8.87902001911354 11.8790980384268 10.4616808128497 8.75226883453316 8.84629084461097 10.335913275471 10.9005914015131
11.1020176175024 9.28258560000273 8.48701094664534 7.93711166821365 7.8310708940836 7.77240888859762 9.14075153416247 6.56101686011479
8.77090390545544 13.8458558680148 7.26277236007261 7.60381926582489 8.63230518035566 5.59905111927946 8.77366920561425 10.0511393226434
7.76093772604609 10.5398417498665 8.85624431730535 7.57323710872924 9.54959367992649 7.371001285948 9.55717525567588 4.76128355339528
7.77088720607128 8.31184136579837 7.23739306635019 8.10171717402329 8.25171217515534 9.54492720470535 9.95725824063049 8.14770345179851
9.46687013532377 0.855050473563892 8.78669945399627 1.95147691682831 0.0528699644315927 0.36228510752199 -4.7214937539917 None

hsa-mir-526b 3.57882632718144 NA NA 2.74614076083059 NA 3.12607346778978 3.03488680954607 3.55391020465608 5.05642681748355 NA NA NA NA 2.59515203784242 NA 1.1278336520023 NA NA NA 1.91760585980085 NA 2.53490755279901 NA NA NA NA 4.27177575998938 4.47946593925188 NA 2.17016354847238 4.65978313251431 NA NA NA 2.87284768580148 NA 5.79998043847874 4.95185120085129 4.51972870026359 NA -1.05818311347655 3.49985332753084 -1.74907025926947 0.0826858526351466 0.453268810354485 -4.73023896013227 None

hsa-mir-129-3p 5.17493373403686 5.73737737825382 NA 1.49630042110573 4.97914095473921 5.5598209818884 5.6083735832903 NA 3.63611244890433 NA 7.15116403793659 3.51819952465803 NA 4.25371806289019 NA 4.57057579858611 NA 5.40145253685519 NA NA 4.68206045119965 1.5791675625166 NA 5.02318299960067 NA 2.86408325179746 NA 3.46462131349875 3.42737483459733 NA 4.36394747540165 NA NA NA NA NA NA 5.11179180798447 3.09299208371317 NA 1.02290614633876 4.31887577349783 1.78033728891395 0.0773482644166025 0.427899114157902 -4.73434591538285 None

hsa-mir-1914 1.61111101460881 NA NA 3.67921156521786 NA NA NA NA NA NA NA NA NA NA NA NA NA NA NA NA NA NA NA NA NA NA NA NA NA NA NA NA NA NA NA NA 4.6287393802987 NA NA NA -1.98357809038536 3.30635398670846 -1.24478068273242 0.215799904191602 0.710148198577697 -4.74072103050936 None

hsa-mir-324-5p 3.85350016436157 3.89967531600997 2.65834044246467 4.28705286402932 3.80058569218243 5.82700478049632 4.85433783353674 3.85587880191605 5.58466683602353 3.28147816850443 4.9538662797702 5.8871253172826 3.2779819738577 5.40748040773347 NA 3.98882178826899 4.3687986656834 4.24962001327156 4.08820225298673 3.45063632551341 4.88929200048632 4.81022009231732 3.82844376716844 5.16521932128527 5.21389229810532 NA 5.10912837029889 5.0839043462066 4.40721274666096 6.62843875676607 NA NA 6.93733983616677 6.13233218510097 4.14660171316001 2.75956329701039 5.42560399146353 4.86151882530177 3.50688135979736 7.68014594527034 -0.799854865302098 4.67113313267943 -1.913991817068 0.0575849617015699 0.366418493848134 -4.74231792323917 None

hsa-mir-367 1.78404861372468 NA NA NA NA NA NA NA NA NA NA NA NA NA NA NA NA NA NA NA NA NA NA NA NA NA NA NA NA NA NA NA NA NA NA NA 3.06306848566852 4.40064276056598 NA -1.94780700939257 3.08258661998639 -1.22634555219211 0.222629984588161 0.710148198577697 -4.75824620138569 None

hsa-mir-2278 NA NA NA NA NA NA NA 4.86667802091673 NA NA NA NA NA NA NA NA NA NA NA NA NA NA NA NA NA NA NA NA NA NA 3.85429800974194 NA NA NA NA NA NA 2.01430383976927 NA NA 1.93237709616113 3.57842662347598 1.21407179554894 0.227263457616843 0.710148198577697 -4.76977381024342 None

hsa-mir-591 2.57631670165407 3.291407398673 NA 3.61367746510038 3.60769289118016 6.42077901746189 3.61647982496512 NA NA NA NA NA

NA 3.03862911800558 NA 3.06814915550856 NA NA NA 3.3395728768536 NA 0.558228511064629 NA NA NA NA NA NA NA NA NA
NA NA NA NA NA NA 3.24642648915795 3.07574167213592 NA 1.32572382581409 3.2877584268134 1.53996459950407 0.126170009647967
0.58429337716315 -4.7742933880264 None
hsa-mir-1285 2.24277382904919 NA NA 3.4817120706183 NA NA NA NA NA NA NA NA NA NA NA 1.65106507123788 3.1037957476469 NA
NA NA NA 5.60285707403191 NA NA NA NA 3.86187311188034 2.32799994960552 NA NA NA NA NA NA NA NA NA NA
4.19379093848139 NA -1.37679358886172 3.30823347406893 -1.50838111974124 0.134141870285305 0.599167020607697
-4.77449960655348 None
hsa-mir-329 1.83758091485005 NA NA NA NA NA NA 1.93571032549238 NA NA NA 2.29294818165601 NA 3.35600045709259 NA
3.28877813291149 NA NA NA NA NA NA NA NA NA NA NA NA NA 4.32729100266192 NA NA NA NA NA NA NA NA NA NA
-1.78508740026142 2.83971816911074 -1.26901165638252 0.206988935718297 0.710148198577697 -4.77634675683198 None
hsa-mir-556-3p NA NA NA NA NA NA NA NA NA 4.06947394833542 NA NA NA NA NA NA NA NA NA NA NA 2.14353597152743 NA
NA NA NA NA NA NA 2.18406856898795 NA NA NA NA NA NA NA NA NA NA 1.90567167807773 2.79902616295027 1.20266161443761
0.231633044401765 0.715938193056917 -4.78038962712301 None
hsa-mir-520b 0.345467772876064 NA NA 2.3542031344281 NA NA NA NA NA NA NA 2.89809880726504 NA NA NA NA 3.47286368884313
NA NA 3.73648569833657 NA NA NA NA NA NA NA NA NA NA NA NA NA NA NA NA NA 0.272832617541227 1.79117301642077
NA 1.52942100336878 2.12444639081584 1.40668036960202 0.162184879490239 0.657032302917723 -4.78818609040339 None
hsa-mir-338-5p 0.171539226002595 NA NA NA NA NA NA NA NA NA NA NA NA NA NA NA NA NA NA NA NA NA NA NA NA
NA NA NA NA 2.20451566855649 NA NA NA NA NA NA NA NA NA NA -2.03297644255389 1.18802744727954 -1.10616510325785
0.27103664015056 0.742886790958125 -4.79178832991031 None
hsa-mir-1228 1.79603460495994 3.72701994690294 NA NA NA NA NA NA 3.00012260071996 NA NA NA NA NA NA NA NA NA NA NA NA
NA NA NA NA NA NA 4.66485723324291 NA NA NA NA NA NA NA NA NA NA NA NA -1.82379818238196 3.29700859645644
-1.22008004335611 0.224964209271294 0.710148198577697 -4.79553704013826 None
hsa-mir-23b 10.7597120475754 8.12973363392422 7.628637688472 6.48810292495594 9.26756768565497 8.81216299073563 9.46310165119962
10.7009748354574 8.71140966333711 8.10566858976919 6.95815310438786 11.2078767878884 7.5330588472323 6.58675675492475 8.9761877503397
8.27990444684852 6.53269313092809 9.1109166438803 7.11136138936872 10.6059641024333 10.584799931061 10.0678114261269 8.63942260726077
8.49303375905208 9.97097354108362 8.54708954145888 8.70561734655277 8.24185347422109 9.27612114454323 8.44738237742235 10.4063837082116

7.95386251115803 5.66396484936335 11.6167833132665 10.4486345106251 9.36532815298976 9.04685040634645 11.4056120084971 10.7589057899315 9.45072736964344 -0.806060654975104 8.95152756095322 -1.9100686534752 0.0580396513482742 0.366418493848134 -4.79849521769329 None
hsa-mir-611 NA NA NA NA NA NA NA NA NA NA NA NA NA NA NA 2.46945027742959 NA NA NA NA NA NA NA NA NA NA NA 4.4843528323791 NA NA NA NA NA NA NA NA NA NA NA NA -2.01490255494951 3.47690155490435 -1.09633089990473 0.275296874345877 0.745003004575917 -4.79956169414993 None
hsa-mir-589 NA NA NA NA NA NA NA NA NA NA NA NA NA NA NA 4.69957789135271 NA NA NA NA 2.32561180963103 2.8377072123282 NA NA NA NA NA 3.00901933320599 NA 2.17429452542311 NA NA NA 3.83365463268633 NA NA NA 3.63113209975803 NA NA 1.73100795584726 3.21585678634077 1.25307180272422 0.212689407427874 0.710148198577697 -4.80063883334705 None
hsa-mir-943 1.86094647425359 2.53015850087186 NA NA NA NA NA NA NA 4.14661549921505 NA NA NA NA NA 1.37490384324212 NA NA NA NA NA 3.49595633734756 NA NA NA 4.06115515282606 NA NA NA 2.8694123302578 3.27857442926842 NA NA NA 5.22864325001669 NA NA NA NA NA -1.30859222054765 3.20515175747768 -1.51668475558207 0.132010150835729 0.594045678760781 -4.80204310371759 None
hsa-mir-518b NA NA 3.07928105954587 2.30648741045551 NA NA NA NA NA NA NA NA NA NA NA 2.83605770698691 NA NA NA NA NA 2.84023155991976 NA NA NA NA NA 5.11333274237984 NA NA NA NA NA NA NA NA NA 4.66148852136416 NA NA -1.46440888222516 3.47281316677534 -1.3928254325503 0.166348507182858 0.659922038363576 -4.82174600051627 None
hsa-mir-1539 0.183217641946531 2.20602888528577 NA NA NA NA NA 3.03838699434016 NA NA NA 2.61384179957105 NA NA NA NA NA NA NA NA NA 3.27867499043829 NA 4.27835400197354 NA NA 2.50234887116207 3.13969577163315 2.62179540839059 2.02986316632545 NA NA 4.51888308311497 NA 3.05377981690196 NA NA 2.95425477110382 3.48895713447835 NA -1.17629187126634 2.85057730976184 -1.56883413726282 0.119245867886815 0.566183136501965 -4.82493201347127 None
hsa-mir-486-3p 2.23446981773395 NA NA NA NA NA NA 2.32352248080959 NA NA NA NA 2.52192346210247 NA NA NA NA NA NA 3.72726709144408 NA 3.51998226226497 NA NA NA NA NA NA NA 3.66782774457795 NA NA NA 5.05700537908784 NA NA NA NA NA NA -1.37980941562106 3.29314260543155 -1.4102320862348 0.161136204864209 0.657032302917723 -4.84709792563149 None
hsa-mir-514 3.46667280495859 5.18509201720061 NA 1.40278909627854 NA 1.4017038038854 4.61476035058878 NA NA 2.52601414244162 1.29236781722663 1.79138046622993 NA NA NA 4.05359861089001 NA NA NA 1.90871004932742 NA 2.69729613345296 NA NA NA 5.10355024475135 NA 1.97146715894726 NA 4.69933405401767 3.49576978044331 NA NA 3.30727678566782 NA 4.27207957400889 NA

3.06197891152235 5.32020186451941 NA -1.00557491824514 3.24063387717677 -1.68092132641531 0.095207906561882 0.486528539464533 -4.86229553248166 None
hsa-mir-522 NA NA NA 3.5996025234135 NA NA NA NA NA 4.14189289124652 NA NA NA NA NA 2.22129461677936 NA NA NA NA NA 1.60708143885147 NA NA NA NA NA NA NA NA NA NA NA NA NA NA NA NA NA NA 1.71384857162832 2.89246786757271 1.14634980922564 0.254062111818661 0.731348500441128 -4.86449051012026 None
hsa-mir-302c NA 2.58288285978876 NA 1.97225912235766 NA NA NA NA NA NA NA NA NA NA NA 2.24646830429926 NA NA NA 3.79071385609094 NA 3.13529921661802 NA NA NA NA NA NA NA NA NA NA NA 5.09439299418546 NA NA NA NA NA NA -1.46676506976758 3.13700272555668 -1.31309329775201 0.191754994429476 0.705050375859597 -4.87546501248123 None
hsa-mir-186 8.3186158054997 8.64660176485336 7.75792862344446 7.12699354557955 10.4989384319136 10.0499402698777 4.88453271446096 7.65654504326993 6.30458255236903 3.7546399721622 8.23298156060633 6.75905044992552 7.33713975298491 6.99060389887945 7.41106811508797 7.31428226606626 5.12587836095778 6.90739598925954 8.8545274819336 7.50890416422856 5.42135851709671 9.66384465395187 5.11979326715957 6.51883975756307 7.62863208605538 6.75474525192781 8.53197845974956 6.23860346671571 6.1345000604824 6.76587583133208 5.58961599141186 6.5287068607982 7.30472974380809 6.10205492003224 5.49602632210874 5.21827207179996 7.97806813985226 6.68592735591811 6.06470172236525 6.0386040621653 0.782813611053312 6.98065073264136 1.86710838672138 0.063844978043174 0.38887395717206 -4.87672117146664 None
hsa-mir-586 NA NA NA NA NA NA NA 3.57677130106077 NA NA NA NA NA NA NA NA NA NA NA NA NA 1.4371263255517 NA NA NA NA NA NA NA NA NA NA NA NA NA NA NA 2.44001042454832 NA NA 1.63820292601076 2.4846360170536 1.03248732683772 0.304058967619057 0.756336745107844 -4.92714288492877 None
hsa-mir-548l NA NA NA 4.55198989119885 NA NA NA NA NA NA NA NA NA NA NA 2.44684868749712 3.68625746748478 NA NA NA NA 2.17598866474918 NA NA NA NA NA NA NA NA NA NA NA NA NA NA NA 2.05104313874453 NA NA 1.4481827803134 2.98242556993489 1.22972243180406 0.221322550116812 0.710148198577697 -4.93091274426469 None
hsa-mir-892a NA 3.51483594830973 NA NA NA NA NA NA NA 2.6981663785378 NA NA NA NA NA 2.18214377928239 NA NA NA NA NA 2.00420692436717 NA NA NA NA NA NA NA 1.62678650661079 NA NA NA NA 0.691188516491953 NA NA NA NA NA 1.35765471955334 2.11955467559997 1.29600690315784 0.197557580146056 0.710148198577697 -4.93605857102276 None
hsa-mir-2054 2.29997777336489 NA NA 1.1991091476988 NA NA NA NA NA NA NA 2.01391744092321 NA NA NA 2.96269761883376 NA NA NA NA NA 3.29035250993433 NA NA NA NA NA NA NA NA 1.93509254337269 NA NA NA 4.23311261579608 NA 5.95969116003628 1.38435128377406 NA NA -1.24159452737752 2.80870023263712 -1.4128274335383 0.160328264182521 0.657032302917723

-4.94046866176636 None
hsa-mir-765 NA NA NA NA NA NA NA NA NA 2.29503254306839 NA NA NA NA NA 2.26009078824436 2.51124429181956 NA NA NA NA
4.97983687919697 NA NA NA NA NA NA NA NA NA NA NA NA NA NA NA 2.59486637074937 NA NA -1.43189575059573
2.92821417461573 -1.21387357738238 0.227294531219727 0.710148198577697 -4.94749085825397 None
hsa-mir-764 2.51190974799568 NA NA NA NA NA NA NA NA 4.87174775754004 NA NA NA 2.92473954645211 NA 1.82343232442445
3.51498258915717 3.4829193614813 NA 0.944641287868967 NA 4.62033067030882 NA NA NA NA 4.85957403324097 NA NA
3.59421806971393 4.76985712639723 NA NA 4.35846030324587 2.76187789761639 NA NA 2.4780708493998 NA NA -1.05257376214333
3.39405439748877 -1.53271256779506 0.127905017886461 0.58429337716315 -4.95933165277105 None
hsa-mir-551b 2.86860331557981 NA 2.04606840059271 5.40261514836426 5.16636129960609 3.04332825379304 4.56450102790099 3.92397470570839
4.41371318392225 4.86332329271625 2.48120875524214 4.27841623750845 NA 4.45833190664757 NA 2.9483707398167 NA 5.18374452485499 NA
3.36162055915958 4.93689402806408 3.09206170498293 NA 3.84536495599443 NA NA 4.73318408956387 1.51274450397596 1.65384222821475
2.31427914869275 2.80002639736892 3.35924696297552 1.48980157118151 NA 2.61157899548141 NA NA 4.2617585376233 3.76709518228198 NA
0.827621451140258 3.54935927349338 1.72300133619629 0.0871368630337771 0.458268310175156 -4.96234474472778 None
hsa-mir-346 0.258254486882456 4.24303988593543 NA 1.85295348990689 NA 3.74818292164467 3.02600407447109 NA NA 4.54126113425673 NA
4.10359536568837 NA NA NA 3.00536390726202 NA NA NA 1.9384442676317 NA NA NA NA NA NA 4.64593866755503 NA NA
2.45264110134435 3.33580152200924 NA NA 5.09567230196328 1.9859262948555 NA NA 2.20292058438142 5.90171365182539 6.32238362120213
-1.02430810328878 3.45059395757739 -1.58163678423568 0.116230203617905 0.560694502252773 -4.96779958933107 None
hsa-mir-1976 NA NA NA NA NA NA NA NA NA NA NA NA NA NA NA 0.116057727610293 NA NA NA NA NA NA NA NA NA NA
NA NA NA 1.14503833796984 NA NA NA NA NA NA NA NA 2.15472355864976 NA -1.53382322069951 1.13860654140996
-0.966683860441041 0.335776178531102 0.772857509371917 -4.97803913066074 None
hsa-mir-145 9.94191585277053 8.19504619559765 15.7536001416495 11.9150435907759 12.2893990378274 14.2272855170148 14.619739516486
13.4880794052871 9.52838792125985 8.9609224933427 11.6332313107235 12.3757813479781 12.8627967432734 13.0363592266353 8.26518350118002
12.8225036305369 13.5015612054083 9.47098069668668 13.7238621863594 13.6217214038947 10.303670000818 13.1102301376752 12.1657875312223
12.4274116235597 10.0597950225495 8.13456528131796 8.96841993374411 10.427006462627 15.5836290628905 12.0900665605489 6.69919500195124
9.7761743544461 11.5803799477774 9.9487868593902 13.4196948902878 13.5405032308487 11.5080217219198 12.2068779273801 10.7373191719299
9.8751106124195 0.883537779469192 11.5699011564998 1.79521421396372 0.0746400416141497 0.420635000872264 -5.00383209970395

None
hsa-mir-508-3p 2.46414929488383 NA NA 2.64318004501571 NA NA NA NA NA NA NA NA NA NA NA 3.10408586275242 NA NA NA
1.88081666438734 NA 2.91571205898773 NA NA NA NA NA 4.98944741340786 NA 4.43199340831454 NA NA NA NA NA NA NA
0.702816390247046 3.78151422110482 5.28891187602245 -1.16200792792092 3.22026272351237 -1.37981087002837 0.170221777782429
0.669812834583758 -5.01274435441444 None
hsa-mir-548a-3p 2.40020641098245 3.51426870467594 3.68369596247328 3.81422966804466 NA 4.95296991409432 5.00444438840654 1.22931071142426
4.6790743611626 3.64835465280121 3.60496408688806 2.16511157758866 4.1186505111274 2.22778567983307 4.47342847502096 3.56980686458377
5.42517768804288 NA 4.10477873776384 3.79960471095412 1.65286322916632 4.6610889455647 NA 4.04518543579882 NA 4.31856824753638
6.99984237114139 NA NA 1.89965560942035 4.71948235980351 3.56571057821244 NA 5.06249894907915 3.1769848745458 NA 6.47619721981472
5.66698867771308 6.18034843829128 4.46920995305675 -0.802703033819253 4.04095274984415 -1.72490644409711 0.0867282317081414
0.458268310175156 -5.01436890153371 None
hsa-mir-938 1.85768889289811 3.56398539343512 NA 0.880994604924969 NA NA NA NA NA NA NA NA NA NA NA NA NA NA NA NA
NA NA NA NA NA 3.51184917049843 NA NA NA NA NA NA NA NA NA NA NA NA NA NA -1.41095954007903 2.45362951543916
-0.939499544792707 0.34946996176629 0.79183870225991 -5.03554731242199 None
hsa-mir-548q 3.5094411583044 1.72061293601862 NA NA NA NA NA NA NA NA NA 1.66717311759489 NA NA NA NA NA NA NA NA NA
NA NA NA NA NA NA 2.07369688927149 NA NA NA NA NA NA NA NA NA 5.18263901460966 NA NA -1.32909221463461
2.83071262315981 -1.11468528128126 0.267320157450099 0.742886790958125 -5.04649007976212 None
hsa-mir-331-5p 0.894840239652293 NA NA 1.92892962659952 NA NA NA NA 4.10212318847508 NA 2.95657998399487 NA NA NA NA
2.22849988939064 2.81835137833104 NA NA 0.712266558381637 NA 2.93685553640537 NA NA 4.20968378200239 NA NA NA NA NA NA
NA NA NA 2.84149191800955 NA NA 3.34452110511121 NA NA -1.09862510469283 2.63401301875942 -1.36728923799629
0.174080364295659 0.670878793960294 -5.05208947751992 None
hsa-mir-548f 2.90607006270854 2.82409560318029 NA 3.44210298437512 NA NA 2.28247064988938 NA 3.2264575888449 3.56562655195058 NA
2.98023336330995 NA 2.43719107848186 NA 3.2730677862897 3.10126694050892 NA NA 2.7688322858737 NA 3.93527318614789 NA NA NA
NA 4.15489724937304 3.41277302377335 NA NA NA NA NA NA NA NA NA 4.49138948356712 NA NA -1.01609097249599
3.25344985588496 -1.40689027166445 0.161951943816027 0.657032302917723 -5.05897149658899 None
hsa-mir-520a-5p 3.95141607020234 NA NA 2.22590545220612 NA NA NA 2.45094437561234 NA NA NA NA NA NA NA 1.30349608408392 NA

NA NA NA NA NA NA NA NA NA NA 3.515505693079 NA NA NA NA NA 4.60734458403226 NA NA NA 2.92647013269095
3.50905618968015 NA -1.15665365434441 3.06126732269838 -1.27499913534064 0.204825546932644 0.710148198577697
-5.06790002969715 None
hsa-mir-1293 2.48886734000277 NA NA NA NA NA NA NA NA NA NA NA NA NA NA NA NA NA 3.11820048232486 NA
1.49456573409472 NA NA NA NA NA NA NA NA NA NA NA NA NA NA NA NA NA NA 1.30896817706909 2.36721118547412
0.825652133322893 0.410749374137668 0.834709667241015 -5.07603909845835 None
hsa-mir-1307 NA NA NA 2.30826096185959 NA NA NA 3.36430457670705 NA NA NA NA NA 1.67421942256556 NA 2.13480506802509 NA NA
NA NA NA 1.39682896776336 NA NA NA 5.90016895863469 NA 2.09702141540007 NA 1.30440400177515 NA 6.03104194071347 NA NA NA
NA NA 4.37382769692829 NA NA -1.14681798957985 3.05848830103723 -1.32767881115243 0.186815501908169 0.699206619743943
-5.07753755670646 None
hsa-mir-1287 1.80286509673849 2.6886761877494 NA 3.3254120828957 NA NA NA 3.79795930000135 NA NA 3.89168539866167 2.38655425242593
NA NA NA 1.10695615304265 NA NA NA 0.886003418472873 NA 1.67809689420926 NA NA NA 5.43953615278548 NA 2.27900775644914
NA 2.86446005037254 NA NA NA NA 5.4233851176459 NA NA NA NA NA -1.05113320804396 2.89004598934234 -1.40703107316693
0.161950895361515 0.657032302917723 -5.07853935590663 None
hsa-mir-573 2.76334881886663 NA NA 4.75044932744334 NA 3.60676602909668 NA NA NA NA NA NA NA NA NA NA NA NA NA NA NA
1.85910990851923 NA NA NA NA NA 4.3894109968511 NA 2.1227507768322 NA NA NA NA NA NA NA 1.82923280274664
2.51779214893941 NA 1.16319539835783 2.9798576011619 1.23683113968946 0.218616755149518 0.710148198577697 -5.08611002326331
None
hsa-mir-1910 4.51434025026574 NA NA NA NA NA NA 2.94937361780734 NA NA NA NA NA NA NA 2.83170812991715 3.40471616796222 NA
NA NA NA 5.51268357248867 NA NA NA NA NA NA NA NA NA 5.19839044224609 NA NA NA NA NA NA 3.11820398822826 NA
-1.1847247928329 3.9327737384165 -1.20372478664579 0.231136762484276 0.715938193056917 -5.08782871361281 None
hsa-mir-548p 2.79154896158083 2.37795546824083 NA NA NA NA NA NA NA 6.47290230026669 NA NA NA NA NA NA NA NA NA NA NA
NA NA NA NA NA NA NA NA NA NA NA NA NA 4.53247573662728 NA 5.79203073848038 NA NA NA -1.28145099419105
4.3933826410392 -1.06408790430653 0.289528378143758 0.750504307264223 -5.09382196269078 None
hsa-mir-1288 1.41842426322076 NA NA NA NA NA NA NA NA NA NA NA NA NA NA NA 2.28885146170236 NA NA NA NA
0.842935514233285 NA NA NA NA NA 5.18803253655521 NA NA NA NA NA NA NA NA NA NA 3.34889996900175 NA

-1.27298481080186 2.61742874894267 -1.05989298977119 0.291424562491201 0.750504307264223 -5.09764970331748 None
hsa-mir-185 3.8492769715678 3.63736668176074 4.67277534978875 3.71877671009858 3.88822159841585 5.7591420247066 3.04232141378593 NA 5.83200952614754 3.87812990438328 3.07669970903899 5.44131984900815 1.77276689398966 3.53014793696059 NA 1.70602671684646 1.02214284770336 5.21467204926327 4.64803727694812 4.35588800683306 4.69004086237222 5.15165196134427 4.69001770172163 6.10338867289746 3.63227189917055 5.72509722138689 4.66816264505611 5.47401666106149 3.74250715813945 4.33015117766704 3.03387575462676 6.37208261669001 5.35364061409403 3.05037555632005 5.46640116605824 NA 3.94990084383743 3.77412298091469 2.97079339625891 NA -0.72959874568725 4.20067278769067 -1.7057457393095 0.0901896545951901 0.464823604452133 -5.10684854573602 None
hsa-mir-1205 0.485051298719718 NA NA NA NA NA NA NA NA NA NA NA NA NA NA NA NA NA NA NA NA 1.548387285573 NA NA NA NA NA NA NA NA NA NA NA NA NA NA NA NA NA NA -1.06333598685328 1.01671929214636 -0.578572942152608 0.564044535875673 0.895722046824318 -5.11236543059322 None
hsa-mir-485-5p 0.397794146764182 NA NA NA NA NA NA NA NA NA NA NA NA NA NA 2.61499074239792 NA NA NA NA NA 2.51695929242568 NA NA NA NA NA 3.13298567176801 NA 2.47682421636637 NA NA NA NA 2.01934593160788 NA NA 3.32864215625622 NA NA -1.18855900910378 2.35536316536947 -1.10688803050847 0.270623215181574 0.742886790958125 -5.11784756829428 None
hsa-mir-139-5p 4.36709523718363 4.5867382197434 4.72821294919489 3.49996365617839 7.43757809069266 4.68933071166465 5.04031208202451 6.54029057080655 3.94552886557394 4.05710316458081 3.98475552763446 3.19798870377275 4.92891784571199 3.85926770216579 7.69649793573613 4.10122864300154 6.72488144768692 5.02698615526184 5.19616412590221 3.37845669341075 6.70976391741662 6.333161264687 5.34565880575189 6.18680977376982 2.35516157885496 6.06223173413254 4.22066498992827 6.40769637282966 5.6885864196273 5.48719401327213 6.37827102500885 5.77899062429108 6.28469374731967 4.62657536406503 7.60553406579536 4.29589664584023 5.62938550781463 1.45272587163584 8.6791146896792 6.15801408957393 -0.73494160866831 5.21683572073055 -1.72703438907604 0.0862273189930037 0.458268310175156 -5.11996045055758 None
hsa-mir-219-2-3p 2.62072288617975 NA NA NA NA NA NA NA NA NA NA NA NA NA NA NA NA NA NA NA NA NA NA NA NA NA 3.34793389609584 NA NA NA NA NA NA 4.24769219975554 NA NA NA NA NA -1.17709016174594 3.40544966067704 -0.742061073996362 0.459597206800001 0.857357456751318 -5.12695091644915 None
hsa-mir-1254 1.9630452028824 NA NA NA NA NA 3.49258035076478 NA 3.57684185617712 6.72720295445865 NA 1.5623016707554 NA NA NA NA 4.14266695552252 NA NA 3.44665297288845 NA NA NA NA NA NA NA 3.32881084933223 NA NA NA NA NA NA NA NA NA 2.06949520152051 1.90450222872435 NA 1.12448656825278 3.22141002430264 1.23975943371044 0.217494047968346 0.710148198577697

-5.12739826756963 None
hsa-mir-653 NA NA NA NA NA NA NA NA 2.33478734702032 NA NA NA NA NA NA NA NA NA NA NA NA NA NA NA NA NA NA
4.28588639927415 NA NA NA NA NA NA 2.73535540174595 NA NA NA NA NA -1.17583355348973 3.11867638268014
-0.739707527115294 0.461018671404106 0.857357456751318 -5.12830676767058 None
hsa-mir-924 NA 2.88222146251643 NA NA NA NA NA NA NA NA NA NA NA NA NA 4.06782873574571 NA NA NA NA NA
3.72704428971052 NA NA NA NA NA NA NA NA NA NA NA NA NA NA NA 0.758624619255713 2.29309078801557 NA
1.21543853347047 2.74576197904879 1.02441793070288 0.307797903051905 0.756336745107844 -5.12942809047475 None
hsa-mir-200b 10.8172889710703 11.0299659705961 11.335262877546 7.55922066769243 10.7698925955054 7.67857028622164 8.00818936639195
8.06476391921047 11.3956665202612 11.8129741819544 11.2666045785781 12.3253476574495 10.4256363563494 13.4311259317987 12.2319946605968
8.46631488277886 9.90283111029394 10.1004503975685 9.00712253209932 12.2175582808221 10.2731348143384 9.58105120664369 9.22859230439026
8.00392041308128 9.8990146228073 12.5223189920104 9.40383744580532 10.7912171269973 7.65702965601609 7.94121641154732 11.3638888975091
8.60419126619838 8.41270269746989 9.97187509570324 11.8967889534252 10.3060044463186 9.57701060921829 8.54307911076201 9.69288333518612
9.33879126298837 0.741911653818425 10.02138326033 1.71993922971214 0.0875132404390045 0.458268310175156 -5.13179758100878
None
hsa-mir-548d-5p NA NA NA NA NA NA NA NA NA 2.80502430738725 NA NA NA NA NA NA NA NA NA NA NA NA NA NA NA NA
NA NA NA NA NA NA NA NA NA NA NA 1.87489387076534 NA NA 0.93013043662191 2.3399590890763 0.506094320097793
0.613789881618678 0.898995297558389 -5.14087596363818 None
hsa-mir-553 1.7846923915145 NA NA NA NA NA NA NA NA NA NA NA NA 4.52457830006522 NA NA NA NA NA NA NA NA NA NA
NA NA NA NA NA NA NA NA NA NA NA NA NA 2.02284126288029 NA NA 1.13179408290957 2.77737065148667 0.707256350156311
0.48087007240122 0.862756303026751 -5.14656739661131 None
hsa-mir-409-3p 1.79533927340759 NA NA 4.76285028525148 NA NA NA NA NA NA NA 3.4785966075224 NA NA NA 2.56430333804349 NA
NA NA 2.85994162476796 NA 2.25815738089696 NA NA NA NA NA 6.40141267637474 NA NA NA NA NA 5.13502603177928 NA NA NA
2.61444252911404 4.37049722567955 NA -1.06370094297033 3.62405669728375 -1.28319011954403 0.20190907456415 0.710148198577697
-5.14754346334821 None
hsa-mir-450b-3p NA NA NA NA NA NA NA NA NA NA NA NA NA NA NA 3.61181185041456 NA NA NA NA NA NA NA NA NA NA
NA NA NA NA NA NA NA NA NA NA NA 2.72739546463549 NA NA 0.88441638577907 3.16960365752503 0.481220796375413

0.631302866210405 0.898995297558389 -5.14978741476881 None
hsa-mir-593 2.06435463410005 NA NA NA NA NA NA NA NA NA NA NA NA NA NA NA NA NA NA NA NA NA NA NA NA NA
NA NA 2.6220008379636 NA NA NA NA NA NA NA 3.7304212440408 NA NA -1.11185640690215 2.80559223870148
-0.700547535466976 0.485032150457825 0.862756303026751 -5.1502414746731 None
hsa-mir-187 NA NA NA NA NA NA 4.06848886259986 NA 5.05264422237988 NA NA NA NA NA NA NA NA NA NA NA NA
1.66123160420267 NA NA NA NA NA NA 4.87011750271581 3.59265150314349 NA NA NA NA NA NA NA NA NA NA 1.18589967246921
3.84902673900834 0.997990411786406 0.320388978149344 0.76664505485736 -5.15241308291198 None
hsa-mir-323-3p NA 3.35244380515301 NA 2.53068170854581 NA NA NA NA NA NA NA NA NA NA NA NA NA NA NA NA NA
1.85341678867476 NA NA NA NA NA NA NA NA NA NA NA NA NA NA NA NA NA 1.08814596817465 2.57884743412453
0.686110971198603 0.494055111730828 0.863522412677361 -5.15803014283547 None
hsa-mir-146b-3p 2.74292497511356 NA NA NA NA NA NA NA NA NA NA NA NA NA NA NA NA NA NA NA NA NA NA NA NA
NA 4.05575927179812 NA NA NA NA NA NA NA NA 3.85733673202997 4.98785394356381 2.63475107558177 NA -1.14100028062986
3.65572519961745 -0.789921044398657 0.431207167082805 0.846625918812306 -5.16107688549645 None
hsa-mir-211 NA NA NA 4.12348256851166 NA NA NA NA NA NA NA 4.78656371928546 NA NA NA 2.00106648840944 NA NA NA NA NA
NA NA NA NA NA NA NA NA 3.18193312261663 2.87421935849315 NA NA NA NA NA NA 1.44203304989068 NA NA 1.1376424150687
3.0682163845345 1.07434284658972 0.284908925316021 0.746956878111133 -5.16764106335579 None
hsa-mir-433 3.58123432935155 NA NA NA NA NA NA 5.63560655103115 3.38051235482498 3.83003052097524 3.38818787132306 2.89698891978561
NA NA NA 2.35621142320225 NA 3.47240114441578 NA 3.94778077810273 NA 1.84704403285681 NA 1.32056398135569 NA NA NA
3.20036169346576 NA 4.2708542433266 NA NA 0.149581079763167 5.63958154369719 3.22869296229475 NA NA 1.90666560574152 NA NA
0.914465623077576 3.17954700208905 1.43961805839484 0.152446132761443 0.657032302917723 -5.17076725014081 None
hsa-mir-1269 NA NA NA NA NA NA NA NA 4.26580203137572 NA NA NA NA NA NA NA NA NA NA NA NA NA NA NA NA NA NA
NA NA NA NA NA NA NA NA NA 4.94898853720863 NA NA NA -0.683186505832911 4.60739528429218 -0.371729379618228
0.710800022521549 0.918721477043092 -5.18369933845769 None
hsa-mir-219-1-3p 3.41173890268316 NA NA NA NA NA NA NA NA NA NA NA NA NA NA NA NA NA NA NA NA 2.81941299305597 NA
NA NA NA NA NA NA NA NA NA NA NA NA NA NA NA NA NA 0.59232590962719 3.11557594786956 0.322291118219728
0.747835830170649 0.923995650122037 -5.19616748431102 None

hsa-mir-581 2.91486863177383 NA NA 2.31925003964241 NA NA 3.00837778115569 NA NA NA NA NA NA NA NA 3.18639490216656 NA NA
NA 4.56632825009463 NA NA NA NA NA NA NA 3.56652371520185 NA 1.62027733836517 NA 4.6125098443537 NA NA 5.66588684615978
NA NA 5.63432705713093 NA NA -1.02086103927566 3.70947444060445 -1.23996147278118 0.217419573177753 0.710148198577697
-5.19787204673027 None
hsa-mir-770-5p NA NA NA NA NA NA NA NA NA 4.9035531562043 2.98428924922637 3.13413461237531 3.97038934114134 3.65812963831539
NA 4.46903721674648 3.15917896328692 3.63977936693523 NA NA NA 3.6713896463393 NA NA NA NA 4.10500738324852 NA NA
3.9560819866088 3.87539667626859 NA NA 6.27203672929597 5.47273658282782 NA NA NA 4.48216584318604 5.09573123069178
-0.876506816779436 4.17806485141864 -1.39777186384829 0.164647953167904 0.657501428875801 -5.20216936232239 None
hsa-mir-513b NA NA 3.23921703871453 NA NA NA 3.03853473332789 NA NA NA NA NA NA NA NA NA NA NA NA NA NA NA NA NA
NA NA NA NA NA NA NA NA NA NA NA NA NA 2.21456662761931 NA NA 0.924309258401899 2.83077279988724 0.583297876447002
0.560860624929269 0.895722046824318 -5.20885013271347 None
hsa-mir-296-3p NA NA NA NA NA NA NA 3.68725004717121 NA NA NA NA NA NA NA NA NA NA NA NA NA NA NA NA NA NA
NA NA NA NA 4.07209692029222 NA NA NA NA NA 5.15153938028744 NA NA NA -0.92456810311862 4.30362878258362
-0.582591657819835 0.561334406377424 0.895722046824318 -5.20917097440121 None
hsa-mir-1825 1.77532152665408 NA NA NA NA NA NA NA NA NA NA NA NA NA NA 2.8920537297131 NA NA NA NA NA
2.3608844719904 NA NA NA NA NA NA NA NA 4.48297961903331 NA NA NA NA NA NA NA NA NA -1.08824441732827
2.87780983684772 -0.83849261224711 0.403516965015879 0.834242612656822 -5.20933491031858 None
hsa-mir-671-3p 1.64611431259005 NA NA NA NA NA NA NA NA NA NA NA NA NA NA NA 4.53919006306422 NA NA NA
1.68285662860219 2.37533833015087 NA NA NA NA NA NA NA NA NA NA NA NA NA NA NA NA NA NA 1.06355470845061
2.56087483360183 0.816167524998959 0.416114406539044 0.835205686670525 -5.22484228019713 None
hsa-mir-518e 2.55811894140258 NA NA NA NA NA NA NA NA 2.88836036439578 NA NA NA NA NA 2.21794327371351 2.83474609644216 NA
NA NA NA 1.78827062813026 NA NA NA NA 4.49487021638675 NA NA 4.41604858142898 NA NA NA NA 3.79471895331157 NA NA
2.77562179783554 4.40921737527804 NA -0.988332423073349 3.21779162283252 -1.19949345749139 0.23270959508981 0.715938193056917
-5.22655170885455 None
hsa-mir-1470 1.87158976372863 NA NA NA NA NA NA NA NA NA NA NA NA NA NA NA NA NA NA NA NA NA NA NA NA NA
2.1330745805433 NA NA NA NA NA NA NA NA NA NA NA NA -0.26148481681467 2.00233217213597 -0.142276798362109

0.887118212709437 0.983331401220203 -5.22657747273991 None
hsa-mir-665 NA NA NA 2.96565311465269 NA NA NA 4.6846438239149 3.96327661964081 NA NA NA NA NA NA NA NA NA NA NA NA
NA NA NA NA NA NA NA NA NA NA NA NA NA 4.80996563305857 NA NA NA NA NA -0.938774446989103 4.10588479781674
-0.628700667680991 0.530808586769464 0.890713562955474 -5.22971953633836 None
hsa-mir-2116 1.02730620164242 NA NA NA NA NA NA NA NA NA NA NA NA NA NA NA NA NA NA NA NA NA NA NA NA NA NA
NA NA 0.990312454090996 NA NA NA NA NA NA NA NA NA NA 0.0369937475514239 1.00880932786671 0.0201287096709832
0.983976683644686 0.992203913441046 -5.23379527613608 None
hsa-mir-2277 2.32359887346426 NA NA NA NA NA NA 3.4089024291362 NA NA NA NA NA NA NA NA NA NA NA NA NA
0.981281238944097 NA NA NA NA NA NA NA 2.67973404096013 NA NA NA NA NA NA NA NA NA NA 1.03574301134812
2.34837914562617 0.799807841191254 0.425493345525863 0.841140264363592 -5.23594389126461 None
hsa-mir-631 2.89548905549896 NA NA NA NA NA NA NA 3.88497865683803 NA NA NA NA NA NA NA NA NA NA NA NA
1.38621689268265 NA NA NA NA NA NA NA NA NA NA NA NA NA NA NA NA 3.32698300657615 NA 1.03363390653909
2.87341690289895 0.797463880149075 0.426847298333763 0.841140264363592 -5.23751632491441 None
hsa-mir-491-3p 3.87750118225541 NA NA NA NA NA NA 3.40922732752779 NA NA NA NA NA NA 4.78628837440167 1.78420971917151
2.77296394920923 NA NA NA NA 2.3591057455371 NA NA NA NA NA NA NA NA NA NA NA NA 4.08966153607962 NA
5.94576384499282 4.9537919305719 NA NA -1.01104265378224 3.77539040108301 -1.15904087895059 0.24877038365769 0.731348500441128
-5.2387104042166 None
hsa-mir-1268 2.66073373034738 3.97274067642351 NA NA NA NA 1.79149461095062 NA NA NA NA NA NA NA NA 0.825749963031323 NA
NA NA 3.13940437853479 NA 1.04532995155977 NA NA NA NA NA NA NA NA NA 3.01057075870444 NA NA NA NA 6.57265817924385
NA NA NA -1.06482829131183 2.87733528109946 -1.0908725970606 0.277561447832984 0.745003004575917 -5.23948434650359 None
hsa-mir-548m NA 5.75714476931914 NA 2.23415707816397 NA NA NA NA 4.14218248597601 5.10946405690789 NA 0.893921997794814 NA NA
NA 3.63937305242604 2.48810596901894 NA NA 1.73288112312679 NA NA NA NA NA NA NA NA NA NA NA NA NA 4.2606399005521
NA NA NA NA NA NA -1.0109860839604 3.36198560369841 -0.718637407117763 0.473779337727036 0.862756303026751
-5.24324962773684 None
hsa-mir-1279 1.75184619840964 3.46445019004511 NA 3.59825609311847 NA NA 2.69437925986841 NA NA NA NA NA NA NA NA
2.40361247031398 NA NA NA NA NA 3.17928827176045 NA NA NA NA NA 3.83088654557189 NA NA NA NA NA NA 4.37073982874816

NA NA NA NA NA -1.01112937300905 3.16168235722951 -1.08492030484048 0.280178494966558 0.745003004575917
-5.24534036907349 None
hsa-mir-891a 2.31177764263015 4.59339367084383 2.40669519534678 4.30605083924642 NA 3.0182179929015 4.22333729994524 NA 6.26407217789661
4.17312558310268 2.69546728161481 2.13603310315651 NA NA NA 4.11098839881712 NA 2.70465597833962 NA 2.49192391214516 NA
1.83516400344103 4.78518886764045 NA 7.87451630909581 5.38474911777312 NA 5.12718155751785 NA 3.39894165342062 5.14211812846891
4.1895471089269 3.55859942309373 4.43629951626446 3.73715643342135 5.02568210691699 5.52602877465066 0.553474666714602
3.58103762090821 NA -0.781988833884689 3.91397944158004 -1.54258220435343 0.125231022517525 0.58429337716315 -5.24540818406212
None
hsa-mir-875-5p 2.33089616153551 NA 2.87485898004473 NA NA NA NA 0.177671769102836 NA NA 2.84765818889127 2.24553506639076 NA
NA NA NA NA NA NA NA NA 2.62357882743742 NA NA NA NA NA 2.30016320086035 NA NA NA NA NA NA NA NA NA
4.41591723988852 NA NA -1.01789572286908 2.47703492926892 -1.07963772866798 0.282515266635948 0.746826054308547
-5.25051125289922 None
hsa-mir-328 2.93675939217556 5.08446272746335 3.61391680386634 3.72706719574917 NA 4.27653882576837 2.96741526807953 NA 4.1139737860088
4.90296933816801 2.44267254662286 5.0085411683327 5.61937530728714 3.85165515802396 NA 3.67462015021052 3.35925331967343
7.26731115449034 1.68520530994551 4.07614163334473 3.77374374737201 4.63784418925063 6.28166939050599 5.80273507037003 6.36186948566399
2.78792254176973 3.36925061524496 4.17093410941333 5.9764593861233 7.16444191044183 3.19999633218869 5.9178079306392 2.01015575480536
4.20040725933954 4.38071301841896 4.00677407554827 6.51716073916801 5.12299619385133 3.71250796489356 5.3784901939493
-0.702936402200235 4.4157232160586 -1.62190430336668 0.106974452132502 0.537546621965823 -5.25133438138058 None
hsa-mir-1262 0.69039108304349 2.92598653593767 NA NA NA NA NA NA NA 2.81771257142794 NA NA NA NA NA 3.78961868805106
2.35467194200155 NA NA NA NA 0.0404112562220211 NA NA NA NA NA 5.08588479759385 NA NA 4.29787850692528 NA NA
3.00097025475278 NA NA 5.14095516522127 NA NA NA -0.997543832050699 3.01444808011769 -1.18526535391123 0.238264804677521
0.721791040320554 -5.25914571601503 None
hsa-mir-1236 1.91233824156646 2.08687047362221 NA NA NA NA 3.01270725098622 NA 4.3053504720092 NA NA NA NA NA NA
1.41658732341342 NA NA NA NA NA 1.81480775261086 NA NA NA NA NA 4.20869514011472 NA 2.9591476904112 NA NA NA
4.52655354885424 3.78440565040356 NA NA 3.66298049342583 NA NA -0.945994293650567 3.06276763976527 -1.21781381514749
0.225674607716888 0.710148198577697 -5.25935487311509 None

hsa-mir-1302 NA NA NA NA NA NA NA NA NA 3.69354258077296 NA NA NA NA NA 1.28978679797281 NA NA NA NA NA NA NA NA
NA NA NA NA NA 3.51521621298389 NA NA NA NA NA NA NA 3.45047660179665 NA NA -0.991181718017385 2.98725554838158
-0.76367395870191 0.446646536326901 0.849614704748016 -5.25967769415945 None
hsa-mir-339-5p NA NA NA NA NA NA NA NA NA NA NA NA NA NA NA 3.14488964721594 NA NA NA NA NA 1.71234405672282 NA
NA NA NA NA NA NA NA NA NA NA 2.95295157011809 3.97430952071818 NA NA 0.385846850743084 NA NA 0.888526647640397
2.43406832910362 0.608135064106816 0.544302627504899 0.891941408965673 -5.26379439843594 None
hsa-mir-1908 2.44128651759368 NA NA 3.42259937951706 NA NA NA NA 1.80798739576593 NA NA NA NA NA NA 4.25323947019089 NA NA
NA NA NA 0.130478023114067 NA NA NA NA NA NA NA 4.24245167351237 NA NA NA NA 1.03556056167463 NA NA
2.47799378700227 NA NA 1.00965717944106 2.47644960104636 1.09044468797891 0.27774902243233 0.745003004575917 -5.26605888833319
None
hsa-mir-1323 NA NA NA NA NA NA NA NA NA 3.71330450387404 NA NA NA NA NA NA NA NA NA NA NA NA NA NA NA NA NA
NA NA 3.52920584252531 NA NA NA NA 2.53442680007919 NA NA 2.65085177072099 NA NA 0.808476366098877 3.10694722929988
0.542726250731692 0.588382725115829 0.898214641126189 -5.26996720412739 None
hsa-mir-1258 1.54784092525233 NA NA NA NA NA NA NA NA NA NA NA NA NA NA NA NA NA NA NA NA 2.668790233488 NA NA
NA NA NA NA NA NA NA NA NA NA NA NA NA 1.78423127638456 NA NA -0.67866982968395 2.00028747837496
-0.427862349711614 0.669569496077045 0.911469750803578 -5.27014631295164 None
hsa-mir-448 2.50582913063973 NA NA NA NA NA 0.492011998810921 1.15582277277813 NA NA NA NA NA NA NA 4.12619020659104 NA
NA NA 0.482367216088346 NA 1.69815314977264 NA NA NA NA 4.12018020679344 NA NA NA NA NA NA 3.42727698908918 NA NA
NA 1.71550870520217 NA NA -0.987835497732724 2.19148226397396 -1.12625802296448 0.262332570754993 0.739612194734905
-5.27305539810483 None
hsa-mir-515-3p 0.763109109224327 4.5771607879855 NA 2.03527167849872 NA 3.46306744342893 NA 3.86919767269699 2.92372351643509
4.07898235413321 NA 3.50687715917844 2.83503746445032 NA NA 4.97831757642641 2.4959651076395 3.54765761549638 NA 4.37250515828294
NA 3.80928750758673 NA NA NA 3.28264550810277 5.49784531037126 3.29895572546951 NA NA 4.7938335752828 NA NA 4.45410950858648
2.95485197410611 NA 4.44475012061475 6.01509774103131 2.67942092570289 NA -0.781012663233403 3.68163784959702 -1.45749074956046
0.147347496428763 0.643844495264813 -5.27368635442296 None
hsa-mir-1973 NA NA NA 1.75148400337243 NA NA NA NA NA 2.6550684463486 NA NA 3.19051615159268 NA NA NA NA NA NA NA NA

2.06534657255297 NA NA NA NA NA NA NA 4.52752832104857 NA NA NA NA NA NA NA 2.99264686630751 4.54794281360801 NA
-1.00100994294136 3.10436188211868 -1.01608852005628 0.311693332983889 0.760935545705608 -5.27441153135743 None
hsa-mir-922 2.66770224752164 NA NA NA NA NA NA NA NA 2.10579828848669 NA NA NA NA NA NA NA NA NA NA NA
0.709784343507075 NA NA NA NA NA NA NA NA 4.41232794796237 NA NA 5.08022853292356 NA NA NA NA NA NA
-1.0140300067935 2.99516827208027 -0.841424095088567 0.401864736676603 0.834242612656822 -5.27647242349696 None
hsa-mir-767-5p 1.24602679981109 3.09607255825 2.05864234548082 NA NA NA NA NA NA NA NA NA NA NA NA 3.71848460342612
3.13276667468932 NA NA NA NA 2.58469461704505 NA NA NA 4.39915616586024 3.74723470434298 3.49781685545502 NA 1.89246021354369
2.96777492412562 NA NA 5.46498272424184 NA NA NA 2.94511566231081 4.41988174808731 NA -0.896170027558814 3.22650789976214
-1.25700162058509 0.211125713154788 0.710148198577697 -5.28392116611872 None
hsa-mir-876-3p 3.76094775980084 NA NA NA NA NA NA NA NA NA NA NA NA NA NA 3.95155098167213 NA NA NA 3.10659917800809
NA 2.96889367071057 NA NA NA 1.7873405971994 NA NA NA 1.36834711123896 NA NA NA 4.33650814403552 NA NA NA NA NA NA
0.991093592364242 3.04002677752364 1.00551018109377 0.316735690770748 0.763966486139044 -5.28401087452922 None
hsa-mir-320d 1.53820070958288 NA NA NA NA NA NA NA NA NA NA NA NA NA NA NA NA NA NA NA NA 2.02668031658683 NA NA
NA NA NA NA NA 1.62954957235992 NA NA NA NA NA NA NA NA 3.22992233073155 NA -0.757183363643221 2.10608823231529
-0.507223850142347 0.612982137872706 0.898995297558389 -5.28487453990681 None
hsa-mir-605 NA NA NA NA NA NA NA NA NA 3.58835536201299 NA NA NA NA NA NA NA NA NA NA NA NA NA NA NA NA NA
NA NA 5.07180009221004 NA NA NA NA NA NA NA 3.31212219834286 NA NA -0.603605783263459 3.99075921752196
-0.379377173696803 0.705123136216398 0.917276465053135 -5.28542682379457 None
hsa-mir-597 4.04505903982678 NA NA NA NA NA 3.9419037668097 2.57179949688059 NA NA NA NA NA NA NA 1.82411714694349 NA NA
NA NA NA NA NA NA NA NA NA NA NA 2.71462390017019 NA NA NA NA NA NA NA 1.51399023371409 NA NA 0.981412795673001
2.76858226405747 0.877717410988884 0.381919686022159 0.818464062400206 -5.29057582481608 None
hsa-mir-622 3.10524426084624 NA NA NA NA NA 4.02400395481484 4.86447240604442 NA 4.58694399185604 NA NA NA NA NA
0.502817678719349 NA NA NA NA NA NA NA NA NA NA NA NA NA NA NA NA NA NA NA NA NA 4.2540017910065 NA NA
-0.837305332550323 3.55624734721457 -0.579797098361931 0.563181031964466 0.895722046824318 -5.29286391704618 None
hsa-mir-875-3p 2.83207320551106 NA NA NA NA NA NA NA NA NA NA NA NA NA NA NA 3.57146908218077 NA NA NA NA NA NA
NA NA NA NA NA NA NA NA NA NA NA NA NA NA 2.64532951921445 NA NA 0.556441624631465 3.01629060230209

0.350914129954869 0.726309218266589 0.919356179319592 -5.29354475707125 None
hsa-mir-548d-3p NA NA NA NA NA NA 3.71719630243338 5.10283594724778 NA NA NA NA NA NA NA 3.41520864584342 3.51971511101784 NA NA NA NA 0.387469009571199 NA NA NA NA 3.3749703616355 NA NA NA NA NA NA NA NA 5.06396021179486 NA NA NA NA 0.996605807301751 3.51162222707771 0.992477411275527 0.323022128699535 0.76971986556492 -5.29570196998451 None
hsa-mir-769-3p NA NA NA NA NA NA NA NA NA NA NA NA NA NA NA 2.03471248700146 NA NA NA NA 2.45074345593974 2.2267951953546 NA NA NA NA NA NA NA NA NA NA NA NA NA NA NA 3.5645959589848 NA -0.71266571642492 2.56921177432015 -0.477858617308466 0.63367011865919 0.898995297558389 -5.29644716891434 None
hsa-mir-550 NA NA NA NA NA NA NA NA NA NA NA NA NA NA NA NA 3.03207953613494 NA NA NA NA NA NA NA NA 2.74991225183479 NA NA NA NA NA NA NA NA NA NA NA 2.25019534180496 NA NA 0.532025739315065 2.6773957099249 0.335648716428241 0.737761089592822 0.923995650122037 -5.29763859586999 None
hsa-mir-555 1.33229272621951 NA NA 1.72317120746314 NA NA 3.63841405575253 NA NA NA NA NA NA NA NA 1.60399243782346 NA NA NA NA NA 2.87851482921036 NA NA NA NA 4.16456214029388 NA NA NA NA NA NA NA NA NA NA 2.10610416273407 NA NA -0.975259437264778 2.49243593707099 -0.989998849660435 0.324226941713913 0.76971986556492 -5.29790843207298 None
hsa-mir-516b 0.763414351122794 2.23205235192989 NA 3.4923918625846 NA NA NA NA 5.75245010536888 3.41950104059543 NA 0.408216102214426 NA NA NA 1.11995161115222 NA NA NA NA NA NA NA NA NA 3.10714963529279 NA NA NA NA 3.3659465270805 NA NA NA NA NA 4.57334936069545 NA 2.61841938653647 NA -0.960790880977268 2.80480384859759 -1.15527976624061 0.250262137346717 0.731348500441128 -5.29917364097695 None
hsa-mir-886-5p NA NA NA NA NA NA NA NA NA 3.29614126102229 NA NA NA NA NA NA NA NA NA NA NA NA NA NA NA NA NA NA NA NA 3.70044263384088 NA 1.93103580480345 NA NA NA NA NA NA NA 0.480402041700126 2.97587323322221 0.301927735671527 0.763264742602166 0.92518658980654 -5.3060380272731 None
hsa-mir-1913 0.937507738131364 NA NA NA NA NA NA NA NA NA NA NA NA NA NA 3.73284826617158 NA NA NA NA NA 2.91328897647481 NA NA NA NA NA 2.60489148263708 NA 4.26073623722049 2.96618623079286 NA NA NA NA NA 4.61775031828496 NA 2.4434629899563 NA -0.965874703742945 3.05958402995868 -0.915097939112172 0.362014845589158 0.799563015989105 -5.30786812361421 None
hsa-mir-518d-3p 2.51143902727155 3.50244810961619 NA NA NA NA NA NA NA NA NA 3.39786152320124 NA NA 5.3867947390666 3.79907608133448 3.59801456834634 NA NA 3.37771131098729 NA 2.45772042819238 NA NA NA NA NA NA NA 0.737482606335883 NA

NA NA NA 3.02634292079318 NA NA 4.68807731392879 NA NA 0.925929234090826 3.31663351173399 1.14446776406854 0.254698482740691 0.731348500441128 -5.3106895955955 None
hsa-mir-629 NA 1.96477819632021 3.12434002112282 3.30701809759315 NA 4.24793898270563 4.12148421724291 NA 2.68200613745056 2.89827745280958 0.0344939558814166 0.649914685347091 3.13173864209498 NA NA 2.4925201704365 1.96698337317637 NA NA 3.75651985300685 4.00954858351089 3.52940562380482 NA 2.5490007262931 NA NA 3.94536369590233 4.28971509001859 NA 3.25310862645068 NA NA NA NA NA 4.41876989683316 NA 2.57345222055801 2.22708691662753 NA -0.77725422113928 2.96243023478124 -1.41063354537742 0.160714455741839 0.657032302917723 -5.31070848426158 None
hsa-mir-1183 NA NA NA 3.03148747675669 NA NA NA 3.97958334017896 NA NA NA NA NA NA NA NA NA NA NA NA NA NA NA NA NA NA NA NA NA NA NA NA NA NA NA NA NA 3.12252837468683 NA NA 0.383007033780995 3.37786639720749 0.241426686196514 0.809664038963128 0.943796895210634 -5.31888474009462 None
hsa-mir-554 NA NA NA 1.53055268685152 NA NA NA NA NA NA NA NA NA NA NA NA NA NA NA NA NA NA NA NA NA NA NA 1.20780624505372 NA NA NA NA NA NA NA NA NA 2.53864427924999 NA NA -0.342672575300335 1.75900107038508 -0.215752880328656 0.829571171385281 0.950544070924647 -5.323472792757 None
hsa-mir-1227 NA NA NA NA NA NA NA NA NA NA NA NA NA NA NA 3.65850242170266 NA NA NA NA NA NA NA NA NA NA NA NA NA NA 4.19777351180445 NA NA NA NA NA 4.85132824323598 NA 3.69049813117827 NA -0.588030873703574 4.09952557698034 -0.39465264330545 0.693840068787969 0.917276465053135 -5.32550647592438 None
hsa-mir-637 NA NA NA NA NA NA NA NA NA 4.04583211078134 NA NA NA NA NA 4.36645655227402 NA NA NA NA NA 2.89957222953798 NA NA NA NA NA NA NA NA NA NA NA NA NA NA NA 2.34102236147531 4.66828242515286 NA 0.903185326138963 3.6642331358443 0.765420015528131 0.445596768333208 0.849614704748016 -5.32918844950092 None
hsa-mir-876-5p 2.43817808603395 NA NA NA NA NA NA NA NA NA NA NA NA NA NA 3.06267383010004 NA NA NA NA NA NA NA NA NA NA NA 2.46729078845345 NA NA NA NA NA NA NA NA NA NA NA NA 0.283135169613544 2.65604756819581 0.178593369838321 0.858578306358572 0.969518199876815 -5.32920156306363 None
hsa-mir-580 1.75425770777631 5.60802070899899 1.86383649103325 3.69963113175216 NA NA 3.25285062092709 1.44537666758264 3.11872179028568 4.86098941218214 NA 3.08980830563486 NA NA NA 2.02234415727511 3.54990671061314 NA NA 2.78131284939439 NA 3.34282046640915 3.53471368163017 NA NA NA NA 2.87049016643805 3.1508237053863 3.98538544412 NA NA NA NA 2.36866841434028 4.35890574602248 NA 3.96267950602502 4.98083973801073 6.02622922252914 -0.770900896303151 3.43766421110759 -1.40504090801793 0.162367586185317

0.657032302917723 -5.32962538685812 None
hsa-mir-492 NA NA NA 2.41189307424315 NA NA NA NA NA NA NA NA NA NA NA 1.87544064515043 NA NA NA NA NA
1.94661494084187 NA NA NA NA NA NA NA NA NA NA NA NA NA NA NA NA NA NA 0.19705191885492 2.07798288674515
0.124311434515824 0.901290597660175 0.985980621282861 -5.33563064119066 None
hsa-mir-559 0.379903065965026 NA NA NA NA NA NA NA NA NA NA NA NA NA NA 0.550549374794948 NA NA NA NA NA NA
NA NA NA NA NA NA NA 0.296531534805821 NA NA NA NA NA NA NA NA NA NA 0.168694685574166 0.408994658521932
0.106458642758419 0.915408122287768 0.985980621282861 -5.33724173735717 None
hsa-mir-218 4.78973662226009 5.57904642671347 3.59231086088516 4.88352964510506 5.44819777065128 6.0435039143295 4.84071968142944
3.94213912484933 5.8180067010716 5.7440806689733 6.2816683766982 4.30123091210821 4.48797476237088 6.7995778179439 6.30433681273833
5.47631491802813 3.96470030557394 4.95948668701329 6.43264911832679 6.50624582296887 6.40195828062963 8.5719289967687 7.50141978665648
5.93459360886945 7.07740478886857 5.60347434795472 5.36216050717988 6.02104217923052 6.82277079774665 4.5479363369383 5.96066683205822
7.29947353417443 6.19492259384898 3.66311605857496 7.01036471310919 3.73982929917803 5.09232157668842 6.31009536849468 4.26809544032224
5.46373167188577 -0.632592488456954 5.62606909173041 -1.59089695958486 0.113745560452403 0.557630674412999 -5.33889402065678
None
hsa-mir-519c-3p 2.06933066702322 NA NA NA NA NA NA NA NA NA NA NA NA NA NA NA 2.05078491772734 NA NA NA NA NA NA
NA NA NA NA NA NA NA NA NA NA NA NA NA NA 2.17668238710179 NA NA -0.116624594726511 2.09893265728412
-0.0736014324670216 0.94145827128832 0.992203913441046 -5.33955544069233 None
hsa-mir-152 4.974315378308 7.01042796523141 5.9774369415062 5.8937043573534 4.61908377542483 5.3592495079266 3.85371532663387
6.12203362163293 4.27987707974081 4.9370555304017 4.69233460274804 4.69420797289776 4.03387570011403 6.27289151009712 4.39331560248458
3.31266055699938 7.33210901842683 4.90121264373797 5.45971588576813 5.86253984677016 5.57790372921832 5.80019298631196 5.69613453050834
5.6680451898133 6.9561253878996 6.9880785612221 5.94842718538582 5.91545205816761 5.55587223174186 4.67053318770192 7.32482800658792
6.84606075332176 4.13280412978184 NA 5.18026072762943 4.22968839717443 7.95670355707587 5.48592621864383 6.17721289075011
4.61615203489835 -0.628617214781094 5.50533755353944 -1.58163550860017 0.115860769871764 0.560694502252773 -5.34105012667925
None
hsa-mir-608 NA NA NA NA NA NA NA 2.22004307116496 NA NA NA NA NA NA NA 2.24301754781809 NA NA NA NA NA
4.22827749828254 NA NA NA NA NA NA NA NA NA NA NA NA NA NA NA 1.85364543342687 NA NA -0.80943115636318

2.63624588767312 -0.623757466748309 0.534038804356725 0.890713562955474 -5.34133505015755 None
hsa-mir-1181 1.8449240866136 NA NA NA NA NA NA NA NA NA NA NA NA NA NA NA NA NA NA NA 2.51763547793455 NA NA NA
NA 2.57734592995428 NA NA NA 1.91670172341909 NA NA NA NA NA NA NA NA NA NA -0.492303623822373 2.21415180448038
-0.330765328501561 0.741431744693294 0.923995650122037 -5.34406897916325 None
hsa-mir-615-3p 1.91120054135483 NA NA NA NA NA NA NA NA NA NA NA NA NA NA NA NA NA NA NA NA NA NA NA NA
NA NA NA 2.85479691350266 NA NA NA NA 4.14319268862621 NA NA 0.161127747495472 NA NA -0.475171908519951
2.26757947274479 -0.312697045480145 0.755084802203468 0.923995650122037 -5.34872706163087 None
hsa-mir-208b 0.765518245908505 NA NA 1.52106539543357 NA NA NA NA NA 4.66116225473343 NA 2.15751587188269 NA NA NA NA NA
NA NA 2.21474447394694 NA NA NA NA NA NA NA NA NA NA NA NA NA NA NA NA NA 3.67608518546234 2.66302153132727 NA
-0.905552110013778 2.52273042267068 -0.831527560946544 0.407377201732006 0.834709667241015 -5.35279144349938 None
hsa-mir-548c-5p 3.17666857205866 NA NA 3.11172290031596 NA NA NA NA NA NA NA NA NA NA NA 1.19276590411536 NA NA NA NA
NA NA NA NA NA NA NA NA NA NA NA NA NA NA NA NA NA 2.06192098750038 NA NA 0.43179813799628 2.38576959099759
0.288379504464098 0.773582371878894 0.925536052069392 -5.35458408607342 None
hsa-mir-33b 2.17832654597217 NA 2.91821889917533 2.53795362803329 NA NA NA 4.4554080354126 NA NA NA NA NA NA NA
2.71699829265962 2.05182184228409 NA NA NA NA NA NA NA NA NA NA NA NA 3.43429938012117 NA NA NA NA NA NA NA NA
NA NA -0.62451150619832 2.89900380337975 -0.450283258975573 0.653343242842411 0.898995297558389 -5.35836718927724 None
hsa-mir-520d-3p 0.282102597501947 NA NA NA NA NA NA NA NA 3.62305059823173 NA NA NA NA NA NA NA 2.50479750154121 NA
NA NA 3.63430082148378 NA NA NA NA NA 4.74688307230329 NA 0.325426366202077 NA NA NA NA 4.27569716271335 NA NA
2.33912950214819 NA NA -0.927637152545175 2.7164234527657 -0.957590102955874 0.340236157333013 0.7787256184192
-5.36308575221371 None
hsa-mir-508-5p 1.79968235932509 NA NA NA NA NA NA NA NA NA NA NA NA NA NA NA NA NA NA NA NA 2.021600757129 NA
NA NA NA NA NA NA 2.2254087199103 NA NA NA NA NA NA NA 1.8878130075018 NA NA -0.245258468855277 1.98362621096655
-0.164873973322432 0.869336539150591 0.976444950362989 -5.37702382694855 None
hsa-mir-18b NA NA NA NA NA NA NA 3.48495707698703 NA NA NA NA NA NA NA NA NA NA NA NA NA 3.41357631163873 NA NA
NA NA NA NA NA 0.933619817429241 NA 2.71446879903408 NA NA NA NA NA NA 5.20396629035271 NA 0.41854927237334
3.15011765908836 0.284919346395033 0.77622077038177 0.926853711960807 -5.38078650358901 None

hsa-mir-657 3.12405752484183 3.46958027781102 NA 2.4767232453319 NA NA 2.10685930217652 NA NA NA NA NA NA NA NA 3.68139059098092 1.28957143385465 NA NA 2.91312190973914 NA NA NA NA NA NA NA NA 2.30630241134416 NA NA NA NA NA NA NA NA 4.86595334997929 NA -0.863084411413727 2.91484000511771 -0.837535314209125 0.403978180590037 0.834242612656822 -5.38302944489122 None
hsa-mir-647 1.57462250733674 NA NA NA NA NA NA NA NA 3.4858996410437 NA NA NA NA NA 2.55011999683926 NA NA NA NA NA 4.24408556143055 NA NA NA NA NA NA NA 2.98084564661426 NA NA NA NA NA NA NA 2.95831646021791 NA NA -0.857535174347674 2.96564830224707 -0.81625332464374 0.416035978526027 0.835205686670525 -5.38374909387742 None
hsa-mir-1303 NA NA NA NA NA NA NA 3.06771552254222 NA NA NA NA NA NA NA NA NA NA NA NA NA NA NA NA NA NA 3.40462939206807 4.29953767034887 NA NA NA NA NA NA NA NA NA 1.59028252089321 NA NA -0.0304343385611632 3.09054127646309 -0.0202584769649119 0.983872759874835 0.992203913441046 -5.38776071454729 None
hsa-mir-760 2.53451593355759 NA NA NA NA NA NA 4.05251503991934 NA NA NA 2.43468254632119 NA 4.0858762739067 NA NA NA NA NA NA NA 2.25330947774005 NA NA NA NA NA NA NA NA NA NA NA NA NA NA NA 1.77421050198606 3.24514135900609 NA 0.852677002182137 2.911464447491 0.86975287378394 0.386224402648884 0.820046883089004 -5.3984348656055 None
hsa-mir-2113 2.11697060258627 4.70534651525067 NA 3.30957861534973 NA 4.03519535037532 NA 1.35508772760702 2.475215881986 5.69517938386271 NA 2.43631283836673 NA NA NA 3.39218013915157 NA 2.80735288798189 NA 3.25133558188544 NA 2.43639259298603 NA NA NA 4.93390508337384 NA NA NA NA NA NA NA NA 3.48082834148554 NA 5.97470205599052 3.48143475528915 NA NA -0.826929336333802 3.49293864709553 -1.18210365041213 0.239400013373318 0.721791040320554 -5.39898345422337 None
hsa-mir-302b NA NA NA NA NA NA NA NA NA 4.04739080300764 NA NA NA NA NA NA NA NA NA NA NA 2.45148476067036 NA NA NA NA NA 4.13365554024332 NA 3.25789414406226 NA NA NA NA NA NA 5.67548319495558 NA NA NA 0.16776139302476 3.91318168858783 0.115268772945968 0.908434047473267 0.985980621282861 -5.4083549627195 None
hsa-mir-520g 1.29504557299107 NA NA NA NA NA 3.86327532852833 NA 4.1731646175912 NA NA NA NA NA NA 1.86226275852417 NA NA NA 3.33690754285867 NA 2.70145171744731 NA NA NA NA NA 3.37512847554634 3.10979552080167 NA 7.22716909470836 NA NA 2.47393092258213 NA NA NA NA NA NA -0.871363982118473 3.34181315515792 -1.03913921154609 0.30083848597596 0.756336745107844 -5.4095946544927 None
hsa-mir-614 NA 4.33664230883908 NA NA NA 3.8644918256305 NA 3.37929275627513 NA NA NA NA NA NA NA NA NA NA NA NA NA NA NA NA 3.19127057573819 NA NA NA NA 5.06446031144804 NA NA NA NA NA 5.73828479976288 NA NA NA -0.8045295987348

4.2624070962823 -0.763741211023803 0.446579204173796 0.849614704748016 -5.42059634438922 None
hsa-mir-623 0.312340749577194 NA NA NA NA NA NA 2.53316696037356 NA NA NA 3.39381214810624 NA 1.66573083888737 NA NA NA NA NA NA NA 2.19932079047536 NA 3.21497690671498 NA NA NA NA 5.01511968521271 2.29642393652563 NA NA NA NA NA NA NA 1.4030275422916 NA NA -0.849511098007965 2.44821328424052 -0.972026921493017 0.333017598543591 0.772344661237635 -5.42168039282603 None
hsa-mir-1251 4.85036260095326 4.64360055634434 2.14808887182885 3.8331523474183 NA NA NA NA NA 2.99357917992586 NA NA 4.31052712932689 3.50070863785515 NA 2.60833683853636 2.50361360348428 NA NA 1.67784266769306 2.82134494570846 3.64639801456689 NA NA NA NA NA 4.30870661436341 NA 4.55459032669232 4.96036060292863 NA NA 4.51458456879685 4.0799819134927 NA NA 2.86566624677498 4.67403512449805 NA -0.740315240865842 3.65765688374677 -1.27923753584797 0.203119102037772 0.710148198577697 -5.42776204408822 None
hsa-mir-762 2.80652087518234 NA NA NA NA NA NA NA NA NA NA NA NA NA NA 0.969619330632148 NA NA NA NA NA NA NA NA NA NA 2.75034519719504 NA NA NA NA NA NA NA NA NA NA 2.12141102383222 NA NA -0.547808007606386 2.16197410671044 -0.423183946220737 0.672962044056798 0.911469750803578 -5.42984798731124 None
hsa-mir-28-3p NA NA NA NA NA NA 1.94146823342821 NA NA NA NA NA NA NA NA NA NA NA NA NA NA 2.06261857441793 NA 2.32965841944987 NA NA NA 2.10006565288507 1.90102302288987 NA NA NA NA NA 3.15280390143782 NA NA 1.46423689954032 NA NA -0.226932845008604 2.13598210057844 -0.16456661259978 0.869570378681468 0.976444950362989 -5.43057904689127 None
hsa-mir-1305 NA NA NA NA NA NA NA 1.44680230391766 3.75832752316196 NA 2.91238367723222 NA NA NA NA 2.07824883661968 2.32128609884466 NA NA 3.06570996733739 0.0663122700376664 3.41303528377245 NA NA 3.89919942704075 NA 4.21970873933598 NA NA NA 5.57955134847609 NA NA 3.37203149775078 NA NA NA NA 3.34867519430969 NA -0.816946993203462 3.03702093598746 -1.12572622846499 0.262482603106583 0.739612194734905 -5.43308029606369 None
hsa-mir-449a 0.969711766622156 NA NA NA NA NA NA NA 2.20588900592184 2.98929442671406 NA NA NA NA NA 3.48882688642421 2.86004459447227 NA NA NA NA 1.22603496505456 NA NA NA 3.59609436188733 NA 2.10773203198543 NA 9.08895899425366 NA NA NA NA 2.87817242400667 NA NA 1.45147007317817 NA NA -0.888657139030064 2.98747541186549 -1.05098458755896 0.295369322230152 0.754693649596531 -5.43553556127036 None
hsa-mir-1231 2.71604529490506 NA NA NA NA NA NA 3.85657985854468 3.35142724215888 3.36593729721812 NA NA NA NA NA 4.39341285615134 1.47084546668056 NA NA NA NA NA NA NA NA NA NA NA NA NA NA NA NA NA NA NA NA 3.03896791547317

NA NA 0.153406753803271 3.17045941873312 0.110220931871039 0.912423859015857 0.985980621282861 -5.43672354450893 None
hsa-mir-511 NA NA NA 2.53234678633171 NA NA NA NA NA NA NA NA NA NA NA 3.26922569567905 NA NA NA NA NA NA NA NA
NA NA NA NA NA 2.24848417291187 NA NA NA NA NA NA NA 2.55854685782241 NA NA 0.49727072563824 2.65215087818626
0.385734713772674 0.700415377173397 0.917276465053135 -5.44263272296143 None
hsa-mir-1284 1.10661954208656 NA NA NA NA NA NA 4.81288336798252 NA NA NA NA NA NA NA NA NA NA NA NA NA NA NA NA
NA NA NA NA NA 2.59418142499246 NA NA NA NA NA NA NA 0.656009103657053 3.58361958162915 NA 0.681814751608318
2.55066260406955 0.565671923053534 0.572723978314732 0.895722046824318 -5.44417774329365 None
hsa-mir-106a+hsa-mir-17 5.0013697058883 2.53973817827003 4.16155585609648 4.50465135948991 5.25497978205365 5.06261353236425
3.02133642674207 5.94108251795068 3.65912856116852 7.30585640130851 4.64946119883541 4.3756428596964 3.69753548308848 3.53309037364151
6.17324633993787 4.64306225601616 6.02128800018839 4.95936737143762 6.95817633787955 4.35586551187042 5.53993860636742 2.93952353031089
4.91772867371645 6.85291880718375 4.83534972613877 3.2854012189805 5.98962527541311 6.06749399610664 3.99187541107943 6.02015816513481
6.38050712285111 5.85708974340987 5.64888359439597 5.76221410950037 3.27872936100514 4.67941415695948 6.21875446247192 5.76097861245473
7.57945960426954 6.56500578237798 -0.617600095310183 5.0997524503513 -1.51870312327559 0.130949600303475 0.593703826939815
-5.44795165661454 None
hsa-mir-1250 1.08129724747558 4.17532472827512 NA 3.78795417656998 NA NA NA NA NA 4.34053909103243 NA NA NA NA NA
3.44450388609519 2.62819911094962 NA NA NA NA 1.98147804624134 NA NA NA NA NA 2.98160892542097 NA NA NA NA NA NA NA
NA NA NA NA NA 0.761426220901833 3.05261315150753 0.721547775510645 0.472006285994827 0.862756303026751
-5.44847193911997 None
hsa-mir-1290 1.9814495738629 1.65111045174002 5.05525472784732 NA NA NA NA 4.49702263439955 NA NA NA NA NA NA NA NA NA NA
NA NA NA NA NA NA 4.66722415971806 NA NA NA NA NA NA NA NA NA NA NA NA NA 3.35443713575874 NA
-0.714621300775952 3.5344164472211 -0.630010846546597 0.529932211010202 0.890713562955474 -5.45416142116727 None
hsa-mir-1537 1.79455033821698 NA 3.15158795208612 5.52690977074004 NA NA 2.77052301869315 2.10854921823457 NA 5.51013387895813
4.40236005772449 4.08802099349075 3.36295952196665 NA NA 3.72152121460725 2.86544065617044 NA NA 4.12586596767216 2.56073619673972
5.72420561851136 NA NA NA NA 3.41653639658307 3.52709998265103 2.51845207161873 2.62428589293161 NA 2.94976704303973
4.21524901694831 NA 2.01158665746896 NA 2.20376332198695 0.167546238645188 NA NA 0.717287175974791 3.27598482720371
1.32380846434858 0.187846554558074 0.699206619743943 -5.45906213839757 None

hsa-mir-1978 NA 4.7956208538882 NA 1.79681351231027 NA NA NA NA NA NA NA NA NA NA NA 3.46370636606674 NA NA NA NA NA
2.00822423470997 NA NA NA NA NA NA NA 3.44638989430292 NA NA NA NA NA NA NA NA NA NA 0.624739846248626
3.10215097225562 0.525957847204763 0.599934980879116 0.898995297558389 -5.46295587265561 None
hsa-mir-767-3p 2.30851199223941 NA NA 2.4959347752267 NA NA NA NA NA 5.0346978071826 NA NA NA NA NA 0.859707401352336
NA NA NA NA NA 0.584046858530412 NA NA NA NA NA NA NA NA NA NA NA NA 4.90974287459198 NA 4.92691286673631 NA
NA NA -0.798854539285972 3.01707922512282 -0.779294355747103 0.437386925830569 0.848349147761226 -5.46560837397351 None
hsa-mir-571 0.926220886430539 2.886765790092 3.10188651841052 NA NA NA NA 4.15398920496836 2.82218195097489 4.34790392023284 NA
NA NA NA NA 2.25055920060461 NA NA NA 3.82826940903553 NA NA NA NA 4.49079218770209 NA NA NA NA NA 3.52124704334327
NA NA NA NA NA NA 3.46220835161296 NA NA -0.78502708412578 3.25382040576433 -0.905252461119984 0.367138534038588
0.800637806057634 -5.4715237292744 None
hsa-mir-412 NA NA NA NA NA NA NA NA 3.68300146726264 NA NA NA NA NA NA 2.54811831527557 NA NA NA NA NA NA NA NA
NA NA NA NA NA NA 5.61009880004607 NA NA NA NA NA NA 0.7085414791624 4.86781858602926 NA -0.613259730476806
3.48351572955519 -0.504640842536667 0.614781188786446 0.898995297558389 -5.47247482261206 None
hsa-mir-588 NA NA NA NA NA NA NA NA 1.9155327099128 NA NA NA NA NA NA 3.77369552698597 NA NA NA NA NA NA NA NA
NA NA NA 3.53806524761009 NA NA NA NA NA NA NA NA 5.81145720981989 0.94346017558078 NA NA -0.586380092554203
3.19644217398191 -0.483728116458605 0.62950291167686 0.898995297558389 -5.48143249017309 None
hsa-mir-516a-3p 3.25273604521053 NA 3.41855971309843 3.31789605254304 NA NA NA 4.30232051568028 NA 4.14841516791069 NA
1.22724567172831 NA 3.55941459350531 NA 3.01530232240285 5.24634110812972 NA NA 4.59596318796956 1.54105796046661 1.4072213427586
NA NA NA NA NA 4.42946446097348 NA 2.40351200397975 NA 3.46946673614053 2.64307391407053 NA NA NA NA 0.423581449203955
3.93707014712881 5.55945518531882 0.740207971146641 3.25779460937999 1.2302933022921 0.220835298057236 0.710148198577697
-5.48647993196629 None
hsa-mir-507 NA NA NA 2.74251176807901 NA NA NA NA NA NA NA NA NA NA NA 3.47580565089422 NA NA NA NA NA NA NA NA
NA NA NA 4.02151626837196 NA NA NA NA NA NA NA NA NA 2.72293128398937 NA NA -0.263065066694049 3.24069124283364
-0.203633117522321 0.83900495601747 0.957278465215654 -5.4879589828041 None
hsa-mir-766 1.74663576784375 2.3112118366608 NA NA NA NA 4.60192372776197 NA 1.75840482486973 3.4395000204863 2.18065689664941
1.82133816930271 NA 2.44996040083205 NA 2.18467410233342 NA NA NA 4.76443151677354 NA 3.39826776095257 NA 3.35322663502403 NA

NA NA NA 4.75335933710321 2.4612035835841 4.1191509463222 NA 3.3176977839575 1.93503235752905 4.20654858803399 NA NA NA 3.41502255842098 NA -0.714071779307257 3.06411825339165 -1.22726280263817 0.221967996085752 0.710148198577697 -5.4900421476571 None

hsa-mir-1298 1.50568852000254 NA NA 4.30847116360768 NA NA NA NA NA NA NA NA NA NA NA NA NA NA NA NA NA NA NA NA NA NA NA NA NA NA NA 4.60318685455878 NA NA NA 2.10436169421097 0.369328971829192 NA 0.548120668272129 2.57820744084183 0.452844975757317 0.651517512594415 0.898995297558389 -5.49397069215596 None

hsa-mir-517a 2.38126307428693 NA NA NA NA NA NA NA NA 4.10736589285011 NA NA NA NA NA NA NA NA NA NA NA NA NA NA NA NA NA 3.87155659927085 NA NA NA NA NA NA 3.03038500895489 NA NA NA NA NA -0.20665632054435 3.34764264384069 -0.159660235367564 0.873432583071206 0.977027038454738 -5.4947099511896 None

hsa-mir-541 NA 4.02864378177966 NA 1.38963817707448 NA NA NA NA 2.90852548386405 NA NA NA NA NA NA NA NA NA NA NA NA NA NA NA NA NA NA NA NA NA 1.60132259378461 NA NA NA NA NA NA NA 2.88726880928419 NA NA 0.531306779371664 2.5630797691574 0.448705421251358 0.654492430560751 0.898995297558389 -5.49558872062615 None

hsa-mir-512-3p NA NA NA NA NA NA NA NA NA 3.9658855770164 NA NA NA NA NA 1.36237643117641 NA NA NA NA NA NA NA NA NA NA NA NA NA 3.53595861005966 NA NA NA NA NA NA NA 1.62272084975821 NA NA 0.0847912741874697 2.62173536700267 0.064934712698828 0.948340244036871 0.992203913441046 -5.503702307266 None

hsa-mir-944 NA NA NA NA NA NA NA NA NA 3.36070662494188 NA NA NA NA NA NA NA NA NA 2.35104902441677 NA NA NA NA NA NA NA NA NA NA NA NA NA NA NA NA NA 2.68643350957433 2.92921367764404 NA 0.04805423107014 2.83185070914426 0.037254156045173 0.970347820266708 0.992203913441046 -5.50489805207145 None

hsa-mir-491-5p 0.859234872292889 2.3238215853925 3.00047875683287 NA NA NA NA NA NA NA NA NA NA NA NA NA NA NA NA NA 0.655919292391461 NA NA NA NA NA NA NA 4.46833349312965 NA NA NA NA NA NA NA NA NA NA -0.500947987921135 2.26155760000787 -0.417333531292591 0.677216144083258 0.911469750803578 -5.50736986021013 None

hsa-mir-1203 2.28332306827708 3.43607594722408 1.65197862441842 NA NA NA NA NA 3.82416117016999 NA NA 3.38429221154591 4.61143218954848 2.06751570368875 NA 1.81539715554551 4.84725256879411 NA NA NA NA 1.97855413848403 NA NA NA NA 5.05671678817684 3.49195553042017 NA NA 4.93250112902679 NA NA NA NA NA NA NA NA NA -0.762550936614476 3.33701201733232 -0.978309418656108 0.329849865282046 0.770661199795522 -5.51105104916614 None

hsa-mir-204 4.0643135796968 2.47366970192848 NA NA NA 2.5812710197084 NA NA NA NA NA NA NA NA NA NA NA 7.96003288263686

NA NA NA NA NA NA NA NA NA 2.66096528899292 NA NA NA 4.2897342376777 NA NA 3.73830854844556 NA NA NA NA NA
0.70681910428724 3.96689932272667 0.69024345810129 0.491414260821816 0.863522412677361 -5.52461580903309 None
hsa-mir-888 1.74403732353109 NA NA NA NA NA NA NA NA 4.68088635845456 NA NA NA NA NA 3.00617044150553 NA NA NA NA NA
3.27434857352824 NA NA NA NA NA 5.46564185924773 NA NA NA NA NA NA NA NA NA 2.50831220887835 NA NA
-0.605736172721046 3.44656612752425 -0.567530336867597 0.571455699542084 0.895722046824318 -5.53679415885341 None
hsa-mir-617 NA 3.98701849697078 NA NA NA 3.05375445769516 NA 1.54048092887154 NA 3.26478466496526 NA NA NA NA NA
1.64778407959034 3.46105803330667 NA NA NA NA 2.90702413467977 NA NA NA NA NA NA NA NA NA 3.9229124422996 NA NA NA
NA NA NA NA NA -0.589154844923057 2.97310215479739 -0.561759330461867 0.575350243793433 0.896475961259535
-5.53971394450377 None
hsa-mir-566 2.47250177076687 5.20005214012238 2.45568636998939 2.11414238271934 NA 2.96303328820777 4.57308508698762 NA 7.28707840138499
5.54357750278866 NA 3.54590743744373 4.26465699644487 1.80424501892171 NA 2.64034900873432 1.80763843376366 2.65925147727686 NA
1.02344421996491 0.0302618820347389 1.72879332506563 NA NA 4.9622752346581 NA 3.67614481167557 3.03274728712007 NA NA
4.99775203504148 6.34478794557084 NA 6.05831292556319 NA NA 5.19209877461258 4.70478935868872 4.08079268197922 NA
-0.716546660845237 3.66013099221259 -1.29244945570946 0.19840170064342 0.710148198577697 -5.54470796561603 None
hsa-mir-519d NA NA NA NA NA NA 2.8731099568392 NA NA NA NA NA NA NA NA 2.62263122831727 NA NA NA NA NA
2.54168316423802 NA NA NA NA NA 3.49599688343627 NA 4.04461394094188 NA NA NA NA 4.75192007308116 NA NA 2.19021269898127
2.89469479149497 NA -0.571982999450694 3.17685784216625 -0.546588066134916 0.585698496163602 0.898214641126189
-5.54720377630533 None
hsa-mir-1301 NA 3.97839331374749 NA NA NA NA NA 3.9243036342621 NA NA NA NA 4.20721534827161 NA NA 0.654117159246701 NA
NA NA NA NA NA NA NA NA NA NA 3.81970389078173 NA NA NA NA NA NA NA NA NA 3.51414507378762 NA NA
-0.475917118402699 3.34964640334954 -0.420802866270634 0.674681192527006 0.911469750803578 -5.55073111783546 None
hsa-mir-551a 3.06204777779563 NA NA NA NA NA NA 5.16119434196144 NA NA NA NA NA NA NA NA NA NA NA NA NA
3.14495225896167 NA NA NA NA NA NA NA NA NA NA NA NA NA NA NA 3.14440592753113 5.08757919921455 NA 0.319308597976084
3.92003590109288 0.269397609526211 0.788108727150863 0.933778355913257 -5.55145044993775 None
hsa-mir-1201 3.6887681551078 NA NA NA NA NA NA NA NA NA NA NA NA NA NA 4.34330437280401 0.822805023932879 NA NA NA
NA 3.78796217707445 NA NA NA NA NA NA NA NA NA NA NA NA NA NA NA NA 2.7534236432208 NA -0.319067059532729

3.07925267442799 -0.267243213975758 0.789762788583352 0.933778355913257 -5.55195235075078 None
hsa-mir-501-3p 0.115872916813468 NA NA NA NA NA 1.87693683000517 4.43030267478885 4.46419784691482 NA 1.96604528190617 NA NA
NA NA 1.63643575854971 4.47305973075149 NA NA NA 2.51449460721087 4.05374398712448 NA 1.8486936928328 NA NA NA
2.09381047751688 5.40247934699484 3.03698885477012 NA 4.75001644623085 NA 3.75676666458561 3.2238962009455 NA NA 3.05998190267087
3.92992964055881 NA -0.715639627442402 3.14631404784285 -1.13086890248392 0.260233473482083 0.739612194734905
-5.55257597342933 None
hsa-mir-670 2.37530457635993 NA NA NA NA NA NA NA NA NA NA NA NA NA NA 3.93913280297057 NA NA NA NA NA
2.64161045807779 NA NA NA NA NA 1.13824841514471 NA 4.27842702397154 NA NA NA 6.76046332370046 5.119432989463 NA NA
3.74168813949434 2.56176424945155 NA -0.591586253092378 3.61734133095932 -0.553975208427592 0.580639952080888 0.89755603144822
-5.55917190069182 None
hsa-mir-886-3p NA NA 3.5020226258103 NA NA NA NA 3.97927958092542 NA 4.74758709365958 NA NA NA NA NA 3.51553097373876 NA
NA NA NA NA NA NA NA NA NA NA NA NA 2.6999677266879 NA NA NA NA NA NA NA 2.21583029325654 5.02424243379555 NA
0.622758250620185 3.66920867541058 0.632122056830609 0.528546017386397 0.890713562955474 -5.55930547259312 None
hsa-mir-1257 2.38932527946497 NA NA 1.92507549454832 NA NA NA 4.83980994545379 NA 3.99625105199542 NA NA NA NA NA NA NA NA
NA NA NA NA NA NA NA 2.45758263445985 NA NA NA 2.10095415855435 NA NA NA NA NA NA 3.47059493509213 NA NA NA
0.611238200163514 3.02565621422412 0.618753108515294 0.53728730584007 0.890713562955474 -5.56685665934481 None
hsa-mir-147b 1.85386707203898 3.74856621311933 NA NA NA NA NA NA 3.77392910517914 NA NA NA NA NA NA 3.69615526041179 NA NA
NA NA NA 1.00394923310946 NA NA NA NA NA NA NA NA NA NA NA NA NA NA NA 1.92328499217591 5.02143989857836 NA
0.618571371399399 3.00302739637328 0.618376013613547 0.537534932374216 0.890713562955474 -5.56706733040145 None
hsa-mir-125a-5p 9.36745880792786 9.3286318520339 8.6916746566352 7.84912323933843 8.03448048380582 8.08576437727423 8.44447992071524
8.88275715382019 9.21757952866473 10.3393688127012 6.71436509976916 10.249258325128 8.41939377573508 6.99370527373193 7.30457033083523
7.90563086019973 10.382258546593 8.46166730236036 9.01844758507293 8.88736913275734 10.1021914395993 8.25302281781619 9.5779460343641
7.61972953957476 9.60694941020171 7.45357822092593 6.55727481223001 7.36716519123947 7.96669565805635 5.60308484025685 6.82560762434595
9.54632424353513 8.05064542747398 8.23952759819055 7.06958214812258 9.34434581072472 9.09210037827612 7.47561698069185 9.33859305765935
5.97028193804243 0.57588609468861 8.34095620591067 1.43542570084713 0.153255141803239 0.657032302917723 -5.56765168955151 None
hsa-mir-1182 2.62397379247456 NA NA NA NA NA NA 3.80084132363091 NA NA NA NA NA NA NA NA NA NA NA NA 2.09315455026159

NA NA NA NA NA NA 1.7096520464639 NA NA NA NA NA 5.44812867259653 NA NA NA 1.92451657574868 NA NA 0.418544596785059
2.93337782686269 0.36868509424506 0.71303756427225 0.918721477043092 -5.56882867881105 None
hsa-mir-1294 2.2815986185275 NA 2.84740677363576 3.7780536411733 NA NA NA NA NA 4.13030726534856 NA NA NA NA NA
2.71105750848727 2.72264620060115 NA NA NA NA 0.596005548827664 NA NA NA NA 2.44613862931039 NA NA 3.14640982018475 NA
NA NA NA 5.09261064875568 NA 4.85468011622589 3.67980653294088 5.90172427176426 4.61947099867518 -0.713594152873328
3.48627975531845 -1.01024362572291 0.314354261570415 0.761950701132497 -5.57577019064371 None
hsa-mir-548j 3.56327256964753 NA NA NA NA NA NA NA NA 5.11529303520407 NA 3.57058891134939 NA 4.01610120256523 NA NA NA NA
NA NA NA 4.18484257841568 NA NA NA NA 2.76451491346786 NA NA NA NA NA NA NA NA NA NA 3.50484463985012
3.24788068567964 NA 0.64079322533823 3.74591731702244 0.710855283623073 0.478582948981452 0.862756303026751 -5.57802739553933
None
hsa-mir-299-3p 1.88169021815551 NA NA NA NA NA NA NA NA 4.62181386407458 NA NA NA NA NA NA NA NA NA NA NA
1.38255490514909 NA NA NA NA NA 3.45794094480358 NA NA NA NA NA 5.23871617870625 NA NA NA NA NA NA
-0.107985301771262 3.3165432221778 -0.0896131596671575 0.928751231987836 0.985980621282861 -5.57947929632949 None
hsa-mir-516a-5p 1.93389537488344 NA NA NA NA NA NA 2.59774178581194 NA NA NA NA NA NA NA NA NA NA NA NA NA NA NA
NA NA NA NA 1.31700320919683 NA NA NA NA NA NA 2.69579222033513 NA NA 2.57541917847777 NA NA 0.0697470443444455
2.22397035374102 0.0593597676772435 0.952769062229135 0.992203913441046 -5.58143713165584 None
hsa-mir-548o 1.43813284048415 NA NA NA NA NA NA 2.13710694095376 2.86357908516323 NA NA NA NA NA NA 2.31807627385201 NA NA
NA NA NA 0.0209595611293982 NA NA NA NA NA NA NA NA 4.09531201719734 NA NA 3.05679001622991 NA 5.27394476214645 NA
1.34908403440528 3.47809717072822 NA -0.689807475192812 2.60310827022897 -0.811049601964131 0.418949999170005 0.836512746687128
-5.58757318225817 None
hsa-mir-525-3p NA 4.35550918195829 NA 2.59634926158015 NA NA 4.97089089662797 4.27400030234976 NA 4.04295416658024 NA NA NA NA
NA 2.13251921985589 NA NA NA 2.60192960976786 NA NA NA NA NA NA NA NA NA NA 3.44729698624355 NA NA NA NA NA
4.69890380810163 NA NA NA -0.505364305926852 3.68003927034059 -0.489667203946462 0.625274898897232 0.898995297558389
-5.58919708433717 None
hsa-mir-512-5p 0.662118177090589 3.80267271267044 2.13054560444375 NA NA NA NA NA NA NA NA NA NA NA NA 1.1695788190471
NA NA NA NA NA NA NA NA NA NA NA 2.25496558160313 NA 1.85187279924403 3.4013018894566 NA NA NA NA NA NA NA NA

NA -0.561484595121617 2.18186508336509 -0.567746912714534 0.571299649065641 0.895722046824318 -5.59419378330865 None
hsa-mir-639 3.74109069908842 2.38860531913789 NA 3.19980058693969 3.99286678856699 NA NA NA 3.32325981099008 NA NA NA NA NA NA
1.08638822173685 2.32970693175027 NA NA NA NA 4.62330659583424 NA NA NA NA 3.51299372862491 5.18905450100616 NA
4.61103546453684 3.02637114818882 NA NA NA 3.06111051305447 NA NA 1.74883624000139 2.5793984945224 NA -0.678053570262556
3.22758833626529 -1.02346016399125 0.308075324526725 0.756336745107844 -5.60128352730274 None
hsa-mir-1259 NA NA NA NA NA NA NA NA 1.9305774843477 NA NA NA NA NA NA 3.9563789303994 2.69607156166303 NA NA NA NA
2.61551335445537 NA NA NA NA NA 4.72924191725248 2.5589457591814 NA NA NA NA NA NA NA NA NA NA NA
-0.440224351493041 3.08112150121656 -0.416594784708569 0.677748203461025 0.911469750803578 -5.60300148854234 None
hsa-mir-302d 3.08930120094548 NA NA 2.23779227102145 NA NA NA NA NA NA NA NA NA NA NA 4.21733606513629 NA NA NA NA NA
2.77119811348053 NA NA NA NA NA NA NA NA NA NA NA 3.27548766242478 NA NA NA 2.19775464248846 NA NA 0.433329706236482
2.9648116592495 0.412854103464856 0.680479106858048 0.911842003189785 -5.60438544253527 None
hsa-mir-1197 NA NA NA NA NA NA NA 4.68418865210159 4.40030787127259 2.51247302620724 NA NA NA NA NA NA NA NA NA
2.41864578255447 NA NA NA NA NA NA NA 2.79516744033388 NA NA NA NA NA NA NA NA NA 4.7404169259941 NA NA
-0.263888350130016 3.59186661641064 -0.234777511751794 0.814797242024056 0.94485141719328 -5.6043920034893 None
hsa-mir-454 3.46169823321477 2.96085695158881 6.26472051052757 3.84091518476173 3.33704118087167 NA 5.85711873895785 5.02956668064269
3.98746317281821 NA 6.98460070544753 6.13699660859541 3.05958458219868 5.68293466776573 NA 4.42136898858372 3.94924553836451
4.28067042139123 NA 4.8262043432084 1.9154543711192 3.28318955375779 5.73165040524444 5.91299538147203 5.29166385351247 NA
3.71590274509635 4.26932675091894 5.50487238165231 4.6283027585603 NA 5.00777086368517 2.63880120620724 NA 4.58548932957548
4.18390583765479 4.3615049556235 2.03881565425226 1.1585732485097 NA 0.615797950756035 4.32216268143064 1.32950265447757
0.185822049786076 0.699206619743943 -5.60528414509547 None
hsa-mir-369-5p 2.14484336968995 0.942366508131602 NA NA NA NA NA NA NA NA NA NA NA NA NA 3.00237570514958 NA NA NA
2.27256123525534 NA NA NA NA 3.53030802160886 3.99598733007587 1.12738208071205 3.53167224754238 NA NA NA NA NA
2.24944265430445 NA NA NA 2.01039813414214 NA NA -0.650328373507674 2.48073372866122 -0.785281410808163 0.433843132525709
0.846625918812306 -5.60665816224919 None
hsa-mir-620 3.86886352940833 NA NA NA NA NA NA NA NA 7.02714530723305 NA 3.54164281083741 NA 3.03809408527538 NA
2.5590177228427 NA NA NA NA NA 1.80278454185388 3.85490234083274 NA NA NA NA NA NA NA NA NA NA 4.01534430905543

3.77390984271837 NA NA NA NA NA 0.645217432504269 3.72018938778414 0.73311715619867 0.464935138238277 0.857357456751318 -5.60939029030556 None
hsa-mir-1225-3p 2.0833549330289 2.19230941717942 NA 2.16516516087094 NA 2.71267639928968 3.57036548313 4.47671300154085 3.61007603607543 3.1932846620797 NA 3.73174347832545 1.93928457766986 2.95468549624305 NA 2.20281937783507 3.55674395817665 2.36159164256826 NA 1.3319060086349 3.27757991329207 3.36311184127563 4.98249071597534 NA NA NA 3.64916160993665 NA NA 2.85585502828753 3.07912722395796 4.99872441246641 NA 3.28726726055933 3.88393238200321 NA NA 0.77060190513571 3.47055879143803 NA -0.614340910944172 3.06542810449908 -1.23896576578271 0.217500520454605 0.710148198577697 -5.61009111864191 None
hsa-mir-25 8.946494356312 7.17033210720143 6.81483666548876 7.36514353885124 6.25267318772899 7.7804889805457 6.50319689441073 7.98964244716248 4.11794972448431 6.77457258231326 6.81902893066492 10.3657270247586 5.09672621358954 9.3683649463239 9.05900664183243 6.60420792403753 9.44128320623738 8.78022856460651 7.90439605113693 5.5212224869481 7.01704575786014 10.1840624931705 9.17566331754519 7.80268104279151 7.74992929897499 6.73966417511758 6.97678801000221 8.99231901576819 9.0500137950053 7.75458112293057 7.31695410994367 9.21590615997024 5.80835457520232 6.49679662624403 9.25355917621971 8.12692818902542 8.22691516340635 8.82617968663791 7.64251097818948 8.01165226131132 -0.584649124034099 7.72610068574878 -1.40231993452966 0.162893195723226 0.657032302917723 -5.61341432165426 None
hsa-mir-155 7.75242630965548 4.16216308229464 6.19984006499836 7.67047697516557 7.55283312678205 6.7280978938179 5.64713491105157 6.95660338874355 7.54713774893321 3.25058440974992 6.70584498497395 5.1844355642303 5.78319359557045 2.32109266505257 2.70394540786223 3.82534656561588 4.69792024511969 3.46877596135734 1.98047179303748 2.63069168612142 4.61164498706264 4.12867122757905 6.0953570266716 6.44369199339654 4.97046268680483 6.10801950015491 5.79242925568333 5.10651174842069 7.43839895322887 4.94791072712049 3.04224320580886 7.67312069379614 6.51748921581389 6.63055299461357 5.66538057762469 6.6729229279136 5.56844960100273 5.32575681566372 6.80971281354299 5.48866350012519 -0.613418703594738 5.44516017080405 -1.40134515997293 0.163183824647919 0.657032302917723 -5.61474601877461 None
hsa-mir-92b 5.20483845894842 5.23479409636259 4.86603323918815 5.87454206699195 4.930431043254 1.22444987666867 5.24243313098101 4.18586856666151 4.42648968490816 7.26547804376422 4.10469395165778 6.85358518716837 6.21239041070469 3.40069956945541 6.89401292313778 5.65672405821272 5.73306136706548 6.43204811519484 4.06956128173504 4.92862738449392 4.149408387309 5.73036828012156 7.33347758441709 6.26978225622267 4.94140203711627 7.08959103814294 5.10467373183101 5.9201218861362 6.33437683843608 6.36648502463552 6.03585464348799 4.89865373892869 5.21950856825174 5.17819215842943 5.66282150121432 5.20577058355036 5.71955962583935 6.01634070114569 6.65627020971303

4.11174695853869 -0.560182164845648 5.41712920525056 -1.40048412193495 0.163440871372568 0.657032302917723 -5.61592158383143 None
hsa-mir-628-5p NA NA NA NA NA NA NA NA NA 4.15778970988933 NA NA NA NA NA 2.95189806576726 NA NA NA NA NA 2.09952671825756 NA NA NA NA NA NA NA 5.19861071773517 NA NA NA NA 3.54864220952886 NA NA 4.12518475583535 4.37648767602156 NA -0.314846527647406 3.7797342647193 -0.291346559590625 0.771304512562158 0.92518658980654 -5.62123510236637 None
hsa-mir-496 0.277071423273554 NA NA 3.24831059057062 NA NA NA NA NA NA NA NA NA NA NA 3.2879711417651 2.23717684161402 NA NA NA NA 1.79066484950473 NA NA NA NA 4.08930601491158 NA NA 2.5329095854395 NA NA NA NA NA NA NA NA 2.97631847295396 NA -0.584667231396619 2.55496611500413 -0.638492874775951 0.524395757430372 0.890713562955474 -5.62273794469135 None
hsa-mir-576-3p 2.97977138633666 4.87863302476057 NA 3.70642563443895 NA NA 3.81413981674273 NA 2.99055514898656 5.01157178543795 NA 1.45428837989539 NA 4.36821982734478 NA 0.681456229548001 3.01243082967155 NA NA NA NA 2.93687774493256 NA NA NA 4.66702454480112 NA 2.62390854464968 NA NA 3.62662355126529 4.04851272598066 NA 5.14623703698072 4.78256619983858 4.70078660648768 5.12281930054658 2.87282449297844 NA 2.77117618060917 -0.64655596905373 3.62842138058255 -1.15054737461315 0.2520228582839 0.731348500441128 -5.62338556175192 None
hsa-mir-1827 NA 2.65025494619267 NA NA NA NA 1.47797880501071 4.03793971340236 NA NA NA NA NA NA NA 3.72780593650331 NA NA NA NA NA 3.80170014235266 NA NA NA NA NA NA NA 1.91688672056993 NA NA NA NA NA NA NA NA NA NA 0.114201418815968 2.93542771067194 0.101715782932809 0.91915869218585 0.985980621282861 -5.62410907000309 None
hsa-mir-220c 1.33471127898023 NA NA 3.83267321808387 NA NA 2.57658562978723 NA 3.82597442010968 3.06944495036864 NA NA NA 2.08653093054791 NA 3.88113248598325 3.2694201826536 NA NA NA NA 4.58336693598073 NA NA NA NA 4.45761577969304 2.82282427404279 NA NA NA NA NA NA NA NA 3.44490641608374 1.92853406938931 4.61692734940378 NA -0.657803333701264 3.2664748515077 -0.95601131166518 0.340934599108904 0.7787256184192 -5.62623569508322 None
hsa-mir-190 3.22083673215105 3.27796243501857 NA NA NA NA 3.82449740453117 1.04689021796713 NA NA NA NA NA NA NA 2.23237535246546 NA NA NA NA NA 1.92896384644943 NA NA NA NA NA 3.61192366745652 NA 0.701445776845835 NA NA NA NA NA NA NA 2.19886198354232 NA NA 0.610213609853149 2.44930637960305 0.704779370899037 0.482330798303859 0.862756303026751 -5.62820372014822 None

hsa-mir-1977 NA NA NA 3.60369761107212 NA NA NA NA NA NA NA NA NA NA NA NA 2.84686918029699 NA NA NA NA NA NA NA
NA NA NA NA NA 6.37895955560435 NA NA 2.44364505137139 NA NA NA NA 0.550970413676884 3.4701140543551 NA
0.014361126932624 3.21570931106281 0.0124059622251135 0.990123137272231 0.995862509864406 -5.62859855756379 None
hsa-mir-409-5p 2.76310215529915 1.7557521518476 2.49481539420316 2.46481261590708 NA NA 1.71909807490447 4.40348494310751 NA
3.26765401313173 2.58541346421923 2.38634035376634 3.14340093996271 NA NA 1.79138284583797 NA NA NA NA 1.66805873167517
1.74108203332237 NA 4.17981096692481 NA NA 3.6259339242194 4.64559328943468 2.11442783540268 NA 2.95857834492229 4.04204732822485
3.91676622821962 NA 4.77212418499736 NA NA 0.903711010139393 3.08914868073283 4.39918631615501 -0.619180540137375
2.95132190943989 -1.19406858976915 0.234568984236746 0.717995418755115 -5.63364117603187 None
hsa-mir-632 1.49067769108434 NA NA 3.93259728719273 NA 1.91429880001705 NA NA NA NA NA NA NA NA NA 2.93389067807919 NA NA
NA NA 1.79782640047925 3.38864191170816 NA NA NA 1.91178440156213 NA NA NA 4.65265688319333 4.17205221131982 NA NA NA NA
NA NA NA 3.18472172353806 NA -0.616747807873464 2.93791479881741 -0.741140123197073 0.460062781597289 0.857357456751318
-5.63793445218382 None
hsa-mir-584 1.46553344785155 NA 3.36335654626511 2.20027545030616 NA NA NA NA 2.5568848195743 4.15048022475796 NA NA NA NA NA
2.14078934535642 3.95067934064905 NA NA NA NA 3.48701782235698 NA NA NA NA NA NA NA 4.40032022193617 NA NA NA NA NA
NA NA 3.24349310077028 2.68206724161189 NA -0.620653285988752 3.05826341467599 -0.776549168326898 0.438946822722227
0.848349147761226 -5.63908517622384 None
hsa-mir-513a-3p NA NA NA NA NA NA NA 4.02289362030988 2.56788170697084 NA NA NA NA NA NA 1.29440431416892 NA NA NA NA
NA 1.52613987024143 NA NA NA NA NA NA NA NA NA NA NA NA NA NA 5.71813388748031 1.5909559485635 NA NA
-0.3166833549452 2.78673489128915 -0.292145603387668 0.770699550272182 0.92518658980654 -5.64235999888669 None
hsa-mir-619 NA NA NA 2.1871749895324 NA NA NA 3.19398707891956 NA NA NA NA 2.79299047251378 NA NA 1.34616431996807
2.3836194506231 NA NA NA NA NA NA NA NA NA NA NA NA NA NA NA NA NA 4.84704017017419 NA NA 0.321115497881275
3.50206859691027 NA -0.509287492677197 2.57177007206533 -0.533124193892601 0.594954795164386 0.898995297558389
-5.65074567644267 None
hsa-mir-320b 2.71796551631287 NA NA NA NA NA NA 3.77073341660034 1.69551721265886 NA NA NA NA NA NA 3.29491386157914
1.13343328074492 NA NA NA NA 3.42442287400191 NA NA NA NA NA 2.35816622989543 NA NA 2.23842507307909 NA 2.40767172293738
6.32529129239222 NA NA NA 1.78595517452733 3.61950533135103 NA -0.643121299304258 2.89766674884004 -0.840737638297929

0.402146850251865 0.834242612656822 -5.65129368729741 None
hsa-mir-520f NA NA NA NA NA NA NA 2.70724075962896 NA NA NA NA NA NA NA 3.38509760141086 NA NA NA NA NA
2.18922051592269 NA NA NA NA NA 4.59709496981778 NA 1.2666302897675 NA NA NA NA NA NA NA 2.94694694384177
3.71406148054694 NA 0.103378340540574 2.97232750870521 0.0954446565978873 0.924125669299916 0.985980621282861
-5.65487992225097 None
hsa-mir-1233 1.11320532801248 NA NA NA NA NA NA 4.02913771345377 NA NA NA NA NA NA NA 3.52103829685691 NA NA NA NA NA
2.53418378937835 NA NA NA NA NA 3.22891745072412 NA NA NA NA NA NA NA NA NA 0.754934496908697 3.36448265003322 NA
0.417164182679957 2.64941424648108 0.419514229982641 0.675613137617904 0.911469750803578 -5.66036390537728 None
hsa-mir-758 2.71540062017983 5.44232819857709 NA NA NA NA NA NA NA NA NA 4.61925821121289 NA NA NA NA NA NA NA NA NA
2.72200664156633 NA NA NA 3.17518066334956 NA NA NA 2.85882165072838 NA NA 3.84869718156523 5.97295397958423 NA NA
4.64400887758494 2.85720639432174 NA NA 0.533442049699403 3.88558624186702 0.596130672619549 0.552215731053636
0.895722046824318 -5.66832738852885 None
hsa-mir-101 6.44419808414996 7.1483705691313 5.86364844408649 6.99072392713165 5.50185186739806 9.13537491939574 6.21140315401955
5.697172134838 9.48615044633186 7.5285641651736 5.10811507017871 8.02148998096523 7.36445549326557 5.89067070327269 7.98981758429725
6.92125885552279 6.34401996438853 6.67561035689551 6.93064618962195 7.74377570190595 7.35244121481352 9.05795036214761 8.68641240161295
5.83307556510794 7.00554144614375 6.67900248837894 7.96711890779871 6.69093000110292 5.83704923691199 6.84736834347187 8.71609947103826
9.550570194261 7.311229579545 5.98033657862219 9.31163526445238 5.04011198988697 9.20895619304269 8.0600796695317 7.64694096561813
7.25297478973388 -0.5519253525626 7.22582855687982 -1.36109341422361 0.175532671706071 0.670878793960294 -5.66894848402809
None
hsa-mir-199a-3p+hsa-mir-199b-3p 12.2174519212272 11.5089209146626 11.2218874584582 11.6077035615801 9.77755202301703 9.01312376984726
11.2475445584214 10.1734014909468 12.4300450787022 10.9665358537289 12.2189940364154 11.6097815160395 11.2309178496902 10.1358905977907
8.31703746275203 11.6971996805963 10.7890922877654 11.7849772119723 8.58896335441177 11.4911546326258 10.7379506636923 11.63331101663
10.3131298422705 9.07070576265552 10.3333507469806 9.94163795828268 11.4893688193896 9.92084382166529 11.520134782964 9.39892060647693
9.82412113411376 9.04537622771585 9.23992627852397 11.9451377343924 10.1620556646018 10.8508427684369 10.4194801956976 12.0561681641809
11.2508510907676 8.0169007669438 0.542898060713455 10.6299597326758 1.36029098017572 0.175785819976329 0.670878793960294
-5.67001337879664 None

hsa-mir-548b-3p 2.04793374930793 2.83643607791455 3.94869419176479 2.66374911145655 NA NA 4.31458863691799 2.57980173301344 NA 2.45207588903079 NA 2.82525089477047 NA NA NA 4.56446785771836 3.61827873654128 NA NA 3.66167284998676 2.08176301503388 3.34531449223267 NA NA 4.82201893828084 2.97522577706452 6.58878723100406 3.2590047254923 5.87674379840697 3.24397404391528 3.3860646564057 3.74141134080408 NA 4.55850073573198 2.75121125242852 NA NA 2.65548991591565 4.29732316263549 NA -0.598895245071107 3.56383131255099 -1.17252136455177 0.243060795079059 0.725572571448875 -5.67370507735488 None

hsa-mir-718 2.51343558431335 4.68672109049709 NA NA NA NA NA NA NA NA NA NA NA NA NA 0.452303267738455 2.17423010376262 4.25020079194885 NA NA NA 2.1978409364047 NA NA NA NA NA 3.96490751411265 NA 2.70003052055358 NA NA NA NA NA NA NA 4.81452420151968 3.35214857484236 NA -0.59051218183452 3.11063425856933 -0.714234571600346 0.476475296795475 0.862756303026751 -5.67432730787771 None

hsa-mir-564 0.164517902119752 NA NA NA NA NA NA 4.87182218946492 NA 3.39567302119051 NA NA NA 2.86319096251542 NA 2.66246985200031 NA NA NA 1.59935851719754 NA 2.71002382259398 NA NA NA NA NA NA NA NA NA NA NA NA NA NA NA 2.23216710848391 NA NA 0.121743275209131 2.56240292194579 0.113938490241595 0.909481059816442 0.985980621282861 -5.67467825483964 None

hsa-mir-2115 NA NA NA NA NA NA NA NA NA NA NA 3.93148149120348 NA 3.51561790480559 NA 0.443354020025199 NA NA NA NA NA NA NA NA NA NA 2.97465362704847 NA NA 2.47048935222336 NA NA NA NA NA 2.9024353188222 NA 0.697149655717694 NA NA 0.36896915022516 2.41931162426371 0.369650431695484 0.712314217827347 0.918721477043092 -5.67817208456723 None

hsa-mir-578 2.70601832209414 NA NA 4.04568586601909 NA NA NA NA NA 3.99086542257469 NA NA NA NA NA NA NA NA NA NA NA NA NA NA NA NA NA NA NA 3.52116782320023 3.97188099828257 NA NA NA NA NA NA 3.11192541064376 NA NA 0.0458651261871195 3.55792397380241 0.0438168021830206 0.965126115385152 0.992203913441046 -5.67961945747877 None

hsa-mir-642 3.43625107126602 3.7098373691702 2.4457186507925 3.45608345464884 4.323391669715 3.55280670932066 NA 3.52223446176907 NA 2.60225448555367 2.66307859144107 0.936632866995269 NA 4.4309588299086 NA 3.45559871836318 2.49967512892288 NA NA NA NA 3.19120953848729 NA NA 4.0452421666486 NA 5.45586683418625 3.23180312069109 NA NA NA 5.18833574894613 1.72978173544038 NA NA NA NA 3.5576683861817 NA NA -0.614913668598791 3.37172147692242 -1.03415554005177 0.302991264170175 0.756336745107844 -5.68081421874158 None

hsa-mir-612 NA 3.4856926755926 NA NA NA NA NA NA NA NA NA NA NA NA NA 3.67734061642207 NA NA NA 2.99807437373575 NA 1.84742811032602 NA NA 6.21383358253333 NA NA NA NA 4.15189508617299 NA NA NA NA NA NA NA 4.1905194002344

2.68455676937481 NA -0.430610701144836 3.656167576799 -0.452274568050564 0.651905276939225 0.898995297558389 -5.68708411173049 None
hsa-mir-33a 2.75107784122219 2.51324681209061 NA NA NA NA NA NA 5.55923031036335 3.13611510301324 3.06042199549681 3.05255201520235 NA NA NA 2.96753275814184 NA NA NA 2.94208083243189 NA 3.88433739654868 NA NA NA NA NA 4.60885285483007 NA NA 4.51704594785185 NA NA 4.02103627301822 4.18152413759376 NA NA 1.88339999696205 NA NA -0.601583892638821 3.50560387676907 -0.87593869075032 0.382764287888653 0.818464062400206 -5.69573178649325 None
hsa-mir-520a-3p NA 3.8691221003409 1.57926335962071 NA NA 3.3697898357556 NA 4.75854382020861 NA NA NA 3.1529763560617 NA NA NA NA 3.45609320715779 NA NA NA NA 2.19952067339511 NA NA 2.97979909635398 1.05109170965285 NA 2.37206767459789 NA NA NA NA NA NA NA NA NA NA 5.32377270990945 NA 0.57904774040903 3.10109459482314 0.735400064694326 0.463525187732286 0.857357456751318 -5.69903354961725 None
hsa-mir-609 NA NA NA NA NA NA NA 3.29543402534289 3.78578105020374 NA NA NA NA NA NA 2.03842889109416 NA NA NA 3.94607157643912 NA NA NA NA NA NA NA 2.78677154813266 NA 2.67430630911338 NA NA NA 2.60463947815752 3.26049361674894 NA NA NA NA NA 0.434876147731852 3.04899081190405 0.482614028081662 0.630267950479312 0.898995297558389 -5.70286025388485 None
hsa-mir-1252 1.54281667341937 NA NA 2.15511275621187 NA NA NA 4.19218434459562 NA 4.60750822802862 NA 2.33212453374322 NA NA NA 2.69945866032934 2.38419361631918 NA NA NA NA 1.72705846995817 NA NA NA NA 3.83329512895368 NA NA 2.62806834212892 NA NA NA NA NA NA NA 3.13769336866467 3.4903062243827 5.81699474511815 -0.594131454251447 3.11898577629642 -0.824734719215057 0.411125656999306 0.834709667241015 -5.70998983828475 None
hsa-mir-562 2.17545023007103 NA NA 2.02532365029741 NA NA NA 2.93615840846975 3.08539350829864 NA NA NA NA NA NA 3.75903070961082 NA NA NA 2.19622012114371 NA 1.85036009306795 NA NA NA NA NA NA NA 3.47813812641826 NA NA NA NA NA NA NA 1.50052970002537 NA NA 0.419920131478033 2.55628939415588 0.465519284560166 0.642414269086426 0.898995297558389 -5.71030413237813 None
hsa-mir-1245 2.31663579687086 3.8126019598848 NA 2.46778650102099 NA NA NA 3.76501790901091 NA NA NA NA NA NA NA NA NA NA NA NA NA NA NA NA 1.77903647116121 NA 4.56070246661792 NA NA 5.19165668625326 NA NA NA NA NA NA 2.48618808569918 NA NA -0.413885385736002 3.29745323456489 -0.450619548123594 0.653094396044002 0.898995297558389 -5.71656261017824 None
hsa-mir-1244 NA NA NA 1.38384355987412 NA NA NA 2.75016328509414 NA 3.81794914696046 NA NA NA NA NA 0.973263304721749

4.3735331805506 NA NA NA NA NA NA NA NA NA 3.17084291160175 NA NA NA 3.26281545536012 NA NA NA NA NA NA
1.78269403405126 4.34870979385283 NA -0.481515053276277 2.87375718578523 -0.551986541106028 0.58199736035863 0.89755603144822
-5.71695835121484 None
hsa-mir-520e 2.42518488455917 3.79599011526742 2.9600246499366 5.70968635959134 3.09468995377645 NA NA 5.50649772982575 NA
4.04738299036923 5.57006090161165 3.16788371464094 5.4538488082436 4.8880487177164 5.22889523654037 2.699913779899 2.90931462284981
8.11775658068584 NA 3.71190178736328 3.11162499206046 7.77115587321046 6.58270404747228 4.47307463354826 NA 4.13122584088135
2.94391939202473 3.1519086967828 6.14990965318303 7.62004159428402 5.33269461491058 9.16066519907436 4.43209278405866 4.86690850786485
3.20144077665631 5.63805324305061 5.77528193178225 1.04888334759596 3.68649057834864 NA -0.618339431655732 4.6577987217549
-1.25817954996791 0.210370591458794 0.710148198577697 -5.72185674170979 None
hsa-mir-330-3p NA 4.02115026450648 NA NA NA NA NA 4.59634653219805 NA NA NA 2.52122254854419 NA NA NA 2.36368730810773 NA
NA NA NA NA 0.143020052209948 NA NA NA NA NA 2.02445158511575 NA 2.86676324762257 4.66446575246235 NA NA
3.13251129647873 NA 4.33853255343876 NA NA NA NA 0.513977582117762 3.06721511406846 0.607095891969671 0.544939353551185
0.891941408965673 -5.72191765798142 None
hsa-mir-520d-5p+hsa-mir-527+ hsa-mir-518a-5p 3.14138143210576 3.99979591141379 2.97946245716272 1.90882235261195 4.73513587159641
4.03366349533262 1.9644138981471 4.56399421633584 NA NA 2.23886741322037 4.10107478711459 4.47805698686079 4.00147621170413
5.86563143006763 2.51353894207075 3.82083776945082 3.98247722926754 7.37154453775089 3.35419570419535 1.23725237740362 3.04129185969577
4.70903059726017 NA NA 2.52144627263588 4.50120926355387 5.77243410890392 2.88217706062052 4.42470593084619 3.21481897550803 NA
2.92145579180759 NA NA 3.48393682222191 2.41222636153281 1.0442527994013 1.50707205695399 5.27048358227724 0.573434334092317
3.57570195475854 1.24114880418825 0.216590487102508 0.710148198577697 -5.72621817566857 None
hsa-mir-934 0.94842306119134 NA 1.98407238497465 NA NA NA NA NA 4.8098129787819 NA NA NA NA NA NA NA NA NA NA NA NA
NA NA NA NA NA NA 1.64061903891565 NA 3.03449326761763 NA NA NA NA NA NA NA 3.43689915458229 2.84566728975993 NA
-0.158650212736244 2.67142673940334 -0.159299265346424 0.873709012412699 0.977027038454738 -5.72856097776813 None
hsa-mir-1261 3.75576192364656 NA NA NA NA NA NA NA NA NA NA NA NA NA NA 3.37311533090669 2.46999994381265 NA NA NA NA
NA NA NA NA NA 3.95427013265383 NA NA NA NA NA NA 4.55293085318359 NA NA NA 0.336019858717462 3.33531003918424 NA
0.154993011853853 3.11105829744357 0.154996864468202 0.877092077748688 0.977027038454738 -5.72917368996358 None
hsa-mir-610 NA NA NA 3.13635408426532 NA NA NA 2.62919264068075 NA NA NA NA NA NA NA 1.36960169605673 NA NA NA

3.16783979172099 NA 0.856222385680213 NA NA NA NA NA NA NA 1.82769328667538 NA NA NA 4.69163051352017 NA NA NA NA NA NA 0.117231657889025 2.52550491408565 0.117624239859638 0.906568002183251 0.985980621282861 -5.73379051462598 None
hsa-mir-1915 1.91845711804924 2.64965273725593 2.71477505878472 1.37534847518064 NA NA 4.72489132876507 NA 4.45256382909273 2.78297339022625 NA 2.04924620117558 NA 2.12628745203427 NA NA NA NA NA 4.90476246391348 2.84484589175017 2.52457263062309 NA NA NA NA NA NA NA 2.82397511944053 3.74594807218207 NA NA NA NA NA 5.57420383397421 3.99779315788556 3.39628231099553 NA -0.588335768388089 3.21215171007818 -0.928411600078649 0.354962640283265 0.794711743973271 -5.73429210710269 None
hsa-mir-641 NA NA 3.52274527147191 NA NA NA NA NA 3.89308562779913 2.4653234827952 NA NA NA NA NA 1.9787612912074 NA NA NA 2.63948771377327 NA 2.84183925396542 NA NA NA NA NA NA NA NA NA NA NA NA NA NA NA 1.9189068885201 3.06561781974279 NA 0.291092689999944 2.7907209186594 0.31220368217967 0.755439229701764 0.923995650122037 -5.73596803957881 None
hsa-mir-324-3p 1.93198744219913 2.21967405674035 3.3211207396971 4.41449273317995 NA 2.8563204932796 4.77148557801929 3.42178204633101 NA 2.90405864968166 5.33313798406044 2.04512882915045 3.3795570761261 3.84804841469044 NA 4.99984636589286 5.54852529561669 2.06467582961479 NA 2.32527325805183 2.08915452092208 3.94275218383886 NA 3.71928059202245 4.75187223089509 NA 2.69877239380135 2.10670986744039 5.32873778403062 1.89926970514282 4.69013454814691 4.11269636008173 5.7875144603809 6.51821541167586 4.09351504973918 2.53441742885421 4.43707097295088 3.14504444896673 6.61842235866646 NA -0.566287991217887 3.75329379120873 -1.23526339104511 0.21876563770188 0.710148198577697 -5.73678726129366 None
hsa-mir-654-5p 1.60565894003519 1.80522370563545 NA 1.19770701086138 NA NA 3.96025702816705 NA NA 4.04436805385834 NA NA NA NA NA 4.08747025146774 NA NA NA 2.5926728111491 NA 1.71336467465303 NA NA NA NA 4.26833494845823 4.00374241152334 NA 3.17968668008859 3.90376992645126 NA NA 3.49388207474494 NA NA NA 2.19295140618146 3.91420007101918 NA -0.577547552543682 3.06421933295295 -0.872135469094313 0.384813698991178 0.81993872965258 -5.73770786462659 None
hsa-mir-2053 1.54958513822651 NA NA NA NA NA NA NA 2.40375006718189 NA NA NA NA NA NA 3.05257254277596 NA NA NA NA NA 2.56156449233607 NA NA NA 3.93119479745528 NA NA NA 1.5472663213577 NA NA NA NA NA NA NA 1.05916054185818 NA NA 0.0605060444763135 2.30072770017023 0.0613468656658936 0.951188115158961 0.992203913441046 -5.73835496670103 None
hsa-mir-425 5.16783978830457 6.2065996741592 10.5515297988057 5.93962706708809 7.10203162215516 7.57596300410076 5.67211533865449 5.88986122441356 5.5812866879505 4.14592107322817 6.5596635014994 6.16585609657638 4.53377840026102 6.07086892747872 5.3725230560846 5.858436656554 6.39749889570548 5.37979458243341 6.47931686831305 4.82018750551594 6.04444532593393 5.45239180605578 5.95216212687936

6.37153413690859 6.28936696777274 5.88522754524825 5.01421402841666 5.64735744392336 4.65457492048505 5.5130543301817 3.75025981760102 8.63821952515802 5.74152701540991 NA 5.15225941955848 4.72659746654514 6.90988774367311 5.23001432675257 3.95683079253478 4.36136667412699 0.53188807198172 5.81441003031917 1.29375379690535 0.197759512357685 0.710148198577697 -5.74384855251082 None
hsa-mir-590-3p 3.48993511748506 5.83537027523203 4.1745472395768 3.81672064598088 NA 5.56533714888905 2.6138285995877 3.10684772950459 4.73257887388047 4.9054040713222 2.15250121721375 2.22244520441635 NA 2.8002107072672 NA 2.68497937401738 3.63301731744995 3.18015632818314 NA NA 3.72815957847254 1.88459784096998 NA NA NA 1.9099139200328 NA NA 2.81580893072017 2.52057405317024 4.57884603396101 NA NA 5.2228828338208 1.32106807759623 NA 3.95229169103311 4.23674094339997 1.81685797528826 NA 0.571130607109668 3.41929314340276 1.11757817293696 0.265725234877262 0.742886790958125 -5.74845386798032 None
hsa-mir-520c-3p 2.48429031475505 NA NA NA NA NA NA NA NA 4.10676358023008 3.63557944810365 NA NA NA NA 1.37017714240323 NA NA NA NA NA 1.60635000734289 NA NA NA NA NA 2.9550949392777 NA NA NA NA NA NA NA NA NA 3.69209045310025 4.66591541551178 NA -0.330660082435152 3.06453266259058 -0.360409337682381 0.719188280238884 0.919356179319592 -5.7501540620626 None
hsa-mir-1247 4.32447369507481 4.07986210738397 3.78945602837065 1.80607197133277 NA 4.17923474269694 5.12908683533303 2.86187433392056 2.92908854275798 NA 4.14077138122879 3.09797268645401 2.11680318017732 2.21295109157691 NA 2.4310138890711 4.45443796779655 NA 5.37338702743068 4.06147002229191 3.52006382844878 4.04191343519867 NA 5.59655828591206 4.83728830291537 NA 3.7649481893156 NA 2.28187287488338 5.21097202257959 NA 4.13251816440848 6.11202794233198 NA 4.3107055400056 NA NA 1.5926469337761 NA NA -0.56566328286666 3.79220263046939 -1.12680959920493 0.261800420607748 0.739612194734905 -5.75095709585978 None
hsa-mir-370 1.77570348247362 3.50191864704216 NA 4.01068119468073 NA NA NA NA NA NA 4.12358581068525 2.25217292392751 NA NA NA 3.76187360472266 3.46990091433438 NA NA NA NA 1.64885234508575 5.01171657334154 3.05411226221427 NA NA NA 0.599720531994451 NA 1.90662463369417 NA NA NA NA NA NA NA NA 4.07276300439096 NA 0.555202238384521 3.01458660989134 0.768271079653179 0.443805030230799 0.849614704748016 -5.7525391032884 None
hsa-mir-937 NA 3.73542719978006 NA NA NA NA NA NA NA 4.87779201535999 NA NA NA NA NA 3.30858100273926 NA NA NA NA NA 3.8939421783501 NA NA NA NA 4.02102051988362 3.04653765584338 NA NA NA 4.94492849954587 NA NA NA NA NA 2.5928861423618 3.46817328405204 NA 0.312685359286967 3.76547649976846 0.346489897411475 0.729589217363284 0.919356179319592 -5.75467715537098 None
hsa-mir-557 2.56358015510704 3.40577851750647 NA 4.70661081819745 NA NA NA 2.3614005274513 NA 3.92329456001743 NA NA NA NA NA

2.09228207706806 NA NA NA NA NA 2.48339310226137 NA NA NA NA NA NA NA NA NA NA NA NA NA NA NA 2.88998621248004 5.09924535269453 NA -0.315383779920689 3.28061903586485 -0.345540088138154 0.730301177270455 0.919356179319592 -5.75497905253003 None

hsa-mir-506 NA NA 0.818361932672886 3.27465885762269 NA NA NA NA NA 3.48366420220232 NA NA NA NA NA 1.37507336977947 4.09236690826163 NA NA 1.59540729562396 NA 2.04533562449993 NA NA NA 3.64274758435082 NA NA NA NA NA NA NA NA NA NA NA 2.49878984857306 3.38723587096158 NA -0.453605137735855 2.62136414945483 -0.544579610702995 0.587057730596325 0.898214641126189 -5.75541371017873 None

hsa-mir-892b NA NA NA 2.6182268731292 NA NA NA NA 5.61010914953324 5.69087589772187 NA 2.266470774157 NA NA NA 0.961183702720711 NA NA NA 2.27422343570827 NA NA NA NA NA NA NA NA NA 3.10865660634237 3.73206231686883 NA NA NA NA NA NA 0.781316897785167 3.46445723188776 NA 0.465225042274017 3.05075828858544 0.539998590950088 0.590203158085105 0.898718445265955 -5.75772600565059 None

hsa-mir-759 NA NA NA 4.17817104535826 NA NA 2.00659780502944 2.9075497814668 NA NA NA 0.845624793337871 NA 3.29676399192115 NA 3.91494945776863 1.43147397025867 NA NA NA NA 2.65864086588626 NA NA NA NA NA 3.09902324770895 NA 3.72691991219265 4.0197363309605 5.8823991979797 NA 4.3198203868286 NA NA NA 0.513039103222749 1.57914870909946 NA -0.570393705643311 2.95865723993465 -0.833968004784891 0.405896635079435 0.834709667241015 -5.76872309901361 None

hsa-mir-93 5.06760524372467 6.30131586158452 3.51974349827478 5.37960060630159 4.562811726922 5.90648719820506 5.10112998722987 4.99737564601662 5.1885110589609 4.85470200598899 4.44869596665077 6.14678052960236 5.79104034843399 5.98862527068424 6.76945384411589 4.37150509144274 4.61743709618741 5.31716325356011 NA 3.82889263753352 5.65626650507679 5.7464316488046 5.99237471181351 5.90123072871649 4.68747617372275 5.67332392463429 6.67733748201538 7.31894292021661 4.51966200211614 5.59096758636598 4.33707367369964 7.31490804419685 4.58279034920824 5.68523039423729 5.64923624111504 6.83107874093047 5.16555685648498 4.53863412793002 7.18113291171801 4.16841492746584 -0.494646820080288 5.41992171338177 -1.26701310810309 0.207134464023146 0.710148198577697 -5.77727102814567 None

hsa-mir-650 1.78619167825601 NA NA 5.24301081100238 NA NA 2.64363285616854 NA NA NA 3.51308234592424 2.53737546977128 NA 2.74762881598734 NA 2.61238364999902 2.07642871853555 NA NA NA NA 0.855694563523135 NA NA NA NA NA NA NA NA NA NA NA NA NA NA NA 4.20170029890332 2.59267415772839 NA 0.344943786487262 2.80089121507265 0.39353173554907 0.694622401569823 0.917276465053135 -5.77799514485489 None

hsa-mir-431 3.55529042651376 NA NA 5.37904177100699 NA 2.17397629669661 NA 2.77909176323603 0.954601344439106 NA 1.22235541717148 2.72575834158955 NA NA NA 2.44627496049894 3.0594619357107 1.97089114445846 NA 2.01149372672758 2.33738836883673 1.9295222104352 NA NA NA NA 4.65408076242012 4.2797898158482 NA NA NA NA NA NA 3.3511122125193 1.61486927848016 NA 3.27879904895152 3.56719968345396 NA -0.555846342795493 2.80478939521023 -0.933496082215068 0.352315061380348 0.792708888105782 -5.78401518376284 None
hsa-mir-300 1.57896545122421 4.19009089307364 NA NA NA NA NA NA NA NA NA 2.01698671878864 NA NA NA 4.39209860735719 1.73448244458271 NA NA NA 1.75547724985507 1.91168248538784 NA NA NA NA NA 2.49269257478217 NA 3.66987616304013 NA NA NA NA NA 3.15907398058804 5.63849957239087 2.90048011717645 4.83968766402523 NA -0.513408902900447 3.09846876325171 -0.689251070485198 0.491969084003314 0.863522412677361 -5.78471874005868 None
hsa-mir-596 1.80644833608625 4.57276882395673 NA NA NA NA 1.97040295917015 NA NA 3.42707224001124 NA NA NA 3.68797072074217 NA 1.79961936489913 NA NA NA 3.76648584373266 NA 4.21203786295523 NA NA NA NA NA 2.53877375119634 NA NA 3.26376267608223 NA NA 3.77018279341214 4.50201888778943 NA NA 2.77719043169781 NA NA -0.506265597389103 3.23805651474858 -0.713896915659055 0.476649258643959 0.862756303026751 -5.79069079781312 None
hsa-mir-1248 1.95972394600656 NA 3.53524559674963 3.26864404232167 NA NA NA 2.95310584348002 NA NA NA NA NA NA NA 3.80249828015205 3.02509085387028 NA NA 1.58306942520453 0.438530088234552 1.52253757278503 NA NA NA NA 4.38754463923043 NA NA 4.18606364805098 NA NA 3.45429813249957 NA NA NA NA 2.48812104722876 4.79573772402969 6.12650281428157 -0.549577245751896 3.16844757694169 -0.805447150447074 0.422097690884394 0.837252985537137 -5.79100393828542 None
hsa-mir-220a 4.76552724494766 4.1029937958422 NA 3.72439306935312 6.62057229795621 3.12478959775861 NA NA 3.90122618686427 5.23334380063636 3.69873047537648 2.41051699621933 NA NA 8.1902738417915 4.14218390965804 2.13660574820646 4.35602470853948 5.26966919153928 3.29347220823088 6.88049930688342 2.86347085033714 NA NA NA NA 5.01869649009966 7.25227618141144 NA 2.81310407505989 4.97221731615549 NA 5.07355758771242 5.0757403882937 NA NA NA 4.47113881889118 2.63301173202735 6.97707511866661 -0.580534934430036 4.57696580532531 -1.07649222297988 0.283620508186828 0.746826054308547 -5.79211833765632 None
hsa-mir-887 2.01265973588448 NA NA 2.6114624390333 NA NA NA NA 4.7544009996123 2.59899941638205 0.888037995763986 NA NA NA NA 2.68063826909454 3.73461635199048 NA NA NA NA 2.79922550307939 NA NA NA NA NA 1.9366906162381 NA 2.19157673343945 NA NA NA NA 2.00730769890484 NA NA 2.45786097380411 NA NA 0.475869867444128 2.55612306110225 0.636867842694067 0.525409350187766 0.890713562955474 -5.79303017639204 None

hsa-mir-383 3.15600879941312 4.46734243593613 NA 6.31034888401566 NA NA 3.88438658192395 2.23949373553889 4.3176485525532 3.47394746641463 NA NA NA 4.70717610779483 NA 4.21726038140258 4.44142997114186 4.96773344377355 NA 3.59384615366842 4.09215581966106 3.23646613148149 NA NA NA 4.26280606065593 2.41880013753728 3.7385517403407 NA NA NA 4.12219999279151 NA 3.44957801442186 NA NA 4.30674121342378 2.63030109216747 2.1799105246224 5.30304307699289 0.535274257577186 3.89205114424666 1.02218743289848 0.308555289048971 0.756336745107844 -5.80020567197327 None
hsa-mir-1276 0.37217548148988 3.72071883224827 NA NA NA NA NA 3.75323051857226 NA NA NA NA NA NA 4.04124184941623 NA NA NA NA NA 3.16497605472814 NA NA NA NA NA 2.75593651814351 NA NA NA NA NA NA 3.92298975170204 NA NA 2.54097481299384 NA NA -0.124377613960223 3.03403047741177 -0.13533260069458 0.892580536080546 0.985762020616427 -5.80142771746667 None
hsa-mir-188-3p 3.44085679783057 NA 2.0496880911877 NA NA NA NA NA NA 2.73463460234053 NA NA NA NA NA 2.95339147703203 2.01659304806839 NA NA 2.80121415417169 2.23853901558111 1.73826338762998 NA NA NA NA NA NA NA NA NA NA NA NA NA NA NA NA 4.2668717237027 NA -0.0818283471994445 2.69333914417163 -0.0905835482010568 0.927976480139453 0.985980621282861 -5.80607617399822 None
hsa-mir-636 4.86022002019695 2.95525435276753 NA 1.55483726426689 NA NA NA 4.53761295034727 NA NA NA NA NA NA NA NA NA NA NA 1.44629450443289 NA NA NA NA NA NA NA NA NA 2.14240835322217 5.7016394840683 NA NA 3.71503621382298 3.27346092353979 NA 4.03346040594807 1.90868829843712 4.00979962293106 NA -0.469798081879049 3.34489269949842 -0.612526483380749 0.541334478961897 0.891870739929026 -5.8073600319269 None
hsa-mir-153 1.76081615345568 3.85952607507165 NA NA NA NA NA NA NA NA 1.17692973666643 NA NA NA NA 3.65037981016799 3.41857693168199 NA NA 2.94648675418759 NA 1.30796743918149 NA NA NA NA NA NA NA NA NA NA NA NA 3.43469830259394 NA NA 3.75985401028416 NA NA -0.0320540071479742 2.81280391258788 -0.0350851095522583 0.97207092181503 0.992203913441046 -5.80928291561007 None
hsa-mir-146b-5p 6.72354556733343 4.32001523692262 3.39256576287449 5.03929021729204 5.53842877004375 4.91005657011081 5.17892218062815 5.22882400980568 5.07037941844602 3.99354891399252 5.19893368777642 5.05680077097726 NA 4.94291575752693 6.63903611340567 4.45393389611996 5.74356537785429 6.89792192464101 4.15279938839435 5.22557574765183 4.71281633757262 6.29691405505928 6.92801090444986 6.98536549505778 3.85585462679073 7.08411033364159 6.29610765051925 5.77571682422986 6.41330241542366 5.1044652190863 5.39374553288977 6.7062778359825 5.56465328917153 3.95678673593559 6.35790429412292 3.97210369897794 5.56301178614392 5.59674441527128 5.11456589385146 4.89857305958954 -0.486374714304305 5.39189973629653 -1.23942186982966 0.217144894294613 0.710148198577697 -5.81104068652602

None
hsa-mir-885-5p 3.36020512371448 NA 4.43312617234899 NA 3.91266191325198 5.00812694719372 4.73154108811494 4.47649540450126 NA
3.48331628029856 3.89890702817644 3.52601142010112 3.46052218551991 3.69143299520771 NA 2.70956092994364 3.26988296785435
3.20526005643997 4.23286111961061 2.96182239950745 4.84317091773858 3.61444366996805 NA 3.76836658436785 NA 3.61724828713709
5.25678932347304 6.2435878839992 4.24689324387665 1.78523255962129 2.85595099149921 3.54164050676794 4.63921151112361 NA NA
5.75932391595772 NA 2.11933767289424 4.20606941804338 7.99513793829094 -0.526885251330682 4.0275528534369 -1.14440008894601
0.254404030096727 0.731348500441128 -5.81244236797433 None
hsa-mir-2114 1.77724445934887 2.79427999604661 1.99379051050206 2.57769710942296 4.1302719899775 NA NA NA 2.7021893536963
4.76184914367134 NA NA NA NA NA 2.97562954563741 3.12297900511548 NA NA 3.09558432322709 1.2285542396832 3.3584890145018 NA
NA NA 4.42003128843014 4.35382891816897 2.69366840379158 NA 3.36255559527053 NA NA NA 4.84786493603555 2.17161826380044 NA
4.88388535233424 0.2667966463665 3.7527525588588 7.30696541011269 -0.560766008614975 3.299023912 -0.982981863415777
0.327421875724766 0.770661199795522 -5.81442638831836 None
hsa-mir-137 4.64490401913325 4.80359012111072 NA 5.40830882346785 NA 3.78275725329874 NA NA 6.81219761869028 4.85283001941177
6.50373365199109 NA 1.45489882767862 2.90046051976135 NA 3.58835852116228 5.3419061298335 NA NA 3.09032606016973 NA
1.93344319630446 5.09457439663188 2.38364389721226 NA 3.49758981915883 NA 3.27998115568149 3.39027900981355 4.68124746307488
4.94845188236741 3.27688214674991 NA NA 4.76181880751748 NA NA 4.2944466843661 5.0689099816491 NA 0.54775026043182
4.15814750025986 1.02822983524245 0.30570151766545 0.756336745107844 -5.8150166618079 None
hsa-mir-509-5p 3.77923273735073 1.54525053515433 2.86323451405719 4.0633675480728 NA NA NA NA NA 2.98885443075859 NA NA NA
3.0487717156426 NA 1.2191995354436 NA NA NA NA NA 2.40842715892673 NA NA NA NA 4.08479821153969 2.39628010666339 NA NA
NA NA NA NA NA NA 4.52044567969158 3.08388210945219 3.05297339326953 NA -0.470956678998161 3.00420905200177
-0.664416036868682 0.507673118201846 0.874648257930609 -5.8229970548073 None
hsa-mir-133a (+++ see message below) 8.46638263397796 7.86951093129941 10.1376924597863 9.43372325514444 9.0763541588552 10.8797643585437
9.2359541058909 7.66865087992806 8.53850673387379 9.06145343726789 8.98160258181633 10.0146768637998 9.68241068492745 8.40531739872698
10.9108612888388 8.00858282265591 9.52528405156665 8.68556549839008 11.8042570895335 9.15277136977715 10.339143186102 8.09296154560313
7.84167498939665 9.39463440809564 10.5953168980026 9.45212405012459 11.8606158935504 11.4882014354165 10.8394618960592 9.06772411639087
9.52666573296271 8.55993442631755 8.83829538247522 10.6183800046656 8.54587011593162 9.22720940881162 11.7239298297252 8.3841094137991

9.83548970083742 11.235244244249 -0.496383203695817 9.52515773207792 -1.23971910003619 0.217022152549749 0.710148198577697 -5.82304831802457 None
hsa-mir-1179 3.11477076371331 3.66252842745168 NA NA NA 3.03451803100538 NA 4.23818672052173 NA NA NA NA NA NA NA 3.01569633356667 4.45472788679222 NA NA NA NA 4.4609645734767 5.2586450725286 NA NA NA NA 4.69497941928784 3.00075183826253 2.78733777354212 NA NA NA NA 3.95850288704363 NA NA NA NA NA -0.440125566848407 3.80680081059937 -0.601601050060252 0.548560885849409 0.894005984235659 -5.82653769138143 None
hsa-mir-576-5p 1.35300993699965 4.32781177652464 NA 3.46554636188621 NA NA NA NA NA 2.9786074423856 NA NA NA NA NA 3.83045385353134 NA NA NA NA NA NA NA NA NA 5.81275894653272 NA NA NA NA NA NA NA 4.4230885801875 3.04058925357978 NA NA 0.390577394893225 NA NA -0.225667669532818 3.29138261628007 -0.252967344408509 0.800731870084716 0.941293012855703 -5.82842845454663 None
hsa-mir-1911 0.4556998331796 4.61220401798233 3.04257838883204 2.21974432878995 NA NA NA NA NA 3.145064203133 NA 1.87469318639142 NA NA NA 2.69852217887661 4.39687381855094 NA NA NA NA 3.54880542464704 NA NA NA NA 4.81500593685564 NA NA 2.2918310766694 4.14068893981111 NA NA NA NA NA NA NA 1.52079050594917 NA -0.457751882319486 2.98173091074371 -0.615821759112636 0.539154975918322 0.890713562955474 -5.83000420285681 None
hsa-mir-320a 3.98179121146838 3.61921643190503 2.54647201478961 3.01515343081609 3.94715929867931 3.46674688929232 3.57960113511607 3.88685653933178 3.47278713522128 4.80636345123998 3.27772350649939 5.78552830398608 NA 4.88235283250455 NA 4.47995502858741 5.73806977141843 4.67697757097332 NA 4.56571047727335 3.44969295782013 5.11363045571873 NA 3.91196716110911 5.18163768650618 6.28564656459369 4.75737082012026 5.69517230017228 4.68642977632205 4.09498406972813 6.3808403595804 NA 4.97178828644242 3.71542296634142 3.11959960132467 4.06600619347385 4.39846241633489 3.22274931089463 5.03775489707539 NA -0.491805340850344 4.34757708390178 -1.16680188628458 0.245226120068966 0.728430297544761 -5.83144602811273 None
hsa-mir-873 2.20958052786175 NA NA NA NA NA NA NA 4.23736387976853 1.94254292605786 NA NA NA NA NA 3.48010884407735 NA NA NA 0.749733570664364 NA 0.81505665496191 NA NA NA NA NA NA NA 2.13422943276743 NA NA NA NA 4.21231453039912 NA 4.13058034422271 3.04262447103533 NA NA -0.34309513699133 2.69541351818164 -0.415269127913267 0.67868974811079 0.911469750803578 -5.83188689627606 None
hsa-mir-1263 3.6457232800103 2.15679090358673 NA NA NA NA NA 1.59098513824222 NA NA NA NA NA NA NA 0.8184098982038 NA NA NA 2.22404548486584 NA 1.47316902609133 NA NA NA NA NA NA NA 1.81489489346822 NA NA NA NA NA NA NA 1.85842880046659

3.97002044080014 NA -0.191937349224792 2.17249642952613 -0.22218361595266 0.824553517111223 0.948410748216205
-5.83521026960854 None
hsa-mir-635 0.893233160658489 5.90134912035661 4.1328105994416 3.80570020856911 4.13948356903371 5.65953547395481 2.51239735394319
3.8965842267352 1.7949721382271 5.26381918049928 1.91997936687911 1.95565844873514 1.96905409970058 NA 4.47560406975188
2.73408782297102 3.49675267215402 4.26451489059978 NA 3.59966278542172 2.53038748425936 4.48457999762052 3.08899937879137 NA
3.94802258978033 4.20272569487586 4.35822082101215 3.9498341307651 NA 5.4146203836887 5.3461985934024 NA NA 5.55747105347067
2.32182986010797 NA NA 3.51784314837607 3.169650519326 NA -0.524056907518618 3.68727686590674 -1.11022293265942
0.268799860591041 0.742886790958125 -5.83798334019814 None
hsa-mir-1255a 2.03057588595223 4.33900778592041 NA NA NA NA NA 2.97151804062541 3.49624692361276 NA NA 3.47469015524976 NA NA NA
NA 3.87613551884025 NA NA NA NA 3.21821048845483 NA NA NA NA NA NA NA NA 2.86767129446305 NA NA NA 3.03728676948726
NA NA 3.25941293973468 NA NA 0.26905034533185 3.25707558023406 0.329192974453087 0.742586232290983 0.923995650122037
-5.84307234652275 None
hsa-mir-1299 2.6857262638949 3.22973471655336 NA NA NA NA NA NA NA NA 3.35951195083294 2.33242851220238 NA NA NA
3.12885466203565 NA NA NA NA 1.98073548392765 NA NA NA NA NA NA 2.32224321708999 NA 2.32069901808066 NA NA NA NA NA
NA NA 3.3180232649263 3.2886461890092 NA 0.301181786497086 2.7966603278553 0.377116282719589 0.706756224467989
0.917276465053135 -5.84601599840741 None
hsa-mir-661 1.40070856062415 4.87320101618964 NA NA NA NA NA 4.3414398246013 3.72598364246809 NA NA NA NA NA NA
4.0298109297285 NA NA NA NA NA 3.29956383520906 NA NA 4.92450256806655 NA NA NA 4.57490053534698 NA NA NA NA NA NA
NA NA 4.07584727963503 2.95182475022266 NA -0.29109899897372 3.8197782942092 -0.357334071717062 0.721472173000771
0.919356179319592 -5.85280765202727 None
hsa-mir-1291 NA NA NA 1.70853883132595 NA NA NA NA NA 4.26020031070695 NA NA NA 3.31813067686626 NA 2.47026030627844 NA NA
NA NA NA 1.18789525996137 NA NA NA NA 3.12251648234296 NA NA NA NA NA NA 4.23917145015463 NA NA NA 2.92985275358345
3.52948651198828 NA -0.0625019603117374 2.9740058425787 -0.0722533689506406 0.942522114312361 0.992203913441046
-5.85568371982966 None
hsa-mir-518c 3.90813742295169 2.93069410864995 NA NA NA NA NA NA NA NA NA 1.20994867136758 NA NA NA 3.54551564503529 NA NA
NA 1.21511605722015 NA 0.535630035162237 NA NA 4.79425685968278 NA NA NA NA NA 3.78127401612224 NA NA 3.97092894357119

NA NA NA 1.18222989474927 NA NA -0.290981568812612 2.70737316545124 -0.347969060830842 0.72847577059289 0.919356179319592
-5.85589555381851 None
hsa-mir-1180 0.279654110333887 NA NA 0.66725027922186 NA NA 3.76000927436695 4.9359871995396 3.50103343161819 NA 5.0124484872592
2.73769341555567 NA NA NA 2.12771296633267 3.47683940470114 NA NA NA NA 4.24165948557503 NA NA NA NA NA NA NA
2.15801951031108 NA NA NA NA NA NA NA 2.76899536506201 NA NA -0.111932723768356 2.97227524415644 -0.126777465881783
0.899326150135977 0.985980621282861 -5.85636152526726 None
hsa-mir-449b 4.98634344250355 5.15011388586415 NA 4.18071205911068 NA NA NA NA NA 7.0095083074295 NA 3.37588105115559 NA NA NA
4.23980217563762 NA NA NA 2.08104156328101 NA 2.24763732682473 NA NA 3.43699478561758 NA 4.61013115475414 NA NA NA NA NA
NA NA NA NA 6.43682684204684 3.42110202402648 NA NA 0.401376214057774 4.26467455152099 0.518268573076159 0.605213648963684
0.898995297558389 -5.85761263837731 None
hsa-mir-643 0.881382687582664 NA NA 1.38675882439006 NA NA NA 5.24625563150594 NA NA NA NA NA NA NA 3.4654293661383 NA
NA NA 3.7954545893911 NA 1.90313504191251 NA NA NA NA NA 3.50286683369514 NA NA NA NA NA NA NA NA NA
1.54773849186218 4.88497589286161 NA -0.00462284528124757 2.95711081770439 -0.00519655266484587 0.995862509864406 0.995862509864406
-5.85809290943869 None
hsa-mir-569 1.52544105956645 NA NA 3.91288584824973 NA 1.64736772507541 NA NA NA 2.96016468822907 NA NA 4.29039038023196
2.85911098546858 5.39223086010061 2.62310257686851 NA NA NA NA NA 2.954418968371 NA NA NA NA NA 4.00358688258182 NA NA
4.11773690266915 NA NA NA 2.67084074112815 NA 4.77947083425004 3.15489032342858 3.6527768874578 NA -0.467766311652858
3.36962771091179 -0.706392753003524 0.481264115275925 0.862756303026751 -5.86242358014559 None
hsa-mir-342-5p NA NA NA 2.00705155076725 NA 2.81337774309272 2.19541625400345 NA NA NA NA NA NA 2.54465918622667 NA
1.87454275193927 NA NA NA NA NA 1.50597099567657 NA NA NA NA NA NA NA NA NA 3.36807071315831 NA NA 1.74181352760405
NA NA 1.96838406760612 4.11215772666573 NA -0.252269908936284 2.41314445167401 -0.312975052098898 0.754845309389086
0.923995650122037 -5.8667097858605 None
hsa-mir-421 3.59654026457011 4.16298089665884 NA NA NA NA NA NA NA NA NA 3.21926349033945 NA NA 4.27115444496604
2.30695710022219 NA NA NA 0.477332811840812 NA 2.58198750877836 NA NA NA NA NA NA 2.03335625393409 NA 2.25543194188495
NA 4.3803123177423 5.03082396357616 NA NA NA NA 4.07455920170642 NA -0.387040363170807 3.19922501635164 -0.514281108816432
0.60798985645049 0.898995297558389 -5.87256053387908 None

hsa-mir-613 NA NA NA 1.33687243333621 NA NA NA NA NA NA NA NA NA NA NA 2.69510495004592 2.95179736692051 3.53649042281999 NA NA NA 1.91426412027518 NA NA NA NA NA NA NA 1.35107731840767 NA NA NA 4.46390325538469 0.292023909003383 NA 4.00132252009096 1.86753291015837 2.78106262737886 NA 0.24846819889507 2.47195016671107 0.304716217424963 0.76110807903711 0.92518658980654 -5.87741080068594 None
hsa-mir-1282 NA NA NA 5.56957213581589 NA NA NA NA NA 4.47075526482806 NA 1.83973953581723 NA NA NA 2.84388170803089 NA NA NA 1.20471870967746 NA 2.87980948622085 NA NA NA NA 5.21346397748549 3.14499198373681 NA 3.98558862277717 3.49861978133614 NA NA NA 2.35401723383989 NA NA NA 3.68658159141591 NA -0.351848340139273 3.39097833591515 -0.460903381862709 0.645692081869948 0.898995297558389 -5.88411427085906 None
hsa-mir-542-5p NA 3.23575726954786 NA 3.37156253685027 NA NA NA NA NA NA 3.12419769326336 NA NA NA NA 2.52992741395054 NA NA NA NA NA 2.0497860168364 NA 1.95422939850932 NA NA 2.40949396232 NA 4.33841103301197 1.82860771360009 NA NA NA NA NA NA 4.42867826379014 3.0409955077122 2.31781840809651 NA 0.269358690418428 2.88578876812405 0.345962672069076 0.729969249537035 0.919356179319592 -5.88632403046996 None
hsa-mir-602 4.91127992244582 4.84700551735063 NA 5.31342553366958 NA 2.08669103522504 NA NA 6.28865469547962 NA 3.03764575275714 2.57500806911332 NA NA NA 3.38077878539894 2.87634569828497 NA NA 4.05696873986923 NA NA NA NA 5.18154909843684 5.11021643610688 NA 4.1953109175243 NA NA NA NA NA NA NA NA 4.97906152877257 4.74389292591929 2.11387068619119 NA -0.449936557199085 4.10610658390909 -0.670564892520416 0.503730189979601 0.872842829188792 -5.88805208683706 None
hsa-mir-1292 NA 4.55136357552598 NA 2.7814387116708 NA NA NA NA NA 3.03276692139678 NA 2.76396056125309 NA NA NA 2.78445178990128 NA NA NA NA NA 3.17116512473746 NA NA NA NA 5.22644936944142 NA NA 3.69328726124555 2.39825911044734 NA NA NA NA NA NA 2.32288201659339 NA NA -0.179612264543445 3.27260244422131 -0.221563473345007 0.825031984334934 0.948410748216205 -5.88956354324825 None
hsa-mir-455-3p 5.69946089105323 5.65890240141609 6.4357816940825 3.9399155408753 4.78023609355293 7.23158553713639 5.7882033291569 4.73502064715321 5.16409454647165 6.48647781898847 3.41789107809192 5.66202372632619 4.60599173325715 3.72080454460186 5.69186765158321 5.92595304178429 4.97436977366303 4.59226608489197 5.73321461008208 4.91515910610204 3.08872461830823 5.24415049011917 5.68138035624466 3.72150576845497 6.03183668126948 8.48267830894114 5.5396236531828 6.14653707465238 5.70112140702306 4.77850400860399 7.80370906549427 7.25678671489973 5.53621491256698 6.07386752725082 5.87561105277939 4.77018631589251 6.70159097833454 2.73953395233977 6.5186639544662 7.06685062060121 -0.479992880557742 5.49795743279239 -1.18357134386641 0.238461724691444 0.721791040320554 -5.88957041218303

None
hsa-mir-517c+hsa-mir-519a 4.26588421179856 NA 3.91567764681596 3.70956188613836 NA NA 2.4414922881637 6.01002575113591 NA
5.11094814601086 NA 1.77157341726135 NA 3.51796540108682 NA NA 2.59897524173093 NA NA NA NA 1.02201439403347 NA NA NA NA
4.82041417588345 NA NA 3.35115860502434 5.64463970643655 NA NA NA NA 5.03211518675184 NA 4.86757422006315 NA 4.4491450456298
-0.464901969419147 3.90807283274782 -0.701498382082544 0.484291152297617 0.862756303026751 -5.89046515080714 None
hsa-mir-10a 4.98325944908884 7.82654510845258 11.1052615331358 10.1903694913268 6.7261347166241 9.55630419019674 13.6066110248114
12.8924914014599 10.5310441516231 5.89697524845131 8.24964872445508 5.64572323188761 5.1836499466607 9.65089569886748 9.48850920849249
9.37716287701596 6.90633100330728 6.4161116974388 9.02401561935961 10.390658890485 8.03489055214686 10.3938449308639 8.48509337399233
7.99968945162704 7.93132806006368 7.65132278649876 9.13291814394489 6.83412114728232 9.31553590168752 7.40162922910296 6.63878083697847
4.32242401016127 9.38003232906933 6.61262483381589 9.81600506438663 10.7310769097486 7.50853401476867 8.25730619246032 9.57881594985227
6.21765200388955 0.570203874539967 8.39728322338705 1.17815028316509 0.240609002987398 0.721827008962193 -5.89583307888837
None
hsa-mir-1206 1.28536204558974 4.18827991815749 2.84541917505954 3.92182131167667 NA NA 4.50225076979436 1.69912726348915 4.01854120978507
5.06076037916237 2.8944681520172 2.83438720651886 1.95480378807947 4.21565393317606 NA 3.99965861363061 4.28067259686901
4.80904871381872 NA 1.29308012360985 1.79406427510937 3.60226679365855 2.23907937782993 NA NA 4.89585217520422 4.3563477456095
3.54913101391437 NA NA 3.84049208589826 NA NA 4.42386810836427 2.57318469423044 NA 3.72481870095594 4.9289058321207
5.05018986205675 5.09218430573629 -0.488859624641218 3.58185241969389 -1.02368728664481 0.3077680636163 0.756336745107844
-5.90365729192769 None
hsa-mir-450b-5p 2.30202200827573 4.11562929642886 3.42097397524134 3.0297277953052 NA 1.84078319735184 5.24455039623368 4.81064292147763
4.63634008555404 2.70221920991532 1.26018675554084 4.52715722142494 8.37806857892294 3.07248159308268 NA 0.616699733135962
2.90453880840097 NA NA 4.45278514792769 NA 5.53228390233648 NA 3.72361915324436 NA NA 3.84463973147405 4.33765685939195
2.88865107560404 2.61780113233889 3.96367770792872 3.6794531290823 3.18564742606055 4.86176772453763 NA 4.092523735726 NA
4.84534901927317 5.05855136037822 4.6281147121746 -0.507805770418484 3.81915144645902 -1.03931985693525 0.300454423428532
0.756336745107844 -5.90701578805475 None
hsa-mir-616 2.07178037930237 NA NA 2.77046559880534 NA NA NA 2.00016410211938 2.69006785603682 4.28332296666804 NA NA NA NA NA
NA NA NA NA NA NA 1.95948366561469 NA NA NA NA 4.39339364249114 NA NA NA NA 2.72338496425841 NA NA 2.71717911062698

NA NA 1.68446977362215 NA NA 0.0675779492637165 2.72937120595453 0.0834037113710049 0.933670124271925 0.98946060621436 -5.90927820206064 None
hsa-mir-769-5p 2.48436739697989 2.77956714976161 NA 3.45807148466261 NA NA NA NA 4.28258011794134 5.11372871505328 NA NA NA 3.42152714365257 NA 4.15866577701574 NA NA NA NA NA 3.1181660417894 NA NA NA NA NA 3.36722527289017 NA NA NA NA NA 5.47639783090946 NA NA NA 2.27222135659896 NA NA 0.112712772319722 3.630228935205 0.139978101885605 0.888911052663527 0.98351076102038 -5.91172147243734 None
hsa-mir-649 2.93829856580526 NA NA 3.09877761026024 NA NA NA 6.24702960273453 NA NA NA NA NA NA NA 0.460320794989205 NA NA NA 2.67593226641035 NA 1.06374322235763 NA NA NA NA NA 1.58930204208681 NA NA 4.98904625648508 NA NA NA 3.1198897048746 NA NA 4.71713920875764 1.59218506209898 NA 0.238854185263126 2.9537876669873 0.292006437955843 0.77078396112896 0.92518658980654 -5.91291260892058 None
hsa-mir-203 3.80591243175774 6.84389699514189 3.81282746482832 3.82338335455741 NA 5.41247247554251 7.76420208337787 2.89795145066258 4.75551308970494 6.98475736336021 NA 8.04985993293955 4.89367288666803 6.42724514554298 2.63789065665048 7.41644659012981 9.46219936086631 NA 5.75175514316425 4.7061457194346 3.80015446659036 3.84891520429844 4.66211700371831 3.16887992626976 NA 4.90375096093955 5.72546016852173 4.60598296504685 NA 4.99662655554481 3.75795280061504 3.94844603488371 5.23445609578248 6.99802123613939 NA 4.23175999511969 NA 5.92913395464636 10.3029803809438 NA 0.540169178160461 5.36127405916843 1.06113331439646 0.290437506446707 0.750504307264223 -5.91559358133796 None
hsa-mir-183 5.91411741283159 7.51985119678881 6.28952896005166 4.44471473249618 2.35995144883339 3.9070179610491 4.6018963375409 8.38595454800655 4.20591641796298 4.94470332319676 5.11935331640169 4.71794954786675 8.05738217589269 6.68313078499086 NA 5.68486281943757 7.73417935563985 NA 2.83253695272796 7.7927547273112 4.7225565299734 1.8181291460862 3.01486693219965 4.79966753579009 NA 3.99242901050867 4.41927621446684 6.87261545667687 4.15991342896087 5.51316147270427 4.3246586952325 NA 6.07903505519269 5.36344653678767 NA 7.43079359658197 NA 6.46169678596007 6.3110132603721 6.36597966583433 0.518911543349018 5.37779533359867 1.08727389898523 0.278739026934293 0.745003004575917 -5.91725382702611 None
hsa-mir-494 (+++ see message below) 6.00223631449694 5.88704396766183 5.35457015214957 5.88199046376599 5.99868671479211 7.15683058692691 6.9020840682056 7.30495949201226 5.93429694099981 8.73579486164863 8.09300578969396 2.92106565090221 8.24875766416846 6.68238344375582 8.69312769894355 6.83680242728837 6.24032229433902 7.94453513432371 6.78392422645732 6.39849147878958 6.13925966861615 7.22847885428997 6.61054761232596 6.84585397434934 7.77693034440735 9.33532232794391 7.17346946266526 9.09990396517861 5.77352846681604 8.82081560699807

6.70113838826444 8.21637149722923 4.45640939765256 6.84814957531116 7.14689658845686 6.32255715476508 7.79816730708531 4.91350107826662 9.20334544055251 7.09570305862229 -0.475272019923754 6.93768147852796 -1.14783508381953 0.252870458842575 0.731348500441128 -5.93033441926885 None
hsa-mir-656 2.34298423711176 3.73201812510658 1.90602723439874 3.40797439747352 3.39747028148392 5.30181579265443 2.95702874617813 3.17277278591202 2.11737768007277 2.8023850396044 4.00089539762892 4.11882391868225 2.64556894524684 4.47646065779839 4.41686985293676 4.57928661465397 4.85285903061137 4.15033726581995 NA 4.3446851929378 2.1133062536443 4.1242105498234 5.09931972176779 2.87642281945389 NA NA 5.42337252472137 3.81817064092787 NA 3.43908711145258 4.5571151834731 3.76361880659661 4.07861138596876 3.55208804005469 4.35529598521966 2.97279826449981 NA 4.92772533799264 4.92586810136162 5.07458874512295 -0.451816219830724 3.82357830481124 -1.0833515016104 0.280457184143836 0.745003004575917 -5.93336552933637 None
hsa-mir-515-5p 3.31643364962293 3.59700806655905 NA 2.72252875053357 NA NA NA 4.85504525715857 NA 5.19043081718679 NA NA NA NA NA 3.64876456248934 2.7948162036074 NA NA NA NA 4.10551461727659 NA NA NA NA NA NA NA 4.35202699850199 5.0589354027573 4.9525843904678 NA NA NA NA NA 2.12221317444535 3.73953683122756 NA -0.322988477614054 3.88121836321802 -0.454887954077352 0.649995170607462 0.898995297558389 -5.93423606216556 None
hsa-mir-545 NA NA NA NA NA NA 3.30775187251438 3.35501849900416 NA NA NA 3.46029248516934 NA NA NA 3.36470012804571 NA NA NA 2.2968785006152 NA 1.25526767045299 NA NA NA NA 4.93352166926008 3.2937612732725 NA 2.50017197171182 NA NA NA NA 2.17708613158146 NA NA 3.02828476016787 3.23577769586402 NA 0.239232415311081 3.01737605480496 0.320822920628005 0.748895882518723 0.923995650122037 -5.93581677856951 None
hsa-mir-2117 3.26613086057275 4.38417687044381 5.09980649448026 3.33161428967338 5.54918776919403 4.74319936909861 5.6350880784659 3.84248921086923 NA 3.85190288676878 2.78419670927678 2.04135064983669 4.60720137667953 NA 3.70627697523073 3.56582948333357 3.99946221858526 3.67277458941801 NA 2.6720411529426 3.62613501581649 4.11137821876967 2.76475277457147 NA 5.48425852055191 5.51764593097554 3.69380945832597 4.54553472591393 NA NA 3.80888039997124 4.67057998040077 3.0540054181904 6.09372892058057 4.57465946528427 3.12454901013384 5.05484532551641 4.6829866734076 4.02557813815093 5.53082272203023 -0.44773068904243 4.15049646127827 -1.05656700871392 0.2924850948708 0.750504307264223 -5.9510483980123 None
hsa-mir-889 1.70854628962691 3.05497295189196 NA NA NA NA NA 4.33331303463901 3.66952044211624 3.2488257680972 2.52799446595438 3.16910184196746 NA 3.34395482067228 NA 3.29272632996941 NA 3.37462352054208 NA NA 2.83153646281954 1.97148755991775 NA NA NA NA NA 3.06872811481417 NA 4.86872888155657 NA NA NA NA 4.50468479215965 NA NA NA NA NA -0.276675215705842

3.26458301844964 -0.400148599452232 0.6897329810922 0.917276465053135 -5.9710969932968 None
hsa-mir-16 (+++ see message below) 10.3932747593903 10.0009648870561 10.8157093102714 12.7569231964065 10.9144880799394 10.4931375510169 11.7416161482079 12.8219440522961 13.3485595339783 9.53489023258964 10.4904733363024 11.3580503131373 11.964727762155 11.3793100510138 10.2768183828709 11.6417104778988 13.1710950764975 12.4775041238571 11.2574429921603 12.0842959449734 9.67082424652905 11.3765405031926 10.3898465368792 11.7579959073823 13.960992496278 12.8641104940398 10.5999145593927 11.4269621442078 12.4221186971567 12.2546245447459 12.2178635036069 14.9692479509137 9.36110285034571 12.7787721485944 10.9368216575204 13.6380799094626 12.9690826302597 9.50054585463931 13.2759885297151 11.7380273793016 -0.459326316607225 11.6758099689046 -1.11067477174295 0.268491870737396 0.742886790958125 -5.97142067546759 None
hsa-mir-302f NA NA NA 3.88824822210334 NA NA NA 0.746314555012347 NA 4.6123296280099 NA NA NA NA NA 2.50937064097048 1.72064253425153 NA NA NA NA 0.36515681156638 NA NA NA NA 3.80518102072478 NA NA NA NA NA NA 3.48664364124207 2.74287323160477 3.48519912453291 NA 2.70766202678894 1.62266198560031 NA 0.0931842814894965 2.64102361853398 0.121866877424839 0.903205828345281 0.985980621282861 -5.97743761364956 None
hsa-mir-2276 2.93689254732054 3.98025936436628 3.22778764379734 4.0440381323212 4.11372511413196 NA 3.70447755562475 3.88712892001879 NA 3.71966157828207 1.25756866958171 3.48979070676256 4.96455532146152 3.51353934172952 NA 3.65710156828504 3.10796631965671 NA 6.00553239601543 1.96700266452204 NA 5.47040006495406 NA 2.06253826503366 NA NA NA 1.68942910441174 NA 5.06399033641785 4.04625767538678 NA NA NA 5.68952858575534 3.80488176815871 NA 2.61152847390346 4.09928794117367 5.94560823305425 -0.449780804582611 3.7715568573895 -0.862716511151335 0.389818046625731 0.824772919702862 -5.97871367591111None
hsa-mir-556-5p 0.714252776029567 NA NA 2.91924990592904 NA NA NA 0.40061384902251 4.08486443627148 NA NA NA NA NA 5.00228068757038 3.65812076109819 3.9587675179565 NA NA NA NA 1.25255319540112 NA NA NA NA NA 3.74822889318987 NA 4.18655492018151 NA NA NA NA NA NA NA 2.64420798658532 2.89048228623021 NA 0.018187391379203 2.95501476795547 0.0235160098863794 0.981277255041807 0.992203913441046 -5.98419750840605 None
hsa-mir-802 3.51779246457775 NA NA 5.58282808197236 NA NA NA NA 3.20274699717212 4.84558928051684 NA NA NA NA NA 0.37005246889562 2.87506565123801 NA NA 2.40377210159624 2.39681734062056 1.76545687913524 NA NA NA 3.5404096863852 4.5492444821284 1.35648269401748 NA 1.73037493923455 NA NA NA 6.18753841566185 1.45860814313447 NA 4.83841896179279 0.856546466022403 NA NA 0.388845491467982 3.02810265024129 0.579214361030579 0.563474794651112 0.895722046824318 -5.9864660474083 None

hsa-mir-376b 2.36426886417381 4.90153726179817 3.54195010763854 3.76706606367398 4.96031576048882 NA NA 3.58867484358645 4.2147868785784 4.01116023611036 3.10713682324942 2.0151218697855 NA 2.5784849443143 NA 0.964468400463451 0.958810950730465 NA NA 4.18345359130792 2.72841895284429 3.4599355521704 NA NA NA 4.02047086851835 NA 4.89613865111755 NA NA 2.77054500290017 NA 3.57652692849256 NA 4.76683447652409 NA NA 1.2256810897405 5.47026237026513 NA -0.431684643912113 3.39443697775968 -0.777355712010941 0.438334947251273 0.848349147761226 -5.99153257087185 None
hsa-mir-146a 5.8417715253571 6.53050711699628 7.64275279980315 11.1630915395578 5.06641977558113 8.16332832834324 7.70461262110042 4.78399890355867 10.1384127835352 9.36538090375345 9.59365624844451 4.40827189427273 6.33399774344376 3.24593854824198 7.20874943245845 7.2414282119777 10.763468666044 4.79898574094742 4.49392669756453 10.1108299308421 5.74065003489767 7.28475856872033 6.05181175840715 6.22706046702276 6.6527688956754 5.64997704409666 7.65094713287961 6.57753135967087 5.17117944067332 7.74329019756478 7.53798286856271 5.32444416549826 9.84686418621957 5.71230198612552 6.3847549472753 7.5120650194151 6.48361800070084 6.38041605141726 7.30817584014605 7.30673101286854 0.502610021699295 6.97867145974153 1.08974130997195 0.277580870865641 0.745003004575917 -5.99398030428957 None
hsa-mir-379 4.34915729530168 4.23478864434494 2.65974031375385 4.57988746784406 NA 5.18847785854533 3.02189038478782 6.19193078014657 4.93190593307816 4.60966782301644 3.72570986152661 3.01010017474578 3.03584347501641 3.19569499325147 6.363814200713 4.55452243679319 4.49874272239984 3.85258206799212 NA 4.72882035033662 3.55510951689385 3.17802308101311 5.13217397375368 2.65612862543697 NA 3.65698884062741 4.73880814675986 5.19395595355649 NA 1.94068101147036 NA 4.37829857665231 4.80112301679215 6.53760091690704 3.98851952535693 4.07501045691421 1.37930584409303 0.196444567765574 5.51143391845539 NA 0.455484448032751 4.04861419870713 1.00907732823879 0.314636359174115 0.761950701132497 -5.99732070527236 None
hsa-mir-1243 2.60032801617075 2.72844004293501 NA 4.40135213709751 NA NA NA NA NA 2.55769082326515 NA NA NA 3.20068102003523 NA 2.23907457678887 NA NA NA NA NA 1.92469026663224 NA NA NA 3.17744730767045 NA NA NA NA NA 2.72236741218598 NA 6.44957929996523 3.46507879165781 NA NA 0.0875899088829739 NA NA -0.0165310617836947 2.9628599669406 -0.0216166540950271 0.982789217515976 0.992203913441046 -5.99745956383011 None
hsa-mir-1204 2.32855515556179 NA NA NA NA 4.94813005419743 NA 4.40811962841117 NA NA NA 1.81733217557726 NA NA NA 1.32799452959039 NA NA NA 1.92873495631959 NA 3.05950545839459 NA NA NA 3.61925951012474 NA NA NA NA 3.82000120785172 NA NA 3.62340839773982 2.42294000886979 NA NA 2.2315848324452 1.99275212576891 NA -0.17392008927536 2.88679369545018 -0.242841439715683 0.808534472687576 0.943796895210634 -6.0045271130622 None

hsa-mir-548h 1.9699158700044 3.5634455503986 NA 3.98133635629445 NA NA 3.49723585261445 1.88770218812245 4.4478083582161
4.16880194094463 NA 2.58440231785216 2.73817001198605 3.46855834492532 NA 2.89083530959132 1.75249765140971 NA NA 1.93628058246269
NA 2.37291332198623 NA NA 5.30517227199197 3.97584654292462 3.67265311975197 NA NA 1.38883493085523 NA NA NA NA
5.19500044161644 NA NA 2.23406993115148 2.94869117964412 NA -0.395340768657769 3.14191295594021 -0.689171487774616
0.49193916408297 0.863522412677361 -6.0063584031793 None
hsa-mir-1185 1.24278404768936 2.94513262583312 NA 2.91173589292056 NA NA NA 4.99819718022596 NA 4.80012121477817 4.62086031236816
1.66489379347479 NA 4.36353138207228 NA 4.09184314212833 NA NA NA 1.88253550869439 3.00015375526734 1.38866455267265 NA NA NA
NA NA 4.11849002396605 NA 3.70106465534839 NA NA 3.45352337658831 NA NA NA NA 2.6479424519504 NA NA 0.30052370738632
3.23946711974864 0.449076185180135 0.654151403681258 0.898995297558389 -6.00658834523946 None
hsa-mir-601 1.40659503940017 NA NA 2.05395732845303 NA NA NA NA NA 4.10062208195873 4.01733235056718 3.64387901598614
1.68862293342897 NA NA 4.15822899068384 2.97112636887681 NA NA NA NA 1.11724060868746 NA NA NA NA 4.87789765346484
4.80026084027079 NA 3.56310482923383 NA NA NA NA NA NA NA 0.520452572503363 NA NA 0.0292542128373019 2.99379389334732
0.0388028026336798 0.969110791916119 0.992203913441046 -6.00886883073771 None
hsa-mir-373 1.83124693700858 NA NA 3.30118876511621 NA NA NA 4.46219013067 NA NA NA 4.67015160216976 NA NA NA
2.27932603376671 NA NA NA 3.03990650451576 2.73006873344721 2.7408640740159 NA NA NA NA NA NA 2.17232905999412 NA
1.76785009015567 NA 4.6804805056939 NA NA NA NA 3.54514239927835 5.11874606240352 5.02369401743511 -0.208395205595135
3.38308463683363 -0.298010297353781 0.766196192856819 0.92518658980654 -6.01829009027686 None
hsa-mir-149 2.23745134288956 NA NA 2.10839238648922 NA NA 2.10859106266655 NA 3.11100880919702 2.86420938881829 NA NA NA
3.86198278099433 NA 2.1064650033831 2.52854127507684 3.65251077234228 NA NA NA 2.44343586172911 NA NA NA NA NA NA
3.18758954687805 NA NA 1.84661359613625 NA NA 2.49460797962916 NA NA 4.21426567133507 NA NA -0.106285550935174
2.76897610554034 -0.151523060351519 0.879810417712973 0.977027038454738 -6.01924117106657 None
hsa-mir-382 2.25192057764473 2.70463697998282 NA NA NA NA NA 5.22870150510141 NA 4.7261348907335 2.16178306606235 0.661090114697218
NA NA NA NA NA NA NA NA 1.74199529908953 NA NA 2.09344134492599 NA NA NA NA 1.38641723359739 3.92815626579185 NA NA
NA NA 4.6118210485585 NA 5.99233390735103 1.74453587430092 NA NA -0.115531807193739 3.01792062367979 -0.154247304479292
0.877668641538057 0.977027038454738 -6.02125407880287 None
hsa-mir-548i 1.28913823599001 NA 3.94815290988364 3.20915075319863 3.45454442406662 5.4749469380382 2.56533949192853 2.93184368180192 NA

4.78943537544959 1.09608411374849 2.37918717511463 NA 3.77759966067878 3.59042322089202 2.94566145933956 2.65111463492465 NA
5.1352621007578 1.24100850994837 1.71655808414564 2.91978718820214 3.81508418917033 NA 4.24377636485795 NA 5.03313379888101
4.34149352727788 NA 1.6444428193809 NA 3.89541851488671 NA NA 2.93968945725831 2.25460721028524 3.76855266646818 3.99148556954203
4.65415510885808 5.00723034334013 -0.432598838750947 3.35681025094387 -0.919074047987345 0.359643620806583 0.797298174067535
-6.02180128636924 None
hsa-mir-432 2.60403662154801 3.11171187174851 NA NA NA NA 5.78292140268326 NA 2.92526270426593 1.64011423539921 3.89306403078803
2.13091949653213 NA NA NA 2.4817012355561 NA 5.81029497695642 NA NA 1.31347485979876 2.70876431229065 NA NA NA NA NA NA
2.11922451433761 2.16142827433163 3.79603677561756 2.22586736697833 NA NA 6.4878044091883 NA 3.42417182670473 NA NA NA
0.345961965980455 3.21275287733677 0.534440199938957 0.593975287608869 0.898995297558389 -6.02369839390133 None
hsa-mir-498 2.5048524287122 5.08002721300834 5.6863418484171 5.50988079707528 NA 3.87469419999889 4.59499229619606 3.44447285662781
4.92539793970359 5.640777148632 NA 2.56207590330342 5.22154643066121 NA NA 3.7548906218438 4.41058775404512 NA 4.57664336144455
2.43685773828047 NA 2.81252776265569 3.15945454797872 NA NA NA 5.43482720685973 3.30003404507972 2.61062736888407 3.25853752867836
3.27042562887238 NA NA 5.99746814038903 4.27074198526448 4.18071137367876 4.67372691824465 1.29171595168163 5.8170191004355 NA
0.429462756988753 4.08220914630902 0.878180386085518 0.381378120623757 0.818464062400206 -6.02441232854487 None
hsa-mir-140-5p 7.08341766691547 5.39888895517827 6.33637772217087 6.07965662763615 5.60906994488142 5.11398722432867 9.04018802947635
4.45683557920103 5.76256568711845 6.86736320068895 6.13940479677333 7.69303516042997 6.02568568964928 5.6609926018241 7.5662137206042
6.4290885363545 6.36722105018181 7.89941783139378 6.76747723999061 7.05711942861097 7.27464359795645 8.68472069452542 7.22625147669533
7.4075832163639 4.96654685084148 4.94801563193033 7.0174354514703 7.85162827594972 7.10219457579562 8.52185485235328 7.36866157315391
6.79914703707026 7.16255652309985 4.99047085197391 8.02144072877892 7.08116172256344 7.83679883889908 5.6479056111465 5.82106094142198
6.01526294847657 -0.419566735352904 6.67748370234686 -1.05938484111083 0.291133494684246 0.750504307264223 -6.02594442353856
None
hsa-mir-582-5p 4.51676780467741 2.91483956106854 5.27787400824438 4.95512859201423 NA 4.74139624375557 NA 2.04410871699303
2.50188507961568 5.27440669784973 3.12324506155611 3.48520677076526 NA 4.38862011498808 NA 3.17529914223006 3.5612045429093
4.10715705739304 NA 2.97741568812565 2.5553274705174 3.78922543899612 4.44166737259662 1.88217994357152 2.94878797955646
3.95480459122575 2.64192326361628 3.65791139146608 1.70863805112253 2.43174044213496 NA 4.73384889030853 NA NA 4.92980988815833
3.51632756318499 NA 4.96824700849125 2.61820125893415 NA 0.41772763522034 3.59410652120223 0.912048059749847 0.363317191344966

0.799563015989105 -6.03020340479956 None
hsa-mir-1266 2.87505933140098 4.19791480051239 5.13392139220893 NA NA NA NA 3.67360693732481 NA 5.58048706851984 NA 1.83730916395971 NA NA NA 1.69764132659279 NA NA NA NA 3.57175325340368 2.11026789610594 NA NA NA NA NA NA NA NA 4.31295376146811 NA NA 4.54569626928311 NA NA NA 3.23572772125566 3.43393152718953 NA 0.0357935029089145 3.55432849609427 0.0496186182979087 0.960507140768434 0.992203913441046 -6.0313984667861 None
hsa-mir-127-5p 2.78995066605086 4.33279867521627 NA NA NA NA NA NA NA 4.29321359923268 NA 1.75906880200781 NA 1.89257897046174 NA 3.27234092205071 NA NA NA NA NA 2.12073169863435 NA NA NA NA NA 1.53229675351469 NA 4.33586723904681 4.79446387200516 NA NA NA NA NA 5.69695097718355 0.265934276047689 2.8711117372152 NA -0.0315351875415284 3.07363909143596 -0.0425616874462606 0.966120249403835 0.992203913441046 -6.03170787056696 None
hsa-mir-1275 1.48047881813647 4.05508118640609 2.06292336571883 2.93048475502514 5.19837883112614 2.91999473668908 NA NA 4.93723784238081 6.80141187495181 NA 3.29406549573602 2.45115679762518 1.03542037580258 NA 4.70887086739331 4.06764878705556 NA NA NA NA 3.40703308623568 NA NA NA NA NA NA NA 3.66274675114502 4.69639529107974 NA 2.50815947512802 5.07473579582695 4.76917464238954 3.20411885914773 NA 3.32855430834726 4.72335846395638 NA -0.39638643714452 3.69624683669561 -0.69613932593031 0.487570441802258 0.863522412677361 -6.03543595862421 None
hsa-mir-595 4.10292656608274 4.2262864129204 1.77541931767421 4.42520222107031 NA 2.71151209310064 NA 3.16679332967726 2.91255422620366 4.20829850341067 1.90656614326682 2.01076061374369 3.72237888950648 5.14119867862645 NA 2.17522423445718 2.53628871170166 NA NA NA NA 1.90064411170862 2.98149294447679 NA 5.26817278295591 3.00977897126374 NA 3.77519232403948 NA NA 3.3773686996626 NA NA NA 5.07894883383742 4.12701163125024 NA 2.59820354724243 4.01330470398766 NA -0.397196859225191 3.38131368716113 -0.758092713609656 0.449731963192249 0.850120294059329 -6.03565213140186 None
hsa-mir-206 NA 2.95787887090624 NA NA NA NA 5.16202714802218 NA 5.86503269831268 NA NA 3.06859973988455 1.79190783240777 NA NA 3.90811321553082 3.3957463678901 NA NA 3.77273459428619 NA 0.931722926002525 NA NA NA NA NA NA NA 2.87695118029421 NA NA NA 4.35810454553538 4.93437251286355 NA NA 3.09079286573137 5.33776202903168 NA 0.151970715161946 3.67512475190709 0.214204629124296 0.830740838104957 0.950544070924647 -6.03876128914702 None
hsa-mir-660 6.5474204667214 3.93890955848422 7.16238358905476 3.74846020140385 4.88772609519147 4.56579977512092 6.0253682454641 5.6028501529359 3.54137071058433 5.65695330737887 6.3533105901254 4.1670913713425 5.84095102218748 3.89373619721172 5.06098622196219 4.55919453932845 5.1281996172117 4.02224223446511 3.99219464749951 7.84080363836348 5.91082141461979 4.56975786855588 4.61451497204648

5.22709976075649 6.76441208757309 5.54369999156255 4.0937449207165 7.17116960998927 5.01232170669681 4.91813689729154 5.38017824288249 5.97617302941344 5.18103638346641 5.78538970214867 5.82912043096349 4.65067948542513 6.00802033890628 7.49007325535735 5.10535009087661 5.53581984872945 -0.411578392797018 5.33258680550038 -1.0468840259671 0.296843018246405 0.755258818576295 -6.03884877755879 None

hsa-mir-519b-3p 3.38802229395507 NA NA 1.67206459200002 NA NA 2.53774039722019 4.50769495176505 NA 6.51788888785096 NA 1.08720047806143 NA NA NA NA NA NA NA 4.10915392334119 2.20260222056769 NA NA NA NA NA 2.03118473462436 NA NA 1.79999962634316 4.17154782242281 NA NA 4.56339801858886 NA NA 4.82853517343119 1.66349009440152 4.00947528939019 NA 0.244044523842193 3.27266656693091 0.35342616354714 0.724366863751106 0.919356179319592 -6.04122017304774 None

hsa-mir-362-5p 3.41435430694381 4.34035853298417 2.96388007713456 2.47513560916245 5.17467724544384 1.48581960144972 3.70661517772388 1.73655715704989 3.31576552197739 NA 0.747809513510728 4.30945975219997 6.32162372352097 4.16588809750131 6.01380188025296 3.79602848740357 4.22424984158764 NA NA 4.97603737694108 4.49383339080458 2.48667260729624 5.31403032192557 4.48038496892401 5.12998287532428 NA 4.19344566897062 4.45165904692676 2.61454632592858 3.37595358984505 4.38808397795901 NA 2.84727822755302 6.20806057479661 3.99213076695125 4.42633641402606 NA 2.84204612525498 5.0653975907099 NA -0.428596807351961 3.92357285987832 -0.943526671429628 0.347008858796583 0.789608837186188 -6.04681603370447 None

hsa-mir-19a 5.37949756108002 7.28603520914494 0.396217169616095 NA 7.47158996934673 4.02097552946183 3.77672953070685 5.50678717562531 1.80936117089171 NA 7.45022539334104 3.50895410125417 7.26793357813005 3.43889836396004 NA 6.85087834366767 8.88494363530183 NA 4.19752879735048 6.05963330526812 4.21569984071234 3.49262008064015 2.16945643057261 4.59764117649016 NA NA 3.27638654898621 5.27699086964857 6.89705153694942 4.34568508449168 NA 5.8670098091385 5.05197693480819 4.9381544330577 4.05575306883703 6.40750516797519 5.05439149047103 2.97361880528206 6.4695611511222 5.42688740065452 0.47037857684962 4.96432056557529 0.936471566234487 0.350615146771801 0.79183870225991 -6.05330029027811 None

hsa-mir-874 3.17336594900224 NA 1.79803445442223 6.02674341735619 NA NA NA 2.7969560014652 3.50498904734043 3.7022511456492 2.25860981139085 2.37134642130352 NA NA NA 3.79135474330307 NA NA NA 3.12574748414369 2.93288344799763 3.4923463354915 NA 5.76128505962419 2.23507751667393 NA NA NA NA 3.58913004471838 3.36670435932245 NA 2.72142859779135 NA 4.20053792156312 3.23246594576863 5.2371083048365 3.04237409154001 NA NA -0.364273027492128 3.44574952860497 -0.654158569871867 0.514161060452116 0.878297788817638 -6.0556783027941 None

hsa-mir-208a 1.65356208537094 NA NA NA NA 2.81453791143782 NA 2.50906816526628 2.77519210445858 NA NA 3.36085590642904

3.70310957847422 NA NA NA 2.41129086571956 3.76223021865895 NA NA NA 1.01021505590512 NA NA NA NA NA 4.50728126922045 NA 3.23880654121717 NA 4.76194332532213 NA NA NA NA NA 1.9128061702493 2.06242886366067 NA -0.0418493497855503 2.8916662900993 -0.0603990252343021 0.951935489304983 0.992203913441046 -6.05891030474425 None

hsa-mir-891b 4.07131918033488 3.59692943030631 3.77799388610411 4.68018755832822 5.13462782147016 3.26959212625419 NA NA 4.67475980204579 5.03580900576915 NA 3.52575187997901 3.53302565524281 4.07064774018977 5.06373821214711 2.74382753817521 4.55314228945081 NA NA 2.1909926274152 2.12478049274507 1.99573480569243 NA NA NA NA 5.61900307662442 5.72762568325971 2.08228948477041 4.32148112800265 5.44949932575132 NA NA 6.8257350145145 5.37339492716153 5.25221798779261 4.30055162761019 3.23057204672899 4.61215384700975 4.73956873678418 -0.409077601055897 4.19230872198829 -0.856374788874358 0.393270057226085 0.829167288487165 -6.0614754482523 None

hsa-mir-181b+hsa-mir-181d 4.26682552781614 5.2014845038152 7.01885945254156 5.46045735547235 4.41582521908848 4.67302555824515 4.62595673821363 6.60869239399339 5.1554438416772 5.72775359614617 4.45294908590174 5.22205712696606 6.15219271943231 6.49549383786735 NA 4.41387426325015 6.27901973493981 5.00686673559913 4.96602987368736 4.0689592801783 3.53663681910114 4.58847202581291 4.04699528389092 3.86335498253702 4.08085005963621 4.56925307288712 4.00646394031712 4.37531090142878 3.41284030152747 3.62072998925176 5.65768011378063 4.15091992188304 3.6750816136689 7.17872165465364 5.59487798190079 8.13461949328427 5.40983287067553 5.12540483880804 6.48277528576273 5.94075958097306 0.401724481559969 5.06829096350289 0.99820328235113 0.31980488381251 0.76664505485736 -6.07515811854358 None

hsa-mir-654-3p 0.144717183742374 NA NA 3.68128481988921 NA NA 2.16870892262446 NA 3.58205440075476 2.60682107531539 4.72721479895204 0.523441187949858 2.67234810932315 3.83884831533572 NA 2.6880192172034 NA NA NA NA 2.04644161908606 1.40749947357865 NA NA 4.69389012144841 4.63371625068887 3.90380674700626 2.52100670338218 NA NA NA NA NA NA 1.60640428409911 NA NA NA 2.84967487287356 NA -0.294459205911351 2.79421656129186 -0.473679207332802 0.636538950430621 0.898995297558389 -6.0760161072558 None

hsa-mir-302e 3.57472830891291 3.4666682949683 3.90348000062164 NA NA NA NA 4.3204243929661 2.91047757767107 4.87512314180583 3.48083595432872 4.03737660791119 NA NA NA 2.39558865056968 2.28138222150674 NA NA 2.38630381254134 2.6066786495731 3.56290187196945 NA NA NA NA NA 1.77849153727646 2.74608370211773 3.37904156245964 3.81308095923763 NA NA NA 4.54283154695288 NA NA 0.794841040040148 4.66981524908614 NA 0.321818922912998 3.27630775412583 0.564514213239324 0.573380945396661 0.895722046824318 -6.08166704584798 None

hsa-mir-193a-5p 2.44289875740963 4.3839290305486 3.55815743410485 NA NA NA NA 4.33440433546327 2.69386173708093 2.53037054023046
3.58107885797611 2.84439182256507 2.24988154233974 4.3768012498212 NA 6.20243929001496 1.96266778218742 NA NA 3.09239271648306
1.5377650078049 4.02474442695708 NA 4.86781840824631 NA 4.74769045738224 3.28610009562135 4.79145515738388 4.15568105490019
2.39590414737224 NA 3.95677198783459 2.59588292908321 NA NA 5.04913685962425 3.34776960437819 3.48981620897947 4.79056216827223 NA
-0.384266095234985 3.60334717074316 -0.786134297586303 0.433151694212909 0.846625918812306 -6.08349096970929 None
hsa-mir-570 1.86035232769392 NA 2.84557862243288 3.26379968927801 NA NA NA 3.15679213927452 4.09858837502211 3.23878717889325 NA
1.88798815131624 NA NA NA 3.44283932571321 2.24552224217271 NA NA 2.37439089937749 1.94459599488236 1.79193162612539 NA NA NA
NA NA 3.55968323053509 NA NA 6.4131489873014 NA NA NA NA NA NA 1.05520391730716 3.02400730314736 NA -0.123297948099026
2.88770062565457 -0.183948922854894 0.854350481535595 0.966554109504622 -6.0869501491146 None
hsa-mir-151-3p 5.4382319791115 6.83048193577614 7.50580742751107 4.9494952590361 5.36028318373736 5.2024966518628 4.13603821577122
4.75763293219283 3.67566528926538 4.71351666160875 7.27110429697329 4.60546769800592 4.69937604617592 6.60744356527183 7.21417991875073
6.66618608076445 6.28636120903461 5.08760925421027 4.82103995086448 5.24371033339409 4.79129436930319 5.48515622707718 5.89502852182451
5.13624324167595 6.10714196082149 4.72466922138808 6.08738633736931 5.11006412406828 4.08314941851768 4.35763089480979 5.1193990113713
6.37046072514354 6.17216405975921 NA 4.15660648377755 4.42648423884332 5.38950560431991 4.5789840995444 5.52475438137931
4.75254352211226 0.381571318512977 5.36771267519038 0.975267985332332 0.331013682830912 0.770661199795522 -6.09735027546817
None
hsa-mir-1274a 8.90776542639292 5.20923789170407 11.0712936696177 8.83065802070494 8.80668425589606 8.68800331572395 10.9190029299872
7.92630086622491 8.76659023463933 11.0842931859948 7.09437685546273 8.22934926966506 8.10500222385288 6.70238609972806 11.4955491392226
7.06380257315694 11.3574733463702 6.51487819991394 10.2646568810217 5.35229615855745 9.2878285912194 8.94053402013724 8.13236303740564
9.07113806840847 5.49509530507823 8.40340445095555 13.5669301873171 8.74561313710062 9.82389006446775 9.48557062799891 6.97159659921445
9.29504567585816 5.91005116875603 8.91420566614416 8.5120548290908 12.3883465030205 10.6222852819294 5.47391882758968 12.2370124540152
10.589361966641 -0.473832295925547 8.85639617515464 -0.980537341966691 0.328405373279578 0.770661199795522 -6.10480926171931
None
hsa-mir-100 10.4304483437934 9.92497880363151 10.9870148684479 8.43656170798201 8.81778457917339 9.09085775203373 11.6474944006877
9.22411648321959 8.63255303378354 8.11912731745642 11.2875694984084 8.78736554867936 12.753904052522 12.0158020571257 11.5202481241445
8.16551812770421 10.8329000069904 7.3495563953714 8.41770268930497 10.5151612978191 9.85195818992929 10.4130706583372 8.2557076498586

11.8242056499071 9.67946687192314 9.82499505636217 10.3677180199642 8.76092593675985 10.0227529248672 6.16444620651502 9.02186501976884 9.20139731267294 11.2189721385333 7.6067050293978 9.4036265996838 9.02141570167292 9.23548970094294 9.83359606834033 9.75786969636723 9.3245999739118 0.408294034128176 9.64368623734987 0.976449409278731 0.330419498491116 0.770661199795522 -6.10873410013941 None

hsa-mir-34c-3p 1.57414637423899 NA NA 3.58708326473852 NA NA 2.2375792416285 NA 2.29294539400295 NA NA 3.07515905427118 2.74647543672715 3.45213927793423 3.76540159879346 NA 4.2580163843674 NA NA NA NA 1.42373822112042 NA NA NA NA 4.2766263050042 NA NA 5.08327335224047 NA 3.59493270252015 0.0591518066215087 NA NA NA NA 3.87004866367441 3.54463568794663 NA -0.12300061055926 3.05258454786439 -0.187516896631093 0.851558280805262 0.965206096476641 -6.10950291228999 None

hsa-mir-296-5p 5.85474715282127 4.49734106599588 5.37430293661457 5.76290255191698 5.15544320213972 5.44518131785239 4.24241971638322 6.41290273532647 4.71206853749422 3.63228206569119 3.72223792381648 4.65473640494646 3.16750650899784 4.38199714102451 6.84897261053046 6.2157190448711 3.82095118620717 5.0887332651203 5.53666613512198 5.97030230904017 3.13435006223911 6.79771550961057 3.52036829014805 3.78069793028666 5.79868545963754 2.04966411318624 4.89878624518029 2.95912349116145 6.26285067709903 0.922133139177894 4.82404239945237 5.11519075736939 5.04262205775646 5.45990482693098 7.59979243007461 4.28238820875703 6.0637356816144 3.70059154586939 4.81274622388404 5.45464923093541 0.400868776577076 4.82443630230708 0.965603953831656 0.335802101916156 0.772857509371917 -6.11906841929386 None

hsa-mir-28-5p 2.3074514062554 4.79379456508044 1.98997054874482 4.06808143534328 NA 4.16222160596354 4.97892333308587 3.95974220970829 5.37080561912848 7.31582242030925 2.90466902451712 3.21906235222306 NA 2.17545957361057 NA 4.9761311480201 3.95305808874086 NA NA 3.57453185191733 4.38124852328587 4.26457020281225 2.31076259986467 NA 3.72210051024698 2.86502500066939 4.96268789400509 3.31014415092863 4.97299630651295 4.7686291790133 5.4730297093762 4.48600111061605 2.61226019147949 5.4227328443102 3.30615157364932 3.75349013083712 7.55508096104635 6.47542064180269 4.03894975734976 NA -0.387867281590459 4.19487898395317 -0.843473757239013 0.40037747554451 0.834242612656822 -6.13073031864682 None

hsa-mir-500+hsa-mir-501-5p 3.56480416614441 5.16106788880175 3.83567199426859 5.65725378128224 4.05606184149627 4.93976388249824 5.57397303877766 4.10230859063597 4.26091877517002 3.35396234067499 5.42136288854077 3.58168813771523 3.82727569474488 2.92925110978802 NA 3.59408910247206 4.53135296684411 3.63154616564868 NA 2.00751907442153 4.54251849555413 4.45755501042535 NA 3.46839858979422 NA NA 4.63364678160263 4.77526783079368 4.27520646744069 3.87402130375632 5.05276043959846 NA 3.30123501472844 4.69093333421517 5.33177129295316 4.87445884788763 3.98660816666397 4.57187507358554 5.09448308912589 NA -0.349278680323603 4.27153367206214

-0.824989822394247 0.410756542917291 0.834709667241015 -6.14582479844643 None
hsa-mir-655 4.40934052407297 4.25995636227834 3.73348018603395 3.18319976098628 5.14427198128463 4.14079416440376 5.42322618841296
4.43692572961967 3.21748661624453 6.50986235487523 3.92971422087332 3.87018729633861 5.25619600642828 4.10011363552708 6.60770071537409
4.33286449222536 4.59578229567868 4.8999252237143 4.7297478281236 4.42804274719649 3.143717722089 3.91136700219033 5.5559898502364
3.44022841525347 5.07316511400668 6.08368755641268 5.55023351908527 5.1754258692024 3.73821369869854 2.72766682924985 6.27671362562533
5.40141854254511 4.02796988545223 5.2435694648712 4.97105419182742 5.5052944557816 6.14725792101808 5.05440564213933 6.71524371887905
4.6808639083629 -0.360733430161738 4.74080763156547 -0.926188442978205 0.355841079391017 0.794711743973271 -6.15566615953716
None
hsa-mir-1979 4.41510128808855 7.6031897641375 7.50395856165709 7.84329338948414 8.23074090486497 6.70436603498175 6.54297256021211
8.47209035251957 7.65625293921493 8.93131990923747 5.16438989347464 5.86888823969506 8.53720576414148 7.74612414534212 7.01833466576632
5.70445092055794 8.19032766006671 6.92879492048826 9.09859698178417 6.95550425147735 7.78655034925281 7.37968613667115 5.27209950305077
6.30437775558854 7.70060462538345 6.17095431873965 7.5007587403451 7.88367330285252 6.03809444759476 6.61492614265611 6.43389313147514
5.65419558047845 5.36015484341748 6.83741300350293 7.12475734849404 8.53609515506988 4.55718318401753 6.35047632831281 9.59843849275887
8.50195776414951 0.375480649669028 7.06805483252509 0.921762646443165 0.358137812112882 0.796889670494714 -6.1596814533657
None
hsa-mir-640 2.6137069125251 5.77017467800244 3.55576167297549 3.81296884063589 NA NA 4.19448341386572 2.56053313942193 3.78578847455886
7.88576941139648 3.88120918128787 4.16671095949629 2.14313202474285 2.10646891767124 NA 4.70571917298445 4.3314573926848 NA NA
4.1429699902965 2.15604608199749 3.81141276189447 NA NA NA NA 3.22689974859775 4.27997864091599 4.29370412887233 NA
3.75313532908713 NA 2.78629451354192 2.09421822818773 3.08990273278689 NA 4.79329911636079 4.83040120802659 4.59100628224638 NA
0.334932047793437 3.82826492426153 0.667855185969755 0.505353912870474 0.873147305045547 -6.16205740941955 None
hsa-mir-181c 3.58989820417046 4.42326672438667 4.60760780448659 3.96640536493891 5.20884022410093 4.13914571569498 4.50713043607487
5.44120636833155 3.76762084923989 4.24938114678407 5.08976842202062 3.16176581802929 5.61495022755689 4.85797436120983 4.66856263036567
5.73035562866858 2.70661712757352 4.81944134524999 6.00807593738114 4.13253830707374 5.00530062721409 4.13039727805862 NA
3.56627217602495 4.56043768164718 6.8182939373583 3.45591932909732 5.00022612888529 3.01768669320156 4.16339615489127 5.09674585566683
5.93560698505518 4.77900108119477 5.72807069164025 3.98436813576721 5.7818123842814 5.55011611214438 3.36928899018186 6.15065574423047
6.84926672505035 -0.357201984232632 4.70854911166486 -0.903377703962593 0.367788842915364 0.800637806057634 -6.16360954492797

None
hsa-mir-598 3.78613188551072 NA 4.29689502280121 2.14237407234734 4.19056403626934 5.62497206280221 NA 3.69085746189527 NA
3.09447603067477 3.28347459914122 5.49083670564633 NA 3.92719654173759 4.34072230342754 3.4825206131237 4.19565104808087
2.8205337472046 NA 2.72565606860468 3.68622083089927 2.43741822200363 NA 2.99511260633365 NA NA 4.7780221857096 3.81580169898346
5.6236608682726 1.99357229278885 NA NA 2.77863577051989 2.8488065801846 NA NA 5.78682038611214 2.43964285586646 2.59959950673637
NA 0.324247996250282 3.66208059272881 0.660758723320198 0.509880924026653 0.875949279738096 -6.16665715405024 None
hsa-mir-548g 5.30883366503229 3.0659568083459 1.89995672598062 4.59042821987343 NA NA 5.81157144966353 NA 4.52601478873777
7.75436473144668 3.83591830404346 3.32667160944465 3.37584199021452 NA NA 4.80028395332631 4.59942794186584 NA NA 2.03098705806972
NA 2.45553905547848 NA NA NA NA 3.60421081147032 NA NA 6.2254546421207 3.53763294950816 NA NA 4.60982266110356
4.26088363027988 3.4247807375486 NA 1.38171054106113 6.12098509646184 NA 0.267205586230579 4.11578533504897 0.457830964750425
0.647829659286773 0.898995297558389 -6.1687615242933 None
hsa-mir-142-3p 10.574741549936 10.2120263713127 10.139364104284 11.8087740664554 7.2271909530177 11.0071653525981 8.85940171773591
9.21477978688625 9.77730222254096 10.0237837066072 9.50593655601907 9.60446396104401 11.0201257788775 10.7895519688178 7.21454382251109
10.5384672548277 10.574546229442 10.6148443045301 10.1881438745939 11.9464075852464 12.4958875544844 10.12328641752 10.4840426203181
8.90522071527994 10.0127941542753 10.6062712971488 11.6404986893526 9.07923188549578 13.0833125938242 12.5752164322565 10.2041961469627
11.6641517177118 10.7312698699711 8.13548744085454 9.10731636166314 7.81027709072309 11.3975484373248 13.3312301178454 6.90029948851407
10.3528991419287 -0.389943850308557 10.2370499835185 -0.906811037159848 0.365966299419829 0.800637806057634 -6.17310564394453
None
hsa-mir-651 4.71942303308658 2.69290697222661 3.14912820420342 6.28679902624275 5.48213552252389 3.54994781285311 5.34573690524126
2.85940532799267 4.83598468535664 6.03545516251391 3.03358927445731 3.96400509216427 4.55119995361618 5.79817710190467 NA
4.72829305929467 4.31434053641857 3.87663266076973 5.83581046760895 4.69481805976878 2.74131325194962 2.36224480142752 NA
4.31148868492754 NA 3.82783368217872 7.07089306855947 4.54894298329728 3.08048973386012 4.84818102102129 4.99179736416317 NA
1.46771612586882 3.43136129618315 3.36397260511221 5.78564101406163 4.61203312601495 5.24868844825367 6.72045008122257 2.05461184252189
0.368200888880179 4.33948466635743 0.840433617479713 0.402044543900582 0.834242612656822 -6.17765263483462 None
hsa-mir-543 2.12986752987418 NA 2.05059240464226 NA NA NA NA NA NA 4.07488582383291 2.43327253402183 4.88091499427232 NA
4.1468843476658 3.92795671631084 3.58515362954004 3.08065406345643 NA NA 0.768852213300134 2.24308433436629 2.57659730612501 NA

4.42110037799766 NA 1.94129542089734 2.88832339693955 4.04208872175446 2.43871928375509 3.35753103994348 3.83895602699058 5.25482669502763 3.28539368462792 NA 2.47623278764283 NA 5.42387182674208 3.63881237892164 3.13269140390874 NA -0.289398220017678 3.28154235770228 -0.556963709390179 0.578478050843292 0.89755603144822 -6.17769237810647 None
hsa-mir-1975 3.289541882691 3.49986362397086 5.20976974406253 5.664217101309 5.17594799742277 4.32607988313757 5.79009443832351 8.54851913215403 6.95181625168116 6.17337062717917 5.19411580721831 6.96067942679542 6.62541157147739 6.39768928442701 5.2127843785254 5.10888529595038 8.45521342950492 2.6788360012149 4.92081068317785 7.82997554700025 4.40773597025694 7.27563477031005 3.97883453150863 4.50312763839884 4.8182649629466 6.62235366230682 6.17752567285167 4.9513982554672 4.63060446226287 5.22908265355748 4.90477943962813 5.69226316623491 6.52964209455552 4.56725370128268 6.51949442231035 4.24333787849102 3.7597727082439 6.14679688679981 5.85547084795187 5.82592972760367 0.368715932712723 5.51632313900481 0.891140339906932 0.374286118362678 0.809498050327186 -6.18694244530101 None
hsa-mir-141 7.91332090201307 8.08229623680395 9.65300040680989 7.28036679297832 10.7709448283187 8.92876622154177 10.2265952595003 10.3583468646883 9.64761689039693 7.40398837388305 5.49846544610564 6.89726546678744 6.26613728866225 8.77974367079105 11.4955797092581 8.148874156811 8.46484688961173 8.67617560167891 10.6603922917895 6.86616320978082 11.260016194316 3.94136993733541 6.77511815759363 4.9927997712742 7.8245691105427 9.89313814021498 7.03671645075325 10.8699658447433 3.32951617752799 10.3917073006095 9.91741328135928 6.41107786575922 8.65581799269195 10.2155093354636 3.83767197987116 9.64062728931744 8.00869875714964 6.62218281258177 12.2998806226473 11.1363482541406 0.447937061615891 8.37697579460259 0.890658349150952 0.374543874031982 0.809498050327186 -6.18736424818061 None
hsa-mir-217 4.62435465956013 6.59224548910251 NA 7.00268915606695 11.6416855159338 NA 4.98903759854956 4.07052015583156 NA 1.76628735943994 6.99191618303098 2.15078391826047 NA NA 11.9042029113746 4.22401490153958 6.94132090418424 NA 7.9367435697335 NA 7.6079121560066 4.06228024401198 NA NA NA NA NA 10.0179893484852 NA NA 4.42432335603783 NA 5.61634487210573 7.17822083311511 2.32411475609499 NA NA 2.88938895361187 NA 9.39604749975161 0.271847304731612 6.10692837917403 0.376229823183304 0.707352497926547 0.917276465053135 -6.20180294224163 None
hsa-mir-125a-3p 2.1246939260075 4.65046064443821 3.83030174480489 5.08421306461945 5.82502258011532 3.47082676772806 5.45040425692428 7.33370336847883 3.56982455281373 NA 3.47706520090055 4.68915914568508 4.23008721113781 2.86144054114289 5.60338484632914 2.65884294069569 4.70902299038147 3.89282556669522 6.43934136401124 2.17857872193853 4.27401722211884 3.1981292150948 5.49450385826801 5.60054543109873 5.1374745600628 4.09822800436304 2.226696277772 4.92641230999762 4.7401909281583 3.61704793022062 NA

3.86675097900665 5.46378935385141 5.58501764408151 2.7708024872141 4.4483974080407 NA 4.74348723820332 5.32445073121146
8.64364434692416 -0.355574686435122 4.49294014487935 -0.814098115127588 0.416910301306514 0.835205686670525 -6.21340249402996
None
hsa-mir-339-3p 1.68874946591592 2.15750778708141 2.58065346077873 2.11837756165967 NA NA 4.78840500325207 1.77767738284569
1.53072173346488 NA 2.61328797234815 1.96220522185116 NA 2.9671153370217 NA 2.80595872744095 NA 3.4566639413856 NA
1.6239674549275 3.0704838440711 1.73070723724431 NA 2.00942419292993 NA NA 1.69787344808533 NA NA 2.58905192289085 NA NA NA
NA 3.76910285026431 NA NA 1.74395772093097 3.80928484729045 NA -0.0854633695039109 2.49957986255622 -0.15215549199498
0.879299337366446 0.977027038454738 -6.22501367746525 None
hsa-mir-423-3p 5.48966887310237 3.11187085018049 7.39623682119514 6.00533600848715 5.48930152472709 5.98412400344786 7.07238215112732
7.08421574851677 6.5285031824538 4.80564610711395 4.66360903970071 5.00753121383745 6.75987342322018 3.77951769137628 NA
4.77747123153484 7.56817275916172 6.2010379516952 6.92170883317462 3.04540559236545 7.04180688614787 5.13361865765381 5.54222393904914
5.63278450788404 5.89059219596013 4.07436687958202 6.91822890227951 8.47150870653593 6.530404425697 6.85603085371657 NA
6.06587477057369 7.62710082471008 1.72411659369481 5.76765838181714 7.17913874340239 4.98227363628493 3.66872520387652 7.79565989236311
7.52574940998518 -0.354539494989235 5.84524937941138 -0.80593380535323 0.421578693534117 0.837252985537137 -6.23315014637498
None
hsa-mir-140-3p 4.56903648213445 2.36911580979371 4.30747624187425 6.30858795915342 NA NA 3.52665999883371 4.54706389992563
3.5790693948836 NA 3.50708042142788 3.16824779352041 4.56777467835451 3.36705431025405 NA 2.84302797402111 4.99575973681487 NA NA
2.93851125343061 5.86560893858556 4.07300767940649 NA 3.73631203133124 NA NA NA NA 5.62660766898685 2.46951622365222
4.64399160421352 4.90890536218245 3.85413196754885 NA 5.34846419232665 NA 4.4304509800185 0.559365248393233 NA NA
-0.238246370483076 4.00443311404271 -0.456340243429192 0.648881525491083 0.898995297558389 -6.24002274787805 None
hsa-mir-340 5.75056239840187 6.69823204836213 5.35233193881954 6.02141132854282 6.93402284094855 5.91484726380778 7.14934967743847
5.60717816444513 6.75137057917993 6.42681137209198 4.14381692608574 7.51378008664448 7.3794992311472 5.67296760599178 6.74158364241436
5.93184811714365 8.25224506202684 6.62878776218644 6.95652826028635 7.63913408785269 5.80331664713957 6.26401741505646 8.05554417343188
6.21341253420281 5.6289836518965 6.36712113662019 5.63173774488932 4.06441904596473 7.04877602826002 7.39881376633931 7.303390294838
6.81944568073807 6.62660948772402 8.07094386015541 8.2819595263484 8.06908856591789 6.9948155328787 5.71088055065386 7.14620130458141
8.35813451030982 -0.319565153206431 6.6330979962941 -0.816920217972925 0.41527417794157 0.835205686670525 -6.24923305951867

None
hsa-mir-378 2.44364069075176 6.16463163465275 5.55179526901457 4.18702238726738 NA 4.05523739043076 0.85175268652918 3.99438414222023 3.42097863961474 7.54024755040847 2.75785308781827 3.45155172715674 4.0320046606449 1.89759797846957 5.74134186505753 7.57108591676752 5.46135912762658 5.0669776600064 NA 4.27084116424834 3.94962066129579 5.72006654149417 5.08883330755526 1.01033542248393 3.78827772819809 5.42789614028872 5.21768763352459 4.73990630591144 7.14299381419338 4.62406783685652 5.894097141413 6.38335142822793 1.54012911478354 4.66694869177817 5.29847493928332 3.53136059799754 5.31956826868328 4.98274444975279 5.16393504531721 NA -0.351109775577976 4.53920536885741 -0.767562980838959 0.44398244514263 0.849614704748016 -6.24953899320129 None
hsa-mir-210 4.97255634319988 3.80809240774505 3.94492663280452 2.03653871131068 NA 5.91088371567774 1.26825118585085 7.01896450172344 5.86860409409117 5.35973389684604 NA 6.25303778361454 3.49467929729636 3.96807519234518 NA 3.33209115511715 2.19549064835138 NA NA 3.52554360612542 2.17975866605204 1.83363262175438 NA 3.58941210109754 4.78705405597966 3.73953974513043 7.19270389033301 3.80661352885027 NA 7.34426067808053 NA NA 4.14957316704464 NA 2.00019301694499 5.70946838353688 NA 6.22417591231325 5.61333307076326 NA -0.277429145286774 4.32597100035644 -0.523199669510538 0.601677627097585 0.898995297558389 -6.26724319496002 None
hsa-mir-561 2.25280275521118 1.65794418352162 NA 3.64667000356726 NA NA 3.24852201133267 4.24740105086165 5.00447322296891 2.97369524987206 NA 5.59885003048164 2.55248876402594 NA NA 2.98548508528277 4.9153223958307 NA 2.68661410348945 2.78492062569124 3.97860657118958 2.4402159825063 NA 3.24301262277466 NA NA NA 2.87709133818545 NA 3.80653387165027 5.38885391026373 5.03229619261742 NA NA 4.15564817902638 3.11416522716536 NA NA 1.53848825677819 4.05273855661656 -0.175191432940928 3.50761834128796 -0.334996334030771 0.738153760381417 0.923995650122037 -6.27078826546962 None
hsa-mir-548a-5p 3.14420701512505 5.95370910047102 3.26605635325875 3.85629370914715 NA NA 3.96259088295986 3.94709141149348 2.66105139932747 5.63609122846674 2.0918266563387 3.02347858107698 NA NA 5.4769257938977 3.56085774828691 2.84977880529008 1.19577180041152 NA 3.1956223444523 2.59311916514239 3.52043071063726 4.48255689877172 NA NA 4.01916065192606 5.09335246053732 NA 3.01511861989681 3.2071207663618 3.20223930988776 2.92022588303963 2.40499009042221 NA 1.41987197429773 2.72216909867641 NA NA 4.80400700508586 NA 0.249293062922071 3.47234698088167 0.514310348119854 0.60786084431842 0.898995297558389 -6.27175368034342 None
hsa-mir-890 4.52432189361461 NA 3.53998131996829 3.72241292996153 NA NA 4.29375447990719 4.65738553446681 3.18219103977097 NA 2.81183020248766 1.02340627623805 NA 4.31194001709556 NA 5.13785248270077 3.29576265982967 3.07484509886894 NA 2.9606480781864

5.1376285866413 2.45635757907545 NA 1.88380438172405 NA NA 4.98363113988469 4.33325624649952 2.69512148433692 2.75014722614783 NA NA 3.96328569400346 NA 1.60735845127536 2.65512109220281 NA 3.2594026174367 4.87614306207624 NA 0.196279717052572 3.48550358297603 0.384555632844683 0.70117416102408 0.917276465053135 -6.27569271100981 None

hsa-mir-490-5p 3.69551708504159 3.86651983365406 4.03146692217931 3.44251246018282 NA NA 5.14193571434618 4.41241215258805 3.46899843634495 1.53564981198171 NA 3.67833040999265 NA 2.8896608180093 NA NA 4.84061257613342 NA NA NA 3.41608829781983 3.27171607874105 6.43731018631264 3.50811837110885 NA 3.84514390330913 NA NA 3.4576206162435 2.9055468496419 NA 2.30493955699548 1.31422122859668 NA 4.96744904336636 NA NA 3.28326034324849 4.54842328147992 NA 0.122614994848079 3.66362843379643 0.229893648655052 0.818529020313333 0.947357004316583 -6.28162601219896 None

hsa-mir-365 5.17837705407462 4.71657198660241 7.07695297407137 3.56222974971404 4.25630185001264 6.13003276374026 5.16468237577701 5.19692152756881 4.94200121814564 4.56285877574298 4.95672978166255 4.17338747176639 6.52891558564034 3.066277386893 5.13944009733474 0.248031261459376 6.30560067178999 4.97992778628648 NA 0.957991161882055 5.93822937724381 5.7878527672758 7.33852740335453 5.0517055126731 6.56034577762713 6.48960372267562 5.84386992124838 5.72331443568072 3.93172971501886 3.51897483233056 2.05057983982543 6.61231898847499 4.16705653977621 2.47031184901237 4.36822032183906 4.87763075616281 7.52950808996634 1.70232372965983 4.42589911736066 4.17912724263002 -0.341870629614722 4.76180413897438 -0.758121743188448 0.449579798569457 0.850120294059329 -6.28216570042891 None

hsa-mir-301b 0.987866375867886 5.03495757957976 NA 2.21783384210571 NA 2.7828978702372 4.3582677059377 NA 3.38725672176945 4.26083130828166 3.79058813634926 2.22411271148172 NA 4.76180273460719 NA 1.26608120275091 3.14912709655532 NA NA 1.40516555013793 1.00423073650681 1.24503818965647 NA NA 4.07611042346605 NA 3.78933800642384 5.06872816352784 NA 1.95943141213199 2.71870180790219 NA 3.17229426017649 3.76404901910481 4.20551724527079 3.84600690522162 3.41415266367978 3.92057962450046 3.63503971179379 NA -0.224586771991507 3.16466692611202 -0.45110476144406 0.652630025097705 0.898995297558389 -6.28582297419006 None

hsa-mir-134 4.81141878638481 4.32636835419462 2.56431250779433 5.38075043326411 NA 5.0041541514629 2.98160156393129 3.08216493799068 3.42696006473243 NA 4.93220830488668 3.01780744937762 4.65242013774236 4.50118067729714 NA 3.90743013464086 2.81366670115157 NA NA 4.79006115283226 3.34333680819879 4.79204932705505 2.4961039269908 NA 3.84829905834018 NA 3.32200280959456 4.25900547543522 1.42291192836749 5.48856129216102 4.2346135022304 NA 1.9389451888421 4.20315053452337 4.61501156178008 3.44262958114407 4.67861337121337 4.19469234776826 4.09409441942289 3.31346310214035 0.266511088441186 3.87124967477786 0.605503140959535 0.545814892053621 0.891941408965673 -6.2869074824327 None

hsa-mir-487b 5.08846101003146 5.06782893031599 3.54638303753136 5.4754232121283 NA 4.6185390712331 4.60639816943265 4.6014663590468 5.18385647647153 NA 3.01786638989529 3.09328069438718 5.45697020707031 4.78805648469644 NA 3.36268679325151 3.24518908208337 6.19825932213722 NA 3.32817074700482 4.12542638561048 3.0449954284651 5.28226201489079 4.14158689415515 4.43627925951057 3.77433143348052 NA 4.72848338131857 3.30561512823017 2.41151443217118 NA 5.4086075181179 5.19855217639704 NA 4.95714748637054 4.729925010578 NA 2.80500759839525 4.07714539964662 NA 0.255635279347308 4.29373275916307 0.573918638829251 0.566940674282017 0.895722046824318 -6.29118902216385 None

hsa-mir-526a+hsa-mir-518d-5p+ hsa-mir-520c-5p 4.1912095991756 6.29011729543793 2.49549023678273 3.94468819795626 5.56174225884743 4.55862195101013 2.28121930788049 4.6418601367089 4.6732641785478 4.22856160023651 3.97962607249865 2.56621277201559 4.35755450419688 2.35068629439757 5.49669528010805 3.59331997143046 4.75705193572965 4.40397914528669 NA 3.75822890781253 1.58969793208223 2.68157375424211 6.14924115787846 2.59549881213568 6.21562182127791 6.67251503282291 3.1938787400224 5.87277343345514 3.79468015830011 3.12154099958465 4.90857739885084 NA 4.81624188173503 5.11417143187012 5.18067162827138 2.78290077555023 4.1946529841097 5.61529178197747 5.17685977829498 4.26630757653193 -0.305924601733341 4.2650743875014 -0.727337786950198 0.468172171042857 0.860694570545253 -6.2923628969899 None

hsa-mir-215 2.07804715432007 2.61842338526594 4.16699365675287 3.9393059383433 NA 3.3254269659919 NA 4.04365848782274 3.87104255599507 4.63768851900337 4.41112993522852 NA 2.49195996382961 NA NA 4.88754579151646 NA NA NA 2.92505592502434 3.98736298389823 4.45342924795801 NA 2.94929094565097 NA NA NA 1.95421866366004 2.84377988450867 1.28437941715624 NA 5.34524743574005 3.53792491135051 NA 4.7994898625767 NA NA 4.04756116449136 3.58360859939614 NA 0.0903298763135831 3.5731552780644 0.171072571403428 0.864427733847573 0.97429892244876 -6.29311706223752 None

hsa-mir-299-5p 2.68596052170453 4.8437752873459 2.06009812819759 3.71266794284103 NA 3.181128278964 NA 2.84748444311178 3.68494572311893 5.77818949918833 3.17751813841449 4.27589255408742 NA 5.08804517419578 NA 3.45851127454201 NA NA NA 2.72194609541134 5.38730307208888 3.82041166597574 3.70328959318071 4.06568344293971 5.4667435723093 2.76457098402576 3.28266735152921 3.12781853073126 3.15938232952435 4.17789155424316 NA NA 3.72242365782445 NA 3.10267125418491 3.19278793803345 5.92680703451756 3.24156947092427 NA NA -0.221045297228605 3.77350658975557 -0.465961035341333 0.641980836421576 0.898995297558389 -6.2949378042536 None

hsa-mir-627 1.77323759297493 3.5337741481523 NA 2.97013248347285 3.60225905471683 NA 2.5341363464518 NA 4.04791985336146 NA 3.1380133980969 2.12686178152911 4.73477753384349 3.04924706119589 NA 3.22699353355482 3.88461729957231 NA NA 3.17169085055631

2.54649037828985 4.3316478220478 NA NA NA 2.2616636202536 3.44867976722373 3.10693496380374 NA 3.66338781225911 NA NA NA
3.8492097961148 2.86191050913973 3.10428232207024 3.05154420041189 4.21757175606931 4.02318333360765 NA -0.157311861583596
3.29040668875082 -0.321752630998751 0.748139718568229 0.923995650122037 -6.29734495589904 None
hsa-mir-199a-5p 11.1786160673732 10.2146504067199 8.56591421645716 8.6824060033907 7.64673071491116 8.12257116206477 7.72977201011265
8.9806308733905 9.89349998097281 5.43351541206842 8.74635445134394 10.8827223295394 10.062362906942 8.89217808086264 7.85005056524422
6.39489387735159 8.03464301866577 9.10134649194823 8.98413527541438 10.1224626517572 7.73250558291243 9.31024544944682 6.56639514710351
11.5561559929237 10.228350266922 9.18905433415723 10.5260177174199 8.37140314866252 9.73512182165461 7.05804443405299 6.26477900612666
7.25673527428941 9.8305128988041 7.49800329165921 9.29053233377118 8.4539560712568 6.81933410232138 7.98100365038835 8.11224242408473
7.40203598336102 0.316851378260603 8.61754713569623 0.750482021975391 0.454144227795139 0.855778029251465 -6.30044087899414
None
hsa-mir-148a 9.90119517256333 9.85561674146804 10.0133650469452 9.43268285974206 15.1822832969648 11.1407712549562 9.56803985270331
8.62095439919199 9.48671290656806 8.42977438013309 12.0435754603435 7.39651749697687 13.1660668329355 11.1479119230259 13.6524644992537
8.63895225947195 9.37455930350183 11.8692830735597 15.4016883896639 6.48404947388705 12.5141639769037 8.58152183150746 10.2408516620631
8.68459543430255 9.98385767682829 9.66344257448281 8.26608488192612 11.9624662984951 6.8011565713558 9.357417503989 12.8195465894662
9.44838068415826 9.92484037921388 12.6869968838749 10.1838766518832 10.2230558664802 9.61759031542789 8.94134968834671 9.36485037534268
14.2876056547595 0.362640656152436 10.3590029031166 0.737511409708078 0.461967607470184 0.857357456751318 -6.30993558424348
None
hsa-mir-451 5.34111636104991 7.96895206100075 11.3884036719198 12.781004667826 11.7146250974689 8.54685857635556 9.95261305290533
6.55664901541272 9.91177069550141 9.73568980303781 11.1419776975284 9.78260207128292 13.4363199717153 10.6077244858278 14.0626297063213
7.2293094960869 10.1365854030748 10.5028145893479 10.7583012275685 10.8614470173257 8.22712925191093 14.027642259148 12.2857535228689
11.8428972178921 8.46910968551596 10.6327601268274 9.5038401997 6.35745828257495 9.17453621391556 11.818245246128 9.46813529990169
14.082984027431 6.97984471198347 6.65924292111516 9.89142881958281 9.14742571439323 13.3156531458833 8.74507105982337 6.41136678818834
7.80976800880547 0.378355108248401 9.93169217930368 0.736123066019603 0.46280948280861 0.857357456751318 -6.31094214094449
None
hsa-mir-450a 5.90395737968423 7.00374633973138 4.84460678059602 5.41345233471699 NA 4.93066849014429 5.57422045653643 4.62948837459412
6.86278305346406 4.89210083843587 6.04542422953863 4.96318268991224 3.56306407822464 6.85426236869703 4.55816770482663 2.61136365419177

4.63392831645128 NA NA 4.73593135125797 6.44139963420581 4.7203220852628 5.80931842417316 5.24161355411954 6.51585052703652
5.08183589056009 5.55919579634535 4.96259138933289 6.0084414688237 5.3068034761908 5.24901956076908 5.642609636158 4.55236882900586
4.93109612217555 6.39999280590798 4.92936081632798 6.02544181132413 5.53019264908471 4.54200549976088 NA -0.267040864466049
5.3186057893769 -0.655772541542544 0.513006302302109 0.878297788817638 -6.31327510837718 None

hsa-mir-615-5p 2.12399425265932 NA 4.28284027307923 4.21360209546227 4.4392412597751 3.14721957971263 2.4378035404167 NA NA
4.27897051472453 3.56497555939783 2.46839181139309 NA 3.84946989858455 NA 3.10269094555425 3.78107890026906 NA NA 3.47613558216353
1.41706609656145 4.58609052593032 NA NA NA NA 4.10739244110691 4.43422452340264 NA 2.01084204688713 4.56334938774318 NA NA
6.14999213261281 0.927197552301805 3.9166688948776 4.90067447950573 2.03436390260796 4.17268234683463 NA -0.127372472670084
3.53547834174257 -0.246634705950977 0.805567055584798 0.943796895210634 -6.31819278834724 None

hsa-mir-410 3.89576653702452 2.75309412595137 3.25100743543633 3.90508413660581 NA NA 3.7611588644693 3.93906940986562 3.08243645723596
3.92486181277058 4.09946628228227 1.39600401914805 4.90612412680663 3.4166283292677 NA 3.64985968335582 3.10993658257698
3.782312009117 NA NA 1.43810353943101 1.35979994340109 3.76225041754954 0.548677108793019 4.10887745798594 NA 3.3698301091037
NA NA 2.2936721059835 NA 3.7724240730412 5.16897288777162 NA 5.88663867011365 2.61421083519651 5.04413167966241 2.96431146458986
4.1076974155775 NA 0.207739865446604 3.42456577655567 0.435466018480462 0.66390223081877 0.907784682956277 -6.32728814776896
None

hsa-mir-626 2.22101794695269 3.77749180166598 1.75900406625436 3.99595560303269 NA 2.44858288075076 NA 6.59701399369987 3.4426432430781
3.43084453807365 NA 4.08154131047097 NA 3.6660747795898 6.19783218330928 2.77303223630056 3.45511393920612 5.45565144057296 NA
3.86781052055183 2.8347641156953 3.90987641152434 2.43463356808197 2.76714710620913 NA 4.57316200504121 NA 5.87079234414125 NA
4.94814089575599 5.60702286169375 NA 2.46466521601282 NA 3.70317181057188 NA NA 1.50336769508325 3.12925781184526 NA
0.165807212095961 3.73761527130244 0.326099598967508 0.744848521371361 0.923995650122037 -6.32798339575963 None

hsa-mir-190b 4.76216859939872 2.29145678693002 5.22279142817097 2.69613562637006 NA NA 4.66825281868649 4.2205216816733 NA
2.9797508868599 4.92986404444577 3.39022161990809 4.23836512608819 3.25600979236438 NA 3.80240836486922 5.23709991249962 NA NA
3.24092072547408 1.22204821186355 1.9880555812323 NA 4.31293120012825 NA NA 3.44974730044946 NA 3.07097169094033 4.1979122788144
NA NA 3.44247684224215 2.71614689093115 3.75560120855935 7.20722435665468 NA 3.92981667310321 6.14568894245558 NA 0.137445907628713
3.8605610996582 0.268233101897709 0.788927908018552 0.933778355913257 -6.32965643962311 None

hsa-mir-192 (+++ see message below) 9.53932709001468 9.11068054157733 9.40608520010801 9.15110789151323 8.0586857841173 9.8964972619041

5.46393089838572 10.8192767064659 7.30916338085576 5.6300114269891 7.22586134700755 9.22017078256481 8.39591361914715 8.34692663461121 8.01140222248119 9.16691208607839 7.00624362967414 8.17935332213769 7.67667768566151 9.93269950421313 8.31747329462528 7.52691529777519 9.620378637172 6.36189003790963 10.79068794272 7.9893373824802 9.51734674813597 8.9348993644326 6.95836350150226 9.57908993175927 9.25119124816603 6.8285153550917 10.2444164943822 8.43098463862836 7.73656269276122 6.05871372187786 8.95797276927772 9.82919621217808 10.0663932763275 10.4442370132207 -0.294881927245797 8.52478731439829 -0.703493136976638 0.482842882687954 0.862756303026751 -6.33405675537571 None

hsa-mir-148b 7.12026600068889 7.86658837755065 7.46353235173305 6.84280813365686 7.97376589272189 9.03736802585834 7.65066629958924 8.14295203816707 6.7790573896701 7.61567479978119 7.25765137221355 8.6722637159094 7.61592520370117 7.66480561415096 6.55242597420852 7.76515278778969 6.7966860824918 7.70588051150977 7.54805295371517 7.21440137927535 6.43265237502242 6.73283023545113 6.98526660359712 7.48916664713943 6.59162504141817 6.88069602748588 5.21212062944896 7.75188014960604 7.72514977936761 6.77390674403988 8.74077634308663 8.46960434489493 6.63389495312316 7.36654592847037 8.24337202338511 3.5462088400489 10.1337197607532 7.90571438046991 8.2893207710673 7.98744811600853 0.2697012605249 7.42944561495668 0.685847482096893 0.493871711079911 0.863522412677361 -6.34612310182785 None

hsa-mir-29b 3.99809435662915 5.09553333033485 7.01215939486635 1.97175194409386 3.73990650654571 7.53540316962003 5.6388851148314 9.4617276760031 6.43773901498328 7.01918840362177 7.74624716726595 7.96337898175236 5.75056973811048 9.08365745647968 6.84541409650986 7.9324720263655 5.9785987846301 8.67082435165039 8.80806023123811 5.89305701059554 4.87759595041836 5.57395715332255 6.46988363714049 6.25345124632201 6.00098558303946 6.10137377346888 6.33964550979587 7.74927649082977 4.44593527531913 8.18060204912729 5.70950965734708 6.40665653825253 6.49999426143774 6.88098838400614 5.01650715734592 6.55423025687308 6.58730094652384 5.81300532105752 7.6829483258943 7.60494398172824 0.291693862843864 6.48328650638444 0.677187209585871 0.499333937379351 0.870226486242049 -6.35193370667516 None

hsa-mir-337-5p 5.0298814962464 5.78834998531082 4.42728874434294 4.02492020544225 NA 4.26087717893313 4.33922781469769 1.57815004437408 3.75354563497568 4.25399070932989 4.13598962664608 6.1769165151219 5.61905502678375 1.07697292526547 NA 3.4849779291968 3.6308061484079 3.37433460219158 NA 4.5270501644045 3.94799637133646 3.52609217162551 5.55643579822295 2.79367450353317 5.63099692549119 NA 5.44207871547631 2.97970942833306 3.83126006878249 4.00611339151164 NA NA 4.13832728838671 NA 5.24161934296319 4.59640354436191 4.12866839817299 4.52874916399519 4.19261484538441 NA -0.215519835308819 4.188221084664 -0.480788662403004 0.631409416229415 0.898995297558389 -6.35336608271783 None

hsa-mir-361-3p 5.0187868182701 6.15579477929218 4.39364135338988 5.66092332505093 5.36331164723354 4.64684722058339 4.79366995823511 5.42827033749044 3.85649698149969 6.1343161733906 6.03751756317536 4.87069798578666 5.26904141052548 4.69093072914654 5.020279875802 5.65442463827501 3.74134034717336 5.55022870627453 5.06819905832443 4.18251173452602 8.59539595281019 5.50697453460435 6.07790121972614 6.00005616366756 5.43922872489079 5.08430422300841 5.63417246053507 5.7627512537553 6.41631374334396 4.74322960509045 3.74707171049178 6.59671063921217 4.8547148746579 6.73891376413186 5.60070687573929 3.47849810814901 4.68072454738696 3.85231367268261 3.36518206903745 4.61441150859376 -0.262617250403487 5.20817015737401 -0.672431148878068 0.50234742104555 0.872842829188792 -6.35509356881629 None

hsa-mir-577 2.0414011298888 4.15128692135548 2.9350332573932 4.44078577919968 NA 3.87622580633906 NA NA 4.35800541646752 2.87964403985642 3.66267952101876 1.53764765492135 4.12034090144785 3.58018928395935 NA 3.7478489524954 1.87034035397408 NA NA 2.77863591027858 NA 2.19241938830457 NA NA NA NA 3.07490628891255 3.25474052836159 NA 2.34583420468549 3.92353149061594 NA 2.75793652199248 4.26704628900748 4.17016198948797 2.15002534596322 4.1424438399096 2.3617271079976 4.14143665616545 NA 0.0524395477588753 3.26008748384613 0.107509013405445 0.914544232622225 0.985980621282861 -6.35918233408299 None

hsa-mir-105 3.34167224357525 2.49666019326938 1.79873845905178 3.1419482699943 5.46176444563107 6.84559482819313 NA NA 4.56227611419604 NA 2.75698422359482 3.08968773264327 NA NA NA 2.35083598533346 4.5691786404411 4.1486175828705 NA 3.87058027102644 4.15808107254147 2.84207630478976 NA NA 4.14550329029432 4.00425572247331 3.45918984479022 NA 2.32769235939983 1.90768428358777 3.90412482918904 NA NA 4.0215601894165 4.04066237526267 NA 5.37260153184826 2.79282990068812 4.83727087673795 NA 0.0477697237539472 3.70184890657076 0.09489881286079 0.924535425239549 0.985980621282861 -6.36331125381242 None

hsa-mir-205 4.91998121252612 NA NA 4.25730061326546 NA NA 9.83929416494197 NA 9.58658280579705 11.315577938613 1.76108859581427 4.57646226470656 7.20706298262301 4.71363182211801 NA 1.86837472994783 6.25207800369337 NA NA 4.96626628589203 NA 4.94057172203099 6.63863650376357 5.62406132655281 6.91379783419312 1.80805903802755 NA 3.83423314664221 5.22957864889631 11.1281382254392 NA 4.18904234537891 2.25404085370681 NA 9.47546096330215 5.74434465889825 3.48483485118959 NA 11.4522618656499 NA 0.0302802147326489 5.922337053985 0.0444268403632501 0.964629586276861 0.992203913441046 -6.36386954530485 None

hsa-mir-1974 4.47165515434333 NA 2.78713535442466 3.25589590654412 NA NA 6.09186925321375 8.58964062497414 3.59895687389172 4.46457948927313 NA 2.88750792504981 5.86852223220722 4.22184343952899 5.2027303927848 3.9270250601577 3.39097026276114 4.80971029520936 NA 2.79751649627731 2.15086940973427 4.39676181611108 6.15663948418474 5.24884437718575 6.45073966004281 5.98042188702327 4.96664493420684 2.65330622143465 NA 1.8052904233604 4.77596827308317 3.72466832509448 2.96994279447597 NA

5.39062168151878 5.35225938787233 5.10562477338935 2.95484892020875 8.92314165461131 NA -0.223076123224195 4.5428797745056
-0.452048028664803 0.651926722382165 0.898995297558389 -6.36652573095039 None
hsa-mir-135a 5.63273579835335 5.81862409126356 7.28598671630182 3.49325733377641 7.16157929111664 7.06730185765367 3.81742874039615 NA
6.58299808364205 4.23555506296389 8.64255315207398 6.56928549561598 NA 2.77044657252934 6.71579790707243 5.06752823307664
4.91752199122926 6.5305132510246 5.43291979159106 4.65061539600315 6.32163973576881 4.38675311983018 2.6502964592938 3.9674976218907
5.58425327829792 4.29845926982246 5.94359179996033 6.4302808137756 4.27382057802682 6.60503927289184 7.46407911245031 NA
5.37399398433432 5.64895146258919 4.79325993149583 2.95301661844719 4.94011800819199 5.86241857145635 9.96966214202624 5.53883348379026
0.267113894122437 5.55131389270335 0.590695354914612 0.555634913916292 0.895722046824318 -6.36761333962493 None
hsa-mir-518f 3.63585837595369 3.89240067813324 1.49233317652584 4.6501586329073 3.66486872008813 5.52632193685039 5.62817384150487 NA
3.7525938225868 4.51702375365722 0.941034667185127 4.79513932981126 4.14256610668038 4.46748683410866 NA 1.52629227936593
4.58609280236669 3.99345224532102 5.73568714216994 4.10980339336438 2.67558264306155 3.83479994238224 NA NA 4.92600886748953
4.44752593213612 5.66916207822759 2.81273852063406 2.80708413189553 2.48099342735261 5.41215405072105 3.10443942908838 NA
5.58490044335076 4.34075592582695 3.83357614982717 3.53450600192594 5.48176571387017 5.79294009942037 NA -0.223556238237797
4.05283003222914 -0.501414712811239 0.616847799837209 0.898995297558389 -6.37309531023128 None
hsa-mir-1297 1.63537405073277 4.36181291043087 2.14182006524884 3.64112091931258 4.37351882251038 3.86801016670046 3.68593361586363
6.06800476058482 4.89446989243477 3.49650356876817 NA 3.37403048908724 4.3947908869791 4.39380884791519 5.27450924360685
3.90372273093331 5.41468750980903 2.31220265974792 3.6013329050368 5.08790902092724 3.37202764680437 4.25729349150421 NA NA NA
4.90855727080924 4.81722546364696 5.13494236378558 1.66966827686907 2.63658982765694 5.80799396456412 4.34118803410074 NA
4.61644165255069 3.71978411566534 2.95904841114411 6.02457307322871 2.28872993838674 5.67159162254313 5.25904115424233
-0.221816515712052 4.09737883926092 -0.509560151734842 0.611137133380738 0.898995297558389 -6.38124732076308 None
hsa-mir-490-3p 5.58064882395726 1.65598520021993 6.19730509060761 6.49079819195489 5.12938043496277 4.84440679782124 NA 4.87699100958481
4.70213630698696 NA 6.48112133841141 4.15632655927093 NA 5.40106152387999 NA 5.4809053520607 2.3053830516459 2.33259440719923
6.05596153342323 5.92383577559841 4.92085567210281 4.62418330381856 NA 8.91110177966054 4.51195612155054 5.81899766870625
5.02143009202765 6.16206442332473 6.2475638680523 4.24676329302662 NA 5.38588006295754 3.97388713689058 NA 3.88832165213284
2.64596499760017 NA NA 2.71127245369611 6.33480695467606 -0.176075711332474 4.93612551218737 -0.361722154526912 0.718103205719555
0.919356179319592 -6.3885681709085 None

hsa-mir-188-5p 1.54072487409532 4.60565964213249 3.6847763449117 5.94398089084863 3.02784480937194 2.80487522938696 3.15970383450964
3.67034828578785 3.54843958082915 NA 2.37543239784215 3.25485988420022 3.97695618185414 4.73690449406648 7.72002208663848
4.16531518089762 4.91251104739522 NA 6.85391010451522 5.50995376760134 3.82590752330241 4.74485248561315 5.62578480562013
5.21696078621721 NA NA 4.62357020862054 3.66254790844732 3.43268318782264 1.96903729801431 NA 4.75745628860049 3.75005716716145 NA
NA 2.5863833610312 NA 2.52377911037339 4.56960365732989 4.91548484910713 0.179432958117782 4.11551022731706 0.382036683872957
0.7030074234146 0.917276465053135 -6.38944135839605 None
hsa-let-7a 10.6528752682692 10.4295403579898 11.108119718869 9.78736418017665 12.36028674047 11.3690226154999 10.3175398980875
13.2010030176626 11.1905679776836 13.4389309302073 10.4550951814184 7.42918492050684 9.09357919801287 11.1769903212958 9.88590320281142
10.9791882469869 10.861420398601 12.7764502213898 10.210315355837 9.76823865520549 10.6124951211706 11.0665066619779 12.6619274284166
11.3578529009023 11.1399143673931 12.6523869494333 10.4155038507627 12.2456555312926 11.2467267732118 12.2501816466679 10.8237095404657
10.8115981403539 10.6544261645954 10.0594846982204 12.86383987729 8.10520833486305 11.0968577328744 11.2896439008053 9.63664367744565
10.5589550425029 -0.252895096683218 10.9510283686907 -0.617892791004354 0.537586315991964 0.890713562955474 -6.38974522042718
None
hsa-mir-96 8.42323191566934 8.96294098349358 9.30145286532994 8.98621304311522 11.6708028212042 7.91419605637722 6.10202697254872
11.6871148283333 6.04762162204734 9.49189566455615 8.14875470981492 8.70584601772793 4.88436916942149 7.23193819255926 7.6171726911764
8.40469324832916 7.57665969278774 3.7112639685487 7.3574082317821 8.3353960913915 9.90209148195401 6.53739967664933 8.45391831242519
7.80815651866663 8.43241170569092 9.47852911588525 5.54494558730958 7.50207526503396 5.75573719331459 7.34758066600054 8.50514867931486
8.12987610891142 5.30840868399255 8.82725996391994 5.75053001213063 8.53837880902353 7.64354244494543 8.36609760634286 9.15123486988982
8.10871219063594 0.273448194708863 7.89132584195628 0.617602312705176 0.537777307157886 0.890713562955474 -6.38992197972621
None
hsa-mir-22 8.70853802468124 6.38565013448717 5.48931191203633 7.44546518925704 7.27570278910974 10.130761554998 7.21514906127739
7.65103429130423 5.03490886249939 7.31261612832343 8.13526274877344 8.4876750747357 8.10156879915884 8.29374529198124 8.29956464332515
7.58832792349407 6.30121394887424 8.60545099073416 6.18612886914586 7.78620631204003 4.7541865924377 9.92206404830478 7.30344441572632
9.60702066135637 8.68249766508331 7.06682950055563 8.53187542266564 7.79314729400236 7.05936941175962 8.62833265885019 5.91603019339899
11.4632404041664 6.99371330600705 5.93831715146756 10.8716501092845 4.88685554978541 8.68472547493053 7.03952978450729 7.18545885939106
7.41474249913003 -0.265437422628702 7.65443283882619 -0.616663692168906 0.538394690777299 0.890713562955474 -6.39049257557674

None
hsa-mir-10b 4.84968900776594 3.41867112878062 4.44140055943479 5.04846390688932 NA 5.97842120161973 2.46617808500509 6.89697063660552 3.04894823630802 7.00884860016309 5.44950975574378 6.87726183205781 3.07561884264448 4.71246995398395 NA 8.77609508936726 6.14083879880802 3.67664195092336 NA 3.93095735498755 4.5401348533125 5.90427706486275 5.36561350521495 4.25527885795123 4.69060809909969 4.59198614955349 5.61373559968779 3.55900641195538 4.93291266883486 6.18048508039496 3.19242309358556 5.37884254791706 4.31937162287839 3.70269223128935 5.15739776231135 3.80252274891019 6.38452911436497 5.29783914018 NA NA 0.220789436439258 4.93333261409694 0.496127194352296 0.620558631167819 0.898995297558389 -6.39107923898382 None
hsa-mir-302a 3.15181102716736 3.2981482272131 3.13889260429484 2.66940680903521 3.93745352322254 3.66859537016528 4.39268776564354 NA NA 4.23229196237264 3.66804075502399 1.89700519750213 4.75083855720974 NA NA 2.16041092449314 2.94752703988197 3.13726582165772 NA 5.33837062582353 1.49757841867335 4.6970277568189 NA 2.661833544954 NA NA 4.11393312766346 1.66306906863848 3.23100466625976 NA 4.31581249073173 NA 2.31966212202054 6.04801219245089 1.80407133793182 NA 5.13044018524665 2.44803705400392 4.8960691744824 NA 0.0443868391848654 3.47197490537795 0.0910273137839842 0.927603391143145 0.985980621282861 -6.39718935329388 None
hsa-mir-509-3p 7.23527023078571 4.51648782941191 4.535964065683 2.79269462753108 5.54116489890003 2.12750377954566 NA 5.87849023121179 1.66073060835156 4.07408355428028 4.08030255429273 4.25243635383899 NA 6.24704955134361 6.45173725761672 0.796112211539879 NA 4.55175909064708 NA 3.35083700689753 3.1694606801071 4.65772279065529 NA 6.49557510370196 3.3500499864938 3.29291513097098 4.00007749820746 4.95377550819534 4.41439487808618 3.87787538488156 5.91650419869712 NA 7.02490439399674 NA 2.33124138267693 NA NA 2.01429470461085 NA 5.81341985837741 -0.123654687804706 4.31349451171788 -0.24425720731323 0.8073905844545 99 0.943796895210634 -6.40588717353646 None
hsa-mir-30a 8.17699641003077 8.28855866079441 9.80348859247854 10.0903225306612 10.3883543097821 8.74591748147949 9.75603424992351 8.35787007429212 9.78145071590676 9.34267529676464 9.23630002168833 10.2101514270141 8.02477694012294 9.64056691938661 10.6769163039767 8.07333854089184 9.77585684558251 8.41868503536359 9.98607866130187 7.99897509670095 10.682853005358 8.85146556303961 9.91779448877021 8.8176880656252 10.4289962363849 8.06114922343731 7.51568441181241 8.4793246396882 9.7113199295246 8.96775549869838 11.0280515669289 9.31315220972473 9.21613485462661 10.7362658931198 7.56842919791462 10.0534369401906 10.3539778547973 10.9288071621203 9.13553321244807 9.58732400864892 -0.229091492435789 9.35321145192504 -0.590578483039485 0.55569478162344 0.895722046824318 -6.40600453739154 None
hsa-mir-423-5p 7.61386211345092 7.45250144158839 7.8053738154953 6.81190491173319 5.48244795369638 6.38219819486747 6.88508460592267

8.53680241685275 5.87487849758772 5.62370798117904 6.60871897540667 7.41383926848497 5.63653387671831 7.92897699887084 6.46129089106239 7.02164264603765 6.96087215466641 7.76026367564791 8.23168382014714 6.99668743933976 7.51799397406094 9.24093567973978 6.89420007313144 6.60391189417228 5.18822549119261 7.88215750390733 7.07611422229649 6.36772957214042 8.36733141397535 6.65725583081703 6.96717818168184 8.20067352412865 8.59716426289816 6.665850043193 6.77069920889828 7.37251325535534 7.23962019195676 6.21572105382306 5.9335341713912 8.26836857969333 -0.226895322484871 7.08791124518023 -0.588770526173957 0.556903929035579 0.895722046824318 -6.40705494551458 None

hsa-mir-532-3p 1.95499940051537 3.60701609711019 3.3058411844637 5.01972884853496 NA 1.8671927642875 4.59389457060736 2.98650419638461 4.65051268507444 NA 5.06236839719272 4.29007888567988 5.47261964801301 4.06825951469476 NA 4.24263374027326 4.07124609139042 5.62077497337812 NA 3.13171926958048 3.26506861494244 4.54998875222597 3.64065180986163 4.74471366423584 4.61899776663863 NA 3.4771685780511 4.10716712439126 3.86501929295329 3.75565197763747 NA 3.49362917530889 4.05620261488282 3.39291824187875 4.02171633747502 3.17644327143599 5.94972614980207 1.34936691728684 NA NA 0.155059998635799 3.9190568923809 0.350657608470024 0.726366829717168 0.919356179319592 -6.40839452896423 None

hsa-mir-455-5p 6.68591974736993 6.66425649494303 4.47377078767426 5.36888446663807 4.79775853721391 4.18102572654708 6.81060604575927 6.02391392751639 4.56906335435337 7.03329433692308 6.0921941383472 3.94108110912707 2.54541734746323 6.79709879616711 4.45657410461629 4.80127363345138 3.964417142303 4.48835393757447 6.23529558424144 4.64254675804664 5.84222536738963 4.96234826027653 6.23279850846716 5.09886750406591 4.67756868829026 3.7077572002732 4.95090824228158 7.27397621089355 6.59986598995923 5.92286598657741 5.52428587454722 5.35926036723262 5.08945573886685 6.42900235068687 7.28681973277555 6.75842574676065 2.74163024712362 4.4538836044278 6.07696167483272 4.21951823186724 -0.23178397756597 5.3445292875968 -0.573922401254666 0.566882932761999 0.895722046824318 -6.41556031043733 None

hsa-mir-15a 10.2419757628147 9.4541802053104 7.06908832766627 17.4977921178217 8.81395609067971 11.1083982190234 10.6046125081273 8.8823999879227 13.5686570386815 10.0166586839341 10.0747776864131 8.89460394746955 7.68098693242105 8.48673228158543 9.92098725418136 4.56322758842658 8.38368557592757 10.0288614104833 6.51512302817969 10.4017421916208 7.86780034628539 10.2710653098207 11.2418456152327 7.95470058038198 11.3438676858569 10.517453300988 9.91645770259991 9.14041361803318 9.04215306325312 8.06730642679181 9.37918170660944 10.8696439734286 8.58776308966637 9.52047756829106 9.10512892710136 8.5013511456136 10.290708604 9.53347564466764 8.71639338949005 6.83157729825588 0.27548409211613 9.47268029587645 0.570830382054976 0.568971832175591 0.895722046824318 -6.41730428813344 None

hsa-mir-484 4.69745646909888 4.00262108159448 5.60630452936801 4.19772149803774 4.60472083326782 3.91018167500684 5.23185919078736 4.52230701482301 5.98951391148473 5.79156288541858 4.16152263432601 5.54049232751585 6.7987241994101 6.28612805849006 NA 5.05302742410885 7.66734395363079 5.51012704819538 6.28036503124845 1.1447440997995 2.74214412459289 4.2866311758779 5.68287333944454 7.51203966995252 5.34677012244789 4.19548044298681 3.12325223749165 6.03629486269468 6.62213734527408 3.2495539943096 NA 5.34934026534536 3.53904218481205 6.13179124313384 6.46381580341061 3.98548677415243 NA 3.66294383665904 4.71226583956023 5.46062167197314 0.210508233429116 5.00268131891167 0.484881299989913 0.628484921926124 0.898995297558389 -6.42362052387659 None
hsa-mir-20a+hsa-mir-20b 8.87701913522318 7.86191454317309 8.67511869188115 9.05840976060227 9.14375443016302 9.73919386751751 8.82944173729958 7.4100489002779 7.98109020088951 9.67991779168737 7.71642561513532 7.21158287301902 10.5390208020527 9.96262248127276 8.4515486875653 7.61390250273789 8.7978532799618 10.0530290047106 8.90773480039007 8.74140865694972 7.91871889836629 8.01161981914298 8.21342126997213 8.16819797355464 8.46236036271707 9.68873346652882 8.47142157867913 9.38501646499748 8.91476448682511 9.10728743269115 7.20470293008905 9.56036818283524 8.44720525149001 9.13924078452583 8.14010011765014 10.4633555784057 7.38829592577449 6.3260996440571 11.342645914619 6.5261393813422 0.218567114912307 8.65326833066933 0.553147638546423 0.580988706057668 0.89755603144822 -6.42709758886347 None
hsa-mir-130a 4.07847103306832 7.53745774876703 3.34749771834559 2.63104915405375 3.58761983834328 6.91100415227272 3.45555453988104 4.68852255557726 0.926263267345536 3.70842108879618 4.83801109127802 5.54977059518711 4.19580680559154 5.40662170707864 NA 4.24383255211159 2.12554843794559 7.35198213641364 8.23727570731189 5.61556778510503 5.3427029610053 4.55016185908358 4.14985593229886 5.62834987800313 6.84480125510085 NA 4.68830130724104 5.65291268445672 5.67647249544843 4.08221198416441 4.36714495638005 5.55876725237488 4.83685982523219 4.31836713328052 4.86204562928161 5.62331714753277 NA 2.38671859599982 3.46675398875493 5.51426590214779 -0.209348545343346 4.75638618114218 -0.467260729443786 0.641007728669801 0.898995297558389 -6.43188048338433 None
hsa-mir-376a 7.16217655530533 5.87646283675997 4.73883078611213 6.13291964721849 7.42056552778893 6.45458420593374 6.13278262420736 7.3070384994502 5.64570230928694 4.99621622189816 7.67625021069644 7.33577102352926 9.25332949487264 6.07747035099601 7.39640740177619 5.93984079194155 6.78573079255378 6.1333926810816 6.34583841190122 6.99429360297793 6.3940569972154 6.71996746479572 7.47614255090985 7.95298720883421 6.17088846588387 5.32676949900954 6.67927560311529 8.36973527655401 5.96082499214408 7.68170502192378 4.17164208394443 5.29578863273418 4.91810308426804 6.48218749440526 7.13502875589461 6.89726585028365 6.59125328143914 3.09526293825578 6.88121839526134

7.24627706615822 0.217961165662873 6.48129961598296 0.543980464766455 0.587265422980173 0.898214641126189 -6.43205390098801
None
hsa-mir-15b 8.78052774722549 5.89231363860965 8.25468503942531 11.8034134557063 7.48996883283948 12.5373872080754 8.00594987417031
8.54437299237655 7.07132683207659 12.1437154760867 6.23540804648415 7.03710413382621 10.6947070655895 8.64396366417822 4.46365660367499
9.13952329090425 6.34638276751939 5.79952399126823 8.80388840679549 6.95139327427258 7.20898668239503 7.7272243495633 8.45732717582163
6.90375868348578 9.0593189495654 9.75027262316417 7.37856652343587 9.13782396777276 6.68012141519483 9.36228582516936 9.96131301576662
9.85407787422246 5.93223590082953 7.34635400588772 7.30185569270587 5.77204591933344 9.16054560576136 7.83252724686298 9.57410960650898
5.33341772159854 0.24525217780296 8.10933452815376 0.529420654060354 0.597299099375411 0.898995297558389 -6.43975622114799
None
hsa-mir-361-5p 5.30092428905518 5.07743184989328 6.2044570042439 3.08100051298354 4.65421845128454 5.25098079727146 7.61048504896922
5.94776856310108 5.39854578630722 4.44296099901304 7.51950309996557 4.07740902117205 7.52690069321545 6.26904926680495 5.50980314900367
5.91805007846911 7.79346866613836 5.19566925267094 5.14910016117877 8.01107640591426 4.03350171546784 8.55623493452335 4.95740828191951
7.46891688246106 6.78817118821876 5.54668939198385 4.64292198130322 6.56544722750779 7.13929308568557 3.91052639046467 4.36046130625065
1.97381790151581 7.63633665040162 6.50095847280536 7.02331148131217 7.21647166384835 NA 5.67200361965696 6.46360156459572
7.76166587821159 -0.214519825069001 5.90144981319973 -0.49431099764998 0.621817142570791 0.898995297558389 -6.44479032089786
None
hsa-mir-9 3.77758415436374 6.94916361794034 3.93259471453672 5.53743875300304 NA NA 7.16199067008468 4.85309584168653 5.11419134271486
9.16672035711334 4.74417405922249 4.03142668579402 NA 3.41047142721199 NA 2.90466269498911 4.22455299621837 3.55887544704595 NA
5.40383029405226 3.65162452638786 4.11367246611062 8.12123090886684 3.10504042303103 NA 2.08376419660637 4.12835869090221 NA
4.74799803207027 5.82863976286437 4.49959990310241 7.81365288709855 2.39231750355097 5.21475835470773 6.63182671593742 NA
5.67762369761231 6.42205562881099 6.20161750608126 NA -0.0548931215019959 5.01305013741028 -0.109094668200254 0.913283093492844
0.985980621282861 -6.44697362025871 None
hsa-mir-150 11.9466692332731 7.06971162317573 7.33226855042103 11.6754766636389 12.1624942733634 8.01894768744692 6.88484059265944
7.14968519057117 10.6091401569028 5.24428524541782 9.58344697044317 8.76426472662659 9.85839923633487 7.21431073044672 7.8806251748035
6.45012888277057 4.94883556594803 15.250959814073 11.909826027538 10.0295434252691 7.70790813596404 8.44099362560218 9.2257353994274
8.96887538162791 9.11553598677379 9.17011663463202 8.32580249209274 9.45273400876518 10.7535869964123 11.959048882669 8.0256946974298

13.3969702389987 8.28148558245005 6.22647797522771 9.30096429402566 5.32640175286943 9.17960664457213 8.55882247209959 5.78756696622195
7.54919161983375 0.261516999171425 8.86843448897048 0.511203306806708 0.609963002500904 0.898995297558389 -6.44910024325368
None
hsa-mir-1246 3.34264956989985 3.7412729327349 5.84878951684674 6.49051739292636 NA 2.37872075399563 6.47721359843016 6.84790715057295
6.02667078358152 8.24748567665725 3.31393310616536 1.24970189058269 5.29136993217415 2.85284364743719 7.13433540775769 5.35319464850197
5.99502809160943 3.47565371989385 5.21614291323414 5.01251754065204 4.89469995333722 6.24977556617646 4.31404739385691 2.32303901261295
5.27302138235994 8.04858575002928 5.90853059370109 3.93217905145874 2.50463295773252 7.05106324732515 3.02448885274235 4.6804698451389
4.0873006849022 3.18203420483693 3.7911961322367 6.99563033973704 5.34174561158349 6.67875080094435 10.890616873632 4.65819421216209
-0.228555477343535 5.08015258308103 -0.477957094225831 0.633383706045391 0.898995297558389 -6.4526257344652 None
hsa-mir-542-3p 3.64688713059661 2.88976308327721 4.56069067366833 3.06941435050566 6.42101658748123 NA 4.41065896946624 5.63217933598208
4.57139176976884 NA 4.53984179336436 1.65210305870933 3.24126063065821 4.18293426284209 NA 2.49841953358041 3.34796381680357 NA
7.87590045796748 2.8701924254682 5.59971727184821 3.82477526367562 NA 4.12993438120483 NA NA 3.85960313688657 4.55460436741812
5.19424427731104 5.56694724621764 NA 3.24954190239587 2.22951843051936 2.99963194680127 2.59592006535819 3.11549278425066
4.77030101602727 2.39000155352106 4.23710502644247 6.30109875182079 0.0495112786525542 4.06340797818246 0.105797233843183
0.9158930973331 0.985980621282861 -6.4633197586012 None
hsa-mir-154 6.58900296822033 6.08344072572736 5.52662910602849 4.67103524710506 8.59175580467291 5.32312767869863 2.99187809405821
5.55522082817863 5.26744364123835 7.65241878494055 5.95852081012277 5.19710781689656 7.47027535641871 7.5933344615657 3.96186878949596
6.90632377067475 4.38002626139063 5.54801617337839 6.5604374464069 7.18432766942844 7.59845222371456 5.11807682881857 3.22338897408159
6.81754694963204 6.89856762847569 5.78618407434223 5.9392084672206 5.73657424744061 5.32985239799852 3.29278446995025 6.23514972184687
6.00323302129778 6.19064503271312 4.13554796329187 7.77552094819466 6.21321260007398 5.9763021607534 4.68855875532161 4.85010327245818
7.24206242703361 0.19806096349938 5.85157908998268 0.478907760931196 0.632704101980058 0.898995297558389 -6.46486389075478
None
hsa-mir-592 6.75453327348334 4.77514171466079 4.02135396349238 4.99986176696212 4.93767527707828 5.70774200857562 2.91671247331748
3.94884263343321 4.6124231208537 NA 3.7138430729504 4.69077431534553 NA 5.21007167131199 NA 2.03785242280865 1.51047989849999
3.68764898308803 NA 3.48596182382687 4.1299159293914 3.26681551960073 NA 3.8095647227889 5.79558088221193 4.44518615205663
2.46201309662185 4.651549983821 3.58753490423775 6.71927347200744 4.24977087363072 4.26630622422957 2.67762985631969 4.45253118995028

3.16305084659392 NA NA 2.92920696959051 NA 6.34920948560767 0.00348614443927708 4.18643932901089 0.00760316956041683
0.993944317959776 0.995862509864406 -6.4687943084286 None
hsa-mir-377 4.343794640557 6.48269261384791 7.62531697981655 8.0858429511498 6.06777899822993 5.62437447838116 8.20337227860047
6.62554212606935 6.62044913868095 5.15712294035156 8.05102186005133 8.00117889924255 9.81405851253561 6.12242806863068 8.20483879687043
7.78889778910226 6.35715908389122 8.06617937166149 7.73263467775242 6.64609807297197 6.86418665041217 6.10943558937727 7.04148331947432
7.32385920194904 9.07484090714824 6.7878934795946 7.61886471375373 9.09054748211547 8.01846850234293 6.67125024023757 8.10799769787765
4.30536308056515 7.75718182249839 7.40718993279276 7.80244765492747 7.98904844235973 6.58695513467481 7.43925201346067 7.46632777675848
5.92104374286885 -0.188142755339736 7.1751104915896 -0.467287254272633 0.64097487516869 0.898995297558389 -6.47028514033395
None
hsa-mir-1283 (+++ see message below) 2.69366692527296 5.50356602350754 6.00924735118905 6.45427414868683 7.42010194014862 4.08819585810872
4.88210901413279 6.04381135122068 6.75920485328254 4.30606819653645 4.3454459947077 5.2515238306335 6.91948357608161 5.77524320889581
7.79692482361861 4.9505048313255 5.0730116452307 5.01628315136641 6.29966843592126 4.92098441375606 3.28014887868316 3.69087736558648
6.1398599034858 6.53526922533525 5.66594353666113 3.53771506508824 7.9924690294677 7.531203211574 3.71443708472474 5.69708573448333
4.60662136331109 3.22126758801831 4.5003682964249 5.97704396774946 3.71736712777007 5.94643391248657 5.20421238334806 6.23389774711632
6.4115291272206 7.13809150839035 0.188373875834889 5.43127904076372 0.4530182703041 0.651192503122866 0.898995297558389
-6.47676035880094 None
hsa-mir-136 6.51377702851985 6.45289022526113 4.93942412753258 4.29328573765485 5.98539126337461 5.25498025619427 5.58427314724179
5.27215280924958 4.7717395341479 4.51277128149448 3.76047058902658 5.24280403990568 5.53280030323515 5.45202913001856 7.30767404925588
4.84158239606488 3.66701574299014 3.81868092682578 6.17422129981574 4.69131917462244 6.21036861648098 3.5653189323869 6.43217451214245
4.58496354567497 5.20266455781361 5.7883020200229 5.71469253713481 4.32858084646816 2.88825981288326 5.0494091412887 7.11493422179965
5.62117151855595 6.87419073408792 5.13025560067867 3.89145305397552 4.02965961605738 5.19369656735657 5.92730688051552 5.5547134734158
8.48433540378576 -0.175858426504679 5.29139336637393 -0.439770939604383 0.66073821895082 0.905511695516692 -6.48259270848186
None
hsa-mir-142-5p 6.94136847609179 5.94153587467736 7.73233950521996 8.61141385271372 7.74596610069498 7.07039000669647 7.34700253361166
5.42303988057808 8.17536619942238 5.24861695114233 8.10826234875374 6.0702790307858 9.30936425228599 6.39882814070659 7.10353153822641
7.53656663631982 6.65074830433274 5.31998928833523 6.79461588558717 6.52093628969543 7.92748705568931 6.12258720174872 7.67516734490923

5.29646611247271 6.3965596287041 5.58496507690462 6.99523119624671 7.0192988782193 8.64067296601729 7.97969873691521 5.93879658163649
8.90743678550978 5.9454193497702 7.06657175562235 6.48356099535657 6.82972874036356 5.41175902241573 7.23805801333415 6.23327236338982
7.04093810153996 0.165824259455591 6.91959592506609 0.421582049363513 0.67393578101244 0.911469750803578 -6.49031941220357
None
hsa-mir-151-5p 6.93156572986322 7.661599803044 8.11384558341965 8.73519930799262 7.23960117465375 7.62523523643596 5.50408310780899
6.21451682971653 8.78837503554766 5.69989826047184 7.07668680377744 6.05980950397186 6.42471674943531 6.68888779300012 7.87904834565789
7.09742354641745 6.8404897546895 8.15229495810247 7.6004965152152 6.8902111579385 8.85985948969262 6.60915846199596 7.56799741296929
8.34940210054231 7.21274696460482 5.56298684826039 6.02502715294655 8.28366168251489 7.35140934830717 8.54788273639543 8.32588283347528
8.80583998997703 8.54892312473007 5.58673444016703 6.6832710077586 6.11123647943368 7.94116269293838 6.98619008962975 5.92137928098301
7.10363846158258 -0.158020270087246 7.24020939490162 -0.404809358830714 0.686196381748199 0.916375046098117 -6.49715599501699
None
hsa-mir-30c 6.75208344462744 7.09914551482615 7.81316683016402 5.00182169287477 10.153161431066 5.92304701109803 7.74525661208569
9.37140502940742 7.4721227128669 7.26451561231932 8.55996784331888 7.38094623293791 8.9357700997079 7.48238451434358 9.87550083019698
6.10651861341938 5.91769792228174 10.6985902299943 7.78573827405294 7.2591346742213 8.97209272197992 8.20259818972116 6.86519331125506
8.35755993262808 6.59287945294106 7.90842049140831 3.96001020267757 8.87802295273231 7.98974110740011 8.0201317635837 6.81517536625987
8.43869679665109 6.30838211824124 8.17599236044243 7.95833917745529 9.03498834036656 7.45879893649649 3.74286102490868 8.96691520102818
8.49439735489365 0.172838916136997 7.64347929822203 0.403848453864297 0.68690136125431 0.916375046098117 -6.49753927613169
None
hsa-mir-574-5p 5.1871320538652 5.41612369850265 6.61867100643616 5.69153573420188 6.08865989096458 5.06232861214504 4.46689554222962
6.71965007779023 6.33550807004987 5.43431010435703 5.95304846988579 2.37472130045263 3.20598071697257 6.74810644940291 2.98080805548546
4.32598437885824 6.91791040544599 4.03365212718741 3.89465760117416 3.03781952054591 5.05836411693759 4.45205434466572 5.80636690054429
5.76204455067202 4.81234525214009 4.00478626572526 4.65878731517041 5.40040519091086 3.95900591070981 3.68976969541971 5.73501338414575
5.37650039832504 4.26789439104848 2.81666604887482 5.68815607389251 5.70681126019991 3.44282981869182 5.160936821595 4.76322626908331
6.75953707309724 0.158600136705185 4.94537512244507 0.392473619864903 0.6952674023086 0.917276465053135 -6.50200735223421
None
hsa-mir-485-3p (+++ see message below) 5.82663140012073 4.54824948170143 5.36894661872403 4.83243401342738 6.02258215784197 4.47857209560611

5.79669303490491 6.52847214449243 6.82311244494627 NA 4.25358934307245 5.32722753580292 6.12072799091922 5.51170638948097
4.83658637691686 4.62545719526766 4.70690360056863 3.68400139792695 4.75876350898757 4.78392910687286 2.51036840006812 4.35565593624123
6.56216789517792 6.27723233459679 5.45366614249586 1.45759110600511 6.23658534675718 6.79410658221073 5.51034403820971 3.47030767350629
4.84787641849539 4.77119337557987 6.35776933910685 6.03854218477019 5.64660061271433 5.32010334274847 NA 3.64099232994815
8.06916812692729 8.06898104876809 -0.13445612614454 5.26904837031339 -0.316234298792795 0.752271835155051 0.923995650122037
-6.50356212316907 None
hsa-mir-27a 8.34921000950473 7.02400208817473 12.4068686309416 8.70187473097167 7.05950715932908 7.44882791663235 6.2267554673475
7.80934168197156 9.04733957258484 7.79439691951986 6.09312773917582 6.12716627502544 9.11197670313442 7.84450201127967 8.03321142602607
8.5327268503204 8.67027311115587 5.24635272983515 6.94396401855614 6.19885161240602 7.95496609573904 7.13208077665987 7.75238205868888
10.6828696795651 8.29524853232131 7.47337791141886 7.58710411486397 8.53340498672277 7.01938982651903 8.70620940264521 6.95919400141605
7.0302240228625 7.906083188955 7.64148002508196 8.25961139222923 9.9423585456272 7.37379663574154 6.40482557483662 8.69113910440905
6.50294596725356 -0.15892075948319 7.81297421243624 -0.383905689699599 0.701593908795725 0.917276465053135 -6.5052887572899
None
hsa-mir-331-3p 6.07553839607347 8.23197294676072 5.12316965773348 6.87704005955399 5.12617675529941 6.61668564181551 7.14587754305234
6.97925277178466 8.40560730506787 5.46140854139183 5.78722139562905 6.35307482343187 6.52141991628678 5.33368653821845 4.18545940729451
6.65055103431044 7.09539754082617 5.65055538430839 5.41555449540802 6.32502631764802 5.31197161195875 6.75623307721114 7.41343762691012
8.0852185496176 7.99130149850072 5.14438575573211 5.70037550571443 6.36637323884721 6.0783466752367 6.06278513858888 4.05563088865612
8.69746937478554 5.91868746389982 7.10716345587961 6.18045202622897 7.09438359453903 5.96117640336296 6.52042286839771 7.05186291851409
4.88054987904847 -0.150877553986748 6.34347260058812 -0.381181587569646 0.703609779859474 0.917276465053135 -6.50631690913042
None
hsa-mir-320c (+++ see message below) 4.78463205665471 6.55170857750117 6.62362601782747 6.81615601454383 6.87581528311483 5.69030070991959
6.25238554430016 6.20589356659234 6.12496402816765 4.40405405758389 7.39849523196577 7.20143435470902 6.49843265708008 6.32931279875183
8.10944959359895 4.91127752724875 6.31883433786116 9.00335978567409 9.12650714910865 3.22718694261104 5.05624729294879 5.83338254005185
5.30514998886078 5.63191373728975 7.27283237688906 8.20673016700953 7.15158384777985 9.54628913531571 4.35238047479843 6.37859765624992
5.74490421921356 7.78819433229157 5.7871694352541 6.02644071361343 5.49367388757188 5.46123946849503 9.6436203942615 4.99542217643546
7.04667247157723 8.96257182917607 -0.161559495513428 6.50347105949746 -0.37803043185557 0.705944302776452 0.917276465053135

-6.50749712772064 None
hsa-mir-574-3p 7.7287654139166 6.90113552684861 6.65156691751207 4.91587068909284 4.55777848528686 5.34000893454345 6.96904534973649 7.67261407489035 7.20414785874472 7.22173089170183 6.84811733048777 7.3384462030527 5.97775894092508 5.41157683423017 6.98286992976943 6.80247529160549 6.78335597498615 8.22722347344574 4.44876953461595 4.70490729679599 5.77012885589648 5.26552921713184 5.78527387177813 7.39322094769428 8.584013249249 6.93667122183665 5.42613474573291 7.34050827386974 6.69789073135792 6.20345224220818 5.18556814395143 4.70875404798776 5.72440433704817 5.58305238525261 8.75561061607288 5.64259775597884 7.05850355712934 4.73505790512652 6.03901494216441 6.92981886237977 0.146147952117071 6.36133427155088 0.366084593420772 0.714819581227776 0.919053747292855 -6.51188246405455 None
hsa-mir-34c-5p 4.60189403424669 4.84457068750795 4.5321246049318 4.73783833393728 NA 4.03645489600059 4.25262254997179 4.47111565337101 5.11779351375426 6.49184290046338 5.0950822226547 4.59391247990525 4.89931247408492 3.59018290113829 NA 4.0253855479331 4.83145262793675 5.69305405251159 NA 4.80860669734912 3.4237544429176 3.54095633875943 3.58438548470783 4.09471193575657 7.99422885601482 4.82575375492438 3.5412875994256 5.49607093240946 4.39571111213191 4.3889170471481 5.76175981553816 NA 6.72586275135323 3.68114411427512 3.04390848061632 6.10201588026153 3.83291316602039 5.78887469860691 4.9626487639397 NA 0.0100491608720663 4.73737575292873 0.0237645349797239 0.981073221510537 0.992203913441046 -6.51193957616207 None
hsa-mir-452 3.16846206785654 1.74900727296999 4.33503014285621 2.41743611598566 4.41767539525271 4.99807690750271 2.5680351069491 4.93512837599514 5.40838425528918 4.81647746780259 2.40990320146205 4.26426155470779 5.35597232018377 4.19090481956295 NA 4.38556185683195 2.62060700505005 5.60951258823093 NA 5.47970460191476 2.46538300122639 5.80854129362912 3.85965231667911 5.12637262395202 3.793575481141 2.90568662925542 4.63500593717973 4.41080854412358 2.38686785044056 5.86052536879051 4.24576874630925 6.84257841500953 1.67730172816221 2.89641330743569 4.89736103018918 4.90282557664263 5.61336915768098 4.27808288740339 3.65628926567217 3.29420594283638 -0.115045140943269 4.12333568842534 -0.272815025053343 0.785377060573222 0.933778355913257 -6.51481543375413 None
hsa-mir-579 (+++ see message below) 2.8248876611313 3.51549316972045 6.02241157718468 3.77383338913181 7.08986131532369 5.49508337082245 6.65813483300946 6.50740082361619 6.16358746559235 4.03034833392475 4.51541205612817 8.14922255795501 5.28246768579467 5.01262060583055 6.92909576419277 4.49116038621789 4.35892128824656 6.10975907334243 7.97505235968971 3.23899067446633 6.68969643443118 3.80100480902559 5.90574233007941 4.89687335057909 5.32000522175405 5.499235535232 7.14125806572729 7.36610753701801 4.46685439459521 4.61618102439165 6.03821502754013 6.49354127839459 3.50585416383696 6.36384812744589 3.17216099529002 6.2808324498644 4.51503134157589 NA

4.67675597750311 8.38811430055689 -0.126355536478221 5.46874504502981 -0.292995630749859 0.769934596801124 0.92518658980654 -6.52293288686708 None
hsa-mir-301a 4.42699339823639 6.7259466329996 4.46240641785877 4.96854202793746 5.81758580030156 7.44195826927855 6.24012713612295 6.20800084606846 3.98281356430101 5.67394004358245 5.33165245778423 5.36312257104237 6.64963968597502 6.97787126454327 6.91397877467259 5.68452729557323 4.84128539707518 7.76072746808792 7.44213794131333 4.68025315500422 4.61938222664912 4.82033040371988 8.53665858853372 6.21258211610595 5.39562698580241 6.87861238030364 3.91293557160066 4.13210773130875 6.08288998786343 5.23497665180592 5.76031658007445 4.95455577033942 6.49489441320695 6.22354420521695 4.90620788155218 7.84751956503457 7.30834829170263 4.86252290740315 8.6194470106927 7.42985121094437 -0.131990016605115 5.94567051569049 -0.323039149355906 0.747117065991337 0.923995650122037 -6.52651864012514 None
hsa-mir-600 3.84953636184511 5.19126537049023 3.87608189906892 4.16804947352167 4.70825113768064 4.6643361892089 3.25129471923165 3.44733128883385 3.47721538425601 5.76058442711727 3.23708679225889 3.3709098843988 6.17553264982047 4.21182366737091 4.82621035654563 3.08922802733665 2.77983425240403 3.87979625664956 6.00098649489971 4.44948899443363 2.08156825483961 4.16876843814825 NA 4.77596501014422 4.86058807285278 6.20779243610073 5.5676540294023 4.47497562746306 3.91765266223145 NA 5.0366249804865 3.81891592964524 3.44525250911285 5.76554243292397 1.4521710173171 5.05523955249847 4.48358699595753 2.30161594696034 4.99672186228377 5.19269499249921 -0.0905539714573402 4.26363616784842 -0.220572038558859 0.825730750934507 0.948410748216205 -6.52752611978417 None
hsa-mir-30e 7.44506014435395 6.59141604956851 7.84388065716999 6.09256509932111 9.3960164536884 7.32437386610287 7.24816459734811 8.19156078712118 9.88128693216481 9.88420171401652 7.76341531097143 6.47716109973457 7.5930687638439 7.08874392735279 8.99748314059267 7.28812514868901 8.43618733925706 7.27399790412978 7.69408690367488 5.85111883859899 6.27937394989419 8.21760712443924 7.36239375452873 6.46089919539532 5.99819559673601 6.10684049927961 8.22300345998848 9.26381858540727 9.37326695357912 8.55884449417699 8.86531671396411 7.25119388105443 7.65440760383598 9.86416967627518 6.60098554846654 7.65085714998311 8.13381220358185 7.41604389416429 8.70060597758416 8.8805803186005 -0.125015095161731 7.78060328146589 -0.313058544754393 0.754672577661387 0.923995650122037 -6.52965147252088 None
hsa-mir-193a-3p (+++ see message below) 3.88390018609581 4.5820095081598 5.19038112658764 4.83504479409516 5.33597116045648 4.62336258960346 6.43713297457548 2.92288678061857 4.69036155228988 6.11315817256861 5.13476388825819 4.68849517595273 2.6445650126557 4.90166562533888 3.10658890536208 5.9049196214017 3.70778360049426 5.00783753812985 5.19835081581278 2.57290029941308 4.39937490046575 3.99552356342369

NA 4.26436619500829 5.92128319847592 3.04225718451746 4.55609405706839 4.17681501184888 3.95375446998209 5.40561829188943 3.62144856458909 5.2527727820721 2.99799769577236 4.39744234036892 3.51726988085887 4.37696465886645 NA 4.87551851587233 5.05555413886669 7.04813129828428 0.0819824803806739 4.5352701598974 0.202504430786288 0.839801505968625 0.957278465215654 -6.53129595995296 None

hsa-mir-495 4.58164735017108 3.47681867606687 4.75569867740783 4.92002729756087 5.09522244166707 4.46134649888964 3.88707657618909 4.19312584646311 3.34063544800954 5.7407660337924 5.25503428924657 4.77579548394187 5.05559818747612 6.17314148583284 5.89447332743128 3.29230780175557 2.68092104983898 5.26736117435618 6.99824756030675 4.17822297143983 4.54191655535824 5.65204062555017 4.40356665996944 4.6115939416285 3.73376941563909 5.03896786004407 5.93189131458082 5.46914201804952 6.01937796031512 4.7412110720182 5.00876391460989 4.2545101831326 4.68038688579227 5.07279839110246 5.94691936699631 4.89985944447834 4.38102834582327 3.9396897598795 2.86082986990582 5.11954079858022 -0.114216810280518 4.75828181403243 -0.296514369608495 0.767248903807649 0.92518658980654 -6.53462828848109 None

hsa-let-7f 4.44901663732502 2.02376531229564 8.75360830269974 6.32387705510568 7.57764464493499 5.09559003683428 7.39561344988585 6.99267733454536 6.23478368251576 6.70065295864562 7.13921582061657 7.76841695660035 5.43673758918899 11.7939201835014 8.44378171158515 4.52374694954984 6.60427369084391 8.23801431009519 7.070221972763 5.42163153324309 5.97171338087806 6.77412319091809 7.76440394893153 6.18359257200659 6.56636201635862 7.67244262800414 7.54807177813803 7.63448175261571 5.67430844877314 7.95592954991138 5.51390136564945 4.08413142372862 7.90050764234067 9.20336968001047 7.06068107255527 5.33210236081793 4.92295873165541 2.97010340538403 6.91859990876546 7.65772645475522 0.133883941028881 6.63241753612433 0.295304646564054 0.768170974165966 0.92518658980654 -6.53498161126927 None

hsa-mir-99b 7.30981552320563 7.96956818675712 9.25305204960864 6.64441512555108 5.02804733751497 7.77869049587627 7.56323106688286 8.18779842051902 6.90918501811277 7.20223403736192 7.74405767141565 7.92360014998497 6.78203485636724 8.35461594696616 6.90498281926796 5.38739833387403 8.56400998307431 7.38460604989844 6.36776474941543 7.57150797059915 8.21469992292161 8.31519498221338 8.93408390026469 6.93460426551313 8.19663308675975 6.21888208749824 4.94486712802832 6.85517697003317 9.36250816597885 6.78133870226648 6.82097098866841 8.3793368287665 7.18106656003656 6.75985345510523 8.80829888353268 7.81627390381312 6.83276309669484 7.10428374431156 8.18831703429275 6.46174621323975 -0.114014206384271 7.39853789280482 -0.290605361971108 0.771755977898324 0.92518658980654 -6.53634043954666 None

hsa-mir-590-5p (+++ see message below) 4.50003530972779 6.98108876666738 7.22717732188583 5.48684802690423 6.96223208465754 5.03790368752085

6.28576329885022 6.29887557934722 5.48230358097032 8.80076518966692 6.09894655750339 6.10301536781838 7.01379371125908 4.6794953329724
7.01535760447616 5.09547142499747 8.37302153897399 5.91459285636862 7.33904748017467 5.98261066468477 6.64039504387692 5.52255066255621
3.46556445993083 6.09018636100949 6.73704100798628 7.30482871516731 6.24111231294408 7.50780825446311 5.27756995158807 6.85200413973225
6.95578191199047 6.1515600008742 6.70277819773203 7.48181305693208 6.16162116826133 5.37976346211666 5.33261430684574 8.3157477543383
7.00781761166698 7.79465573138308 -0.112243436298409 6.39003898742057 -0.282349281389827 0.778066292999594 0.927221293831532
-6.53867501089329 None
hsa-mir-374a 9.13310914297145 8.2751584994988 7.26271629271288 5.78195505822702 7.73311664497816 8.76228868592647 6.9135075844173
9.18341629308255 6.95925877980916 6.30325762822787 4.61650576811451 6.58837037057207 8.10458530590672 7.67857764004956 7.92269218181659
5.84950239523532 7.49166350555896 7.76377929973577 6.26179137474957 6.75397490471818 5.72399245118998 6.78391692329675 6.24394407416164
7.84337400086266 7.88674957213366 7.39983513343126 8.83174962090574 6.60128372675874 6.80810386370084 6.83298333151195 6.48763861848054
8.27552200465551 6.95991966112162 8.71816774441967 9.10924649572706 8.10217147496626 9.39419872446877 4.51825314319059 7.97424390923402
6.9117462097039 -0.103390666380613 7.31865670100575 -0.256581604171739 0.797854603048696 0.941293012855703 -6.54552908625138
None
hsa-mir-1260 10.9718319289624 7.68116840729642 8.78959872755732 7.82110485392022 10.8672586804714 8.99622406011829 7.92797646337499
8.65973174764235 9.63044820057302 8.949753834826 7.5112570906162 9.2368137684845 8.75526025551369 10.5674086145684 9.42347523486951
8.64792876022695 9.84335348978079 8.43677061590769 9.78012536740389 8.43492444461682 10.2377292130033 10.419319334599 8.24877630786653
8.61011062210083 6.17529684764609 8.68343928495248 10.746867205319 10.0161635796612 9.26786927818709 8.86788024840224 7.06076050563839
9.36597326169203 7.44177708930877 6.65849389899466 8.32669309306108 10.8625508392782 9.12787621745711 9.63881395794614 9.03143799156495
10.1133507116081 0.101561752922184 8.99583985087545 0.252759267781248 0.800801518399628 0.941293012855703 -6.54649003757855
None
hsa-mir-92a 10.990699635881 10.029206987941 14.9341875460139 8.72659042992197 10.2729697583193 8.87635792719936 8.50741815805412
11.6714444523914 10.6670973767423 10.4139706935681 8.98730764554303 10.6081823478747 14.2276977256815 11.1706865314653 10.7806145127786
8.2962270262638 8.62281582738719 9.57549467839491 8.05792083303704 12.4031874823005 11.1304393441192 10.9935287233255 11.6018001038636
9.11284075987575 10.6061363222105 10.7626540362807 7.39771047226247 9.0865586436382 9.53700954137427 10.3340658809976 11.5624666774021
10.8401997895408 9.50724952238786 10.9761703816335 10.703454099351 10.7303303004689 9.2089267015275 9.86835788300854 11.9956939662216
9.80896859392989 0.102775791666978 10.3396159830045 0.238409669011449 0.811889906807582 0.943796895210634 -6.54996898543052

None
hsa-mir-125b 13.7554001319632 13.1106384629948 9.71028819818481 13.1309423015386 11.6834569852802 18.749890060635 14.3678440120788
14.0543656228817 11.9995708869229 11.0856490919415 12.1961079665041 13.1998998757452 10.6170566155398 12.8695867567326 12.907390490765
14.3445839349687 14.0138826171643 11.2250415813109 13.4885667939263 12.4326701252119 13.5928049428972 12.6101051654923 15.2139929268641
12.7391787516722 12.7997667401554 12.3607172969038 13.7499547517038 11.0760405108316 13.7358508884723 12.176367969645 11.1032563624474
13.6147197017272 11.762877568553 11.7669407952336 12.5622522321059 13.7252296346088 14.4758409508214 14.9965463330706 10.9048569312971
11.8967475018556 0.103939227796596 12.8951720117162 0.237850605460913 0.812322700852934 0.943796895210634 -6.55010041516416
None
hsa-mir-532-5p 4.89619453719314 4.47579891442568 5.01512719435569 3.97334437010118 6.42846141609298 4.94546766395515 5.59836914512699
4.90944506316684 5.50608260683541 4.94426530617251 3.95064175892291 4.74331261781248 5.47796799203911 5.10834118382803 NA
3.64944311475091 6.5815652246048 6.09355495414673 NA 3.49540441207201 5.68627824418351 5.39481886626288 4.2754795387903
6.83072440912294 6.89244694175082 4.0675919735848 4.93699802279563 5.94659207066186 5.68321310991894 4.1268175560183 2.79083722013198
4.3564835828023 5.57084227543406 6.93286193582029 6.94441167478853 4.25662780834987 4.02356051140002 3.04960680073561 3.0236284047789
5.25335016127549 -0.0136703623413199 4.99568312063709 -0.0333347464558852 0.973452721141706 0.992203913441046 -6.55097542511282
None
hsa-mir-744 4.52094613285568 5.26187651273752 5.96769908279183 3.56322812681914 7.37403267322628 3.64921137446671 6.80133458640752
5.56831900616583 4.48435403446952 5.75570205388905 5.67421745999525 4.69627227739522 5.42369915994702 5.15551297962325 7.38344347253235
6.83068733770597 6.32643006899182 3.3678188692611 8.17422244530383 4.07657861531886 6.56351958481096 3.66703685259683 5.85288571381987
6.31329356855275 5.63977210647543 3.2087125559707 6.34821625263377 7.20387547566337 5.92400025100812 5.36351225531405 5.27611787069762
5.27914340273689 7.00263486262459 4.07147899126843 2.47735304306356 5.0337874073932 3.93282679936697 4.78059791032292 6.72634578948568
7.54580293390346 0.0922336321097283 5.45666249744032 0.221094340355423 0.825320718830876 0.948410748216205 -6.553896515473
None
hsa-mir-193b 7.17608033831255 6.8724480953227 8.31831006575644 8.19421919428329 5.57600855877509 6.69904297172904 7.85328273324533
7.64553197116842 7.79083132919321 7.55945538414012 5.17874504372091 5.50140665875421 8.03417514044551 5.51181812966265 6.87595006176557
2.97199091514282 8.76918036117074 6.21541111535873 6.98772697361544 7.68347538004663 5.99964413621485 6.82037366014166 9.74020959984622
7.48717769177268 8.13995043207093 6.47253490218911 7.67964290546879 6.25939453272233 5.40582793386063 5.8617609843663 6.102696255755

8.59139370082308 6.31723704404898 6.02145389240617 6.2414493171847 8.94298844225377 8.69619311915651 4.42962811932455 6.98858209469856
6.88044344716268 -0.0831745894929021 6.91234181582692 -0.200038774218311 0.84172215890573 0.957657475132368 -6.55827385952866
None
hsa-mir-26b 8.6177189617578 6.922690439339 8.7606875215116 9.19456148291758 9.36508438964144 5.37704402121574 8.42567023797698
9.8536213920305 8.03470391813049 7.10431171499283 7.91939478788105 9.83136128608816 9.64501736758796 8.02110472467446 10.877606749387
8.10779257530461 6.85838605770305 9.5481453931736 7.86706243074655 7.9841650164558 7.64915151689918 9.14053418820363 8.06154574693511
8.39092579842209 9.2470691065838 8.34414651759432 8.12949364613393 9.62812252919344 7.71989378281186 8.57340400593006 5.81769228742808
7.3412780979367 9.59950254167602 8.51922917432276 10.5796047948542 8.97489797363304 9.66955536713759 5.67484091078734 9.65030103004865
9.19772168348539 -0.0796390115750469 8.45562602921333 -0.195124846616192 0.845560197085134 0.960212427198372 -6.55923247861968
None
hsa-mir-539 2.98107442145369 3.46459387423892 1.79028154777658 4.19781782387243 NA 4.87016687090764 4.41663541489925 2.0942546890667
5.45369090959476 5.62216224001197 2.59549898948934 3.19483118868171 3.70749181650179 5.33719040131394 4.8771223437563 6.51067350621842
5.12933623857449 5.59427897814701 6.17954129382199 2.80240085445268 3.32417311298188 3.92828931676913 5.2132460163625 4.48300256570278
5.87338342026304 3.61439232645113 3.63864249754215 5.21448438083103 3.18553788449873 1.76583541045491 5.27885438260289 3.63069148872715
3.85375962576989 4.90487725758586 4.87153181917451 4.16933758364902 5.33072199981016 3.27005974716896 3.7382084295437 6.61063070420546
-0.0413491352005533 4.27483854802242 -0.0998478772018479 0.920599792425652 0.985980621282861 -6.56037197373628 None
hsa-mir-523 4.24431223161439 3.69526947155388 3.45187869270919 4.66490357292187 3.92851643044086 4.06072124814002 5.16506328739246
4.8378490467656 3.18141943985601 5.02489692380425 4.58448263807265 4.73573717446602 2.50962932040335 4.57734116831969 6.96139846097828
3.80792811534441 2.93246812446961 4.23711347483449 3.97546582246714 3.92271746185799 3.6409600770864 5.099717353714 NA
2.46689545418491 5.66935893957374 3.29655546685911 4.49001236717302 2.72714872519553 2.01682208988319 3.09852560577875 4.44966760287826
5.44371552323131 2.61956041190008 5.95609052610222 4.71924649485823 3.89185927228055 4.31152595886172 5.85536512156047 5.59781487584311
5.35408500490081 -0.0226721247775732 4.23600099944301 -0.0563405995604036 0.955146225854739 0.992203913441046 -6.56372587394881
None
hsa-mir-362-3p 4.79660530059072 7.53965158463056 4.42971176183701 5.39222150533043 5.981568487963 5.49878383799134 6.9558960043072
6.1202381450665 5.05296112335043 NA 6.70795393299189 5.1539369719804 6.65847157471857 4.70875325914873 6.71661027418009
7.75057652289891 7.27848542764343 3.65628001209852 5.66429521991687 7.35883195192709 6.86538620229301 5.66839972731628 4.45187774489732

5.04371136992473 6.68246580825586 4.68160003114124 5.11798298836642 6.38843231290835 6.04595688524086 5.40821616513675 5.46373969427975
4.51877203506318 5.80616310602246 6.90055743944949 5.68512616908583 5.80605418008952 7.31378580494749 7.80609396359937 6.46428067618859
6.86046552512355 0.0206167610899 5.95899745456158 0.0517090191370953 0.958830055676796 0.992203913441046 -6.56397287713994
None
hsa-mir-335 8.59071738343582 8.24858168907616 6.60081028903433 7.13733889101923 7.48036919247016 9.3526023020309 3.72126980677248
4.39442128129417 6.16104382554851 4.13634342828934 8.87989801117171 7.49609417303953 5.26248196149778 5.99052522806699 8.08158465276267
7.66784764368631 4.99305570563871 9.93645208311244 6.90589366308132 9.2637180462268 6.52568704809832 9.86752394297781 7.4081185809586
6.78632394028746 9.5425681209862 7.20323859143136 5.76269023124716 5.37665288691819 6.34051019229199 7.51973093037964 8.19772503569579
8.80853778846561 5.80579578648553 5.31879225616629 7.96118906568502 6.22195705881514 7.31708310299763 7.45847439810493 4.10128692546362
7.91093438422451 -0.0566885505212715 7.0433967381234 -0.128276318826396 0.898102736431641 0.985980621282861 -6.56990602304835
None
hsa-mir-199b-5p 8.57921346120162 8.80004516276311 9.51937793026756 10.2427454603392 6.10874382851269 7.7736319771388 8.91485733354371
8.83563018098821 8.59769438826456 8.95277430401714 9.51605120264638 10.1166785535339 10.1126142459326 10.1327236377373 8.9498898435335
9.70013712668625 7.45339834942579 10.1588740224399 9.53513027126528 10.2200262437118 8.97335046617272 10.2918057813101 9.71378635172965
8.90103980836775 10.3912889752532 8.25950655344204 8.58274752867438 8.08627935808554 10.0209457639781 8.60960024308747 11.6982810763511
9.81230316877662 8.1538152043366 7.617897671723 9.32645428492708 7.86363769728863 7.61140214305575 9.89504313404484 8.35499219638379
9.13718138986516 0.0459439363547887 9.08803990802007 0.116601349278133 0.907332489073575 0.985980621282861 -6.57131763849115
None
hsa-mir-664 5.014002891556 5.70827628906901 6.0871565642468 6.09975637437684 7.15803862826672 3.26833661463318 8.34905725991318
8.10215878127135 3.57782739804155 7.72529301945278 6.99263498245713 3.7158106702468 6.37698171509635 7.82755582324482 4.88235471563159
5.82440043526586 5.65563200751999 5.09334294486789 7.01871385937605 5.34146045822907 6.76850355117565 6.23754751548528 5.16609676709478
7.26007681346019 5.19412109990165 7.41288056157275 6.42276224106899 6.82865355678783 6.28657065347649 4.31741408299488 5.63395806143219
4.9673317238005 6.3522608060059 5.97018122949317 5.58872787229151 7.52624880216499 4.58202108206395 5.20248043718327 4.94531763453552
8.07810477742408 -0.0461233918325288 6.01400126755441 -0.112437674255442 0.910627246452079 0.985980621282861 -6.57178851108649
None
hsa-mir-27b (+++ see message below) 10.3224114887974 11.0434092807432 10.8799530348726 12.5849581385793 12.5647315618537 11.3040566060512

11.5924122567366 9.81898671383974 12.4464613960291 13.012324857298 11.3395450356763 12.7843739236946 12.0431378009182 13.4338569283979 13.2124491157582 11.7171340907602 9.1189900912272 12.2021321505481 10.0378565901325 11.3595818761556 12.8819797467449 11.0331530755327 11.3757032295604 11.1220194704876 11.3703023899646 11.5231856932658 12.3727443968089 10.2394134441971 11.7994167284508 10.7625053597044 12.6982154556316 9.62620486371426 11.3551836469347 11.6687264464425 11.989696968229 12.2456507028704 12.0379229098306 11.5751182774805 11.2013659429103 13.2001053935742 0.0370074397867182 11.6224344270101 0.0944740195374018 0.924859148494061 0.985980621282861 -6.57362379709915 None

hsa-mir-196a (+++ see message below) 2.19349770611696 3.3588727323842 7.03573289868643 4.26161372513886 6.97433952987098 4.84568418763524 4.55904558517061 6.91410944298998 4.37315570436363 6.13948428696344 4.28687366826391 4.30181965761129 7.3647609548302 5.2189388201706 5.29305797556494 5.8717649487238 8.46848569428676 3.65209430673846 8.32202945055604 5.80036263407072 6.19944744897228 2.97944882342933 6.86244315575786 5.97884367164719 3.87694576829237 3.67342842135605 7.03321517538654 6.02738368763469 4.62311059027793 5.70893522439499 4.36075896338599 4.19307748020644 6.44567238506112 5.36676544337478 4.9013692146988 7.27535427640558 5.4760757349256 4.28109803494189 7.15396594550953 7.50905524990819 -0.0345335392715054 5.47905296514261 -0.0804000403418013 0.936026644265345 0.990217660512286 -6.5748390746132 None

hsa-mir-98 8.74036248197971 6.94247813763393 6.83720793659109 6.00635075985902 4.06933051413265 5.76277816368591 5.14037424651675 8.32164978778045 3.64938922735017 9.46818497919599 3.34509652433825 4.17856941703459 2.45875930999296 5.4788250398049 6.56511086707816 6.75166919100712 7.14794781938319 8.46355848721656 4.62061375610362 4.17609416333326 5.3270352280009 6.54331717557578 7.44030783773791 6.72564929897856 5.35159906898253 5.32403082406105 5.54497162039049 5.54385127329751 4.67761336619434 4.71677740736759 7.24569564045731 4.8871483334252 6.6217558041038 4.30745086458071 7.50976748174086 6.50927802709935 5.92622572918963 5.57977320222445 5.80916195845936 5.97345568186673 0.0279742493142132 5.89223041584381 0.0648810724966047 0.948355384207341 0.992203913441046 -6.57595237442729 None

hsa-mir-720 14.6162141540875 11.5505933316697 15.8714895785025 15.2995957191569 16.7894746832575 14.0201059503332 14.3901706833536 13.3376795794869 11.2350900613174 12.7444607523753 13.6163785547961 14.5739457097616 15.5667161852188 15.6939229512856 16.8010153153927 13.8118329281522 15.5913983541519 13.1648818136738 15.7766267175837 13.3497679801846 13.3196448194658 16.3922152965569 14.9089606958301 13.5167470952563 11.202887935972 12.2547076939599 16.5633838700574 16.6207924263028 12.7828260725925 14.4125214507516 14.9296640626417 13.6254775358927 14.9486607239549 15.1384615802409 13.9546110976246 14.3587213010961 14.8330366072568 13.9819905588976 15.7107256263439 13.8112776923712 0.0267023430337918 14.3767168786702 0.0625466859154097 0.950211046620709 0.992203913441046 -6.57609926054246

None
hsa-mir-374b 7.00957144064592 8.87432852317553 6.84416861336154 4.33132530880766 7.54274470930546 5.92605804713385 6.82402800324235
7.4940783916056 8.77491362759558 4.88939370089201 5.89671610607011 6.12392276910933 6.94578129365822 6.33352767320448 7.84526170759791
6.22478364175991 6.22959120094761 8.44780437272193 5.34010921585209 5.13562347220772 7.29094432564451 6.42325063332036 6.6811696020057
7.05190887988329 6.67548578350807 7.36718326587395 5.40566728149318 6.99457174826913 7.14275314348294 7.56674092596809 5.15422404331348
6.08753079648851 6.62514242041216 8.47814340225124 6.84787570337344 8.60260842297097 6.14566358470137 5.26884164562921 6.52284815288457
5.16964066479696 -0.023423130368819 6.66339815612915 -0.0588874729156318 0.953120391803786 0.992203913441046 -6.57631867921343
None
hsa-mir-544 (+++ see message below) 4.91691859752061 4.89119151056652 6.86715779206954 6.43552112509691 7.10562971102044 3.6247841145933
7.08723420428659 8.15927515839313 5.13266856232636 5.7656632259106 6.25009356862611 7.09879860935013 6.53513067387771 7.76852584767901
8.06115090999622 4.25333494438255 6.90974136018539 4.534550554429 9.00995931101662 5.93046050320399 2.92286866358778 4.47922244053777
6.51348533512363 7.82636773457985 5.0471899088179 6.77254667545603 7.81090896551985 10.1579798391679 5.69428321354663 6.20189605331591
5.58909080642839 6.26920600712727 7.68948709142873 6.335261060223 6.2968166341151 5.88323426165146 4.6790489595579 3.81832696081142
7.48205399680308 9.27814913455097 -0.0204816728909929 6.32713035067203 -0.0468885715169039 0.962664626211535 0.992203913441046
-6.57694540326905 None
hsa-mir-19b 11.8139186340647 11.3697852369045 8.66984879124337 10.4652577365085 7.56602324177014 7.14701851311438 11.3102374923967
11.5214384488043 13.4118565353522 10.5237977706798 10.1626136650969 8.47371390429625 10.4083909004326 10.3128539143123 8.44981595075468
11.9940526716983 8.43719802057065 9.7364903030479 14.7816582694489 8.72843572075903 11.4533203798167 9.90671113898146 10.0280983399272
9.55593875444412 11.7915475783244 8.68554787527772 11.6504408755277 10.9261524827815 10.2458347150004 10.738387048847 9.054088087007
13.7853705348132 9.81837615869355 9.45750353557393 8.87621094955564 9.08130326465408 9.95364759723044 9.99949654516451 9.71486248849268
10.9098714933899 -0.0174152061123483 10.272927889119 -0.0396437928220113 0.968430015520896 0.992203913441046 -6.57725496984135
None
hsa-mir-30b 8.81591083914421 10.8174485532344 11.0645221322452 10.4785682657045 10.0560985350302 11.0714755470384 10.4285458923937
10.4164720082328 10.262093699309 7.95407753027913 12.6494062529965 8.41629524674626 10.8868545381811 10.8450269769147 10.3660233846467
9.27351119866992 10.1588980017248 11.2101624814997 11.5367366465128 11.8921328060585 9.60939814994521 11.9328775853115 9.88512282876272
12.3005919869511 7.26577977289576 10.3300914294157 9.41558399480377 10.4501681049619 11.6965556013759 10.3120473204134 10.4251980911411

11.1357027011026 11.6988216922231 11.419509864419 9.63061474006103 9.16786138007791 9.4986525729936 8.82714993108041 11.3048938685528
12.1022557517992 0.00956915841374162 10.4252284476213 0.0236554792008613 0.981158969419185 0.992203913441046 -6.57775472140863
None
hsa-mir-342-3p 7.64726358375179 8.00293700256109 7.18475946476444 9.63260872847203 8.99809057617628 6.51337908994364 9.31435899893084
7.34579583301142 7.90386418050848 7.14823894089341 7.9166213321155 10.176162892202 8.9404139671658 8.36272447525237 6.54465193021438
8.09137618107911 8.79306672318535 5.79518649584389 7.9134889722455 8.20290133473166 6.76213487762707 8.27318889993632 6.22272819399431
8.42797380679999 7.26653755875037 7.76432621459632 8.10371735615838 8.07067985841315 8.06017706547253 6.50341987648202 7.58817976193518
8.41231436634449 7.85556842424019 10.0443859483926 8.90415908159376 10.005306888738 8.03059426853186 9.02568947155983 7.27336785908527
7.65231150788281 0.00905647082572614 8.01686629973959 0.0230821104484427 0.981615561437343 0.992203913441046 -6.57776795429736
None
hsa-mir-1274b 7.58894795119939 5.98697768131191 11.0390649858171 7.68721041200995 10.426064319437 7.36933901359648 8.91161055563054
9.16572990014137 8.54656832331412 6.03269116560736 7.31475619410181 9.89492196317709 10.6200975598374 8.86081756967388 8.99651558348253
6.77259708739607 11.9390748125766 7.72039319192735 11.3462698799345 6.49787522785042 7.09455965179978 10.3294547725033 6.34839683965091
9.80393702839151 6.77062452924363 6.71711525871821 10.7017159779314 10.1364281361766 10.2874378690062 8.22523748383725 6.45801012057562
7.10838521031285 8.1049167171686 9.39583636408996 9.46728239416428 10.1977025666555 5.6478618963857 7.63254827080547 12.0502594882351
10.3159994962868 -0.00380933469579148 8.63778083624904 -0.00831802470249503 0.993374344641959 0.995862509864406 -6.57799687924873
None
hsa-mir-106b 7.18238309135239 5.81237305575469 6.95993854960516 7.4842178584521 8.93126619946716 9.60054041810252 3.91594540073484
6.35334741646226 6.93814525028997 7.43970377516927 6.94369603320177 6.57331523559205 7.73843277806079 6.36628745068788 8.78662585276797
7.289360683117 6.6678655929548 6.82621081834944 4.84532670586736 5.61519368549379 6.36123896913759 6.72124632821317 8.15882248511043
6.67790012991186 7.97157776060445 7.05129042021607 7.9075337441253 6.73042603889942 7.98698293948983 7.35618246662477 6.64736169466624
7.54725805762691 5.73547747653363 6.11767897461011 6.13581013919775 8.50124767113628 7.19117274024243 6.6057143030302 7.00236253387499
3.81808840717324 0.00224012855292877 6.9123887282977 0.00556321192256397 0.995568640068563 0.995862509864406 -6.57801576246835
None
hsa-mir-129-5p 2.31016604647687 5.40303870391304 NA NA NA NA 4.66923726214861 NA NA NA 3.11350043351991 2.85756884262758 NA NA
NA 1.87653104496493 NA 4.63932529693312 NA NA NA NA NA NA NA NA NA NA NA NA NA NA NA NA NA NA NA NA NA

NA 3.55276680436915 NA NA NA NA None
hsa-mir-607 NA NA NA NA NA NA NA NA NA NA NA NA NA NA NA NA NA NA NA NA NA NA NA NA NA NA NA NA NA NA
4.23935146988558 NA NA NA NA NA NA NA NA NA NA 4.23935146988558 NA NA NA NA None
hsa-mir-202 NA NA NA 2.58606934574463 NA NA 3.32387800953309 3.22619192484387 NA NA NA NA NA NA NA 3.62339939660631 NA NA
NA NA NA NA NA NA NA NA NA NA NA NA NA NA NA NA NA NA NA NA NA NA NA 3.18988466918197 NA NA NA NA None
hsa-mir-330-5p NA NA NA NA NA NA NA 1.6079335505555 NA NA NA NA NA NA NA NA NA NA NA NA NA NA NA NA NA NA
NA NA NA NA NA NA NA NA NA NA NA NA NA NA NA 1.6079335505555 NA NA NA NA None
hsa-mir-935 0.292872881885787 NA NA NA NA NA NA NA NA NA NA NA NA NA NA 3.14920006503259 NA NA NA NA NA NA NA
NA NA NA NA NA NA NA NA NA NA NA NA NA NA NA NA NA NA 1.72103647345919 NA NA NA NA None
hsa-mir-548b-5p NA NA NA NA NA NA NA NA NA NA NA NA NA NA NA NA NA NA NA NA NA 1.26693079168994 NA NA NA NA
NA NA NA NA NA NA NA NA NA NA NA NA 3.5396564637654 NA NA 2.40329362772767 NA NA NA NA None
hsa-mir-298 1.84773304098004 NA NA NA NA NA NA NA NA NA NA NA NA NA NA 2.16220377375579 NA NA NA NA NA NA NA NA
NA NA NA NA NA NA NA NA NA NA NA NA NA NA NA NA NA 2.00496840736792 NA NA NA NA None
hsa-mir-921 NA NA NA NA NA NA NA NA NA NA NA NA NA NA NA NA NA NA NA NA NA NA NA NA NA NA NA NA NA NA
NA NA NA NA NA NA NA 3.54324638731751 NA NA NA 3.54324638731751 NA NA NA NA None
hsa-mir-761 NA 3.30584486504583 NA NA NA NA NA 2.41136725217282 NA NA NA NA NA NA NA NA NA NA NA NA NA NA NA NA
NA NA NA NA NA NA NA NA NA NA NA NA NA NA NA NA NA 2.85860605860933 NA NA NA NA None
hsa-mir-659 NA NA NA NA NA NA NA NA NA NA NA NA NA NA NA NA NA NA NA NA NA 1.60943818632429 NA NA NA NA NA
4.20780486521512 NA NA NA NA NA NA NA NA NA 4.20962508930531 NA NA NA 3.34228938028157 NA NA NA NA None
hsa-mir-933 NA NA NA NA NA NA NA NA NA NA NA NA NA NA NA NA NA NA NA NA NA NA NA NA NA NA NA
1.86721410057276 NA NA NA NA NA NA NA NA NA 3.30043321701945 2.54644543724822 NA NA 2.57136425161348 NA NA NA NA None
hsa-mir-675 NA NA NA 3.25121366484156 NA NA NA NA NA NA NA NA NA NA NA NA NA NA NA NA NA NA NA NA NA NA NA
NA NA NA NA NA NA NA NA NA NA NA NA NA NA 3.25121366484156 NA NA NA NA None
hsa-mir-371-3p NA NA NA NA NA NA NA NA NA NA NA 1.10359241147336 NA NA NA NA NA NA NA NA NA NA NA NA NA NA
NA NA NA NA NA NA NA NA NA NA NA NA NA NA NA 1.10359241147336 NA NA NA NA None
hsa-mir-548e NA NA NA NA NA 3.22583925978552 NA NA NA 3.88584254013528 NA 2.79509597496816 NA NA NA NA 3.51741722731788 NA

NA NA NA NA NA NA NA NA NA NA NA NA NA NA NA NA NA NA NA NA NA NA NA 3.35604875055171 NA NA NA NA None
hsa-mir-1289 0.103415899348479 NA NA NA NA 3.4683702600219 NA NA 4.17123893259031 NA NA NA NA NA NA NA NA
3.31525348297467 NA NA NA NA NA NA NA NA NA NA NA NA NA NA NA NA NA NA NA NA NA NA NA 2.76456964373384 NA
NA NA NA None
hsa-mir-587 4.25204631011376 NA NA 4.73463153782356 NA NA NA NA NA NA NA NA NA NA NA NA NA NA NA NA NA NA NA NA
NA NA NA NA NA NA NA NA NA NA NA NA NA NA NA NA NA 4.49333892396866 NA NA NA NA None
hsa-mir-1281 0.911099826768108 NA NA NA NA NA NA NA NA NA NA NA NA NA NA NA NA NA NA NA NA NA NA NA NA NA
NA NA NA NA NA NA NA NA NA NA NA NA NA NA NA 0.911099826768108 NA NA NA NA None
hsa-mir-1256 NA NA NA NA NA NA NA NA NA NA NA NA NA NA NA NA NA NA NA NA NA NA NA NA NA NA NA NA NA
1.7572582105442 NA NA NA NA NA NA NA 1.17487337412206 NA NA NA 1.46606579233313 NA NA NA NA None
hsa-mir-1469 NA 2.63840137914534 NA NA NA NA NA NA NA NA NA NA NA NA NA NA NA 4.63959580694362 NA NA NA NA NA NA
NA NA NA NA NA NA NA NA NA NA NA NA NA NA NA NA NA 3.63899859304448 NA NA NA NA None
hsa-mir-422a NA NA NA NA NA NA NA NA NA NA NA NA NA NA NA NA NA NA NA NA NA NA NA NA NA NA NA NA NA NA
NA NA NA NA NA NA NA NA NA NA NA NA NA NA NA NA None
hsa-mir-644 NA NA NA NA NA NA NA NA NA NA NA NA NA NA NA NA NA NA NA NA NA 1.55806513728565 NA NA NA NA NA
3.29286840674985 NA NA NA NA NA 4.49582531184396 NA NA NA NA NA NA NA 3.11558628529315 NA NA NA NA None
hsa-mir-184 NA NA NA NA NA NA NA NA NA NA NA NA NA NA NA NA NA NA NA NA NA NA NA NA NA NA NA NA NA
4.06848919399942 NA NA 4.99404704803568 NA NA NA NA 3.15962111553235 NA NA NA 4.07405245252248 NA NA NA NA None
hsa-mir-488 NA NA NA NA NA NA NA NA NA NA NA NA NA NA NA NA NA NA NA NA NA NA NA NA NA NA NA NA NA NA
NA NA NA NA NA NA NA 0.25949801599387 NA NA NA 0.25949801599387 NA NA NA NA None
hsa-mir-582-3p NA NA NA NA NA NA NA NA NA NA NA NA NA NA NA NA NA NA NA NA NA 3.17329486033432 NA NA NA NA
NA NA NA NA NA NA NA NA NA NA NA 2.07519468083047 NA NA NA 2.6242447705824 NA NA NA NA None
hsa-mir-1296 NA NA NA NA NA NA NA NA NA 3.01879966080738 NA NA NA NA NA NA NA NA NA NA NA NA NA NA NA NA NA NA
NA NA NA NA NA NA NA NA NA NA NA NA NA NA 3.01879966080738 NA NA NA NA None
hsa-mir-483-5p NA NA NA NA NA NA NA NA NA 3.37140854300816 NA NA NA NA NA 1.29612360386349 NA NA NA NA NA NA NA
NA NA NA NA NA NA NA NA NA NA NA NA NA NA NA NA NA NA 2.33376607343583 NA NA NA NA None

hsa-mir-583 NA NA NA NA NA NA NA NA NA NA NA NA NA NA NA NA NA NA NA NA NA NA NA NA NA NA NA NA NA 1.87583356501549 NA NA NA NA NA NA NA 1.56671773091807 NA NA NA 1.72127564796678 NA NA NA NA None

hsa-mir-1909 NA NA NA NA NA NA NA NA NA NA NA NA NA NA NA 1.42098130665886 NA NA NA NA NA NA NA NA NA NA NA NA NA NA NA NA NA NA NA NA NA NA NA NA NA 1.42098130665886 NA NA NA NA None

hsa-mir-567 NA NA NA NA NA NA NA NA NA NA NA NA NA NA NA NA NA NA NA NA NA 3.01647063454434 NA NA NA NA NA NA NA 3.37466162430519 NA NA NA NA NA NA NA NA NA NA NA 3.19556612942477 NA NA NA NA None

hsa-mir-662 NA NA NA NA NA NA NA NA NA NA NA NA NA NA NA NA NA NA NA NA NA NA NA NA NA NA NA NA NA NA NA NA NA NA NA NA NA NA NA NA NA NA NA NA NA NA None

hsa-mir-671-5p NA NA NA NA NA NA NA NA NA NA NA NA NA NA NA NA NA NA NA NA NA NA NA NA NA NA NA NA NA 1.66814786542637 NA NA NA NA NA NA NA NA NA NA NA 1.66814786542637 NA NA NA NA None

hsa-mir-297 NA NA NA NA NA NA NA NA NA NA NA NA NA NA NA NA NA NA NA NA NA NA NA NA NA NA NA NA NA NA NA NA NA NA NA NA NA NA NA NA NA NA NA NA NA NA None

hsa-mir-648 0.31856216217572 NA NA NA NA NA NA NA NA NA NA NA NA NA NA NA NA NA NA NA NA NA NA NA NA NA NA NA NA NA NA NA NA NA NA NA NA NA NA NA NA 0.31856216217572 NA NA NA NA None

hsa-mir-198 NA NA NA NA NA NA NA NA NA NA NA NA NA NA NA NA NA NA NA NA NA NA NA NA NA NA NA NA NA NA NA NA NA NA NA NA NA NA NA NA NA NA NA NA NA NA None

hsa-mir-371-5p NA NA NA NA NA NA NA NA NA NA NA NA NA NA NA NA NA 2.7019404968019 NA NA NA NA NA NA NA NA NA NA NA NA NA NA NA NA NA NA NA NA NA NA NA NA NA 2.7019404968019 NA NA NA NA None

hsa-mir-2110 NA NA NA NA NA NA NA NA NA NA NA NA NA NA NA 1.79900213844959 NA NA NA NA NA NA NA NA NA NA NA NA NA NA NA NA NA NA NA NA NA NA NA NA NA 1.79900213844959 NA NA NA NA None

hsa-mir-936 NA NA NA NA NA NA NA NA NA NA NA NA NA NA NA NA NA NA NA NA NA NA NA NA NA NA NA NA NA NA NA NA NA NA NA NA NA NA NA NA NA NA NA NA NA NA None

hsa-mir-624 NA NA NA NA NA NA NA NA NA NA NA NA NA NA NA NA NA NA NA NA NA NA NA NA NA NA NA NA NA NA NA NA NA NA NA NA NA 0.117536070015021 NA NA NA 0.117536070015021 NA NA NA NA None

hsa-mir-885-3p 3.1341154496203 NA NA NA NA NA NA NA NA NA NA NA NA NA NA NA NA NA NA NA NA NA NA NA NA NA NA NA NA NA NA NA NA NA NA NA NA NA NA NA NA 3.1341154496203 NA NA NA NA None

hsa-mir-1278 NA NA NA NA NA NA NA NA NA NA NA NA NA NA NA NA NA NA NA NA NA NA NA NA NA NA NA NA NA NA
NA NA NA NA NA NA NA 2.45913674867245 NA NA NA 2.45913674867245 NA NA NA NA None
hsa-mir-449c NA NA NA NA NA NA NA NA NA NA NA NA NA NA NA NA NA NA NA NA NA NA NA NA NA NA NA
3.65501513901459 NA 1.48976159319236 NA NA NA NA NA NA NA NA NA NA NA 2.57238836610348 NA NA NA NA None
hsa-mir-1280 NA NA NA NA NA NA 2.45724083240191 NA NA NA NA NA NA NA NA 2.78814182622618 NA NA NA NA NA NA NA NA
NA NA NA NA NA NA NA NA NA NA NA NA NA NA NA NA NA 2.62269132931405 NA NA NA NA None
hsa-mir-606 NA NA NA NA NA NA NA NA NA NA NA NA NA NA NA NA NA NA NA NA NA NA NA NA NA NA NA NA NA NA
NA NA NA NA NA NA NA NA NA NA NA NA NA NA NA NA None
hsa-mir-509-3-5p NA NA NA NA NA NA NA NA NA NA NA NA NA NA NA NA NA NA NA NA NA NA NA NA NA NA NA NA NA
3.10810771970143 NA NA NA NA NA NA NA 1.66427530593961 NA NA NA 2.38619151282052 NA NA NA NA None
hsa-mir-521 NA NA NA NA NA NA NA NA NA NA NA NA NA NA NA NA NA NA NA NA NA NA NA NA NA NA NA NA NA NA
NA NA NA NA NA NA NA NA NA NA NA NA NA NA NA NA None
hsa-mir-548k NA NA NA NA NA NA NA NA NA NA NA NA NA NA NA NA NA NA NA NA NA 4.28888044079882 NA NA NA NA NA
NA NA NA NA NA NA NA NA NA NA NA NA NA NA 4.28888044079882 NA NA NA NA None
