# Supplementary material for: MicroRNA-196b inhibits late apoptosis of pancreatic cancer cells by targeting CADM1
Source: Sci Rep. 2017 Sep 13;7:11467. doi: 10.1038/s41598-017-11248-3 (PMC5597590; doi:10.1038/s41598-017-11248-3)
Supplement: Supplementary file 3 — Table S2 [file 41598_2017_11248_MOESM3_ESM.pdf]

MicroRNA-196b inhibits late apoptosis of pancreatic cancer cells by targeting CADM1

Hong-Ling Wang[1,2], Rui Zhou[1,2], Jing Liu[1,2], Ying Chang[1,2], Shi Liu[1,2], Xiao-Bing Wang[1,2], Mei-Fang Huang[1,2] & Qiu Zhao[1,2*]

[1]Department of Gastroenterology, Zhongnan Hospital of Wuhan University, Wuhan 430071, P.R. China

[2]The Hubei Clinical Center and Key Laboratory of Intestinal and Colorectal Diseases, Wuhan 430071, P.R. China

*Corresponding Author: Qiu Zhao, Department of Gastroenterology, Zhongnan Hospital of Wuhan University. 169 East Lake Road, Wuhan 430071, China. Tel: +86-27-67812888; Fax: +86-27-67812892; E-mail: qiuzhaoznyy@163.com

Table S2. Enrichment analysis result.

type	iterm	nsel	nbg	nhitsel	nset	fold	pvalue	fdr	hit	set
Family	let-7 family	79	585	6	9	4.93670886075949	0.000208687636952209	0.00328456193811738	hsa-let-7b;hsa-let-7d;hsa-let-7e;hsa-let-7i;hsa-let-7c;hsa-let-7g	hsa-let-7a;hsa-let-7b;hsa-let-7c;hsa-let-7d;hsa-let-7e;hsa-let-7f;hsa-mir-98;hsa-let-7g;hsa-let-7i
HMDD	Adenocarcinoma	79	585	11	45	2.2626582278481	0.00276760120987559	0.0217798182168471	hsa-mir-200c;hsa-mir-200a;hsa-mir-31;hsa-mir-126;hsa-mir-429;hsa-mir-103;hsa-mir-95;hsa-mir-195;hsa-mir-191;hsa-mir-143;hsa-mir-21	hsa-let-7a;hsa-mir-103;hsa-mir-106a;hsa-mir-10b;hsa-mir-126;hsa-mir-133a;hsa-mir-135a;hsa-mir-143;hsa-mir-145;hsa-mir-151;hsa-mir-152;hsa-mir-155;hsa-mir-16;hsa-mir-17;hsa-mir-181b;hsa-mir-182;hsa-mir-183;hsa-mir-191;hsa-mir-192;hsa-mir-193b;hsa-mir-194;hsa-mir-195;hsa-mir-196a;hsa-mir-200a;hsa-mir-200b;hsa-mir-200c;hsa-mir-203;hsa-mir-204;hsa-mir-205;hsa-mir-20a;hsa-mir-21;hsa-mir-210;hsa-mir-215;hsa-mir-221;hsa-mir-223;hsa-mir-25;hsa-mir-26b;hsa-mir-31;hsa-mir-429;hsa-mir-449a;hsa-mir-449b;hsa-mir-92a;hsa-mir-93;hsa-mir-95;hsa-mir-99b
HMDD	Adrenocortical Carcinoma	79	585	12	47	2.11573236889693	0.00331463780778675	0.0244877323758939	hsa-mir-200c;hsa-mir-1;hsa-let-7b;hsa-mir-126;hsa-mir-376c;hsa-let-7d;hsa-mir-7;hsa-mir-424;hsa-let-7c;hsa-mir-132;hsa-mir-375;hsa-mir-143	hsa-let-7a;hsa-let-7b;hsa-let-7c;hsa-let-7d;hsa-mir-1;hsa-mir-100;hsa-mir-106b;hsa-mir-125b;hsa-mir-126;hsa-mir-130a;hsa-mir-132;hsa-mir-133a;hsa-mir-135a;hsa-mir-137;hsa-mir-139;hsa-mir-143;hsa-mir-145;hsa-mir-146a;hsa-mir-148a;hsa-mir-155;hsa-mir-15b;hsa-mir-16;hsa-mir-17;hsa-mir-192;hsa-mir-194;hsa-mir-196a;hsa-mir-200b;hsa-mir-200c;hsa-mir-203;hsa-mir-210;hsa-mir-222;hsa-mir-23b;hsa-mir-28;hsa-mir-301a;hsa-mir-301b;hsa-mir-30a;hsa-mir-335;hsa-mir-375;hsa-mir-376a;hsa-mir-376b;hsa-mir-376c;hsa-mir-424;hsa-mir-449a;hsa-mir-449b;hsa-mir-484;hsa-mir-491;hsa-mir-7
HMDD	Asthma	79	585	7	12	4.31962025316456	0.000186126203228419	0.00306262207130399	hsa-let-7b;hsa-mir-126;hsa-let-7d;hsa-let-7e;hsa-let-7i;hsa-let-7c;hsa-let-7g	hsa-let-7a;hsa-let-7b;hsa-let-7c;hsa-let-7d;hsa-let-7e;hsa-let-7f;hsa-let-7g;hsa-let-7i;hsa-mir-126;hsa-mir-148a;hsa-mir-148b;hsa-mir-152
HMDD	Breast Neoplasms	79	585	25	109	1.86996547756041	0.000176342834685261	0.00303981457886022	hsa-mir-200c;hsa-mir-1;hsa-mir-200a;hsa-mir-31;hsa-mir-429;hsa-mir-29a;hsa-mir-103;hsa-mir-135b;hsa-mir-107;hsa-let-7d;hsa-mir-497;hsa-mir-7;hsa-mir-214;hsa-mir-18a;hsa-mir-132;hsa-mir-30d;hsa-mir-195;hsa-mir-197;hsa-mir-34a;hsa-mir-124;hsa-mir-143;hsa-mir-133b;hsa-mir-493;hsa-mir-34b;hsa-mir-21	hsa-let-7a;hsa-let-7d;hsa-let-7f;hsa-mir-1;hsa-mir-103;hsa-mir-106b;hsa-mir-107;hsa-mir-10a;hsa-mir-10b;hsa-mir-124;hsa-mir-125a;hsa-mir-125b;hsa-mir-127;hsa-mir-132;hsa-mir-133a;hsa-mir-133b;hsa-mir-135b;hsa-mir-141;hsa-mir-143;hsa-mir-145;hsa-mir-146a;hsa-mir-146b;hsa-mir-149;hsa-mir-151;hsa-mir-153;hsa-mir-155;hsa-mir-15a;hsa-mir-17;hsa-mir-181b;hsa-mir-182;hsa-mir-183;hsa-mir-18a;hsa-mir-18b;hsa-mir-193b;hsa-mir-194;hsa-mir-195;hsa-mir-196a;hsa-mir-197;hsa-mir-199a;hsa-mir-199b;hsa-mir-19a;hsa-mir-19b;hsa-mir-200a;hsa-mir-200b;hsa-mir-200c;hsa-mir-204;hsa-mir-205;hsa-mir-206;hsa-mir-20a;hsa-mir-20b;hsa-m

ir-21;hsa-mir-210;hsa-mir-214;hsa-mir-215;hsa-mir-218;hsa-mir-219;hsa-mir-221;hsa-mir-222;hsa-mir-223;hsa-mir-25;hsa-mir-27a;hsa-mir-296;hsa-mir-29a;hsa-mir-29b;hsa-mir-302a;hsa-mir-302b;hsa-mir-302c;hsa-mir-302d;hsa-mir-30a;hsa-mir-30b;hsa-mir-30c;hsa-mir-30d;hsa-mir-31;hsa-mir-320;hsa-mir-328;hsa-mir-335;hsa-mir-338;hsa-mir-339;hsa-mir-340;hsa-mir-342;hsa-mir-345;hsa-mir-34a;hsa-mir-34b;hsa-mir-34c;hsa-mir-367;hsa-mir-383;hsa-mir-429;hsa-mir-451;hsa-mir-453;hsa-mir-486;hsa-mir-488;hsa-mir-493;hsa-mir-497;hsa-mir-499;hsa-mir-510;hsa-mir-516a;hsa-mir-516b;hsa-mir-519c;hsa-mir-519d;hsa-mir-519e;hsa-mir-520a;hsa-mir-520h;hsa-mir-629;hsa-mir-661;hsa-mir-7;hsa-mir-9;hsa-mir-92a;hsa-mir-93;hsa-mir-96

HMDD Carcinoma 79 585 11 42 2.1435709526982 0.00446861169336391 0.0299562487592173

hsa-mir-200c;hsa-mir-200a;hsa-mir-31;hsa-mir-23a;hsa-let-7i;hsa-mir-214;hsa-mir-138;hsa-mir-375;hsa-mir-34a;hsa-mir-372;hsa-mir-21

hsa-let-7a;hsa-let-7i;hsa-mir-106b;hsa-mir-10a;hsa-mir-10b;hsa-mir-125b;hsa-mir-138;hsa-mir-142;hsa-mir-145;hsa-mir-146b;hsa-mir-149;hsa-mir-155;hsa-mir-16;hsa-mir-182;hsa-mir-183;hsa-mir-184;hsa-mir-192;hsa-mir-200a;hsa-mir-200b;hsa-mir-200c;hsa-mir-205;hsa-mir-20a;hsa-mir-21;hsa-mir-214;hsa-mir-221;hsa-mir-222;hsa-mir-23a;hsa-mir-302a;hsa-mir-302b;hsa-mir-302c;hsa-mir-302d;hsa-mir-302e;hsa-mir-302f;hsa-mir-30a;hsa-mir-31;hsa-mir-34a;hsa-mir-371;hsa-mir-372;hsa-mir-373;hsa-mir-375;hsa-mir-423;hsa-mir-96

HMDD Carcinoma, Hepatocellular 79 585 26 77 2.79031370390754 7.9576305709687e-09 5.76132453338134e-07

hsa-mir-200c;hsa-mir-122;hsa-mir-1;hsa-mir-200a;hsa-let-7b;hsa-mir-126;hsa-mir-24;hsa-mir-103;hsa-mir-107;hsa-let-7d;hsa-mir-497;hsa-mir-23a;hsa-let-7e;hsa-let-7i;hsa-mir-224;hsa-mir-99a;hsa-let-7c;hsa-mir-18a;hsa-mir-30d;hsa-mir-195;hsa-mir-191;hsa-mir-34a;hsa-let-7g;hsa-mir-124;hsa-mir-181a;hsa-mir-21

hsa-let-7a;hsa-let-7b;hsa-let-7c;hsa-let-7d;hsa-let-7e;hsa-let-7f;hsa-let-7g;hsa-let-7i;hsa-mir-1;hsa-mir-100;hsa-mir-101;hsa-mir-103;hsa-mir-106a;hsa-mir-107;hsa-mir-10a;hsa-mir-1202;hsa-mir-122;hsa-mir-124;hsa-mir-125a;hsa-mir-125b;hsa-mir-126;hsa-mir-127;hsa-mir-129;hsa-mir-140;hsa-mir-141;hsa-mir-142;hsa-mir-145;hsa-mir-146a;hsa-mir-146b;hsa-mir-148a;hsa-mir-150;hsa-mir-151;hsa-mir-152;hsa-mir-155;hsa-mir-16;hsa-mir-17;hsa-mir-181a;hsa-mir-18a;hsa-mir-18b;hsa-mir-191;hsa-mir-195;hsa-mir-196a;hsa-mir-198;hsa-mir-199a;hsa-mir-199b;hsa-mir-19a;hsa-mir-19b;hsa-mir-200a;hsa-mir-200b;hsa-mir-200c;hsa-mir-203;hsa-mir-20a;hsa-mir-20b;hsa-mir-21;hsa-mir-22;hsa-mir-221;hsa-mir-222;hsa-mir-223;hsa-mir-224;hsa-mir-23a;hsa-mir-24;hsa-mir-27a;hsa-mir-30d;hsa-mir-34a;hsa-mir-362;hsa-mir-378;hsa-mir-425;hsa-mir-497;hsa-mir-500;hsa-mir-512;hsa-mir-602;hsa-mir-92a;hsa-mir-92b;hsa-mir-93;hsa-mir-96;hsa-mir-99a;hsa-mir-99b

HMDD Carcinoma, Non-Small-Cell Lung 79 585 6 16 3.17359855334539 0.00440128371815057 0.0299562487592173

hsa-mir-1;hsa-mir-30d;hsa-mir-34a;hsa-mir-181a;hsa-mir-143;hsa-mir-21

hsa-mir-1;hsa-mir-125a;hsa-mir-143;hsa-mir-155;hsa-mir-16-1;hsa-mir-16-2;hsa-mir-181a;hsa-mir-199a;hsa-mir-199b;hsa-mir-205;hsa-mir-21;hsa-mir-212;hsa-mir-30d;hsa-mir-34a;hsa-mir-486;hsa-mir-499

HMDD Carcinoma, Oral 79 585 6 15 3.70253164556962 0.00167187969510873 0.0155184730674195

hsa-mir-135b;hsa-mir-224;hsa-mir-197;hsa-mir-34a;hsa-mir-181a;hsa-mir-21

hsa-mir-135b;hsa-mir-146b;hsa-mir-181a;hsa-mir-181b;hsa-mir-181c;hsa-mir-181d;hsa-mir-197;hsa-mir-21;hsa-mir-221;hsa-mir-222;hsa-mir-224;hsa-mir-338;hsa-mir-34a;hsa-mir-378;hsa-mir-520h

HMDD Carcinoma, Renal Cell 79 585 12 26 3.70253164556962 5.56989297199562e-06 0.000155100096604801

hsa-mir-200c;hsa-let-7b;hsa-mir-429;hsa-let-7d;hsa-let-7e;hsa-mir-26a;hsa-let-7i;hsa-mir-224;hsa-let-7c;hsa-mir-34a;hsa-let-7g;hsa-mir-34b

hsa-let-7a;hsa-let-7b;hsa-let-7c;hsa-let-7d;hsa-let-7e;hsa-let-7f;hsa-let-7g;hsa-let-7h;hsa-let-7i;hsa-mir-106b;hsa-mir-141;hsa-mir-155;hsa-mir-16;hsa-mir-200b;hsa-mir-200c;hsa-mir-210;hsa-mir-224;hsa-mir-26a;hsa-mir-30c;hsa-mir-34a;hsa-mir-34b;hsa-mir-34c;hsa-mir-363;hsa-mir-429;hsa-mir-452;hsa-mir-514

HMDD Cardiomyopathy, Hypertrophic 79 585 6 14 4.44303797468354 0.000469342957609485 0.00514855001983738

hsa-mir-1;hsa-mir-24;hsa-mir-23a;hsa-mir-214;hsa-mir-195;hsa-mir-21

hsa-mir-1;hsa-mir-125b;hsa-mir-133a;hsa-mir-150;hsa-mir-181b;hsa-mir-195;hsa-mir-199a;hsa-mir-208;hsa-mir-21;hsa-mir-214;hsa-mir-23a;hsa-mir-23b;hsa-mir-24;hsa-mir-27b

HMDD Cocaine-Related Disorders 79 585 3 4 5.55379746835443 0.00709638542744933 0.0395909718951495

hsa-let-7d;hsa-mir-124;hsa-mir-181a hsa-let-7d;hsa-mir-124;hsa-mir-181a;hsa-mir-212

HMDD Colonic Neoplasms 79 585 18 55 2.7202273314389 4.84229527217088e-06 0.000146075907377155

hsa-let-7b;hsa-mir-126;hsa-mir-24;hsa-mir-107;hsa-let-7d;hsa-let-7e;hsa-let-7i;hsa-let-7c;hsa-mir-18a;hsa-mir-132;hsa-mir-191;hsa-mir-34a;hsa-let-7g;hsa-mir-32;hsa-mir-143;hsa-mir-133b;hsa-mir-21;hsa-mir-630

hsa-let-7a;hsa-let-7b;hsa-let-7c;hsa-let-7d;hsa-let-7e;hsa-let-7f;hsa-let-7g;hsa-let-7i;hsa-mir-101;hsa-mir-106a;hsa-mir-106b;hsa-mir-107;hsa-mir-126;hsa-mir-127;hsa-mir-128b;hsa-mir-132;hsa-mir-133b;hsa-mir-137;hsa-mir-140;hsa-mir-141;hsa-mir-142;hsa-mir-143;hsa-mir-145;hsa-mir-146a;hsa-mir-152;hsa-mir-155;hsa-mir-17;hsa-mir-18a;hsa-mir-191;hsa-mir-192;hsa-mir-19a;hsa-mir-19b;hsa-mir-200b;hsa-mir-205;hsa-mir-20a;hsa-mir-21;hsa-mir-221;hsa-mir-223;hsa-mir-24;hsa-mir-29b;hsa-mir-30c;hsa-mir-32;hsa-mir-34a;hsa-mir-365;hsa-mir-449a;hsa-mir-486;hsa-mir-498;hsa-mir-518c;hsa-mir-552;hsa-mir-584;hsa-mir-615;hsa-mir-622;hsa-mir-629;hsa-mir-630;hsa-mir-92a

HMDD Colorectal Neoplasms 79 585 9 34 2.29812309035356 0.00621927810746098 0.0388168737051875

hsa-mir-31;hsa-mir-29a;hsa-mir-135b;hsa-mir-18a;hsa-mir-195;hsa-mir-34a;hsa-mir-143;hsa-mir-34b;hsa-mir-21

hsa-mir-1179;hsa-mir-125b-2;hsa-mir-135a;hsa-mir-135b;hsa-mir-139;hsa-mir-141;hsa-mir-143;hsa-mir-145;hsa-mir-146b;hsa-mir-150;hsa-mir-17;hsa-mir-181b;hsa-mir-183;hsa-mir-185;hsa-mir-18a;hsa-mir-195;hsa-mir-196a;hsa-mir-199a;hsa-mir-199b;hsa-mir-203;hsa-mir-20a;hsa-mir-21;hsa-mir-221;hsa-mir-29a;hsa-mir-31;hsa-mir-34a;hsa-mir-34b;hsa-mir-34c;hsa-mir-486;hsa-mir-491;hsa-mir-492;hsa-mir-646;hsa-mir-92a;hsa-mir-92b

HMDD Diabetes Mellitus 79 585 3 3 7.40506329113924 0.00195276421691517 0.0172414791834949 hsa-mir-29c;hsa-mir-375;hsa-mir-503

hsa-mir-29c;hsa-mir-375;hsa-mir-503
HMDD Diabetes Mellitus, Type 2 79 585 6 12 4.0391254315305 0.000929002949168572 0.00905632082144501
hsa-mir-126;hsa-mir-24;hsa-mir-107;hsa-mir-197;hsa-mir-191;hsa-mir-21
hsa-mir-107;hsa-mir-126;hsa-mir-146a;hsa-mir-15a;hsa-mir-191;hsa-mir-197;hsa-mir-20b;hsa-mir-21;hsa-mir-223;hsa-mir-24;hsa-mir-320a;hsa-mir-486
HMDD Digestive System Neoplasms 79 585 6 8 5.55379746835443 7.73337745737408e-05 0.00147341191556285
hsa-let-7b;hsa-let-7d;hsa-let-7e;hsa-let-7i;hsa-let-7c;hsa-let-7g hsa-let-7a;hsa-let-7b;hsa-let-7c;hsa-let-7d;hsa-let-7e;hsa-let-7f;hsa-let-7g;hsa-let-7i
HMDD Endometriosis 79 585 8 22 2.82097649186257 0.00234694447575538 0.0197579976796151
hsa-mir-1;hsa-mir-200a;hsa-mir-126;hsa-mir-424;hsa-mir-99a;hsa-mir-29c;hsa-mir-196b;hsa-mir-143
hsa-mir-1;hsa-mir-100;hsa-mir-125a;hsa-mir-125b;hsa-mir-126;hsa-mir-141;hsa-mir-142;hsa-mir-143;hsa-mir-145;hsa-mir-150;hsa-mir-194;hsa-mir-196b;hsa-mir-200a;hsa-mir-200b;hsa-mir-20a;hsa-mir-223;hsa-mir-29c;hsa-mir-34c;hsa-mir-365;hsa-mir-424;hsa-mir-99a;hsa-mir-99b
HMDD Gastrointestinal Neoplasms 79 585 6 15 3.17359855334539 0.00440128371815057 0.0299562487592173
hsa-mir-200c;hsa-mir-200a;hsa-mir-31;hsa-mir-126;hsa-mir-7;hsa-let-7g
hsa-let-7g;hsa-mir-126;hsa-mir-148a;hsa-mir-152;hsa-mir-196a;hsa-mir-200a;hsa-mir-200b;hsa-mir-200c;hsa-mir-221;hsa-mir-222;hsa-mir-31;hsa-mir-338;hsa-mir-451;hsa-mir-7;hsa-mir-98
HMDD Head and Neck Neoplasms 79 585 13 30 3.56540084388186 3.65726425883791e-06 0.000137111280273707
hsa-mir-200c;hsa-mir-1;hsa-let-7b;hsa-let-7d;hsa-let-7e;hsa-let-7i;hsa-let-7c;hsa-mir-18a;hsa-mir-29c;hsa-mir-130b;hsa-let-7g;hsa-mir-181a;hsa-mir-21
hsa-let-7a;hsa-let-7b;hsa-let-7c;hsa-let-7d;hsa-let-7e;hsa-let-7f;hsa-let-7g;hsa-let-7i;hsa-mir-1;hsa-mir-130b;hsa-mir-133a;hsa-mir-142;hsa-mir-146b;hsa-mir-155;hsa-mir-15a;hsa-mir-181a;hsa-mir-181b;hsa-mir-181d;hsa-mir-18a;hsa-mir-18b;hsa-mir-193b;hsa-mir-200c;hsa-mir-204;hsa-mir-205;hsa-mir-21;hsa-mir-221;hsa-mir-29c;hsa-mir-455;hsa-mir-491;hsa-mir-499
HMDD Heart Defects, Congenital 79 585 3 5 5.55379746835443 0.00709638542744933 0.0395909718951495 hsa-mir-1;hsa-mir-26a;hsa-mir-195
hsa-mir-1;hsa-mir-133a;hsa-mir-195;hsa-mir-26a;hsa-mir-30b
HMDD Heart Failure 79 585 23 104 1.87160940325497 0.000254406840438238 0.00341093615698675
hsa-mir-200c;hsa-mir-1;hsa-let-7b;hsa-mir-126;hsa-mir-429;hsa-mir-29a;hsa-mir-24;hsa-mir-107;hsa-let-7d;hsa-mir-497;hsa-let-7e;hsa-mir-26a;hsa-mir-424;hsa-let-7c;hsa-mir-132;hsa-mir-195;hsa-mir-130b;hsa-mir-32;hsa-mir-181a;hsa-mir-133b;hsa-mir-372;hsa-mir-34b;hsa-mir-21
hsa-let-7a;hsa-let-7b;hsa-let-7c;hsa-let-7d;hsa-let-7e;hsa-let-7f;hsa-mir-1;hsa-mir-106b;hsa-mir-107;hsa-mir-10b;hsa-mir-125a;hsa-mir-126;hsa-mir-129;hsa-mir-130a;hsa-mir-130b;hsa-mir-132;hsa-mir-133a;hsa-mir-133b;hsa-mir-135a;hsa-mir-136;hsa-mir-139;hsa-mir-142;hsa-mir-148a;hsa-mir-150;hsa-mir-16;hsa-mir-17;h

sa-mir-181a;hsa-mir-182;hsa-mir-186;hsa-mir-192;hsa-mir-195;hsa-mir-196a;hsa-mir-199a;hsa-mir-199b;hsa-mir-19b;hsa-mir-200c;hsa-mir-204;hsa-mir-205;hsa-mir-208;hsa-mir-21;hsa-mir-210;hsa-mir-211;hsa-mir-212;hsa-mir-215;hsa-mir-218;hsa-mir-22;hsa-mir-23b;hsa-mir-24;hsa-mir-26a;hsa-mir-27a;hsa-mir-28;hsa-mir-296;hsa-mir-297;hsa-mir-299;hsa-mir-29a;hsa-mir-29b;hsa-mir-300;hsa-mir-302a;hsa-mir-302b;hsa-mir-302c;hsa-mir-30a;hsa-mir-30b;hsa-mir-30c;hsa-mir-30e;hsa-mir-32;hsa-mir-320;hsa-mir-325;hsa-mir-330;hsa-mir-339;hsa-mir-340;hsa-mir-342;hsa-mir-34b;hsa-mir-365;hsa-mir-367;hsa-mir-372;hsa-mir-373;hsa-mir-377;hsa-mir-381;hsa-mir-382;hsa-mir-423;hsa-mir-424;hsa-mir-429;hsa-mir-432;hsa-mir-452;hsa-mir-494;hsa-mir-497;hsa-mir-499;hsa-mir-500;hsa-mir-507;hsa-mir-512;hsa-mir-515;hsa-mir-520a;hsa-mir-520b;hsa-mir-520c;hsa-mir-520d;hsa-mir-520e;hsa-mir-520f;hsa-mir-520g;hsa-mir-520h;hsa-mir-523;hsa-mir-525;hsa-mir-526a;hsa-mir-526b;hsa-mir-98

HMDD Hepatitis C 79 585 7 17 3.23971518987342 0.00175487012878491 0.0158815746655035

hsa-mir-122;hsa-mir-1;hsa-mir-24;hsa-mir-23a;hsa-mir-30d;hsa-mir-196b;hsa-mir-638

hsa-mir-1;hsa-mir-1181;hsa-mir-122;hsa-mir-149;hsa-mir-196a;hsa-mir-196b;hsa-mir-199a;hsa-mir-23a;hsa-mir-24;hsa-mir-296;hsa-mir-30a;hsa-mir-30b;hsa-mir-30c;hsa-mir-30d;hsa-mir-431;hsa-mir-448;hsa-mir-638

HMDD Hepatitis, Chronic 79 585 5 12 3.36593785960875 0.00710887616901856 0.0395909718951495

hsa-mir-126;hsa-mir-376c;hsa-mir-224;hsa-mir-143;hsa-mir-372

hsa-mir-126;hsa-mir-143;hsa-mir-145;hsa-mir-15b;hsa-mir-182;hsa-mir-199a;hsa-mir-199b;hsa-mir-224;hsa-mir-28;hsa-mir-342;hsa-mir-372;hsa-mir-376c

HMDD Hypertrophy 79 585 8 17 4.93670886075949 1.49835085115459e-05 0.000361602005411975

hsa-mir-1;hsa-let-7b;hsa-let-7d;hsa-mir-26a;hsa-let-7i;hsa-let-7c;hsa-let-7g;hsa-mir-21

hsa-et-7a;hsa-let-7a;hsa-let-7b;hsa-let-7c;hsa-let-7d;hsa-let-7f;hsa-let-7g;hsa-let-7h;hsa-let-7i;hsa-mir-1;hsa-mir-133a;hsa-mir-206;hsa-mir-208;hsa-mir-21;hsa-mir-26a;hsa-mir-73;hsa-mir-98

HMDD Leukemia, Lymphocytic, Chronic, B-Cell 79 585 10 35 2.46835443037975 0.00211130377393684 0.0181974277658366

hsa-mir-29a;hsa-mir-24;hsa-mir-107;hsa-mir-29c;hsa-mir-195;hsa-mir-196b;hsa-mir-34a;hsa-mir-181a;hsa-mir-34b;hsa-mir-21

hsa-mir-106b;hsa-mir-107;hsa-mir-146a;hsa-mir-146b;hsa-mir-148a;hsa-mir-151;hsa-mir-155;hsa-mir-15a;hsa-mir-15b;hsa-mir-16;hsa-mir-16-1;hsa-mir-16-2;hsa-mir-181a;hsa-mir-181b;hsa-mir-181c;hsa-mir-187;hsa-mir-195;hsa-mir-196b;hsa-mir-206;hsa-mir-21;hsa-mir-22;hsa-mir-221;hsa-mir-222;hsa-mir-223;hsa-mir-23b;hsa-mir-24;hsa-mir-27b;hsa-mir-29a;hsa-mir-29b;hsa-mir-29c;hsa-mir-34a;hsa-mir-34b;hsa-mir-34c;hsa-mir-640;hsa-mir-92a

HMDD Leukemia, Myeloid, Acute 79 585 14 39 2.87974683544304 3.22363532903799e-05 0.000686444699477502

hsa-let-7b;hsa-mir-126;hsa-mir-29a;hsa-let-7d;hsa-let-7e;hsa-mir-26a;hsa-let-7i;hsa-mir-224;hsa-let-7c;hsa-mir-18a;hsa-mir-196b;hsa-let-7g;hsa-mir-181a;hsa-mir-34b

hsa-let-7a;hsa-let-7b;hsa-let-7c;hsa-let-7d;hsa-let-7e;hsa-let-7f;hsa-let-7g;hsa-let-7i;hsa-mir-125b;hsa-mir-126;hsa-mir-127;hsa-mir-146a;hsa-mir-154;hsa-mir-155;hsa-mir-15a;hsa-mir-17;hsa-mir-181a;hsa-mir-181c;hsa-mir-181d;hsa-mir-18a;hsa-mir-196b;hsa-mir-19a;hsa-mir-19b;hsa-mir-20a;hsa-mir-221;hsa-mir-222;hsa-mir-223;hsa-mir-224;hsa-mir-25;hsa-mir-26a;hsa-mir-299;hsa-mir-29a;hsa-mir-29b;hsa-mir-323;hsa-mir-34b;hsa-mir-370;hsa-mir-382;hsa-mir-92a;hsa-mir-98

HMDD Leukemia, Promyelocytic, Acute 79 585 3 6 5.55379746835443 0.00709638542744933 0.0395909718951495

hsa-mir-34a;hsa-mir-181a;hsa-mir-34b hsa-mir-181a;hsa-mir-181b;hsa-mir-181d;hsa-mir-34a;hsa-mir-34b;hsa-mir-34c

HMDD Liver Neoplasms 79 585 8 19 3.70253164556962 0.0002536720134409 0.00341093615698675

hsa-mir-122;hsa-mir-200a;hsa-mir-29a;hsa-let-7i;hsa-mir-375;hsa-mir-133b;hsa-mir-372;hsa-mir-21

hsa-let-7i;hsa-mir-10b;hsa-mir-122;hsa-mir-133b;hsa-mir-148b;hsa-mir-200a;hsa-mir-21;hsa-mir-210;hsa-mir-223;hsa-mir-24-1;hsa-mir-24-2;hsa-mir-29a;hsa-mir-30a;hsa-mir-34c;hsa-mir-372;hsa-mir-375;hsa-mir-470;hsa-mir-486;hsa-mir-629

HMDD Lung Neoplasms 79 585 33 95 2.94418179045295 3.58425849173598e-12 1.29750157400843e-09

hsa-mir-1;hsa-let-7b;hsa-mir-31;hsa-mir-126;hsa-mir-29a;hsa-mir-24;hsa-mir-103;hsa-mir-107;hsa-let-7d;hsa-mir-95;hsa-let-7e;hsa-mir-26a;hsa-let-7i;hsa-mir-214;hsa-mir-224;hsa-let-7c;hsa-mir-18a;hsa-mir-29c;hsa-mir-132;hsa-mir-30d;hsa-mir-197;hsa-mir-191;hsa-mir-34a;hsa-let-7g;hsa-mir-638;hsa-mir-216a;hsa-mir-32;hsa-mir-124;hsa-mir-181a;hsa-mir-143;hsa-mir-133b;hsa-mir-34b;hsa-mir-21

hsa-let-7a;hsa-let-7b;hsa-let-7c;hsa-let-7d;hsa-let-7e;hsa-let-7f;hsa-let-7g;hsa-let-7i;hsa-mir-1;hsa-mir-101;hsa-mir-103;hsa-mir-106a;hsa-mir-107;hsa-mir-124;hsa-mir-125a;hsa-mir-125b;hsa-mir-126;hsa-mir-128b;hsa-mir-132;hsa-mir-133b;hsa-mir-136;hsa-mir-140;hsa-mir-142;hsa-mir-143;hsa-mir-145;hsa-mir-146a;hsa-mir-146b;hsa-mir-148a;hsa-mir-150;hsa-mir-155;hsa-mir-17;hsa-mir-181a;hsa-mir-181b;hsa-mir-181c;hsa-mir-182;hsa-mir-183;hsa-mir-185;hsa-mir-186;hsa-mir-18a;hsa-mir-18b;hsa-mir-191;hsa-mir-192;hsa-mir-196a;hsa-mir-197;hsa-mir-198;hsa-mir-199a;hsa-mir-199b;hsa-mir-19a;hsa-mir-19b;hsa-mir-203;hsa-mir-205;hsa-mir-206;hsa-mir-20a;hsa-mir-21;hsa-mir-210;hsa-mir-212;hsa-mir-214;hsa-mir-216a;hsa-mir-216b;hsa-mir-218;hsa-mir-219;hsa-mir-220;hsa-mir-223;hsa-mir-224;hsa-mir-24;hsa-mir-26a;hsa-mir-27b;hsa-mir-29a;hsa-mir-29b;hsa-mir-29c;hsa-mir-301a;hsa-mir-30a;hsa-mir-30b;hsa-mir-30c;hsa-mir-30d;hsa-mir-30e;hsa-mir-31;hsa-mir-32;hsa-mir-338;hsa-mir-33a;hsa-mir-33b;hsa-mir-34a;hsa-mir-34b;hsa-mir-34c;hsa-mir-376a;hsa-mir-486;hsa-mir-499;hsa-mir-629;hsa-mir-638;hsa-mir-9;hsa-mir-923;hsa-mir-92a;hsa-mir-93;hsa-mir-95;hsa-mir-98

HMDD Lymphoma, B-Cell 79 585 7 21 3.04914370811616 0.00268197209316434 0.0215749755050109

hsa-mir-29a;hsa-mir-18a;hsa-mir-29c;hsa-mir-195;hsa-mir-34a;hsa-mir-181a;hsa-mir-21

hsa-mir-146a;hsa-mir-150;hsa-mir-155;hsa-mir-15a;hsa-mir-17;hsa-mir-181a;hsa-mir-18a;hsa-mir-195;hsa-mir-19a;hsa-mir-19b;hsa-mir-19b-1;hsa-mir-19b-2;hsa-mir-20a;hsa-mir-21;hsa-mir-22;hsa-mir-26b;hsa-mir-29a;hsa-mir-29c;hsa-mir-30e;hsa-mir-34a;hsa-mir-92a

HMDD Lymphoma, Primary Effusion 79 585 6 11 4.0391254315305 0.000929002949168572 0.00905632082144501

hsa-let-7b;hsa-let-7d;hsa-let-7e;hsa-let-7i;hsa-let-7c;hsa-let-7g

hsa-let-7a;hsa-let-7b;hsa-let-7c;hsa-let-7d;hsa-let-7e;hsa-let-7f;hsa-let-7g;hsa-let-7i;hsa-mir-221;hsa-mir-222;hsa-mir-98

HMDD Melanoma 79 585 24 105 1.85126582278481 0.000294026440660048 0.00380134183996205

hsa-mir-200c;hsa-mir-122;hsa-mir-1;hsa-mir-200a;hsa-let-7b;hsa-mir-429;hsa-mir-103;hsa-mir-135b;hsa-mir-107;hsa-let-7d;hsa-let-7e;hsa-let-7i;hsa-mir-214;hsa-mir-99a;hsa-let-7c;hsa-mir-18a;hsa-mir-30d;hsa-mir-191;hsa-mir-34a;hsa-let-7g;hsa-mir-216a;hsa-mir-625;hsa-mir-181a;hsa-mir-143

hsa-let-7a;hsa-let-7b;hsa-let-7c;hsa-let-7d;hsa-let-7e;hsa-let-7f;hsa-let-7g;hsa-let-7i;hsa-mir-1;hsa-mir-103;hsa-mir-106b;hsa-mir-107;hsa-mir-122;hsa-mir-1249;hsa-mir-125a;hsa-mir-125b;hsa-mir-1280;hsa-mir-133a;hsa-mir-135b;hsa-mir-137;hsa-mir-142;hsa-mir-143;hsa-mir-145;hsa-mir-146a;hsa-mir-146b;hsa-mir-148a;hsa-mir-151;hsa-mir-153;hsa-mir-155;hsa-mir-15b;hsa-mir-17;hsa-mir-181a;hsa-mir-182;hsa-mir-183;hsa-mir-186;hsa-mir-18a;hsa-mir-191;hsa-mir-193a;hsa-mir-193b;hsa-mir-194;hsa-mir-196a;hsa-mir-199a;hsa-mir-19a;hsa-mir-19b;hsa-mir-200a;hsa-mir-200b;hsa-mir-200c;hsa-mir-205;hsa-mir-20a;hsa-mir-20b;hsa-mir-210;hsa-mir-214;hsa-mir-215;hsa-mir-216a;hsa-mir-217;hsa-mir-218;hsa-mir-219;hsa-mir-22;hsa-mir-221;hsa-mir-222;hsa-mir-23b;hsa-mir-25;hsa-mir-296;hsa-mir-302a;hsa-mir-302b;hsa-mir-302c;hsa-mir-302d;hsa-mir-30a;hsa-mir-30b;hsa-mir-30d;hsa-mir-30e;hsa-mir-320;hsa-mir-328;hsa-mir-330;hsa-mir-338;hsa-mir-339;hsa-mir-342;hsa-mir-34a;hsa-mir-361;hsa-mir-362;hsa-mir-365;hsa-mir-367;hsa-mir-378;hsa-mir-383;hsa-mir-422a;hsa-mir-429;hsa-mir-452;hsa-mir-488;hsa-mir-501;hsa-mir-509;hsa-mir-517a;hsa-mir-518e;hsa-mir-519b;hsa-mir-532;hsa-mir-550;hsa-mir-584;hsa-mir-593;hsa-mir-621;hsa-mir-625;hsa-mir-646;hsa-mir-664;hsa-mir-767;hsa-mir-9;hsa-mir-93;hsa-mir-99a

HMDD Mesothelioma 79 585 4 9 4.23146473779385 0.00615788899290622 0.0388168737051875 hsa-mir-31;hsa-mir-29a;hsa-mir-143;hsa-mir-21

hsa-mir-106a;hsa-mir-143;hsa-mir-17;hsa-mir-193a;hsa-mir-21;hsa-mir-29a;hsa-mir-30c;hsa-mir-30e;hsa-mir-31

HMDD Muscular Disorders, Atrophic 79 585 15 48 2.36331807164018 0.000239353334795071 0.00341093615698675

hsa-mir-126;hsa-mir-376c;hsa-mir-103;hsa-mir-107;hsa-mir-497;hsa-let-7e;hsa-let-7i;hsa-mir-214;hsa-let-7c;hsa-mir-29c;hsa-mir-132;hsa-mir-195;hsa-mir-34a;hsa-mir-143;hsa-mir-21

hsa-let-7c;hsa-let-7e;hsa-let-7i;hsa-mir-100;hsa-mir-103;hsa-mir-107;hsa-mir-125a;hsa-mir-126;hsa-mir-130a;hsa-mir-132;hsa-mir-140;hsa-mir-143;hsa-mir-145;hsa-mir-146a;hsa-mir-146b;hsa-mir-148a;hsa-mir-151;hsa-mir-154;hsa-mir-155;hsa-mir-195;hsa-mir-199a;hsa-mir-199b;hsa-mir-19b;hsa-mir-21;hsa-mir-210;hsa-mir-214;hsa-mir-22;hsa-mir-221;hsa-mir-222;hsa-mir-223;hsa-mir-28;hsa-mir-299;hsa-mir-29c;hsa-mir-30a;hsa-mir-320;hsa-mir-335;hsa-mir-34a;hsa-mir-362;hsa-mir-376c;hsa-mir-379;hsa-mir-381;hsa-mir-432;hsa-mir-452;hsa-mir-487b;hsa-mir-495;hsa-mir-497;hsa-mir-501;hsa-mir-99b

HMDD Neoplasms 79 585 29 92 2.58731127039805 6.09738585923507e-09 5.51813420260774e-07

hsa-mir-200c;hsa-mir-122;hsa-mir-200a;hsa-let-7b;hsa-mir-31;hsa-mir-126;hsa-mir-429;hsa-mir-29a;hsa-mir-103;hsa-let-7d;hsa-let-7e;hsa-mir-7;hsa-let-7i;hsa-mir-214;hsa-mir-224;hsa-let-7c;hsa-mir-18a;hsa-mir-132;hsa-mir-708;hsa-mir-30d;hsa-mir-196b;hsa-mir-191;hsa-mir-34a;hsa-let-7g;hsa-mir-181a;hsa-mir-143;hsa-

mir-372;hsa-mir-34b;hsa-mir-21

hsa-let-7a;hsa-let-7b;hsa-let-7c;hsa-let-7d;hsa-let-7e;hsa-let-7f;hsa-let-7g;hsa-let-7i;hsa-mir-101;hsa-mir-103;hsa-mir-106a;hsa-mir-106b;hsa-mir-10b;hsa-mir-122;hsa-mir-1224;hsa-mir-125a;hsa-mir-125b;hsa-mir-126;hsa-mir-127;hsa-mir-132;hsa-mir-141;hsa-mir-143;hsa-mir-145;hsa-mir-146a;hsa-mir-149;hsa-mir-150;hsa-mir-151;hsa-mir-155;hsa-mir-15b;hsa-mir-16;hsa-mir-17;hsa-mir-181a;hsa-mir-181b;hsa-mir-182;hsa-mir-185;hsa-mir-18a;hsa-mir-18b;hsa-mir-191;hsa-mir-196b;hsa-mir-199a;hsa-mir-19a;hsa-mir-19b;hsa-mir-200a;hsa-mir-200b;hsa-mir-200c;hsa-mir-204;hsa-mir-205;hsa-mir-206;hsa-mir-20a;hsa-mir-20b;hsa-mir-21;hsa-mir-210;hsa-mir-214;hsa-mir-22;hsa-mir-222;hsa-mir-224;hsa-mir-25;hsa-mir-27a;hsa-mir-28;hsa-mir-29a;hsa-mir-29b;hsa-mir-302a;hsa-mir-302b;hsa-mir-302c;hsa-mir-302d;hsa-mir-30a;hsa-mir-30d;hsa-mir-30e;hsa-mir-31;hsa-mir-331;hsa-mir-335;hsa-mir-34a;hsa-mir-34b;hsa-mir-34c;hsa-mir-367;hsa-mir-371;hsa-mir-372;hsa-mir-373;hsa-mir-378;hsa-mir-429;hsa-mir-451;hsa-mir-519c;hsa-mir-520b;hsa-mir-520h;hsa-mir-532;hsa-mir-7;hsa-mir-708;hsa-mir-9;hsa-mir-92a;hsa-mir-92b;hsa-mir-93;hsa-mir-98

HMDD Ovarian Neoplasms 79 585 23 77 2.36550632911392 2.83070201933007e-06 0.000137111280273707

hsa-mir-200c;hsa-mir-1;hsa-mir-200a;hsa-let-7b;hsa-mir-31;hsa-mir-126;hsa-mir-429;hsa-mir-29a;hsa-mir-103;hsa-mir-135b;hsa-let-7d;hsa-let-7e;hsa-let-7i;hsa-mir-214;hsa-mir-224;hsa-mir-99a;hsa-let-7c;hsa-mir-18a;hsa-mir-30d;hsa-mir-191;hsa-let-7g;hsa-mir-133b;hsa-mir-21

hsa-let-7a;hsa-let-7b;hsa-let-7c;hsa-let-7d;hsa-let-7e;hsa-let-7f;hsa-let-7g;hsa-let-7i;hsa-mir-1;hsa-mir-100;hsa-mir-103;hsa-mir-106b;hsa-mir-125a;hsa-mir-125b;hsa-mir-126;hsa-mir-127;hsa-mir-130a;hsa-mir-133a;hsa-mir-133b;hsa-mir-135b;hsa-mir-141;hsa-mir-145;hsa-mir-146a;hsa-mir-151;hsa-mir-153;hsa-mir-155;hsa-mir-16;hsa-mir-17;hsa-mir-183;hsa-mir-18a;hsa-mir-191;hsa-mir-194;hsa-mir-199a;hsa-mir-19a;hsa-mir-19b;hsa-mir-200a;hsa-mir-200b;hsa-mir-200c;hsa-mir-20a;hsa-mir-21;hsa-mir-214;hsa-mir-215;hsa-mir-218;hsa-mir-219;hsa-mir-22;hsa-mir-223;hsa-mir-224;hsa-mir-25;hsa-mir-27a;hsa-mir-296;hsa-mir-29a;hsa-mir-302a;hsa-mir-302b;hsa-mir-302c;hsa-mir-302d;hsa-mir-30b;hsa-mir-30c;hsa-mir-30d;hsa-mir-31;hsa-mir-320;hsa-mir-335;hsa-mir-338;hsa-mir-339;hsa-mir-34c;hsa-mir-367;hsa-mir-383;hsa-mir-429;hsa-mir-486;hsa-mir-488;hsa-mir-499;hsa-mir-516a;hsa-mir-629;hsa-mir-9;hsa-mir-92b;hsa-mir-93;hsa-mir-99a;hsa-mir-99b

HMDD Overian Neoplasms 79 585 3 4 5.55379746835443 0.00709638542744933 0.0395909718951495 hsa-mir-376c;hsa-mir-34a;hsa-mir-34b

hsa-mir-34a;hsa-mir-34b;hsa-mir-34c;hsa-mir-376c

HMDD Pancreatic Neoplasms 79 585 21 53 3.38057237204183 5.47032973591309e-09 5.51813420260774e-07

hsa-mir-200c;hsa-mir-200a;hsa-let-7b;hsa-mir-24;hsa-mir-107;hsa-let-7d;hsa-mir-95;hsa-let-7e;hsa-let-7i;hsa-mir-214;hsa-mir-224;hsa-let-7c;hsa-mir-132;hsa-mir-191;hsa-mir-34a;hsa-let-7g;hsa-mir-32;hsa-mir-143;hsa-mir-133b;hsa-mir-34b;hsa-mir-21

hsa-let-7a;hsa-let-7b;hsa-let-7c;hsa-let-7d;hsa-let-7e;hsa-let-7f;hsa-let-7g;hsa-let-7i;hsa-mir-106a;hsa-mir-107;hsa-mir-128b;hsa-mir-132;hsa-mir-133b;hsa-mir-143;hsa-mir-145;hsa-mir-146a;hsa-mir-146b;hsa-mir-155;hsa-mir-15a;hsa-mir-15b;hsa-mir-17;hsa-mir-181b;hsa-mir-186;hsa-mir-190;hsa-mir-191;hsa-mir-196a;hsa-mir-199a;hsa-mir-200a;hsa-mir-200b;hsa-mir-200c;hsa-mir-203;hsa-mir-20a;hsa-mir-21;hsa-mir-210;hsa-mir-214;hsa-mir-217;hsa-mir-221;hsa-mir-222;hsa-mir-22

3;hsa-mir-224;hsa-mir-24;hsa-mir-25;hsa-mir-27a;hsa-mir-29b;hsa-mir-30c;hsa-mir-32;hsa-mir-34a;hsa-mir-34b;hsa-mir-34c;hsa-mir-486;hsa-mir-520h;hsa-mir-95;hsa-mir-96

HMDD Parkinson Disease 79 585 6 11 4.44303797468354 0.000469342957609485 0.00514855001983738

hsa-mir-1;hsa-mir-29a;hsa-mir-26a;hsa-mir-29c;hsa-mir-195;hsa-mir-133b

hsa-mir-1;hsa-mir-133b;hsa-mir-16;hsa-mir-195;hsa-mir-22;hsa-mir-26a;hsa-mir-29a;hsa-mir-29b;hsa-mir-29c;hsa-mir-30a;hsa-mir-433

HMDD Pituitary Neoplasms 79 585 6 11 4.44303797468354 0.000469342957609485 0.00514855001983738

hsa-let-7b;hsa-let-7d;hsa-let-7e;hsa-let-7i;hsa-let-7c;hsa-let-7g

hsa-let-7a;hsa-let-7b;hsa-let-7c;hsa-let-7d;hsa-let-7e;hsa-let-7f;hsa-let-7g;hsa-let-7i;hsa-mir-15a;hsa-mir-15b;hsa-mir-16

HMDD Precursor Cell Lymphoblastic Leukemia-Lymphoma 79 585 5 12 3.36593785960875 0.00710887616901856 0.0395909718951495

hsa-mir-126;hsa-mir-29a;hsa-mir-29c;hsa-mir-34a;hsa-mir-181a

hsa-mir-126;hsa-mir-128;hsa-mir-146a;hsa-mir-17;hsa-mir-181a;hsa-mir-181c;hsa-mir-221;hsa-mir-223;hsa-mir-29a;hsa-mir-29b;hsa-mir-29c;hsa-mir-34a

HMDD Retinoblastoma 79 585 6 8 5.55379746835443 7.73337745737408e-05 0.00147341191556285

hsa-let-7b;hsa-let-7d;hsa-let-7e;hsa-let-7i;hsa-let-7c;hsa-let-7g hsa-let-7a;hsa-let-7b;hsa-let-7c;hsa-let-7d;hsa-let-7e;hsa-let-7f;hsa-let-7g;hsa-let-7i

HMDD Sarcoma, Kaposi 79 585 6 11 4.0391254315305 0.000929002949168572 0.00905632082144501

hsa-let-7b;hsa-let-7d;hsa-let-7e;hsa-let-7i;hsa-let-7c;hsa-let-7g

hsa-let-7a;hsa-let-7b;hsa-let-7c;hsa-let-7d;hsa-let-7e;hsa-let-7f;hsa-let-7g;hsa-let-7i;hsa-mir-221;hsa-mir-222;hsa-mir-98

HMDD Stomach Neoplasms 79 585 18 56 2.7202273314389 4.84229527217088e-06 0.000146075907377155

hsa-mir-31;hsa-mir-126;hsa-mir-24;hsa-mir-103;hsa-mir-107;hsa-mir-497;hsa-mir-214;hsa-mir-18a;hsa-mir-132;hsa-mir-195;hsa-mir-130b;hsa-mir-375;hsa-mir-191;hsa-mir-34a;hsa-let-7g;hsa-mir-133b;hsa-mir-34b;hsa-mir-21

hsa-let-7g;hsa-mir-100;hsa-mir-103;hsa-mir-106a;hsa-mir-106b;hsa-mir-107;hsa-mir-126;hsa-mir-129;hsa-mir-130b;hsa-mir-132;hsa-mir-133b;hsa-mir-139;hsa-mir-141;hsa-mir-145;hsa-mir-146a;hsa-mir-148a;hsa-mir-148b;hsa-mir-150;hsa-mir-155;hsa-mir-17;hsa-mir-181b;hsa-mir-181c;hsa-mir-18a;hsa-mir-18b;hsa-mir-191;hsa-mir-195;hsa-mir-196a;hsa-mir-19a;hsa-mir-20a;hsa-mir-20b;hsa-mir-21;hsa-mir-212;hsa-mir-214;hsa-mir-218;hsa-mir-221;hsa-mir-222;hsa-mir-223;hsa-mir-24;hsa-mir-25;hsa-mir-27a;hsa-mir-31;hsa-mir-331;hsa-mir-340;hsa-mir-34a;hsa-mir-34b;hsa-mir-34c;hsa-mir-375;hsa-mir-378;hsa-mir-421;hsa-mir-433;hsa-mir-43c;hsa-mir-497;hsa-mir-650;hsa-mir-658;hsa-mir-9;hsa-mir-93

HMDD Urinary Bladder Neoplasms 79 585 9 34 2.46835443037975 0.00360819484678182 0.0261233306907004

hsa-mir-1;hsa-mir-99a;hsa-mir-708;hsa-mir-34a;hsa-mir-503;hsa-mir-143;hsa-mir-133b;hsa-mir-34b;hsa-mir-21

hsa-mir-1;hsa-mir-101;hsa-mir-1-2;hsa-mir-1224;hsa-mir-1227;hsa-mir-1229;hsa-mir-125b;hsa-mir-127;hsa-mir-133a;hsa-mir-133b;hsa-mir-143;hsa-mir-145;hsa-mir-149;hsa-mir-181b;hsa-mir-199b;hsa-mir-200b;hsa-mir-203;hsa-mir-205;hsa-mir-21;hsa-mir-210;hsa-mir-212;hsa-mir-29b-1;hsa-mir-300;hsa-mir-328;hsa-mir-34a;hsa-mir-34b;hsa-mir-34c;hsa-mir-503;hsa-mir-708;hsa-mir-9;hsa-mir-923;hsa-mir-93;hsa-mir-99a;hsa-mir-99b

HMDD Vascular Diseases 79 585 4 8 4.93670886075949 0.00291866234203418 0.0220115784961745

hsa-mir-126;hsa-mir-23a;hsa-mir-18a;hsa-mir-21 hsa-mir-126;hsa-mir-145;hsa-mir-17;hsa-mir-18a;hsa-mir-19a;hsa-mir-20a;hsa-mir-21;hsa-mir-23a

Function anti-cell proliferation(Hwang etal BJC2006) 79 585 6 11 4.44303797468354 0.000469342957609485 0.00514855001983738

hsa-let-7b;hsa-let-7d;hsa-let-7e;hsa-let-7i;hsa-let-7c;hsa-let-7g

hsa-let-7a;hsa-let-7b;hsa-let-7c;hsa-let-7d;hsa-let-7e;hsa-let-7f;hsa-mir-98;hsa-let-7g;hsa-let-7i;hsa-mir-15a;hsa-mir-16

Function Apoptosis 79 585 15 44 3.17359855334539 3.65737179529576e-06 0.000137111280273707

hsa-mir-144;hsa-mir-29a;hsa-mir-7;hsa-mir-26a;hsa-let-7c;hsa-mir-18a;hsa-mir-29c;hsa-mir-195;hsa-mir-138;hsa-mir-34a;hsa-let-7g;hsa-mir-181a;hsa-mir-372;hsa-mir-34b;hsa-mir-21

hsa-let-7c;hsa-let-7g;hsa-mir-10a;hsa-mir-138;hsa-mir-144;hsa-mir-146a;hsa-mir-148a;hsa-mir-150;hsa-mir-155;hsa-mir-15a;hsa-mir-15b;hsa-mir-16;hsa-mir-17;hsa-mir-181a;hsa-mir-182;hsa-mir-18a;hsa-mir-19;hsa-mir-193;hsa-mir-195;hsa-mir-19a;hsa-mir-19b;hsa-mir-204;hsa-mir-20a;hsa-mir-21;hsa-mir-210;hsa-mir-216;hsa-mir-221;hsa-mir-222;hsa-mir-26a;hsa-mir-27a;hsa-mir-29a;hsa-mir-29b;hsa-mir-29c;hsa-mir-34a;hsa-mir-34b;hsa-mir-34c;hsa-mir-372;hsa-mir-449a;hsa-mir-449b;hsa-mir-494;hsa-mir-7;hsa-mir-92a;hsa-mir-96;hsa-mir-9a

Function Cell cycle related 79 585 19 68 2.65464533078577 3.78760442745047e-06 0.000137111280273707

hsa-mir-200c;hsa-mir-122;hsa-mir-1;hsa-let-7b;hsa-mir-31;hsa-mir-24;hsa-mir-107;hsa-mir-424;hsa-mir-18a;hsa-mir-195;hsa-mir-138;hsa-mir-34a;hsa-let-7g;hsa-mir-503;hsa-mir-124;hsa-mir-143;hsa-mir-372;hsa-mir-34b;hsa-mir-21

hsa-let-7a;hsa-let-7b;hsa-let-7g;hsa-mir-1;hsa-mir-107;hsa-mir-122;hsa-mir-124;hsa-mir-124a;hsa-mir-125b;hsa-mir-138;hsa-mir-140;hsa-mir-141;hsa-mir-143;hsa-mir-145;hsa-mir-145;hsa-mir-15;hsa-mir-150;hsa-mir-155;hsa-mir-15a;hsa-mir-15b;hsa-mir-16;hsa-mir-17;hsa-mir-182;hsa-mir-185;hsa-mir-18a;hsa-mir-195;hsa-mir-196a;hsa-mir-19a;hsa-mir-19b;hsa-mir-200b;hsa-mir-200c;hsa-mir-205;hsa-mir-206;hsa-mir-20a;hsa-mir-21;hsa-mir-210;hsa-mir-221;hsa-mir-222;hsa-mir-223;hsa-mir-24;hsa-mir-27a;hsa-mir-27b;hsa-mir-29b;hsa-mir-31;hsa-mir-320;hsa-mir-331;hsa-mir-33b;hsa-mir-34;hsa-mir-34a;hsa-mir-34b;hsa-mir-34c;hsa-mir-372;hsa-mir-372;hsa-mir-373;hsa-mir-376b;hsa-mir-424;hsa-mir-449;hsa-mir-449a;hsa-mir-449b;hsa-mir-494;hsa-mir-499;hsa-mir-503;hsa-mir-9;hsa-mir-92;hsa-mir-92a;hsa-mir-92b;hsa-mir-96;hsa-mir-98

Function Hormones regulation 79 585 15 62 2.36331807164018 0.000239353334795071 0.00341093615698675

hsa-mir-1;hsa-mir-126;hsa-mir-29a;hsa-mir-24;hsa-mir-135b;hsa-mir-107;hsa-mir-23a;hsa-mir-7;hsa-mir-18a;hsa-mir-29c;hsa-mir-132;hsa-mir-30d;hsa-mir-375

;hsa-mir-133b;hsa-mir-21

hsa-let-7a;hsa-mir-1;hsa-mir-101;hsa-mir-106a;hsa-mir-106b;hsa-mir-107;hsa-mir-124a;hsa-mir-126;hsa-mir-128a;hsa-mir-128b;hsa-mir-132;hsa-mir-133a;hsa-mir-133b;hsa-mir-135a;hsa-mir-135b;hsa-mir-139;hsa-mir-146a;hsa-mir-150;hsa-mir-15a;hsa-mir-16;hsa-mir-17;hsa-mir-18;hsa-mir-18a;hsa-mir-18b;hsa-mir-199;hsa-mir-19a;hsa-mir-19b;hsa-mir-206;hsa-mir-208a;hsa-mir-208b;hsa-mir-20a;hsa-mir-20b;hsa-mir-21;hsa-mir-212;hsa-mir-22;hsa-mir-221;hsa-mir-222;hsa-mir-223;hsa-mir-23a;hsa-mir-23b;hsa-mir-24;hsa-mir-25;hsa-mir-27a;hsa-mir-27b;hsa-mir-296;hsa-mir-29a;hsa-mir-29b;hsa-mir-29c;hsa-mir-30d;hsa-mir-34;hsa-mir-346;hsa-mir-363;hsa-mir-375;hsa-mir-449;hsa-mir-484;hsa-mir-499;hsa-mir-690;hsa-mir-7;hsa-mir-9;hsa-mir-92a;hsa-mir-93;hsa-mir-98

Function Human embryonic stem cell (hESC) regulation 79 585 18 85 2.08267405063291 0.000420585157621423 0.00514855001983738

hsa-mir-200c;hsa-mir-122;hsa-mir-31;hsa-mir-126;hsa-mir-429;hsa-mir-144;hsa-mir-24;hsa-mir-135b;hsa-mir-26a;hsa-mir-214;hsa-mir-132;hsa-mir-195;hsa-mir-326;hsa-mir-34a;hsa-mir-181a;hsa-mir-143;hsa-mir-372;hsa-mir-21

hsa-mir-106a;hsa-mir-106b;hsa-mir-10a;hsa-mir-122;hsa-mir-124a;hsa-mir-125a;hsa-mir-125b;hsa-mir-126;hsa-mir-128;hsa-mir-132;hsa-mir-133;hsa-mir-134;hsa-mir-135b;hsa-mir-137;hsa-mir-141;hsa-mir-142;hsa-mir-143;hsa-mir-144;hsa-mir-145;hsa-mir-146a;hsa-mir-146b;hsa-mir-148a;hsa-mir-148b;hsa-mir-150;hsa-mir-155;hsa-mir-15b;hsa-mir-17;hsa-mir-181a;hsa-mir-182;hsa-mir-192;hsa-mir-195;hsa-mir-196a;hsa-mir-199a;hsa-mir-199b;hsa-mir-200;hsa-mir-200c;hsa-mir-203;hsa-mir-20a;hsa-mir-21;hsa-mir-210;hsa-mir-214;hsa-mir-218;hsa-mir-221;hsa-mir-222;hsa-mir-223;hsa-mir-24;hsa-mir-25;hsa-mir-26a;hsa-mir-26b;hsa-mir-278;hsa-mir-27a;hsa-mir-290;hsa-mir-291;hsa-mir-294;hsa-mir-295;hsa-mir-296;hsa-mir-302a;hsa-mir-302b;hsa-mir-302c;hsa-mir-302d;hsa-mir-309;hsa-mir-30b;hsa-mir-30c;hsa-mir-31;hsa-mir-322;hsa-mir-323;hsa-mir-326;hsa-mir-34;hsa-mir-346;hsa-mir-34a;hsa-mir-367;hsa-mir-369;hsa-mir-371;hsa-mir-372;hsa-mir-373;hsa-mir-379;hsa-mir-427;hsa-mir-429;hsa-mir-430;hsa-mir-451;hsa-mir-470;hsa-mir-486;hsa-mir-489;hsa-mir-9;hsa-mir-93

Function Immune response 79 585 12 48 2.40164214847759 0.000950663511643398 0.00905632082144501

hsa-mir-31;hsa-mir-126;hsa-mir-103;hsa-let-7i;hsa-mir-424;hsa-mir-18a;hsa-mir-132;hsa-let-7g;hsa-mir-181a;hsa-mir-143;hsa-mir-147;hsa-mir-21

hsa-let-7g;hsa-let-7i;hsa-mir-100;hsa-mir-101;hsa-mir-105;hsa-mir-125b;hsa-mir-126;hsa-mir-132;hsa-mir-140;hsa-mir-143;hsa-mir-146a;hsa-mir-146b;hsa-mir-147;hsa-mir-150;hsa-mir-155;hsa-mir-15a;hsa-mir-16;hsa-mir-17;hsa-mir-181;hsa-mir-181a;hsa-mir-186;hsa-mir-188;hsa-mir-18a;hsa-mir-192;hsa-mir-196;hsa-mir-199;hsa-mir-19a;hsa-mir-19b;hsa-mir-203;hsa-mir-20a;hsa-mir-21;hsa-mir-223;hsa-mir-25;hsa-mir-27b;hsa-mir-31;hsa-mir-320;hsa-mir-328;hsa-mir-342;hsa-mir-370;hsa-mir-424;hsa-mir-449b;hsa-mir-500;hsa-mir-590;hsa-mir-710;hsa-mir-9;hsa-mir-92a;hsa-mir-98;hsa-mir-103

Function Inflammation 79 585 9 41 2.29812309035356 0.00621927810746098 0.0388168737051875

hsa-mir-1;hsa-mir-31;hsa-mir-126;hsa-mir-132;hsa-let-7g;hsa-mir-181a;hsa-mir-143;hsa-mir-147;hsa-mir-21

hsa-let-7g;hsa-mir-1;hsa-mir-100;hsa-mir-105;hsa-mir-1224;hsa-mir-125b;hsa-mir-126;hsa-mir-132;hsa-mir-133a;hsa-mir-140;hsa-mir-143;hsa-mir-146a;hsa-mir-146b;hsa-mir-147;hsa-mir-150;hsa-mir-155;hsa-mir-17;hsa-mir-181a;hsa-mir-182;hsa-mir-183;hsa-mir-188;hsa-mir-192;hsa-mir-196;hsa-mir-199;hsa-mir-199a;hs

a-mir-203;hsa-mir-21;hsa-mir-223;hsa-mir-25;hsa-mir-27b;hsa-mir-31;hsa-mir-320;hsa-mir-328;hsa-mir-342;hsa-mir-370;hsa-mir-449b;hsa-mir-500;hsa-mir-705;hsa-mir-710;hsa-mir-9;hsa-mir-98

Function Muscle development 79 585 5 11 3.70253164556962 0.00429984288726856 0.0299562487592173
hsa-mir-1;hsa-mir-24;hsa-mir-23a;hsa-mir-124;hsa-mir-133b
hsa-mir-1;hsa-mir-24;hsa-mir-206;hsa-mir-222;hsa-mir-221;hsa-mir-124;hsa-mir-499;hsa-mir-23a;hsa-mir-208b;hsa-mir-133a;hsa-mir-133b

Function Folliculogenesis 79 585 4 7 4.23146473779385 0.00615788899290622 0.0388168737051875 hsa-let-7b;hsa-let-7c;hsa-mir-143;hsa-mir-21
hsa-mir-143;hsa-let-7a;hsa-mir-125b;hsa-let-7b;hsa-let-7c;hsa-mir-21;hsa-mir-15b

Function lipid metabolism 79 585 7 20 3.04914370811616 0.00268197209316434 0.0215749755050109
hsa-mir-122;hsa-mir-103;hsa-mir-107;hsa-mir-196b;hsa-mir-375;hsa-mir-34a;hsa-mir-143
hsa-mir-122;hsa-mir-33a;hsa-mir-33b;hsa-mir-370;hsa-mir-375;hsa-mir-335;hsa-mir-378;hsa-mir-27a;hsa-mir-27b;hsa-mir-125a;hsa-mir-103;hsa-mir-196a;hsa-mir-196b;hsa-mir-296;hsa-mir-98;hsa-mir-143;hsa-mir-107;hsa-mir-34a;hsa-mir-181d;hsa-mir-10b

Function hematopoiesis 79 585 10 31 2.84810126582278 0.000574004696419560 0.0061114617677612
hsa-mir-1;hsa-mir-126;hsa-mir-29a;hsa-mir-103;hsa-mir-18a;hsa-mir-29c;hsa-mir-196b;hsa-mir-34a;hsa-mir-181a;hsa-mir-143
hsa-mir-103;hsa-mir-143;hsa-mir-145;hsa-mir-378;hsa-mir-146a;hsa-mir-126;hsa-mir-125b;hsa-mir-223;hsa-mir-142;hsa-mir-1;hsa-mir-133a;hsa-mir-34a;hsa-mir-196a;hsa-mir-196b;hsa-mir-221;hsa-mir-222;hsa-mir-17;hsa-mir-18a;hsa-mir-19a;hsa-mir-19b;hsa-mir-20a;hsa-mir-92a;hsa-mir-155;hsa-mir-150;hsa-mir-181a;hsa-mir-15a;hsa-mir-29c;hsa-mir-302;hsa-mir-98;hsa-mir-29a;hsa-let-7a

Function adipocyte differentiation 79 585 10 27 3.21959273527793 0.000170670197786741 0.00303981457886022
hsa-let-7b;hsa-mir-103;hsa-let-7d;hsa-let-7e;hsa-let-7i;hsa-let-7c;hsa-mir-18a;hsa-mir-375;hsa-let-7g;hsa-mir-143
hsa-mir-103;hsa-mir-143;hsa-mir-375;hsa-mir-130a;hsa-mir-221;hsa-mir-222;hsa-mir-448;hsa-mir-378;hsa-mir-27a;hsa-mir-27b;hsa-mir-204;hsa-mir-211;hsa-let-7a;hsa-let-7b;hsa-let-7c;hsa-let-7d;hsa-let-7e;hsa-let-7f;hsa-let-7g;hsa-let-7h;hsa-let-7i;hsa-mir-17;hsa-mir-18a;hsa-mir-19a;hsa-mir-19b;hsa-mir-20a;hsa-mir-92a

Function carbohydrate metabolism 79 585 4 7 4.93670886075949 0.00291866234203418 0.0220115784961745
hsa-mir-122;hsa-mir-200a;hsa-mir-429;hsa-mir-21 hsa-mir-122;hsa-mir-451;hsa-mir-27;hsa-mir-200a;hsa-mir-200b;hsa-mir-429;hsa-mir-21

Function cell death 79 585 20 55 3.21959273527793 4.03847764240907e-08 2.43654817758681e-06
hsa-mir-1;hsa-let-7b;hsa-mir-376c;hsa-let-7d;hsa-mir-497;hsa-let-7e;hsa-mir-7;hsa-let-7i;hsa-let-7c;hsa-mir-18a;hsa-mir-30d;hsa-mir-1226;hsa-mir-130b;hsa-mir-380;hsa-mir-34a;hsa-let-7g;hsa-mir-181a;hsa-mir-143;hsa-mir-21;hsa-mir-630
hsa-mir-16;hsa-mir-184;hsa-mir-205;hsa-mir-125b;hsa-mir-129;hsa-mir-1;hsa-mir-206;hsa-mir-513;hsa-mir-7;hsa-mir-143;hsa-mir-145;hsa-mir-155;hsa-mir-12

26;hsa-mir-130b;hsa-mir-146a;hsa-mir-14;hsa-mir-212;hsa-mir-380;hsa-mir-497;hsa-mir-494;hsa-mir-21;hsa-mir-181b;hsa-mir-34a;hsa-mir-182;hsa-mir-183;hsa-mir-128;hsa-mir-630;hsa-mir-221;hsa-mir-222;hsa-mir-210;hsa-mir-203;hsa-mir-10b;hsa-mir-23b;hsa-mir-885;hsa-mir-181a;hsa-mir-886;hsa-let-7a;hsa-let-7b;hsa-let-7c;hsa-let-7d;hsa-let-7e;hsa-let-7f;hsa-let-7g;hsa-let-7h;hsa-let-7i;hsa-mir-98;hsa-mir-376c;hsa-mir-17;hsa-mir-18a;hsa-mir-19a;hsa-mir-19b;hsa-mir-20a;hsa-mir-92a;hsa-mir-25;hsa-mir-30d

Function cell division 79 585 9 17 4.76039783001808 6.22849936863428e-06 0.000161051197960401

hsa-let-7b;hsa-mir-107;hsa-let-7d;hsa-let-7e;hsa-let-7i;hsa-let-7c;hsa-mir-34a;hsa-let-7g;hsa-mir-124

hsa-mir-15a;hsa-mir-107;hsa-mir-124;hsa-mir-519;hsa-mir-16;hsa-mir-34a;hsa-mir-106b;hsa-let-7a;hsa-let-7b;hsa-let-7c;hsa-let-7d;hsa-let-7e;hsa-let-7f;hsa-let-7g;hsa-let-7h;hsa-let-7i;hsa-mir-27a

Function heart development 79 585 3 7 5.55379746835443 0.00709638542744933 0.0395909718951495 hsa-mir-1;hsa-mir-133b;hsa-mir-21

hsa-mir-1;hsa-mir-133a;hsa-mir-133b;hsa-mir-27b;hsa-mir-208;hsa-mir-499;hsa-mir-21

Function Epithelial-mesenchymal transition 79 585 15 41 2.84810126582278 1.86168525764253e-05 0.000421206289541622

hsa-mir-200c;hsa-mir-200a;hsa-let-7b;hsa-mir-31;hsa-mir-429;hsa-mir-144;hsa-mir-29a;hsa-mir-103;hsa-mir-107;hsa-let-7d;hsa-let-7e;hsa-let-7c;hsa-mir-29c;hsa-mir-30d;hsa-mir-21

hsa-mir-200a;hsa-mir-200b;hsa-mir-200c;hsa-mir-141;hsa-mir-203;hsa-mir-137;hsa-mir-429;hsa-let-7a;hsa-mir-144;hsa-mir-205;hsa-mir-23b;hsa-mir-29c;hsa-mir-370;hsa-mir-155;hsa-mir-369;hsa-mir-450;hsa-mir-542;hsa-mir-30a;hsa-mir-30b;hsa-mir-30c;hsa-mir-30d;hsa-mir-30e;hsa-mir-1915;hsa-mir-1909;hsa-mir-194;hsa-mir-21;hsa-mir-31;hsa-mir-448;hsa-mir-382;hsa-mir-103;hsa-mir-107;hsa-mir-661;hsa-mir-221;hsa-mir-192;hsa-mir-215;hsa-mir-125a;hsa-let-7b;hsa-let-7c;hsa-let-7d;hsa-let-7e;hsa-mir-29a

Function miRNA tumor suppressors 79 585 20 37 4.23146473779385 6.9020201239442e-11 1.2492656424339e-08

hsa-mir-200c;hsa-mir-122;hsa-mir-1;hsa-mir-200a;hsa-let-7b;hsa-mir-126;hsa-mir-29a;hsa-let-7d;hsa-let-7e;hsa-mir-26a;hsa-let-7i;hsa-let-7c;hsa-mir-29c;hsa-mir-195;hsa-mir-34a;hsa-let-7g;hsa-mir-124;hsa-mir-181a;hsa-mir-143;hsa-mir-34b

hsa-mir-101;hsa-mir-1;hsa-mir-122;hsa-mir-126;hsa-mir-141;hsa-mir-200a;hsa-mir-200b;hsa-mir-200c;hsa-mir-451;hsa-let-7a;hsa-let-7b;hsa-let-7c;hsa-let-7d;hsa-let-7e;hsa-let-7f;hsa-let-7g;hsa-let-7i;hsa-mir-124;hsa-mir-125a;hsa-mir-125b;hsa-mir-127;hsa-mir-143;hsa-mir-145;hsa-mir-15a;hsa-mir-16;hsa-mir-181a;hsa-mir-181b;hsa-mir-181c;hsa-mir-195;hsa-mir-26a;hsa-mir-26b;hsa-mir-29a;hsa-mir-29c;hsa-mir-34a;hsa-mir-34b;hsa-mir-34c;hsa-mir-98
